# Mutational Biases Drive Elevated Rates of Substitution at Regulatory Sites across Cancer Types

**DOI:** 10.1371/journal.pgen.1006207

**Published:** 2016-08-04

**Authors:** Vera B. Kaiser, Martin S. Taylor, Colin A. Semple

**Affiliations:** MRC Human Genetics Unit, MRC Institute of Genetics and Molecular Medicine, University of Edinburgh, Western General Hospital, Edinburgh, United Kingdom; Broad Institute, UNITED STATES

## Abstract

Disruption of gene regulation is known to play major roles in carcinogenesis and tumour progression. Here, we comprehensively characterize the mutational profiles of diverse transcription factor binding sites (TFBSs) across 1,574 completely sequenced cancer genomes encompassing 11 tumour types. We assess the relative rates and impact of the mutational burden at the binding sites of 81 transcription factors (TFs), by comparing the abundance and patterns of single base substitutions within putatively functional binding sites to control sites with matched sequence composition. There is a strong (1.43-fold) and significant excess of mutations at functional binding sites across TFs, and the mutations that accumulate in cancers are typically more disruptive than variants tolerated in extant human populations at the same sites. CTCF binding sites suffer an exceptionally high mutational load in cancer (3.31-fold excess) relative to control sites, and we demonstrate for the first time that this effect is seen in essentially all cancer types with sufficient data. The sub-set of CTCF sites involved in higher order chromatin structures has the highest mutational burden, suggesting a widespread breakdown of chromatin organization. However, we find no evidence for selection driving these distinctive patterns of mutation. The mutational load at CTCF-binding sites is substantially determined by replication timing and the mutational signature of the tumor in question, suggesting that selectively neutral processes underlie the unusual mutation patterns. Pervasive hyper-mutation within transcription factor binding sites rewires the regulatory landscape of the cancer genome, but it is dominated by mutational processes rather than selection.

## Author Summary

Regulatory regions of the genome are important players in cancer initiation and progression. Here, we study the patterns of mutations accumulating at short DNA segments bound by regulatory proteins (transcription factor binding sites) across many cancer types and in the human population. We find strikingly high rates of mutation at active regulatory sites across different cancers, relative to matched control sequences. This excess of mutations disrupts the binding sites of particular factors, such as CTCF, and is likely to be driven by selectively neutral processes, such as the replication timing of the genomic regions concerned. However, binding sites involved in regulatory chromatin structures suffer particularly high levels of mutation, suggesting the frequent disruption of such structures in cancers.

## Introduction

Most large-scale surveys of somatic mutation in cancer have focussed on protein-coding sequences, and catalogues of genes that carry recurrent mutations already number in the hundreds [1–3], but it has long been speculated that driver mutations are likely to exist in the 98% of the genome sequence outside protein-coding exons [4]. The landscape of somatic mutation in cancer is complex, whole genome sequencing (WGS) data have revealed variable mutational spectra across cancers, some associated with particular mutagens, some with defects in DNA repair or replication fidelity, and others with unknown etiology [5]. In spite of this, cancers can be classified based upon the constellations of genomic, epigenomic and transcriptomic features they possess, indicating broad changes in regulation during tumour evolution [6].

Over the past decade, our view of transcriptional regulation in the human genome has changed radically as large consortia have profiled chromatin features across multiple cell types [7], including extensive catalogues of active regulatory elements [8]. At the same time, new technologies have allowed the exploration of chromatin conformation within nuclei, revealing maps of three-dimensional nuclear architecture, e.g. Rao *et al*. [9]. The most recent studies of WGS data derived from tumours have made use of these new perspectives, studying patterns of recurrent mutations in putatively functional regulatory sites [10–12]. However, accurately detecting elevated rates of mutation at relatively small numbers of regulatory sites presents major challenges for analysis. Firstly, there are wide variations in the mutational spectra experienced by different cancer types and individual tumours [2]. Secondly, the success of searches for recurrently mutated genomic regions is heavily dependent upon the number of samples available, and even large studies have proved under-powered to detect known hotspots at regulatory loci [11]. Thirdly, the reliable detection of elevated mutation at particular sites requires careful comparisons with control sites, accounting for the features associated with the sites under scrutiny, such as nucleotide composition, fine scale chromatin accessibility and replication timing [11,13]. Some studies of mutation at regulatory sites have suffered from low sample sizes per cancer type but were still able to identify a number of recurrently mutated promoters [14], for example the telomerase reverse transcriptase (TERT) gene in melanomas [15].

Predicting the functional impact of mutations occurring within noncoding regions also remains challenging. Studies of coding sequence variation in cancers have often sought evidence for variants subject to positive selection as a proxy for functional significance [3]. However, this is complicated by a widespread increase in functional (nonsynonymous) mutations, reflecting the relaxation of purifying selection in cancers relative to the germline [16]. Current strategies include the use of regions annotated as functional based upon ChIP-seq data that is restricted to a small fraction of DNA binding proteins [10], and the use of regulatory compendia scores [11]. Robust measures of selection traditionally use comparisons of putatively functional and non-functional sites (e.g. nonsynonymous and synonymous sites), but this has been lacking in studies of selection at regulatory sites in cancer.

Here, we exploit the unprecedented volumes of data produced recently by cancer WGS projects [5,17] and examine the likely functional consequences of mutations at regulatory sites. We develop novel approaches to explore the strength and directionality of selection exercised at these sites, controlling for the mutational spectra seen across cancer types and the variation in mutation rates across the human genome. Significant enrichments of somatic mutations are evident at the binding sites of several transcription factors, particularly CTCF, pointing to elevated mutation rates or suppressed surveillance and repair. These enrichments disproportionately involve mutations predicted to weaken or abolish binding at functional regulatory sites, and we find little evidence for selection preserving binding sites in cancer. However, we discover mutational foci across cancers that are predicted to alter chromatin organisation, and intriguing differences emerge in the patterns and extent of regulatory disruption seen between cancer types.

## Results

### Functional TFBSs are enriched for mutations across transcription factors and cancers

We compiled a total of 9,958,580 somatic single base substitutions across 1,574 tumour samples from 11 different tumour types; consistent with previous studies [2,5], there was a high degree of variation in substitution rates amongst tumour types (Table 1). DNase hypersensitive sites containing sequence-specific transcription factor (TF) binding motifs have previously been shown to closely match signals obtained from Chip-Seq data and can hence be used as a proxy for TF occupancy [18–20]. We established the genomic locations for constitutive DNase hypersensitive sites, active in most cell types, spanning a total of 3.92MB in the human reference genome (see Methods section). Next, we scanned the genome for matches to 118 known binding motifs of 81 transcription factors, and those motif matches inside constitutive DNase regions were labeled as “putatively functional” TFBSs. We found a total of 197,374 functional TFBSs (S1 Dataset), spanning 1.39MB of the genome and containing a total of 4,782 somatic mutations across the 11 cancer types (Table 1). For each motif matrix, we also compiled a list of control TFBSs, i.e. sequences that match a given TF binding motif, but are located outside any regions of open chromatin or genic regions, and are therefore unlikely to be bound, functionally active TFBSs (see Methods section). For each matrix, we compiled the same number of functional and control TFBSs (listed in S1 Dataset). The median distance between functional and control motifs was 10.6KB, with 90% of functional-control sites being less than 55KB apart. Functional motifs showed significantly higher conservation scores across 35 mammals than control motifs, consistent with their differing importance in biological fitness (see Methods).

**Table 1:** Overview of the somatic mutation dataset.

Tissue	# Individuals	# Mutations	# Mutations/	# Mutations in TFBSs
			# Individuals	
Liver	315	3,746,554	11,894	1924
Pancreas	426	2,954,823	6,936	1260
LungAdenoma	24	1,446,336	60,264	680
Breast	119	647,695	5,443	388
Prostate	145	449,313	3,099	223
Lymphoma B-cell	68	425,048	6,251	183
Medulloblastoma	100	124,941	1,249	55
PilocyticAstrocytoma	340	96,515	284	51
CLL	29	52,786	1,820	8
ALL	1	7,741	7,741	8
AML	7	6,828	975	2

Shown are the numbers of samples and point mutations in the 11 tumour types studied. The last column indicates the number of somatic substitutions, which fall into TF binding motifs in constitutively open chromatin regions; substitutions affecting more than one binding site were counted once.

**Fig 1:**
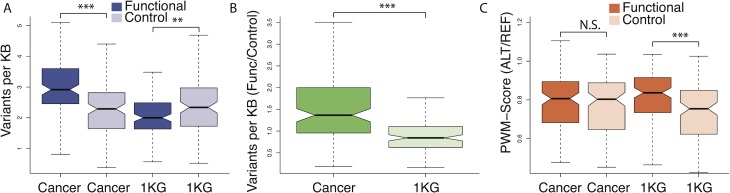
Functional binding sites are enriched for somatic mutations that are deleterious to motif binding potential. (A) In the 1KG dataset, high frequency polymorphisms (>5% minor allele frequency) are depleted at functional binding sites compared to control sites (Wilcoxon test; *p*-value = 0.003), whereas the opposite trend is observed for somatic mutations (Wilcoxon test; *p*-value = 6.522e-09). (B) There are more somatic substitutions in functional, relative to control sites in cancer compared to 1KG polymorphisms (Wilcoxon test; *p*-value = 3.652e-10). (C) The relative change of the PWM-score is lower at functional sites compared to control sites in 1KG (Wilcoxon test; *p*-value = 8.442e-05), whereas the PWM-score introduced by somatic mutations is indistinguishable between functional and control sites. Each of the 118 binding motifs contributes one data point to the plots in this Figure.

**Fig 2:**
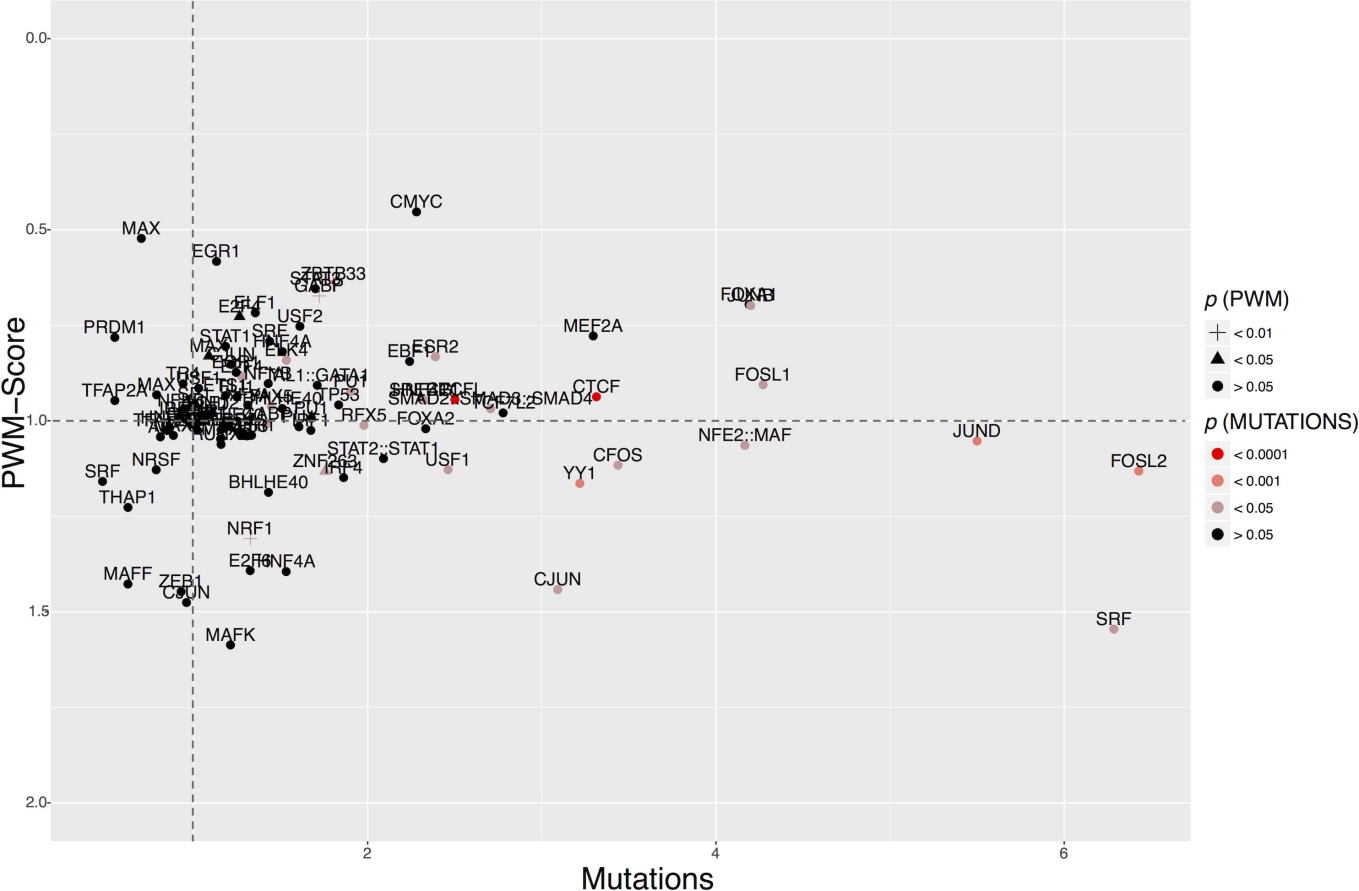
Mutation accumulation and TFBS motif disruption in cancer compared to control sites and polymorphism data. The X-axis shows the ratio of the number of substitutions in functional, relative to control sites in cancer, divided by the corresponding numbers for 1KG polymorphisms, i.e. (cancer_functional/cancer_control)/ (1KG_functional/1KG_control). Values > 1 indicate an excess of mutations in functional binding sites in cancer, correcting for the amount of variability that is tolerated at these sites at the population level. The Y-axis shows the corresponding ratio for the reduction in PWM-score. Values < 1 indicate that the matrix score is reduced to a greater extent in functional, relative to control sites in cancer compared to 1KG polymorphisms. The color and shape of the data points indicate the significance of their departure from random expectation. Note that some motifs were excluded from this plot because the *p*-value for the difference in PWM-score reduction could not be calculated (full list in S1 Dataset).

Considering each TFBS matrix separately, the total number of mutations increased linearly with the length of sequence encompassed by the TFBSs as expected (S1 Fig). This was also true for control TFBSs in cancer and for high frequency germline variants, i.e. 1000 Genomes Project (1KG) polymorphisms at both functional and control TFBSs (S1 Fig). However, in the combined dataset across cancer types, we found a marked genome-wide excess of somatic mutations at functional TFBSs. This excess was seen relative to control motifs and compared with 1KG polymorphism rates (Fig 1A and 1B and S1 Table; χ^2^-test with Yate’s correction: χ^2^ = 298.2; *p* < 10^−4^). Stratifying the data by the type of binding motif, the vast majority of TFBSs (78%, 92/118 matrices) showed an excess of substitutions at functional binding sites compared to control sites (Fig 2), with 27 TFBSs showing significant enrichment for mutations (Fisher’s exact test *p* < 0.05), and none with significant depletion. Accordingly, putatively active TFBSs are common targets for mutations in cancer and, on average, these sites mutate at higher rates than inactive control sites.

We also observed an increase of somatic mutations at functional TFBSs compared to the regions of open chromatin that they occur within: functional TFBSs mutated at significantly higher rates than constitutively open DNase regions (S2 Fig; 0.00348 *versus* 0.00336 mutations bp^-1^; χ^2^ = 4.35, *p* < 0.05). This increase is seen in spite of the fact that constitutively DNase accessible regions suffered higher mutation rates than both the mappable portion of the genome as a whole (0.00321 mutations bp^-1^; S2 Fig; χ^2^ = 25.26, *p* < 10^−6^), and the ENCODE DNase master sites, which are regions that are accessible in any of the 125 ENCODE cell lines (0.00301 mutations bp^-1^; S2 Fig; χ^2^ = 152.89, *p* < 10^−15^). Thus, TFBSs within DNase regions suffer unusually high mutation rates, even relative to the generally elevated mutation rates seen at regions of accessible chromatin, consistent with a mutational cost of factor binding.

### Global relaxation of purifying selection at functional TFBSs in cancer

To quantify the deleteriousness of somatic mutations in TFBSs, we calculated the reduction in the position weight matrix (PWM) score caused by a substitution [21]. Specifically, we calculated the PWM-score for each mutated binding site and compared this to the PWM-score for the reference sequence from the human genome build (hg19), i.e. we calculated the statistic PWM-score(ALT/REF). On average, 1KG polymorphisms reduced the PWM-score to a greater extent at control sites than at functional TFBSs (Fig 1C), as expected if purifying selection in extant human populations often acts to remove deleterious variants at functional sites. In stark contrast, the PWM-score(ALT/REF) values generated by somatic mutation in cancer are statistically indistinguishable between functional and control TFBSs (Fig 1C), suggesting a widespread loss of selective constraint at these sites in cancer. Next, we calculated the ratio of the PWM-score(ALT/REF) in functional, relative to control binding sites for all 118 motifs in both cancer and 1KG; for 68 motifs, the reduction in the PWM-score was greater in cancer than in 1KG (Fig 2), with 4 motifs attaining statistical significance. Hence, in cancer, functional binding sites do not only acquire an excess of mutations, but the changes introduced by these mutations often lead to PWM-scores that are predicted to be more deleterious than substitutions tolerated as polymorphisms. Intriguingly, two TFBS motifs (ZNF263 and NRF1) had significantly increased relative PWM-scores in cancer compared to 1KG (S1 Dataset), suggesting binding is enhanced in cancers, and raising the possibility of adaptive evolution at these particular classes of binding sites in cancer.

### A distinct mutational focus within functional CTCF binding sites

**Fig 3:**
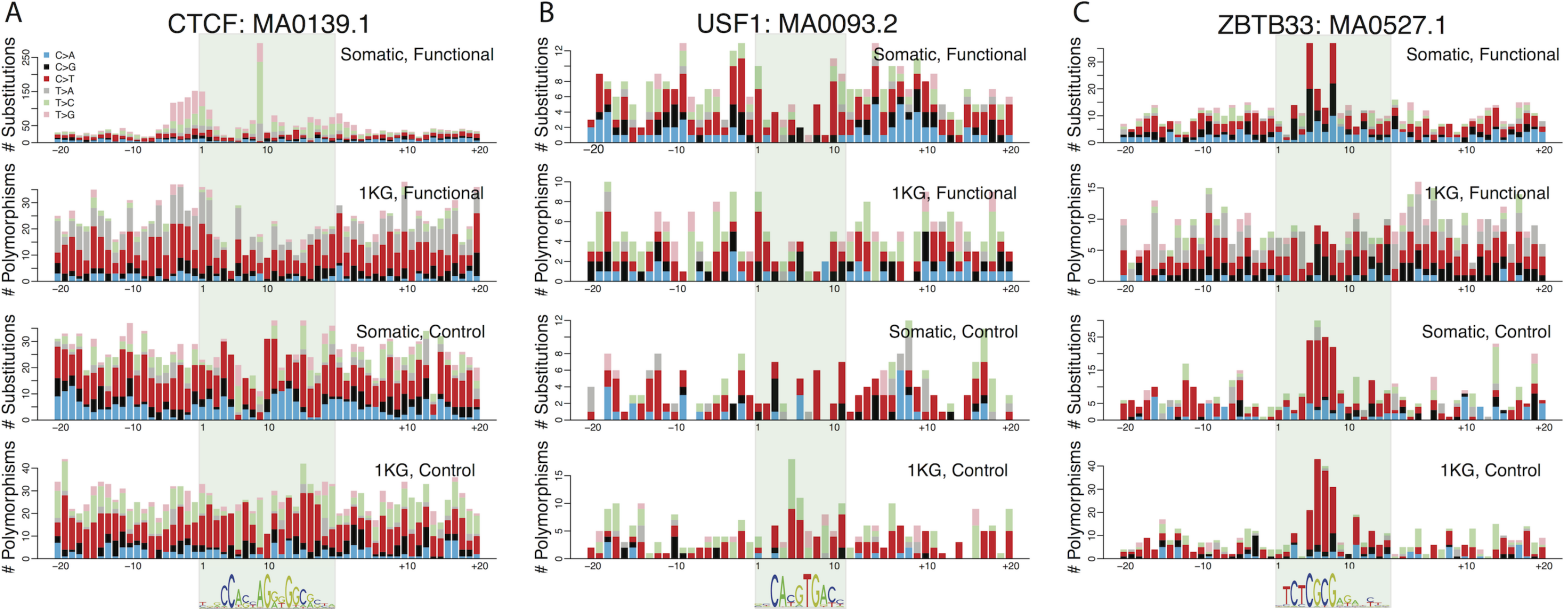
Somatic mutation and polymorphism patterns within TF binding sites. Substitution counts across all binding sites for each of three motifs, selected from the full list of 118 motifs (see S8 Fig for similar plots for all motifs tested). For comparison, substitution counts at control sites and 1KG high frequency polymorphism counts are shown in the panels below. (A) Substitution counts for CTCF: MA0139.1. (B) Substitution counts for USF1: MA0093.2. (C) Substitution counts for ZBTB33: MA0527.1.

CTCF binding sites are among the most common TFBSs in the genome (S1 Dataset), and we found the CTCF-motif to be recurrently mutated at position 9 across cancer types (Fig 3A), a pattern that was previously seen in CTCF-TFBSs identified via Chip-exo of CTCF in a colorectal cell line [10]. Note that the majority of our constitutive CTCF-TFBSs (8,795 out of 10,763) overlap with those identified by Katainen *et al*. [10]. The distribution of mutations within functional CTCF TFBSs in our dataset was significantly different from that of 1KG polymorphisms (Fisher’s exact test, *p* < 10^−5^; S2 Dataset), with the central nucleotide known to be constrained at the population level but highly mutated in cancer (Fig 3A) [10]. Most substitutions at position 9 of the CTCF-motif are T>G, T>C and T >A in cancer (Fig 3A), and mutations away from T at this information-rich central motif position are expected to lead to reduced binding of CTCF [22]. Overall, we observe an exceptionally high mutational burden at functional CTCF binding sites in cancer (3.31-fold excess) relative to control sites, and we demonstrate that this effect is seen across cancer types (Fig 2). This unusual accumulation of substitutions could conceivably be the result of selective processes or mutational bias during cancer evolution. In either case, the mechanisms that lead to a specific site of the motif being subject to high rates of substitution, remain elusive.

We stratified our samples into five mutational spectra (S3 Fig and S2 Table), based upon the genome-wide occurrence of substitutions in their trinucleotide context, consistent with previous studies (see Methods). Since we subdivide the data into only five signatures, a one-to-one comparison with the 21 mutational signatures of Alexandrov *et al*. [5] is not possible. However, we observe a similar grouping of lung adenocarcinoma samples (in mutational group 1, characterized by C>A mutations; Alexandrov *et al*.’s signatures 4 and 5), and observe an overrepresentation of C>T changes across most cancer samples. Interestingly, the excess of T>G/C/A mutations at position 9 of the CTCF-motif was only seen in mutational spectra 3 and 5 (S4 Fig), and it was strongest in spectrum 3 which also shows the strongest T>C signature. In contrast, tumours in spectrum 1 do not show the elevated substitution rate at position 9. Similarly, the total number of mutations in functional motifs, relative to control motifs, is not elevated in spectrum group 1, as it is for samples in spectra 2, 3 and 5 (S3 Table). Thus, the increase in mutation at CTCF binding sites is driven by mutations at position 9, which is heavily mutated in particular subsets of samples with a common mutational signature and indicative of the dominant underlying mutational process.

It has recently been shown that liver cancer is particular prone to asymmetries of A>G/T>C mutations in relation to the transcribed and untranscribed DNA strands [23], and we observe a similar genome-wide trend for the liver cancer samples (S4 Table) here. A:T nucleotides were more prone to mutate to G:C when the ‘A’ nucleotide occurred on the non-transcribed strand and the ‘T’ was on the transcribed strand. Interestingly, the same trend was also seen for the subset of functional CTCF sites that fall into transcribed genomic regions, and these sites mutated at much higher rates than the genome wide average (S4 Table); this further supports the notion that mutations at CTCF-TFBSs follow genome-wide trends in mutational bias.

### CTCF sites implicated in higher order chromatin structure are frequently mutated across cancers

**Fig 4:**
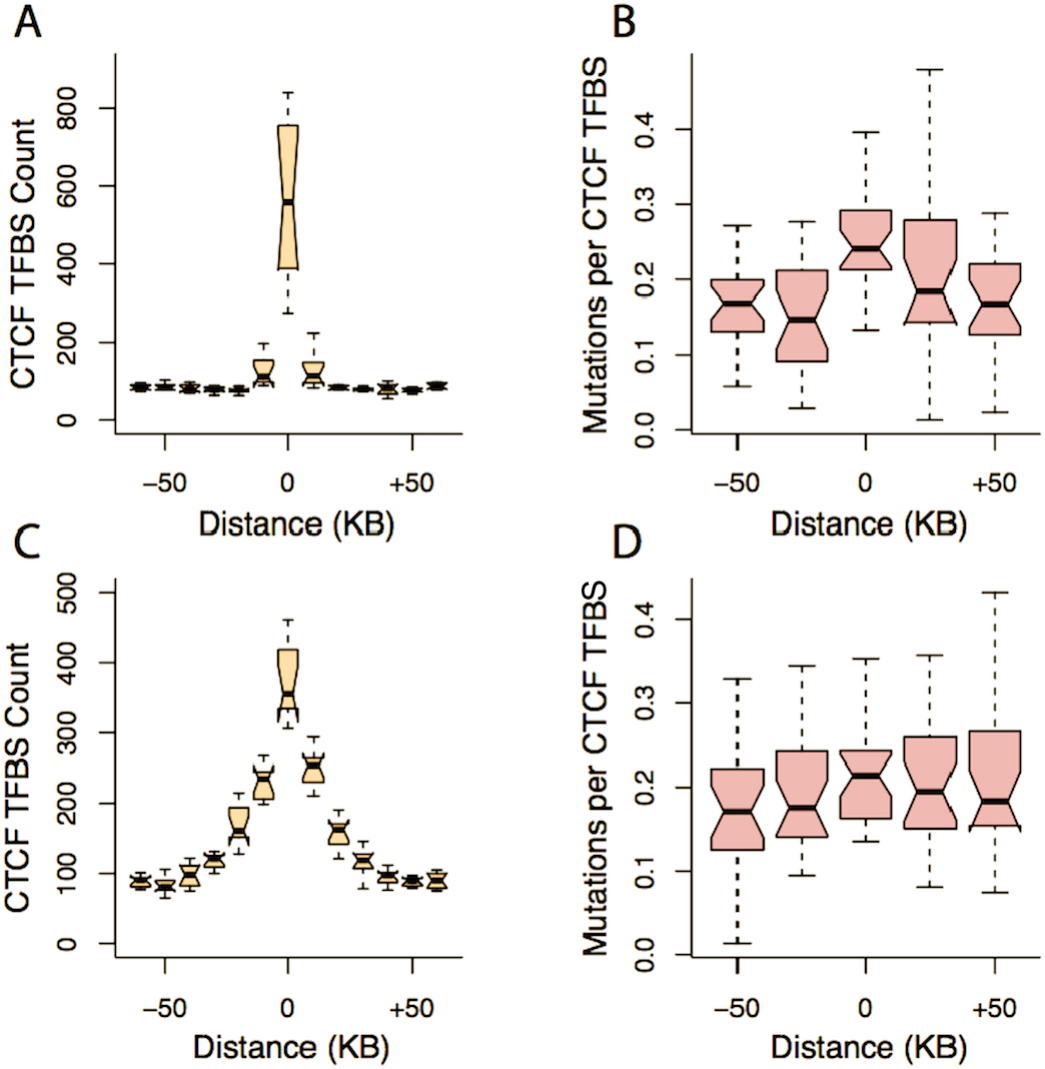
CTCF-binding sites are often mutated when acting as modulators of chromatin structure. CTCF-motifs are highly enriched inside loop anchor points and across domain boundaries (A, C). The number of substitutions in CTCF-motifs is increased for motifs that are located in loop anchor points (B); for domain boundaries, no significant increase in substitution rate was observed (D).

CTCF has long been known to have important architectural roles in chromatin structure [24,25]. Rao *et al*. [9] found that CTCF binding sites delineate a hierarchy of chromatin loops (indicating peaks of Hi-C contact frequencies), and regulatory domains (median size 185KB) that compartmentalize the genome into self-interacting units. The majority of points in the genome marking the beginnings and ends of chromatin loops (loop anchor points) are bound by CTCF, and are thought to link regulatory sites to target promoters. The majority (55–75%) of loop anchor points are conserved across human cell types, and across mammals; many of these loops also demarcate the boundaries of self-interacting regulatory domains [9]. Using a sliding window approach, we found the number of functional CTCF motif instances to increase sharply at chromatin loop anchor points and domain boundaries (Fig 4A and 4C). Functional CTCF motifs were strikingly prone to mutation if they were located within chromatin loop anchor points (Fig 4B and S5 Table), with a similar (though non-significant) trend evident at domain boundaries (Fig 4D), whereas there was no significant enrichment of mutated control motifs (S5 Table). Further, position 9 of the CTCF-motif was more highly mutated when the binding site was located inside a loop anchor point. Inside loop anchor points, 204 out of 792 observed substitutions (26%) were at position 9 of the motif, compared with 15% (83/539) in functional motifs outside loop anchors, despite the motifs having very similar sequence composition inside and outside loop anchor points (S5 Fig). The mutation rate was approximately three-fold higher within CTCF sites within loop anchor points, compared to the rate observed within anchor points in general (S2 Fig; χ^2^ = 1242.00, *p* < 10^−15^), supporting the idea that the CTCF-motif is a hotspot of mutation within this specific chromatin context.

Given the limited numbers of mutations recorded in some tumour samples, we could not rigorously determine if CTCF motifs were highly mutated inside chromatin anchor loops across all tumour types. However, an excess of mutations inside loop anchor motifs was observed in all cancer types with a sufficiently high number of CTCF mutations, i.e. whenever the power to detect this difference in mutation rates at alpha = 0.05 was 80% or greater (S6 Fig). Thus, CTCF sites involved in higher order chromatin structures appear to suffer the highest mutational burden, and chromatin organization may be affected by this increased mutational input across several cancer types. S6 Table lists the number of CTCF-mutations for each cancer type and shows that the highest mutation rates at functional CTCF sites per individual are suffered by liver and lung tumours, with substantial mutational loads also seen for breast, pancreas and lymphoma samples. In contrast, the relatively numerous (Table 1) medulloblastoma and astrocytoma samples show orders of magnitude lower rates per individual, suggesting that different cancer types experience very different degrees of CTCF binding site disruption (S6 Table).

Using the GREAT tool [26] with default parameters, we tested for enrichments of functional annotations at genomic regions associated with mutated functional CTCF-sites. We found modest over-representation of certain functional categories, including biological processes associated with the regulation of cellular secretion, and several cancer-associated MSigDB entries, such as down-regulated genes predicting poor survival of patients with thyroid carcinoma (S7 Table).

We further explored the chromatin context of mutated TFBS instances, examining whether particular functional chromatin states were associated with the propensity of a particular TFBS to undergo mutation (see Methods). Among the 118 TFBSs tested, the mutational load of only five TFBSs (E2F1_MA0024.2; CTCF_MA0139.1, CTCFL_MA0531.1; E2F4_MA0541.1 and YY1_MA0095.2) showed an uneven distribution among chromatin states (Chi-Squared Test, *p* < 10^−3^). In each case, there was an excess of mutations in insulator regions (S3 Dataset). In particular, 16–17% of the CTCF functional binding sites allocated to the “insulator” chromatin state carried a mutation in at least one sample, whereas CTCF TFBSs in “promoter”, “enhancer” and “transcription” regions were mutated less often (5–10% of functional sites). This suggests that CTCF binding sites are particularly prone to mutation when they are involved in specific chromatin contexts. This appears to reflect variation in the rates of somatic mutation in DNAse hypersensitive sites in general, which was 0.0039 per base pair in accessible regions classified as “insulator”, but only 0.0032 in regions classified as “promoter”, “enhancer” and “transcription” (χ^2^ = 128.61, *p* < 10^−15^).

We used logistic regression to assess which genomic parameters were prominently associated with a high rate of substitution across the 118 TFBSs. Factors, which significantly affected the propensity of a binding site to undergo mutation in cancer, included replication timing, the identity of the TFBS matrix, the functionality (i.e. DNase status) of sites and whether sites were present at loop anchor positions (S4 Dataset). Logistic regression analysis confirmed that functional binding sites consistently mutate more often than control sites, that the positioning within loop anchor points increases a binding site’s chance of mutation, and that different binding motifs mutate at distinct rates. In addition, late replication was significantly associated with higher rates of mutation in the regression model, consistent with a general role for replication timing in the nucleotide substitution rate [27,28]. In fact, when we correct for replication timing, the difference in mutation rates between CTCF motifs inside and outside chromatin loop anchor points diminishes (S4 Dataset and S7 Fig). These CTCF binding sites might otherwise have been regarded as candidates for the apparent action of selection in cancer, given their specialized roles as well as the elevated frequencies and specific patterns of mutation observed. It is therefore striking that even for these sites mutational bias emerges as a convincing explanation for the patterns observed.

### Towards a taxonomy of regulatory dysfunction in cancers

Motivated by the patterns of site-specific mutation accumulation in CTCF, we investigated the pattern of substitutions on a per-site basis for all 118 TFBSs, but found few examples of selection acting to preserve motif integrity. For example, ZBTB33, a regulator of the Wnt signaling pathway, binds to methylated 5'-CGCG-3', and showed evidence for preservation of its target TFBSs in 1KG data (Fig 3C). By contrast, in cancer, ZBTB33 binding sites were highly mutated at positions 5 and 8, reflecting the high mutational input evident at ZBTB33 control motifs (Fig 3C). The significantly elevated numbers of mutations at these motifs were accompanied by a reduced PWM-score for the ZBTB33 motif in cancer (S1 Dataset). Examination of most TFBSs suggests a similar situation, but the USF1 binding motif (MA0093.2) was a rare exception. Functional USF1 TFBSs showed a depletion of substitutions compared to flanking regions—in the 1KG polymorphism as well as the cancer dataset—but this depletion was absent at control sites (Fig 3B). In addition, mutations at functional USF1 binding sites reduced the PWM-score to a much lesser degree than control sites in cancer (Fig 2 and S1 Dataset). Due to the relatively modest number of mutations present at USF1 sites in the current data, the comparison with 1KG PWM-scores was not statistically significant, but these observations are consistent with motif preservation at USF1 binding sites in cancer. The complete dataset for each of the 118 matrixes, their controls sites, flanking regions and 1KG comparison, are provided in S7 Fig.

We found no evidence that significantly mutated binding motifs are more likely to be bound by transcription factors which have been reported to suffer recurrent protein coding sequence mutations, i.e. genes that are found in the Cancer5000 gene set of Lawrence *et al*. [2] (S8 Table; Fisher’s test N.S.). This suggests that mutations at TFBSs and those within coding regions have largely independent impacts on regulatory dysfunction in cancer. Further, we found little recurrence of mutations at individual functional binding sites: the most highly mutated positions inside motif instances were mutated in only five out of the 1,574 tumor samples each, at chr6:73122103, chr2:49173806 and chr2:49173798, affecting the binding motifs of CTCF/YY1 and CTCF/CTCFL, respectively. The chr6:73122103 site was also previously found to be mutated in 3.5% of colorectal cancer samples [10]. In contrast, the two most highly mutated sites across cancer genomes in protein coding sequence are a known mutational hotspot in codon 12 of the KRAS gene; these sites carried substitutions in 257 and 67 tumors, respectively. Thus, in contrast to coding sequences, where specific loci suffer detectably higher mutation rates, the mutational burden at regulatory sites requires a genome-wide perspective, encompassing many individual sites that belong to a given class of TFBS.

**Fig 5:**
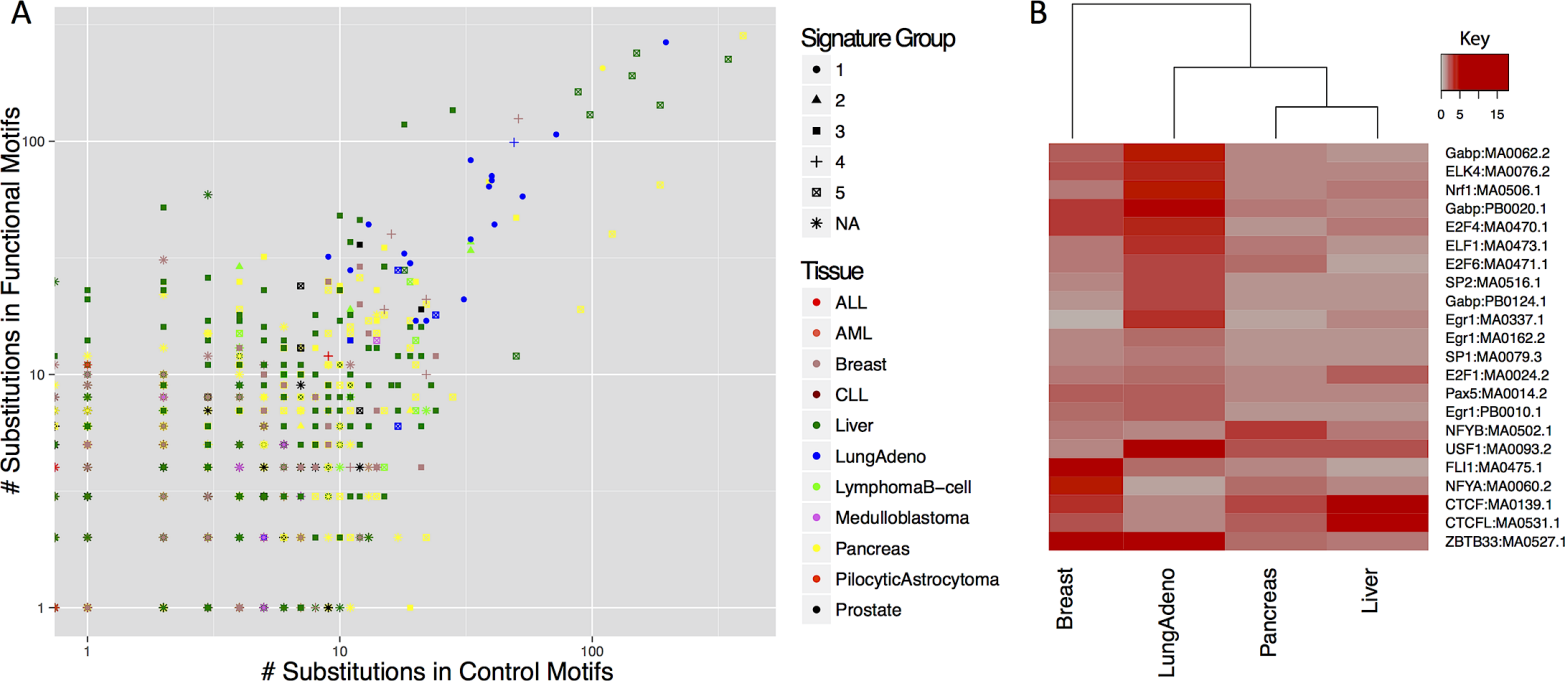
A) The number of mutations inside functional and control TFBSs, plotted for each tumour sample in this study. B) Cancer mutations in TF binding sites, stratified by tumour type. Heatmap of mutation counts in functional motifs, relative to control sites and 1KG polymorphisms. Only motifs that occur in at least 1,000 binding sites in the genome are shown, for the four tumour types with the highest total number of mutations. Increasing shades of red: ratio > 1, indicating an excess of functional mutations in cancer; grey: ratio <1.

In spite of the broad loss of constraint seen across TFBSs in cancer, it was possible to discern differences among cancer types, even with the limitations and caveats of the current data. We found that the particular binding motifs mutated in functional, relative to control sites and 1KG polymorphisms differed markedly over different cancer types (Fig 5; complete dataset in S5 Dataset). Stratifying the data by cancer type reduces the mutation counts in each category, but suggests that lung adenoma tumours (which also possess a distinctive mutational profile; S2 Table) may accumulate more mutations at functional TFBSs compared to other cancer types, with the notable exception of CTCF binding sites. Within cancer types, we observe large variation in the numbers of mutations on a per-patient basis (Fig 5). The high rate of TFBS mutations in liver cancer is in part driven by a small number of outlier patients with exceptional biases to mutation in functional rather than control motifs (Fig 5). With larger cancer sequencing datasets it is likely that such variation among cancer types will become clearer, promising a new perspective on cancer genomics.

## Discussion

We have shown that functional regulatory elements suffer elevated rates of somatic mutations in cancer that based upon the accumulation of substitutions relative to matched control sites appear deleterious to regulatory protein binding. These striking patterns of mutation differ across TFBSs and cancers, and yet a high attrition of CTCF sites is a notably general feature. The unusual patterns of mutation seen at CTCF sites suggest widespread alterations to regulatory chromatin architectures across the genome, underpinned by strong mutational biases rather than selective processes. This raises the possibility that regulatory ‘driver’ mutations in cancers may arise as a byproduct of such biases superimposed upon a genome-wide relaxation of selective constraint at regulatory sites.

The strongest impact of mutation on functional CTCF sites in the current data was observed in liver cancer samples, which showed the most dramatic increase in numbers of mutations observed (S2 Table). We have shown that, by examining aggregated sites across the genome, it is possible to detect these patterns rigorously, while controlling for the influences of sequence composition and regional variation in mutation rates. However, it is important to note that these patterns will remain undiscovered by conventional approaches, most of which are based upon identifying individual genomic regions subject to recurrent mutations, and make it difficult to correct for compositional bias. This is exemplified by a recent publication describing the liver samples studied here, which assessed mutation rates within 500bp genomic windows, did not correct for compositional bias, and was therefore unable to detect the genome-wide increase in mutation rates at CTCF sites [29].

Regions of open chromatin have previously been shown to mutate at a decreased rate [13,28,30,31], presumably as such regions are more accessible to the DNA repair machinery. However, these analyses were based on sections of large, often multi-megabase regions, rather than the short binding motifs, about 10-20bp in size, examined here. Michaelson *et al*. [32] found DNAse I sites often to be *de novo* mutated in the germline, especially when the applied window sizes were small, i.e. 10 or 100bp. Recent studies [10,33–35] have suggested possible mechanisms for increased mutation rates at TFBSs, including the perturbation of lagging-strand replication at strong binding sites, and differential accessibility of binding sites to the nucleotide excision repair machinery. An emerging theme here is that there may be a general mutational burden to regulatory function, where the action of sequence specific binding to DNA interferes with normal replication, damage, surveillance and repair processes. The breadth of effects we observe genome wide, across many transcription factors and tissues of origin, suggests that these are pervasive influences on the mutagenicity of the genome. As the net effect is one of increased mutation rate specifically at functional regulatory sites, it will be important in future studies to explore the mechanistic nature of these interactions and the relative importance of replication, repair and exogenous mutagenesis to the locally elevated mutation rates.

We have shown that the mutation mediated decay of TFBSs can be observed across cancer types and binding motifs, and there appears to be no widespread purifying selection to counteract this. Among 118 motifs tested, not a single motif was significantly depleted for mutations at functional sites, relative to comparisons with control sites or population variation (1KG), suggesting that most binding sites for most known transcription factors are dispensable for tumor survival. Further, considering the per-site mutation rates within motifs, we often observe the same patterns of substitutions at control and functional sites, e.g. CpG mutations, suggesting that the accumulation of substitutions at TF binding sites is mostly driven by mutational rather than selection processes. Finally, the recurrence of mutations in functional TFBSs was two orders of magnitude lower than at sites of recurrent mutation in protein coding regions, consistent with the notion that no individual TF binding site in our dataset is likely to be a major driver of tumorigenesis. However, this does not mean that the aggregated, genome-wide impact of mutations across many TF binding sites is negligible. For example, the widespread disruption of CTCF-binding sites may have drastic consequences for the chromatin organisation and hence regulation of tumour gene expression [36], and possibly for the stable transmission of DNA in subsequent cell divisions [37]. Cancers with a strong A:T>G:C mutational signature were particularly affected by CTCF binding site mutations, and such cancers may show higher degrees of regulatory instability. Consistent with our results a recent study showed that the disruption of chromatin boundary sites may activate proto-oncogenes in T-cell acute lymphoblastic leukemia, and observed a similar excess of mutations at CTCF sites [38].

Many previous studies (e.g. [29]) have used comparisons between binding sites and their flanking regions to assess the relative somatic mutation rates at such sites. Given the inevitable differences in sequence composition between binding sites and flanks, and the large literature supporting the role of compositional bias in mutation rates [2,11], this is a challenging strategy. In addition, since TFBSs are highly clustered in the genome, the neighbouring regions of any given motif may also act as binding sites for other factors, potentially affecting flanking rates of mutation. Third, it has also recently been shown that immediately flanking regions *per se* may undergo increased rates of mutation [33], which is consistent with the mutational input observed at CTCF TFBSs (Fig 3). In this study, we use a metric comparing the rates of mutation in functional *versus* control motifs of matched length and composition, circumventing biases introduced by differences in nucleotide sequence composition of the binding site or its flanks. Nevertheless, for comparison with prior studies in S1 Dataset, we compare the number of mutations in functional and control sites seen for each binding motif, relative to their 100bp flanking regions.

One should note that our global analysis, in common with others to date, was limited by the heterogeneity of substitution rates across tumour types and by the numbers of mutations found within TF binding sites, which bounded the statistical power of our analyses; further, all *p*-values shown are uncorrected for multiple testing of 118 binding motifs. Thus, it was not always possible to meaningfully stratify results by mutational signature group or tissue of origin. Considering each tumour type separately, it appears that some cancer samples have a reduced proportion of mutations in functional motifs compared to control sites (Fig 5). However, the number of samples and/or the overall rate of mutation within these cancers are relatively low, which increases sampling bias. In our genome-wide pan-cancer analyses, the weaker patterns seen in these tumours is overridden by cancers such as lung adenoma and liver cancer, which show an excess of mutations at functional sites (Table 1; Fig 5). Thus, with additional cancer WGS data to explore, many new insights into the regulatory genomics of cancers should be possible.

## Materials and Methods

### TFBS prediction

To detect functional regulatory binding sites in the genome, we used a combination of computational prediction and experimental data: Position weight matrices for 118 transcription factor binding motifs (85 from ensemble Biomart at http://grch37.ensembl.org/biomart/martview/9620562a1888b791f43eb69ee9adcaf0 and 33 additional motifs from Jaspar [39] at http://jaspar.genereg.net/) were used as input to FIMO (of the MEME suite [40]), to find predicted motif matches in the genome. The maximum *p*-value for a motif match was set as the default (*p* < 4.4e-05); if more than 300,000 motif instances were found, the motifs with the largest *p*-values were iteratively dropped. We intersected these motif matches with experimentally defined open chromatin regions: UCSC DNase master sites were downloaded from the UCSC genome browser (http://genome.ucsc.edu/cgi-bin/hgTrackUi?db=hg19&g=wgEncodeAwgDnaseMasterSites), and DNAse footprints came from Thurman *et al*. [8], with footprints calculated as in Neph *et al*. [19]. In order to avoid the erroneous classification of binding sites as active in tumour tissue, we only considered putative binding sites in constitutively open chromatin, i.e. in UCSC chromatin regions that were DNAseI accessible in at least 113 out of 125 ENCODE cell types, or within DNAse footprints that were found in at least 39 out of 41 tissues. We conservatively limited our analysis to these putatively functional binding sites in constitutively DNAseI hypersensitive sites, and accordingly, expect a relative underrepresentation of tissue-specific binding sites in our dataset. The aim was to enrich our ‘functional’ sites for active TF binding relative to control sites. Note that, due to partial positional overlap of motifs, 44% (2,123 out of 4,782) of the somatic substitutions found within functional sites affected more than one TFBS, supporting the functional significance of these sites. As control motifs, we chose FIMO motif matches that were located outside open chromatin regions/DNAseI sites in any tissue of the ENCODE and Thurman datasets; in addition, control motifs had to be in the mappable regions of the genome (i.e. outside DUKE and Dac excluded regions [41]) and more than 2kb upstream of known genes. To minimize the difference in the mutation rate among functional and control TFBSs, we position matched each functional motif instance with a nearest control motif, choosing, for each functional TFBS, the closest motif from the pool of possible control sites. Functional and controls TFBSs both had high and comparable uniqueness scores (S9 Table), suggesting that mutations can be detected in both regions. We note that the GERP conservation score [42] across whole genome alignments of 35 mammals (http://genome.ucsc.edu/) is, on average, higher for functional TFBSs than for control motifs (S9 Fig); this is expected if functional motifs are under purifying selection, and has no impact on our analysis. Functional motifs match the input position weight matrices slightly better than control motifs, with median PWM-scores of 8.73 and 8.33, respectively (S9 Fig). However, since we measure the reduction in score *relative* to the reference allele, this should have negligible consequences for our analysis, and, consistent with this, the reduction in score is lower for functional TFBSs in the 1KG data, even though functional motifs start off with slightly higher scores (see Results section).

### Cancer mutation data

We downloaded whole genome mutation annotation format (maf) files for 11 tumour types from public data resources: 507 samples came from Alexandrov *et al*. [5], and a further 1,067 non-embargoed samples (free of all publication moratoria) came from Release_17 of the ICGC [17], including the projects LINC-JP, BRCA-UK, LIRI-JP, CLLE-ES, MALY-DE, PBCA-DE, EOPC-DE, PRAD-CA, PRAD-UK, PACA-AU, LICA-FR and PACA-CA. The maf files had previously been filtered for germline variants, i.e. they only included somatic mutations. 1KG polymorphism data (vcf files) were from EBI (ftp://ftp.1000genomes.ebi.ac.uk/vol1/ftp/release/20130502/). Somatic point mutations and 1KG common SNPs with a frequency of >5% were intersected with our set of functional binding sites and control motif sites.

PWM-scores [21] were calculated for each motif site that carried somatic substitutions or polymorphisms, and this score was compared to the reference allele, i.e. the motif instance in the human reference assembly (hg19). The relative reduction or increase in PWM-score for each binding site was calculated as PWM-score(ALT)/ PWM-score(REF), thereby controlling for variation in information content between motifs.

To assess the impact of mutations in cancer with regards to the number of mutations per motif site and the predicted change in PWM-score, we divided the data into four separate categories: 1) somatic mutations at functional sites; 2) 1KG polymorphisms at functional sites; 3) somatic mutations at control sites; 4) 1KG polymorphisms at control sites. Variants with a frequency > 5% in the 1KG dataset may be neutral, advantageous or mildly deleterious, but are unlikely overall to be under strong purifying selection. Accordingly, the level of 1KG polymorphism at functional sites, relative to control sites for the same motif, gives an indication of the level of constraint for a given class of binding sites [43] and can be compared to the patterns of mutation seen in cancers.

The significance of enrichment or depletion of mutations inside functional TFBSs in cancer was assessed using Fisher’s exact test for mutation counts in the four classes of sites: functional and control sites in cancer and 1KG, respectively. To assign a *p*-value to the reduction in the PWM-score, we used the methods of Price and Bonett [44] and calculated, for each binding motif, the confidence intervals for the ratios of median relative PWM-scores in cancer (functional/control) and 1KG (functional/control) separately, and assessed the extent to which they overlapped.

Aggregate mutation/polymorphism counts were produced for each binding motif and sample; the shape of the distribution between cancer and 1KG samples (visualized as barplots in S8 Fig) was compared using Fisher’s exact test.

### Mutational spectrum analysis

Mutational spectra were calculated by counting the number of each of the 96 possible substitution types for each cancer sample, and dividing this vector by the expected number of substitutions, which was based on the trinucleotide count in the human reference sequence and assuming that a substitution from any nucleotide to any other is equally likely [5]. The Manhattan distance between each sample-specific mutational spectrum (scaled to a total sum of one) was calculated, with a dendrogram based on hierarchical clustering to relate samples. To avoid errors due to sampling of low mutation counts, the dendrogram shown in S3 Fig only included samples with at least 7000 mutations. Samples were allocated to five different spectra based on their clustering in the dendrogram.

### Transcriptional asymmetries

We divided CTCF-binding regions of the genome, which also overlap transcribed regions, into two groups, based on whether DNA is transcribed from the reference strand or its complement according to the ENSEMBL annotation of hg19. A total of 44,072 and 40,507 basepairs overlap functional CTCF motifs and are transcribed from the reference and complement strands respectively, excluding sites that are transcribed bi-directionally. Next, we counted the number of A>G and T>C changes at CTCF sites in liver cancers; we assessed whether the reference “A” nucleotide was on the transcribed or the non-transcribed strand of DNA (with its complement, “T”, being on the other strand), and calculated the strand bias of these mutation classes as in Haradhvala *et al*. [23]. We repeated the same procedure for all liver somatic mutations that fell into unidirectionally transcribed regions of the genome (612MB and 587MB of DNA for reference and complement strands respectively).

### Chromatin data and integrative analysis

Chromatin loop anchor positions and chromatin domain boundaries based on the Hi-C data of GM12878 (the cell line with the highest resolution of 950bp from Rao *et al*. [9]) were obtained from NCBI GEO (Accession GSE63525). Across domain boundaries and loop anchor points reported by Rao *et al*. [9], we counted the number of somatic mutations and the number of CTCF motif instances. We do not have Hi-C data for the tumour samples in this study; however, to assess if an increase in mutations at CTCF-TFBSs inside loop anchor points is seen across different cell lines, we repeated the analysis with loop anchor points called in IMR90, HMEC, NHEK, K562, HUVEC, HeLa, and KBM7 cell lines [9].

ChromHMM tracks [45] were downloaded from the UCSC Genome Bioinformatics site (http://genome.ucsc.edu/) for GM12878, H1-hESC and K562 cell lines. These datasets were intersected with the genomic location of all functional motifs, classifying each motif into falling into one of six chromatin “colors”, i.e. “promoter” (red), “enhancer” (yellow), “insulator” (blue), “transcription” (green), “repressed” (grey) and “low signal” (white). For each Matrix, we counted the number of mutated and intact functional binding sites, using a Chi-Squared test to assess if different chromatin states showed different propensities for mutation.

A logistic regression model was constructed, modeling the binary outcome variable “mutated/not mutated” in the combined cancer dataset; this variable describes if a given binding site at a particular genomic location is mutated in any of the cancer samples. As predictor variables, we used the replication timing data of Chen *et al*. [46], “Matrix” as a factor with 118 different levels representing the different TFBS motifs included, a binary “Functionality” (i.e. functional vs. control) variable and the binary classifier of whether the binding motif was inside or outside a chromatin loop anchor point [9]. The Wald test was used to test for the significance of individual predictor variables within the model. The fraction of predicted mutated motif positions was calculated for each functional matrix inside or outside loop anchors respectively, keeping replication time constant.

## Supporting Information

S1 Fig The mutation count increases linearly with the number of binding sites in the genome. The number of mutations and polymorphism counts, respectively, are plotted against the total number of base pairs covered by a given TFBS.
(PDF)

S2 Fig Barplots of the total number of substitutions per site across cancer samples. Shown are the rates for the whole genome (outside Duke and Dac excluded regions); regions covered by functional TFBSs; constitutive DNase sites; ENCODE DNase sites; flanks of TFBSs (100bp either side); control TFBSs; flanks of control TFBSs (100bp either side); chromatin loop anchor points; CTCF motifs inside loop anchor points.
(PDF)

S3 Fig Mutational spectrum analysis. a) Barplots of the genome-wide mutational spectra for 5 cancer samples, representing the 5 different mutational signature groups that were derived from a hierarchical cluster analysis in R. The total number of somatic mutations is shown for each sample. b) Dendrogram of all cancer samples that carry at least 7000 somatic mutations; samples were divided into the 5 mutational groups as indicated on the tree.
(EPS)

S4 Fig CTCF mutations across mutational spectra. Barplots of the mutation count for each functional CTCF motif site, divided by the number of individuals in the mutational spectrum group.
(PDF)

S5 Fig Sequence logos of CTCF-TFBSs inside and outside chromatin loop anchor points. Logos were created using http://weblogo.berkeley.edu/logo.cgi [47].
(PDF)

S6 Fig Detection of CTCF mutations within chromatin loop anchors and statistical power. The *p*-value of Fisher’s exact test, which compares the number of mutations in CTCF-motifs inside chromatin anchor points to the number of mutations in CTCF-motifs outside loop anchor points, is plotted against the power to detect a statistical significance at alpha = 0.05.
(PDF)

S7 Fig Replication timing of CTCF-motifs located inside and outside chromatin loop anchor points. Larger values on the y-axis indicate later replication.
(PDF)

S8 Fig Barplots of the number of mutations per matrix site (functional and control sites in cancer and 1KG) for all 118 binding motifs. (PDF)

S9 Fig **Boxplots of the GERP conservation scores (A) and PWM-scores (B) of functional and control motifs, respectively.** In both plots, asterisks indicate *p*-values of the Wilcoxon test of *p* < 10^−15^.
(PDF)

S1 Table Mutation count across tumor samples in functional and control motifs as well as in 1KG high frequency polymorphisms (> 5%). (DOCX)

S2 Table The number of samples classified as mutational spectra 1–5 as well as the number of mutations within functional binding sites. (DOCX)

S3 Table The number of mutations in functional and control sites in CTCF for each mutational group. (DOCX)

S4 Table Strand asymmetry of A:T>G:C mutations in 315 liver samples. ntx = non-transcribed strand; tx = transcribed strand. Column 2 shows the counts of the number of A:T basepairs subject to mutation at CTCF-sites (top) and genome-wide (bottom) within unidirectionally transcribed regions; column 3 lists the number of observed A:T>G:C mutations. Strand asymmetry, calculated as in Haradhvala *et al*. [23], is shown in the last column.
(DOCX)

S5 Table Counts of somatic mutations at CTCF-TFBSs inside and outside chromatin loop anchor points; based on the Hi-C datasets for eight different cell lines [9]. (DOCX)

S6 Table Counts of the number of mutations in functional CTCF-TFBSs. (DOCX)

S7 Table GREAT (Genomic Regions Enrichment of Annotations Tool) analysis. CTCF-motifs, which were found to be mutated in our dataset, were compared to a background set of all functional CTCF-motifs.
(DOCX)

S8 Table Comparison between genes whose binding motifs are significantly mutated in our dataset (*p* < 0.05) and genes that are highly mutated in the pancancer analysis of Lawrence et al. [2]. Shown are the number of genes/their motifs that are in common between each category. Binding motif of hybrid factors, such as TAL1::GATA1 were excluded from this analysis.
(DOCX)

S9 Table UCSC mapability and uniqueness scores for functional (top) and control TFBSs (bottom). (DOCX)

S1 Dataset The number of functional binding sites per transcription factor (with control sites being equal in number); the number of mutations and the relative change in PWM-Score per factor and class of element. The *p*-values shown are not corrected for multiple comparisons.
(XLSX)

S2 Dataset Significance of the Fisher's exact test, comparing the shape of distributions of functional binding sites in cancer versus 1KG high frequency polymorphisms. 1mio simulations were performed to calculate the *p*-value.
(XLSX)

S3 Dataset TFBS matrixes that were significantly enriched for mutations in blue/"insulator" chromatin in all three cell lines tested (Chi-Square test; *p*-value < 1E-3 in each comparison). (XLSX)

S4 Dataset Logistic regression analysis of the outcome variable "mutated/not mutated". (XLSX)

S5 Dataset The number of mutations in functional and control motifs per tissue. The last columns ("ratio" columns") show the ratios of the number of functional over control substitutions, divided by the corresponding 1KG polymorphism counts.
(XLSX)
