# Supplementary figures and images for: Mutational Biases Drive Elevated Rates of Substitution at Regulatory Sites across Cancer Types
Source: PLoS Genet. 2016 Aug 4;12(8):e1006207. doi: 10.1371/journal.pgen.1006207 (PMC4973979; doi:10.1371/journal.pgen.1006207)

**Functional, Somatic**

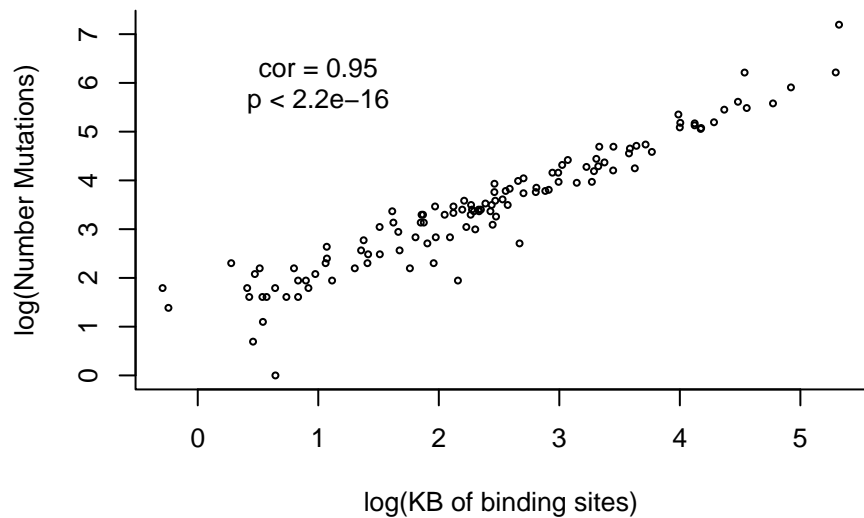

**Control, Somatic**

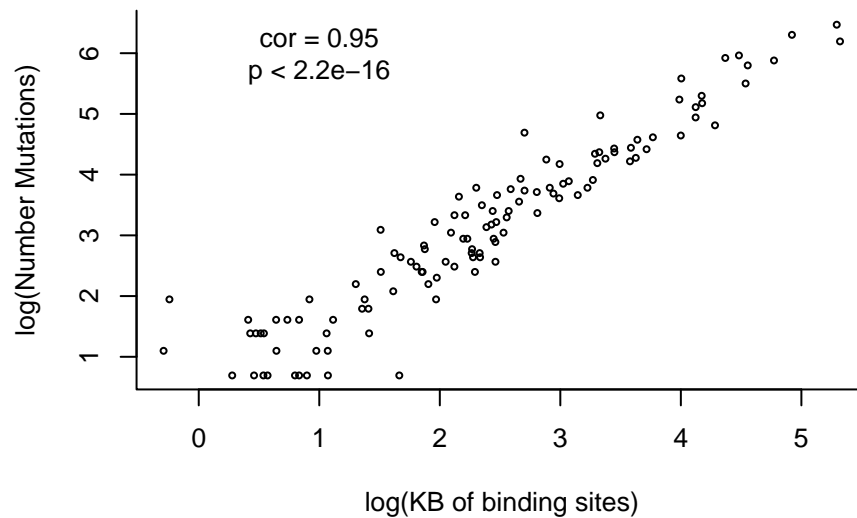

**Functional, 1KG**

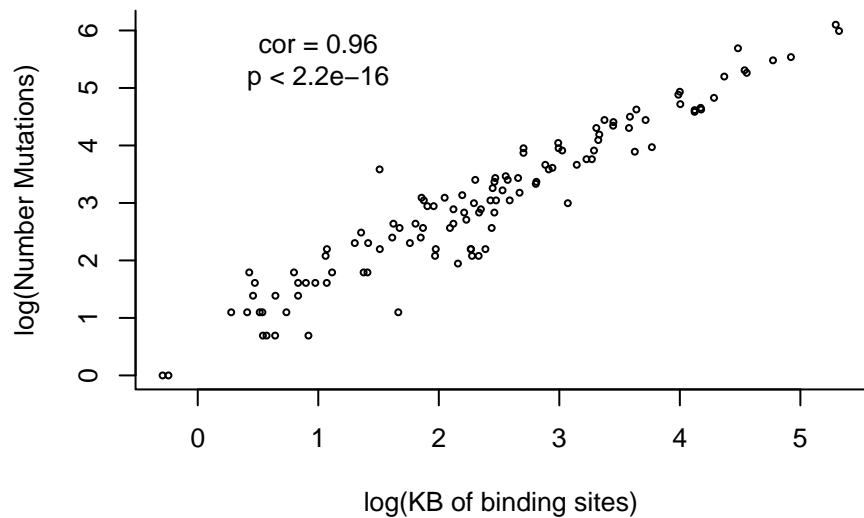

**Control, 1KG**

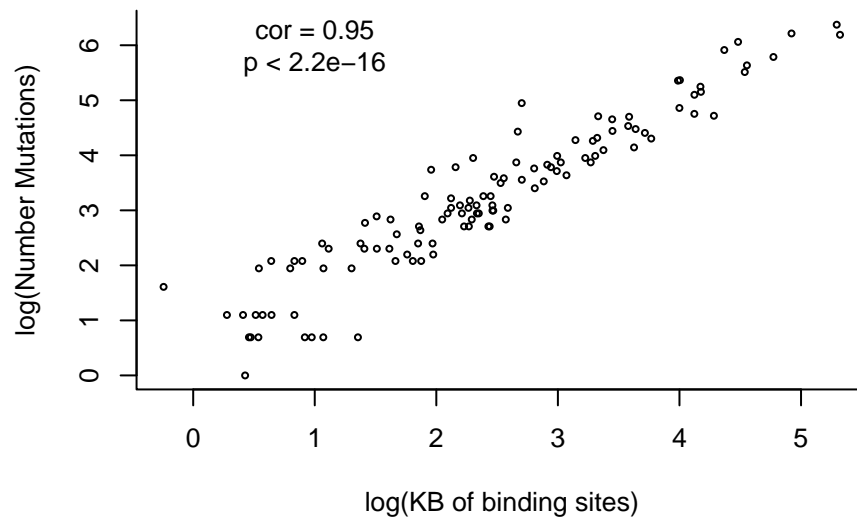

Supplement: S1 Fig — The number of mutations and polymorphism counts, respectively, are plotted against the total number of base pairs covered by a given TFBS. (PDF) [file pgen.1006207.s001.pdf]

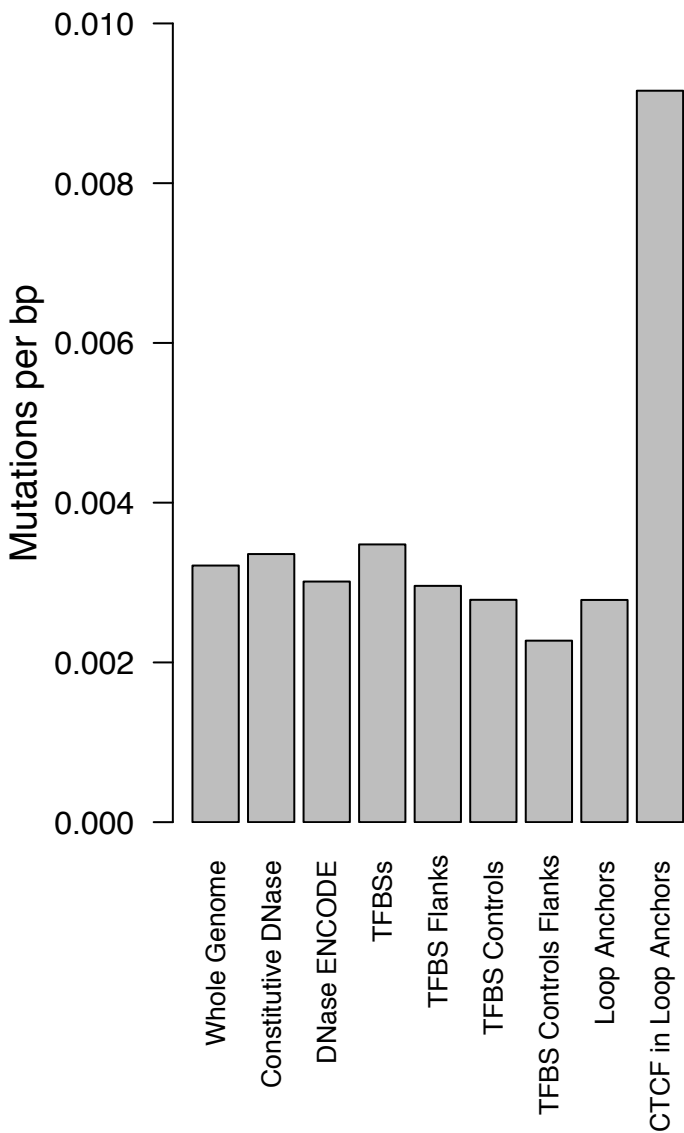

Supplement: S2 Fig — Shown are the rates for the whole genome (outside Duke and Dac excluded regions); regions covered by functional TFBSs; constitutive DNase sites; ENCODE DNase sites; flanks of TFBSs (100bp either side); control TFBSs; flanks of control TFBSs (100bp either side); chromatin loop anchor points; CTCF motifs inside loop anchor points. (PDF) [file pgen.1006207.s002.pdf]

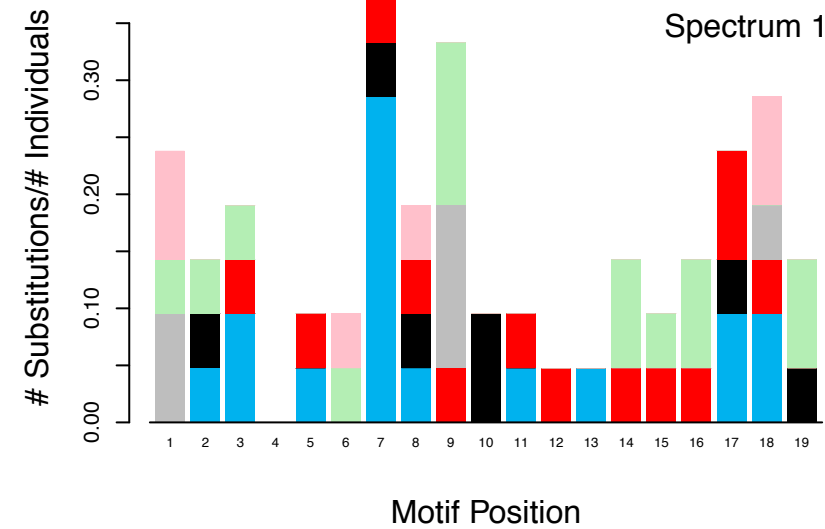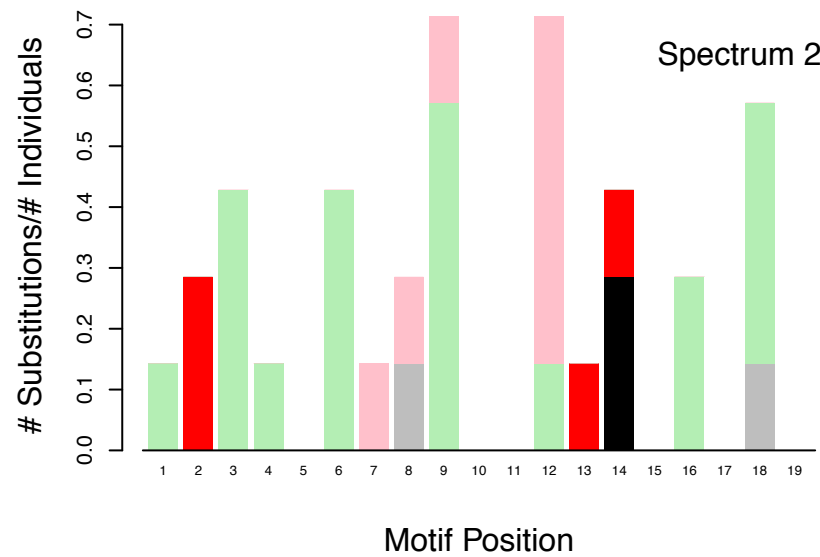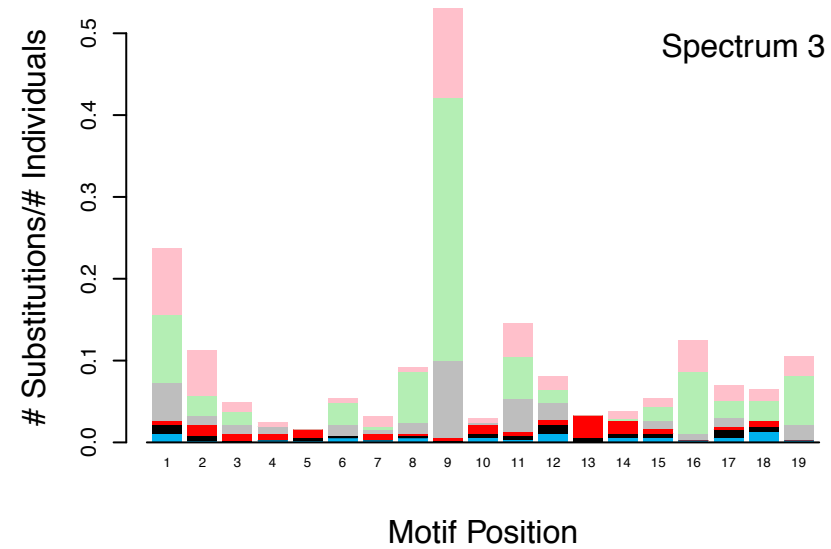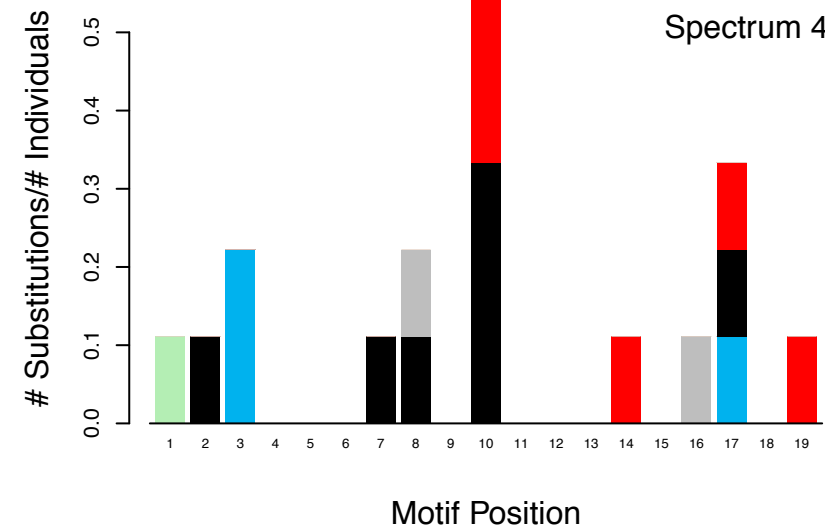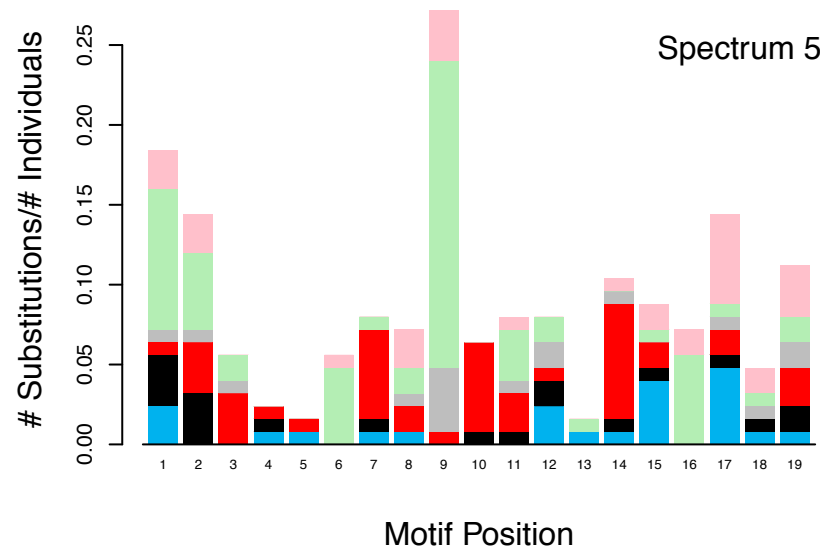

## Legend

- C>A
- C>G
- C>T
- T>A
- T>C
- T>G

Supplement: S4 Fig — Barplots of the mutation count for each functional CTCF motif site, divided by the number of individuals in the mutational spectrum group. (PDF) [file pgen.1006207.s004.pdf]

CTCF-TFBSs inside Loop Anchor Points

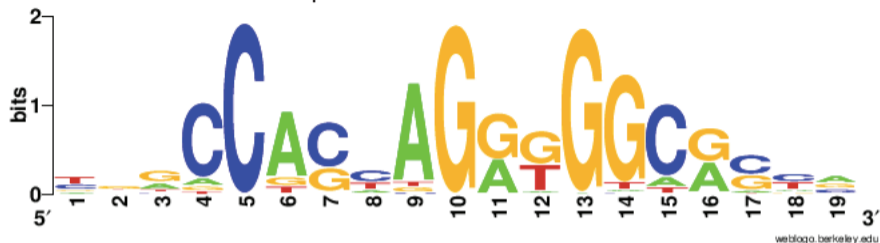

CTCF-TFBSs outside Loop Anchor Points

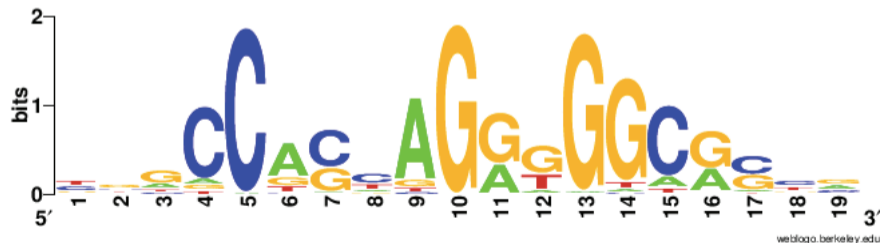

Supplement: S5 Fig — Logos were created using http://weblogo.berkeley.edu/logo.cgi [47]. (PDF) [file pgen.1006207.s005.pdf]

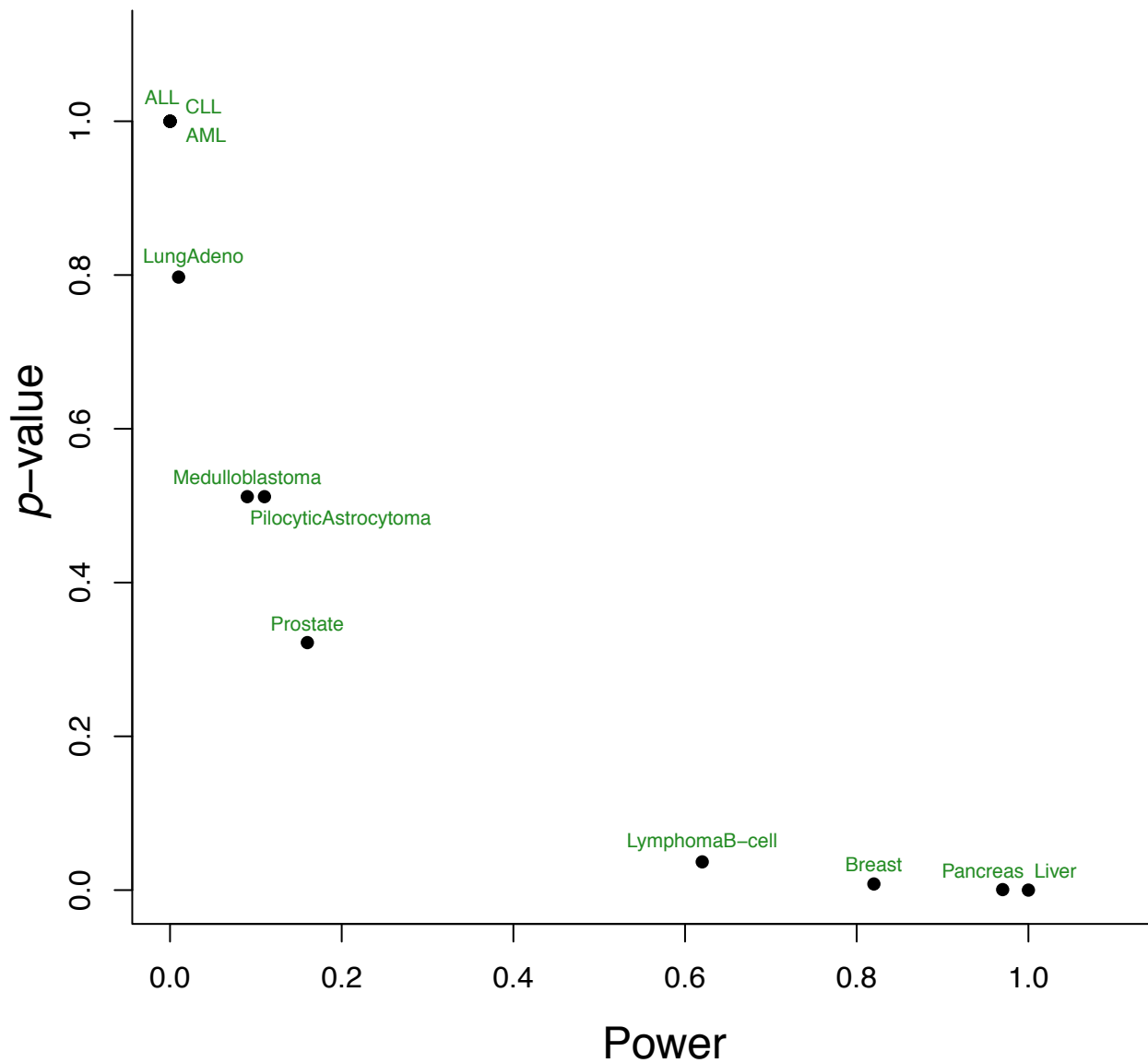

Supplement: S6 Fig — The p-value of Fisher’s exact test, which compares the number of mutations in CTCF-motifs inside chromatin anchor points to the number of mutations in CTCF-motifs outside loop anchor points, is plotted against the power to detect a statistical significance at alpha = 0.05. (PDF) [file pgen.1006207.s006.pdf]

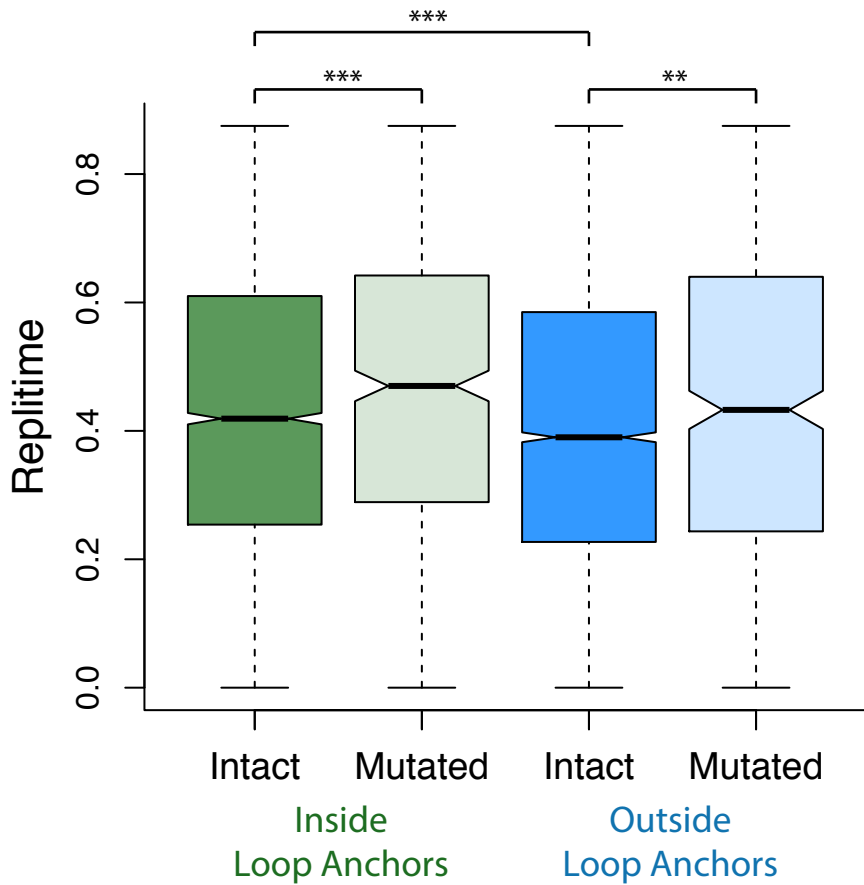

Supplement: S7 Fig — Larger values on the y-axis indicate later replication. (PDF) [file pgen.1006207.s007.pdf]

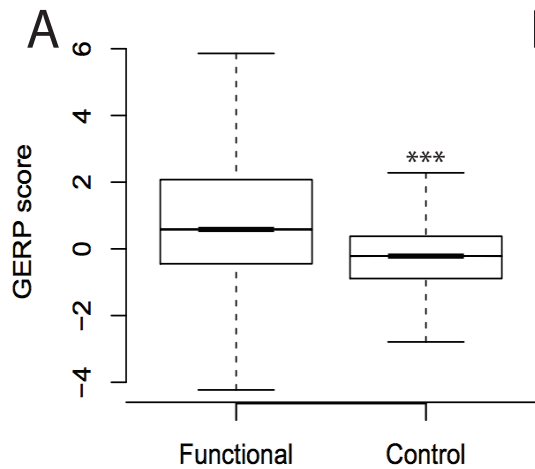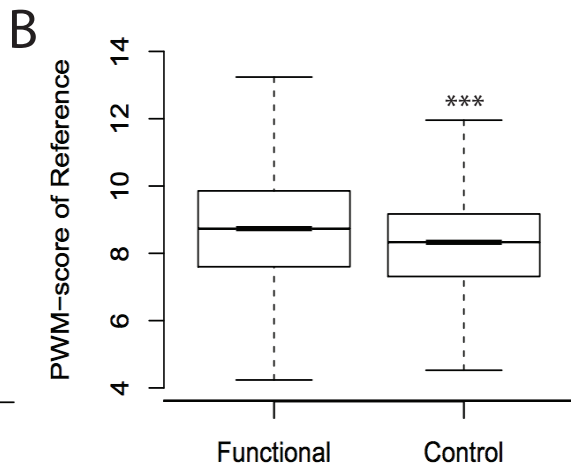

Supplement: S9 Fig — Boxplots of the GERP conservation scores (A) and PWM-scores (B) of functional and control motifs, respectively. In both plots, asterisks indicate p-values of the Wilcoxon test of p < 10−15. (PDF) [file pgen.1006207.s009.pdf]
