# Supplementary material for: Mutational Biases Drive Elevated Rates of Substitution at Regulatory Sites across Cancer Types
Source: PLoS Genet. 2016 Aug 4;12(8):e1006207. doi: 10.1371/journal.pgen.1006207 (PMC4973979; doi:10.1371/journal.pgen.1006207)
Supplement: S8 Fig — (PDF) [file pgen.1006207.s008.pdf]

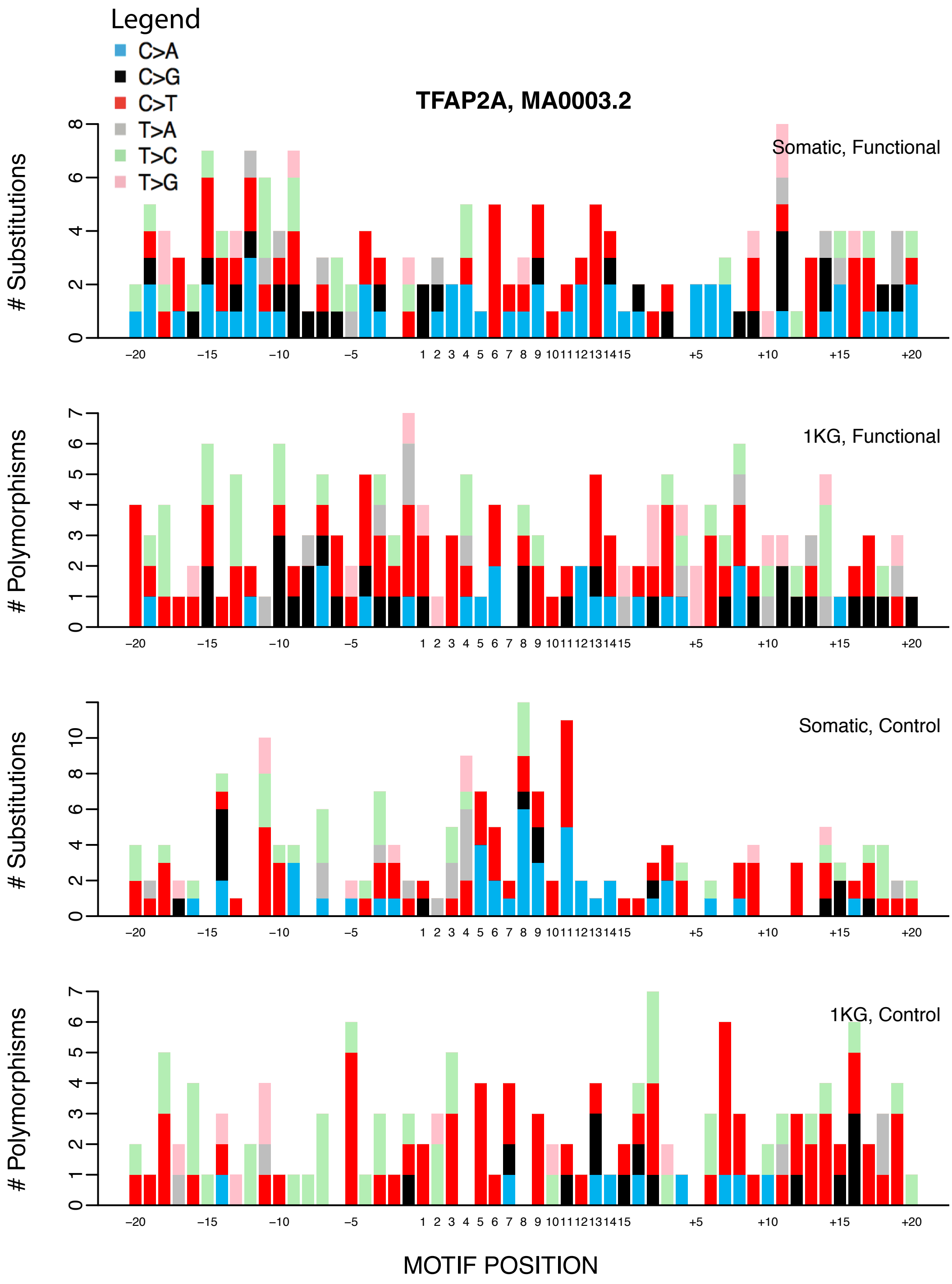
Legend
C>A
C>G
C>T
T>A
T>C
T>G
TFAP2A, MA0003.2
Substitutions
Somatic, Functional
Polymorphisms
1KG, Functional
Somatic, Control
1KG, Control
−20 −15 −10 −5 1 2 3 4 5 6 7 8 9 10 11 12 13 14 15 +5 +10 +15 +20
MOTIF POSITION

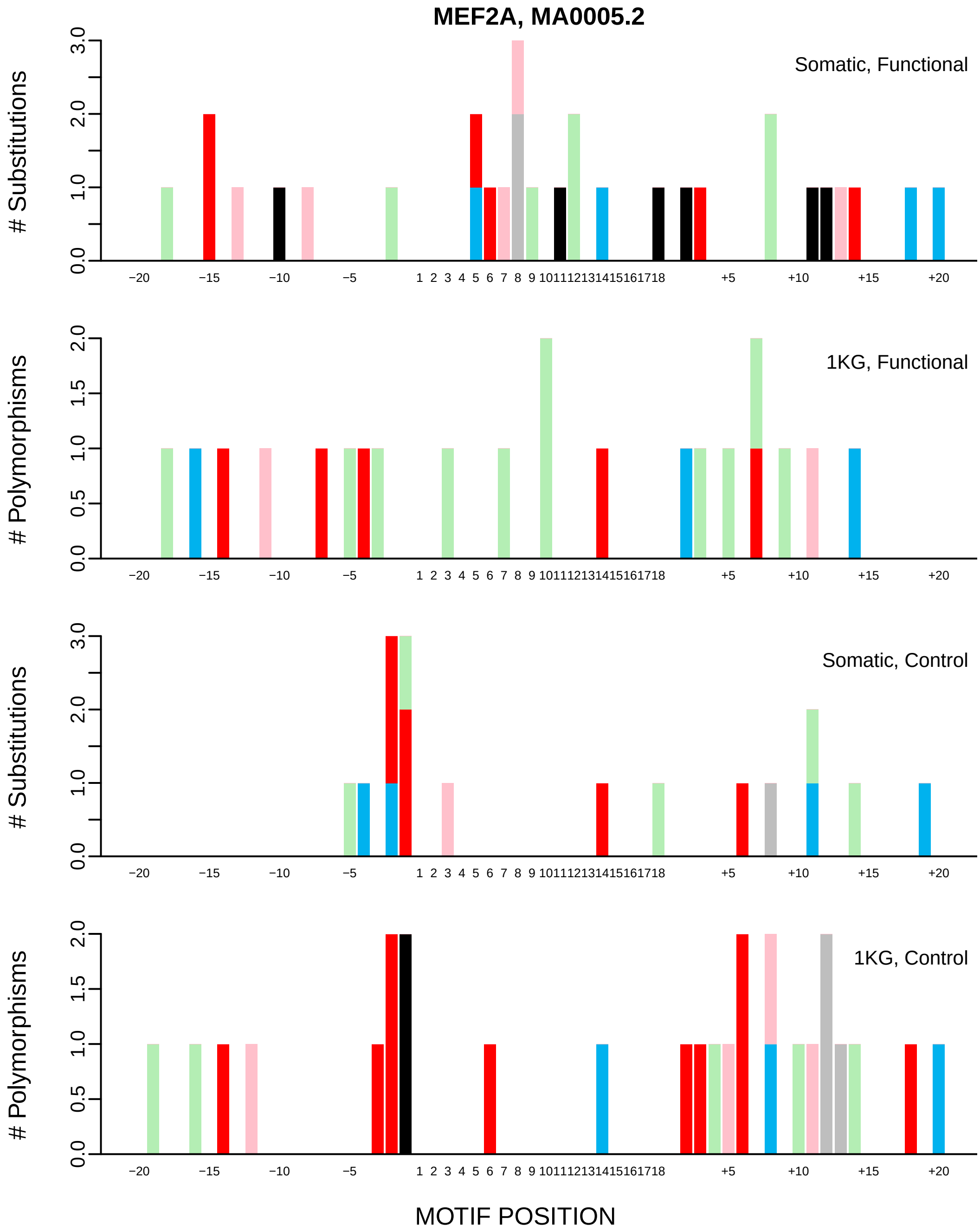

MEF2A, MA0005.2
Substitutions
Somatic, Functional
Polymorphisms
1KG, Functional
Substitutions
Somatic, Control
Polymorphisms
1KG, Control
MOTIF POSITION

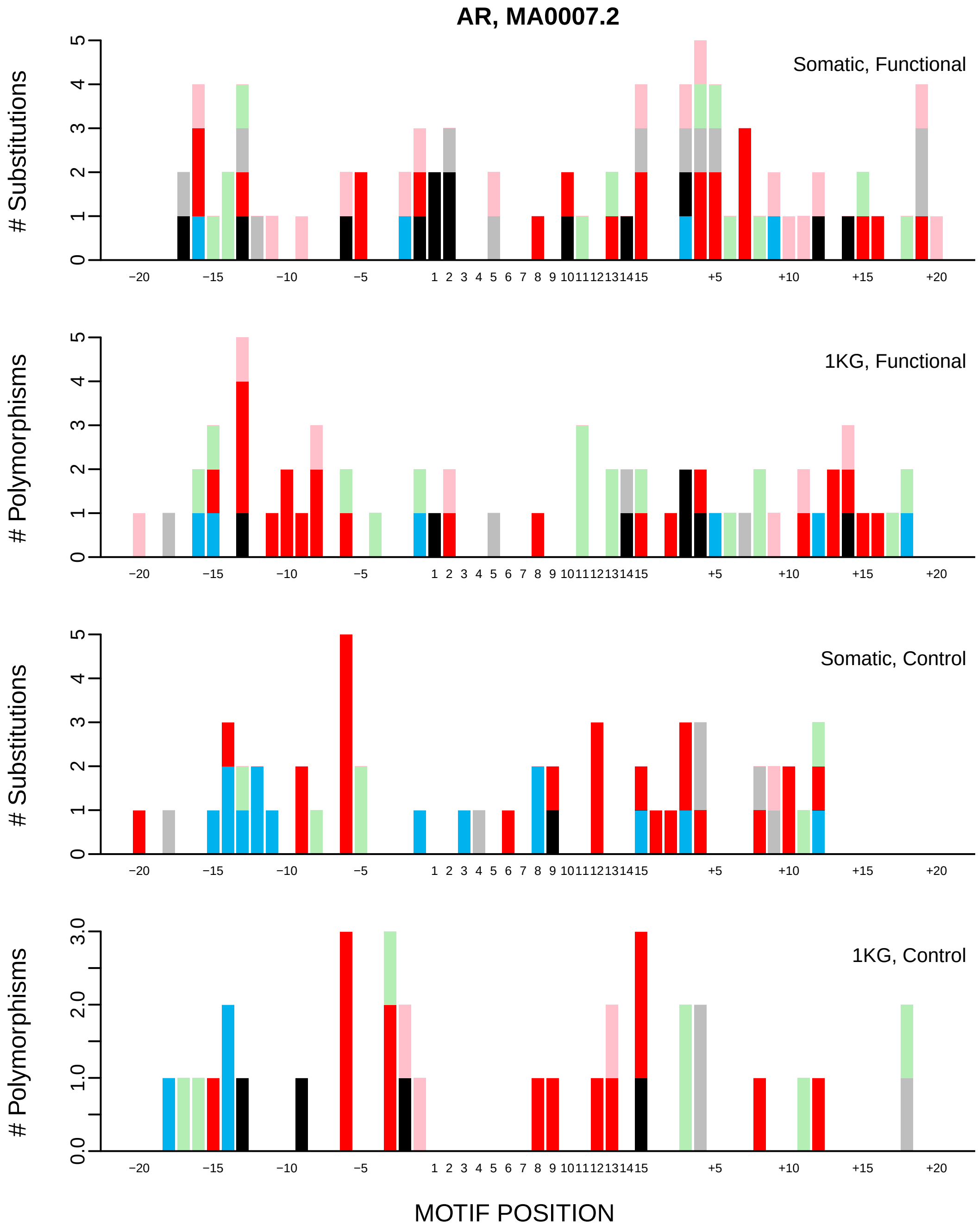

AR, MA0007.2
Substitutions
Somatic, Functional
Polymorphisms
1KG, Functional
Substitutions
Somatic, Control
Polymorphisms
1KG, Control
−20 −15 −10 −5 1 2 3 4 5 6 7 8 9 10 11 12 13 14 15 +5 +10 +15 +20
MOTIF POSITION

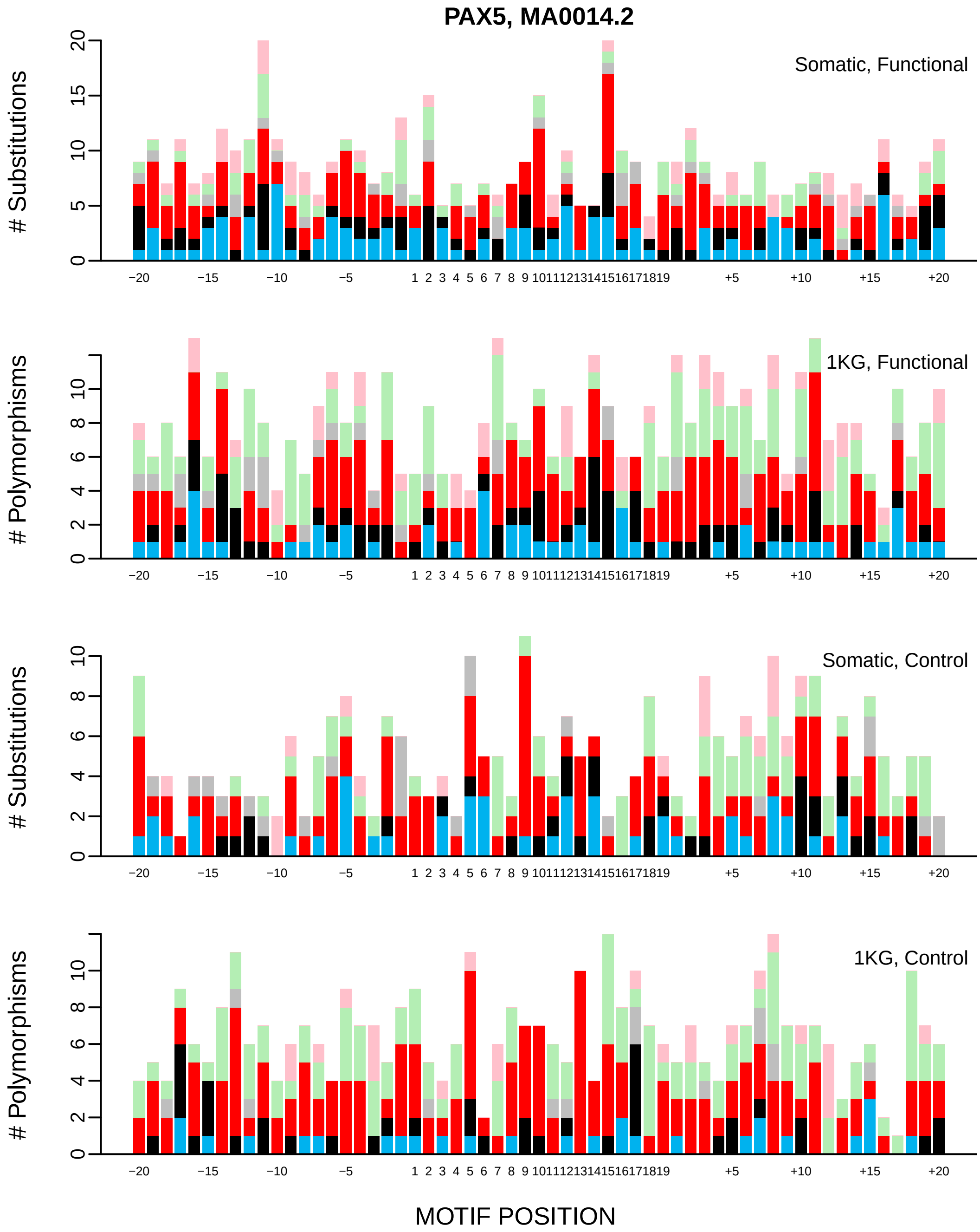
PAX5, MA0014.2
Substitutions
Somatic, Functional
Polymorphisms
1KG, Functional
Substitutions
Somatic, Control
Polymorphisms
1KG, Control
−20
−15
−10
−5
1 2 3 4 5 6 7 8 9 10111213141516171819
+5
+10
+15
+20
MOTIF POSITION

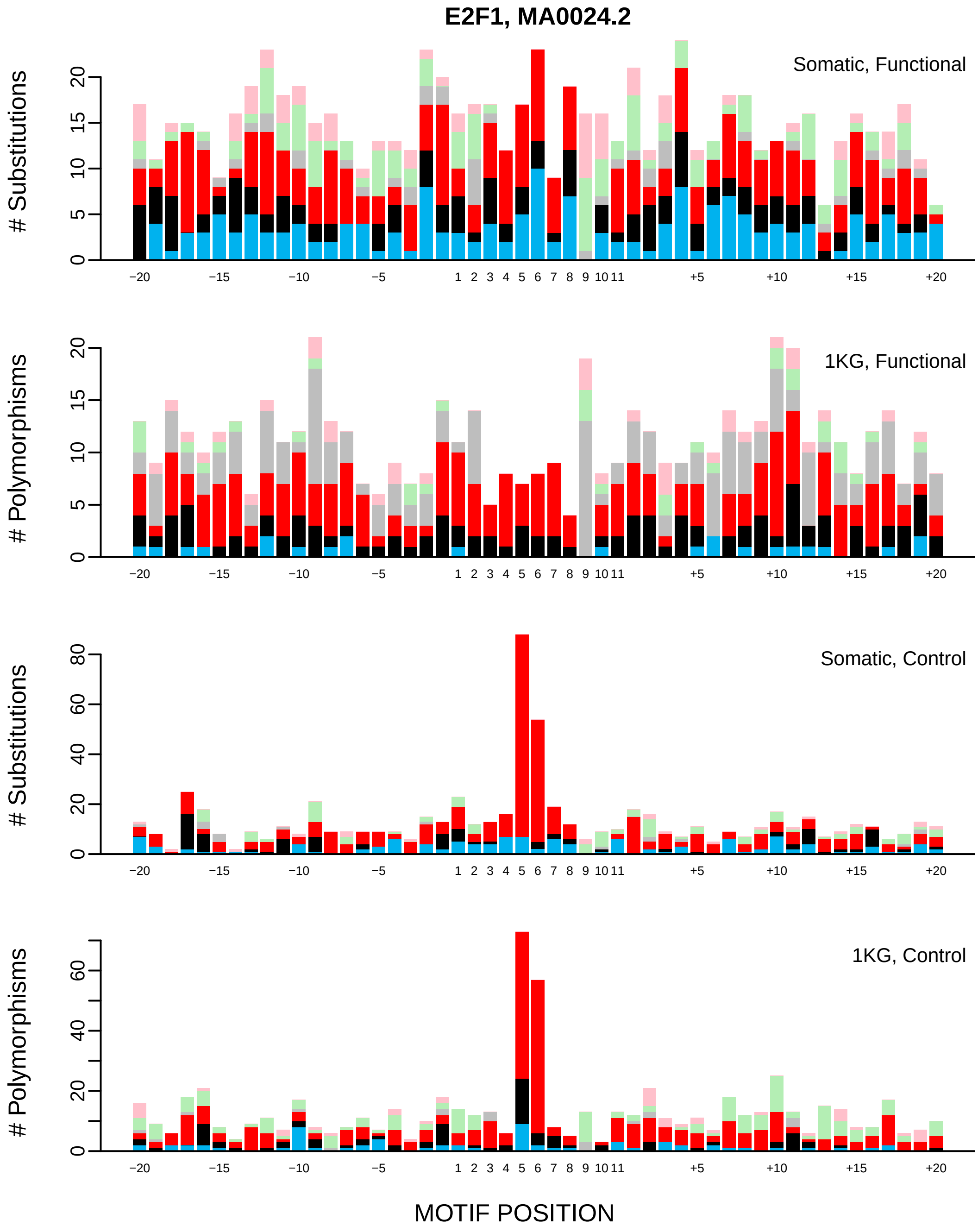
E2F1, MA0024.2
Somatic, Functional
Substitutions
0
5
10
15
20
−20
−15
−10
−5
1 2 3 4 5 6 7 8 9 10 11
+5
+10
+15
+20
1KG, Functional
Polymorphisms
Somatic, Control
0
20
40
60
80
1KG, Control
MOTIF POSITION

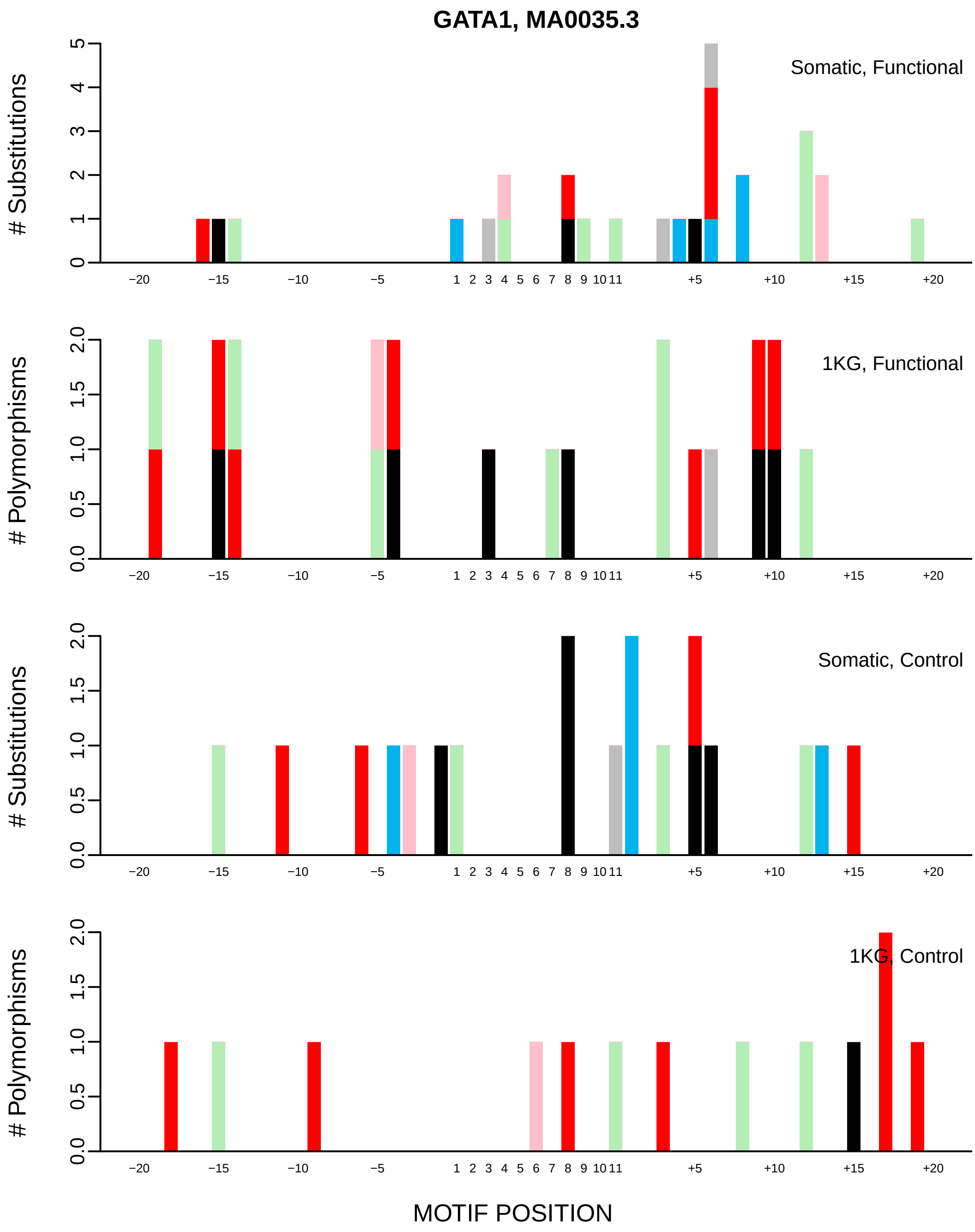
GATA1, MA0035.3
Somatic, Functional
1KG, Functional
Somatic, Control
1KG, Control
Substitutions
Polymorphisms
MOTIF POSITION
−20
−15
−10
−5
1 2 3 4 5 6 7 8 9 10 11
+5
+10
+15
+20

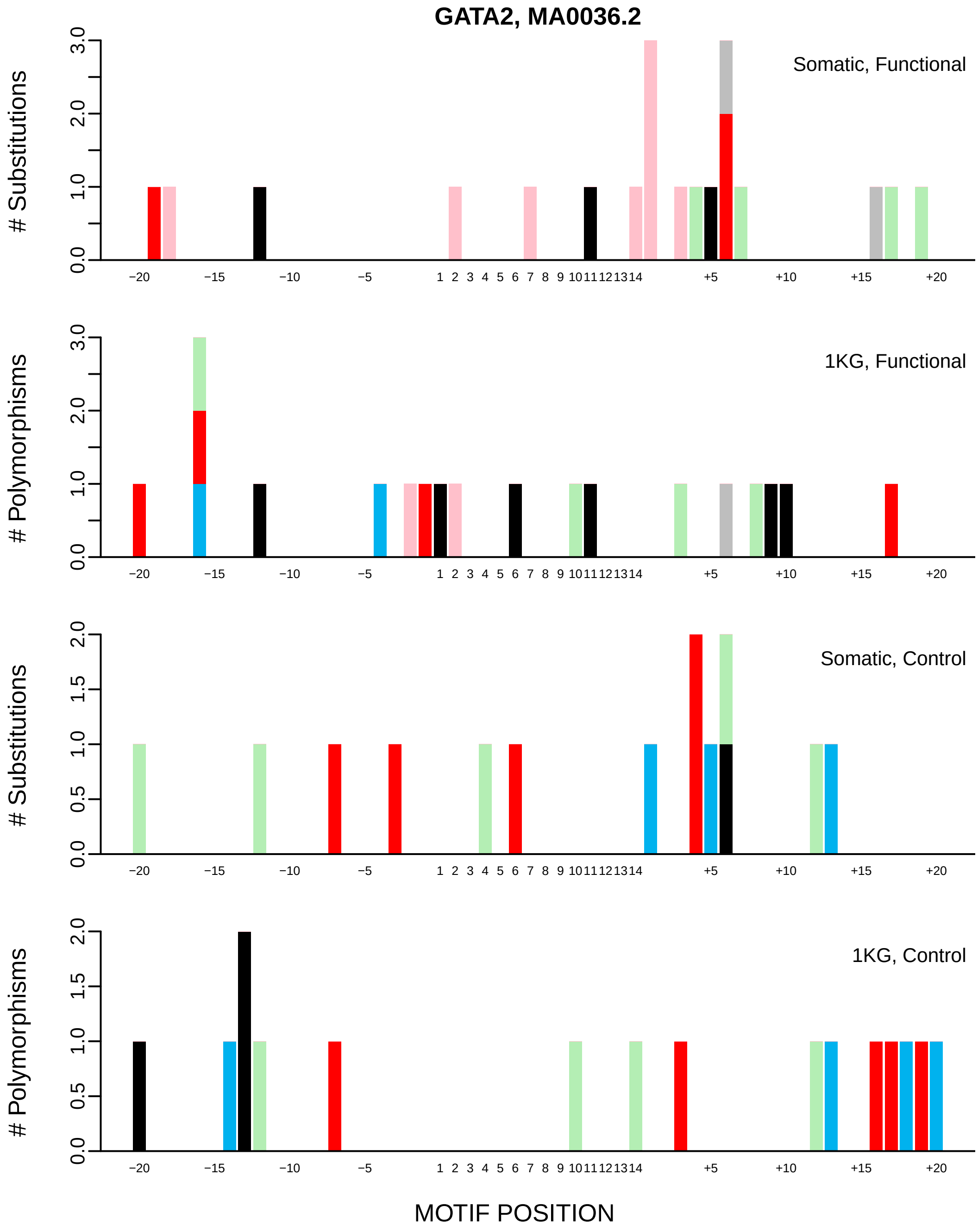
GATA2, MA0036.2
Substitutions
Somatic, Functional
Polymorphisms
1KG, Functional
Substitutions
Somatic, Control
Polymorphisms
1KG, Control
−20 −15 −10 −5 1 2 3 4 5 6 7 8 9 10 11 12 13 14 +5 +10 +15 +20
MOTIF POSITION

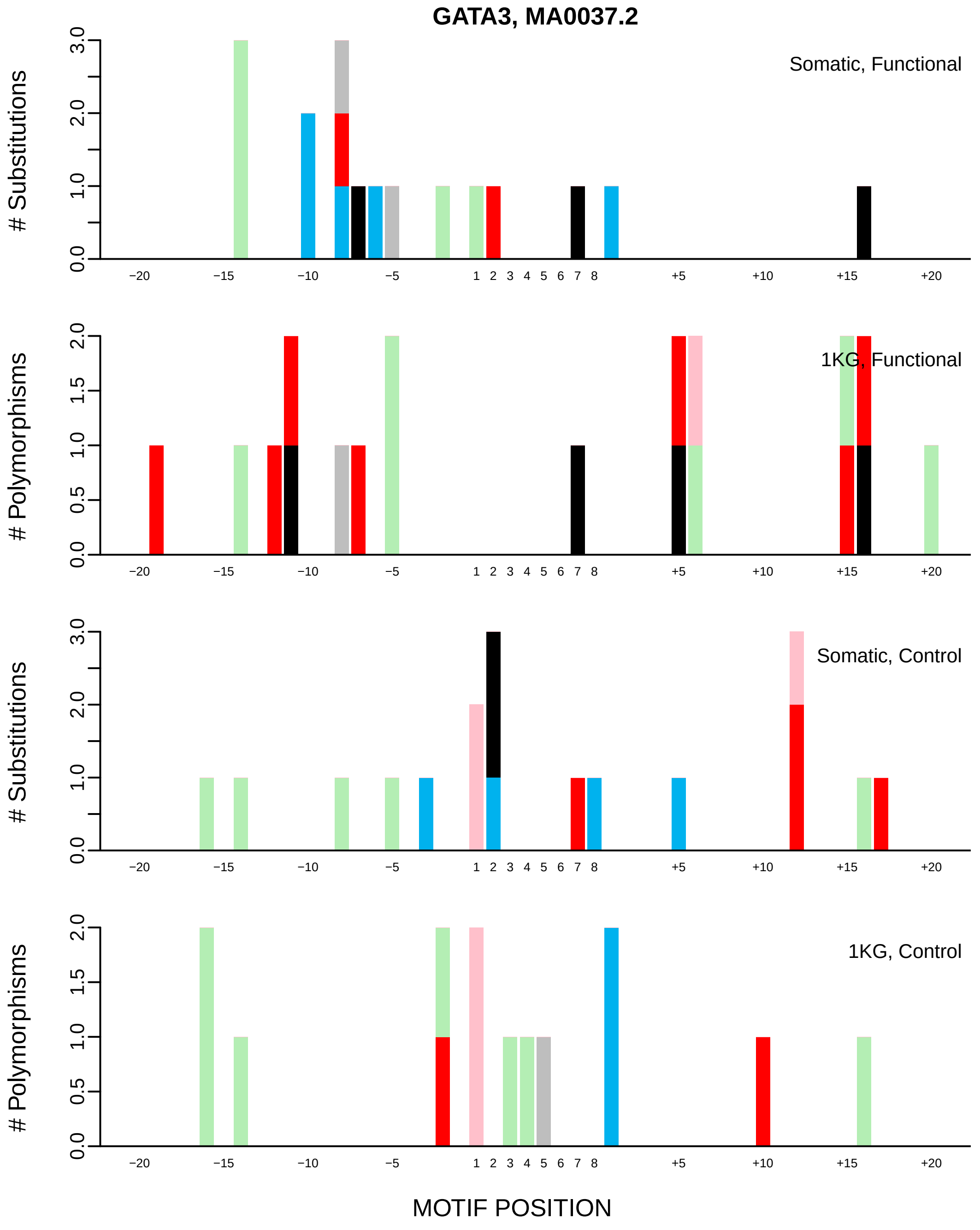
GATA3, MA0037.2
Somatic, Functional
1KG, Functional
Somatic, Control
1KG, Control
Substitutions
Polymorphisms
−20 −15 −10 −5 1 2 3 4 5 6 7 8 +5 +10 +15 +20
MOTIF POSITION

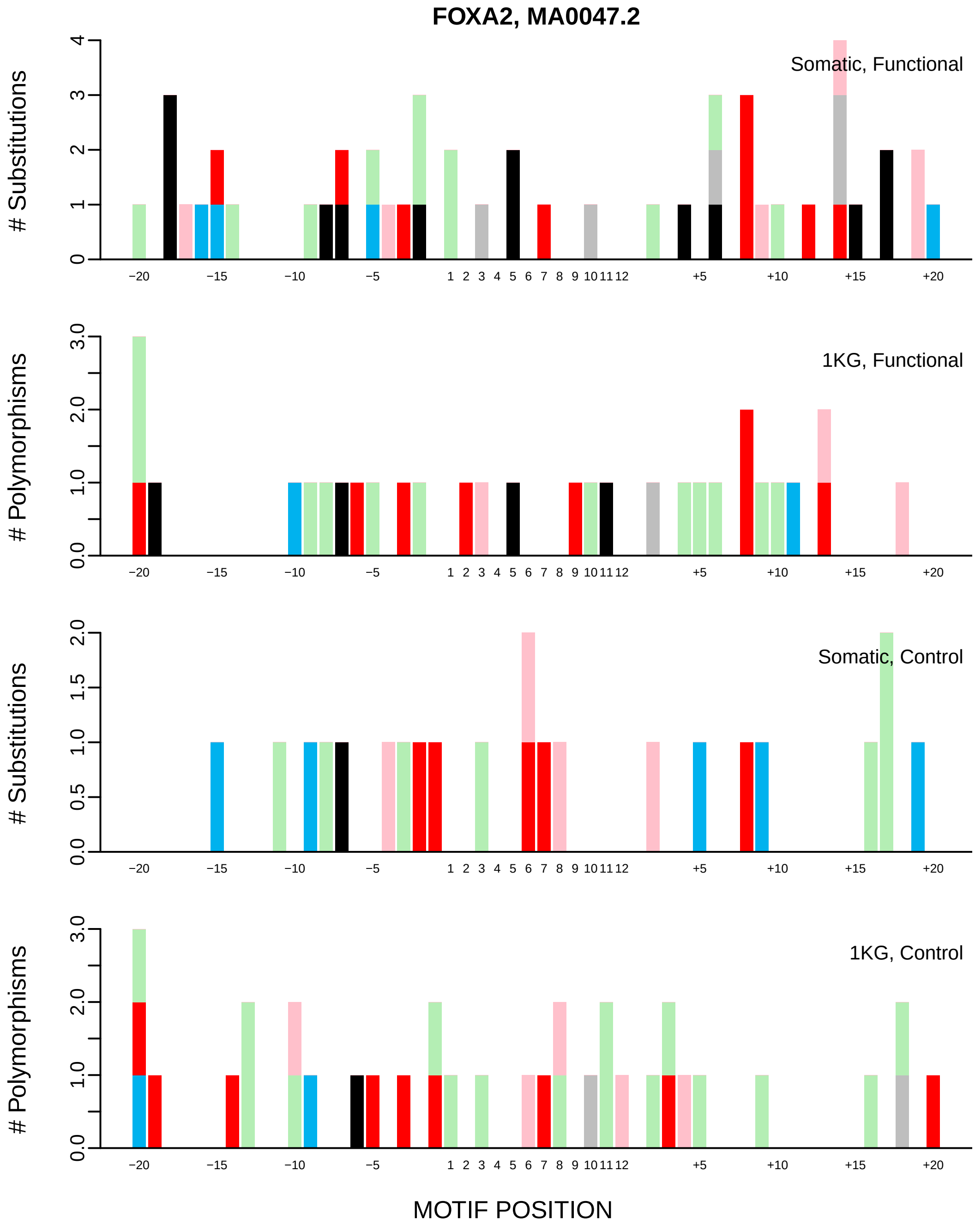
FOXA2, MA0047.2
Substitutions
Somatic, Functional
Polymorphisms
1KG, Functional
Substitutions
Somatic, Control
Polymorphisms
1KG, Control
−20
−15
−10
−5
1 2 3 4 5 6 7 8 9 10 11 12
+5
+10
+15
+20
MOTIF POSITION

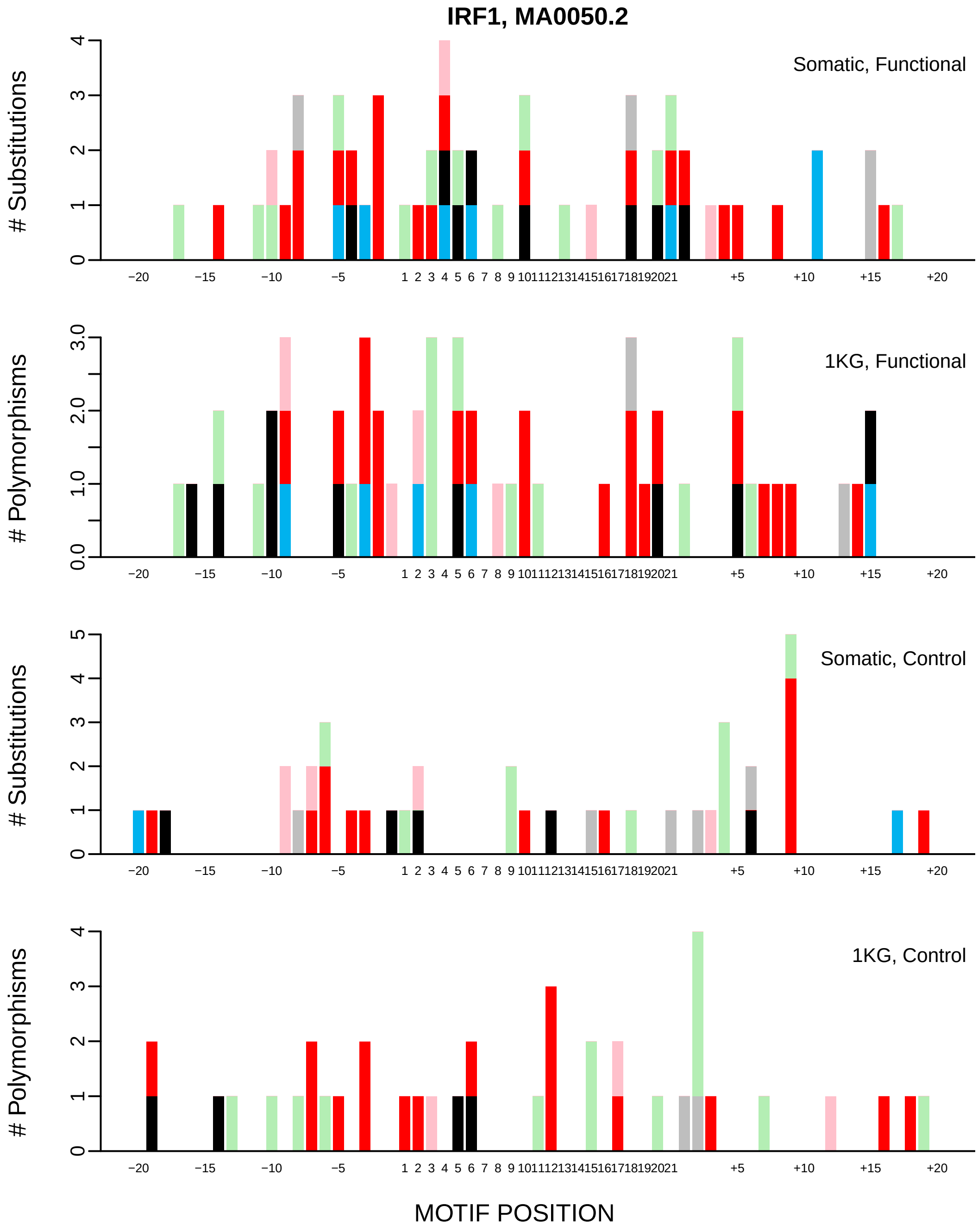
IRF1, MA0050.2
Substitutions
Somatic, Functional
Polymorphisms
1KG, Functional
Substitutions
Somatic, Control
Polymorphisms
1KG, Control
−20 −15 −10 −5 1 2 3 4 5 6 7 8 9 10 11 12 13 14 15 16 17 18 19 20 21 +5 +10 +15 +20
MOTIF POSITION

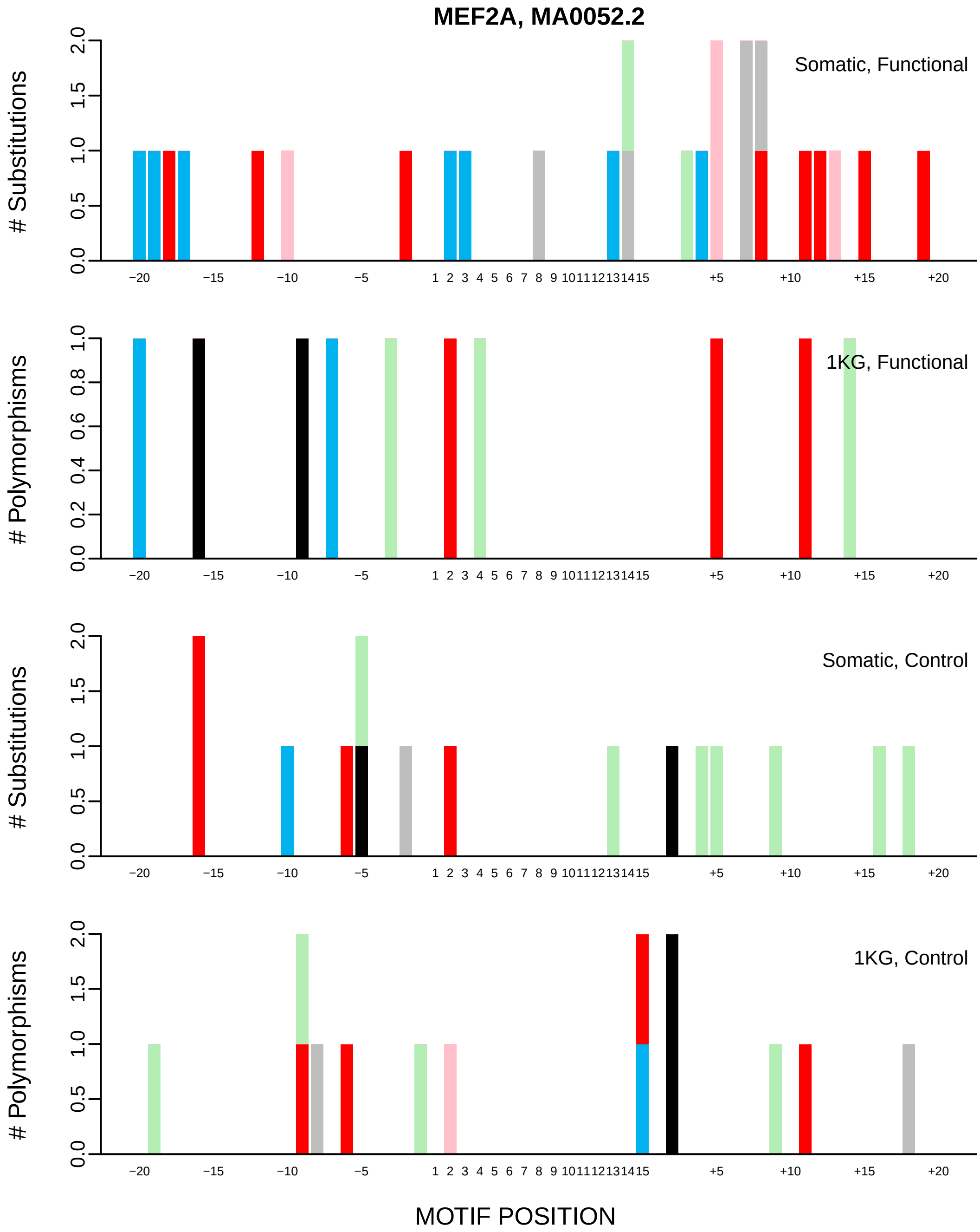
MEF2A, MA0052.2
Substitutions
Somatic, Functional
Polymorphisms
1KG, Functional
Substitutions
Somatic, Control
Polymorphisms
1KG, Control
MOTIF POSITION

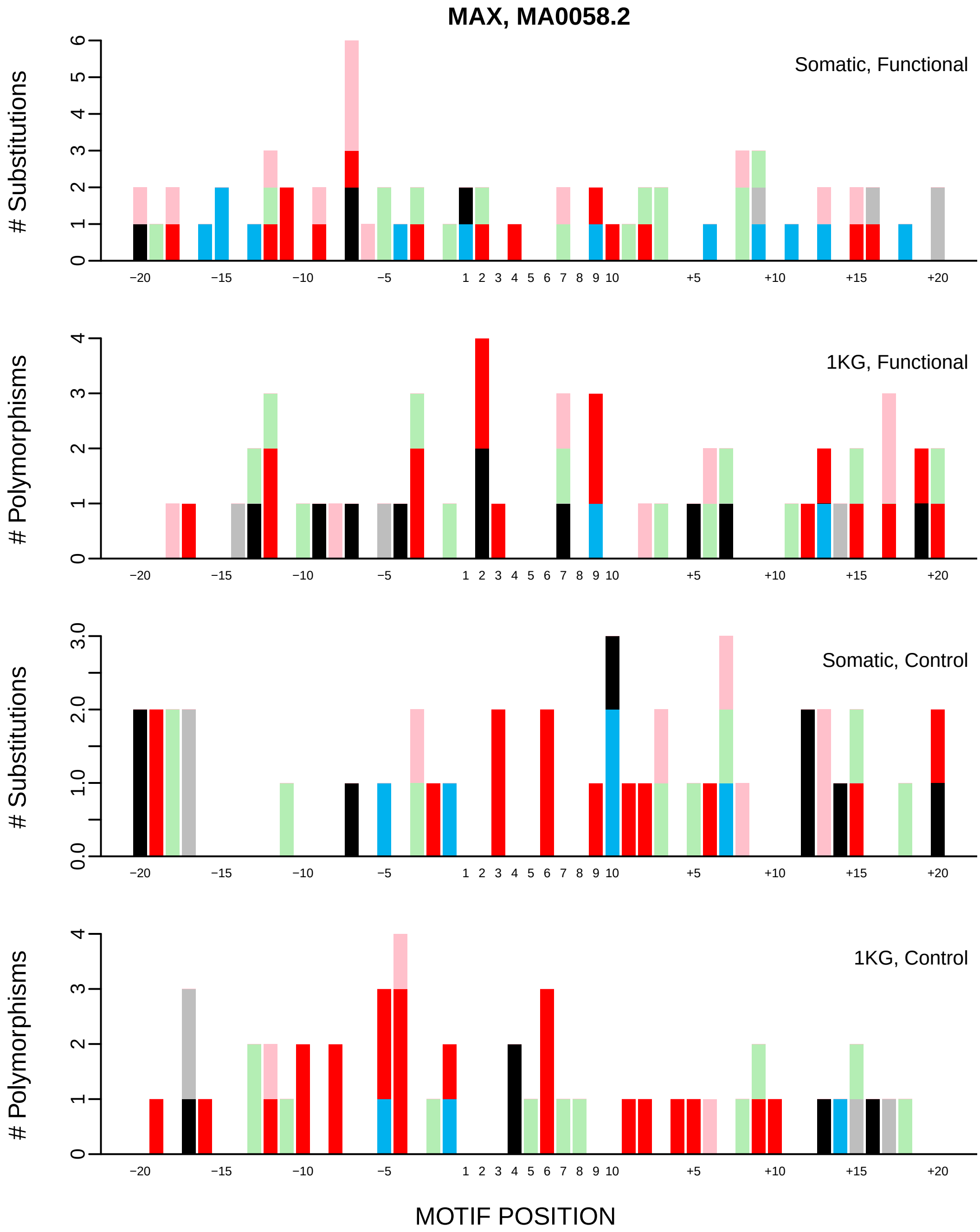

MAX, MA0058.2
Somatic, Functional
1KG, Functional
Somatic, Control
1KG, Control
Substitutions
Polymorphisms
−20
−15
−10
−5
1 2 3 4 5 6 7 8 9 10
+5
+10
+15
+20
MOTIF POSITION

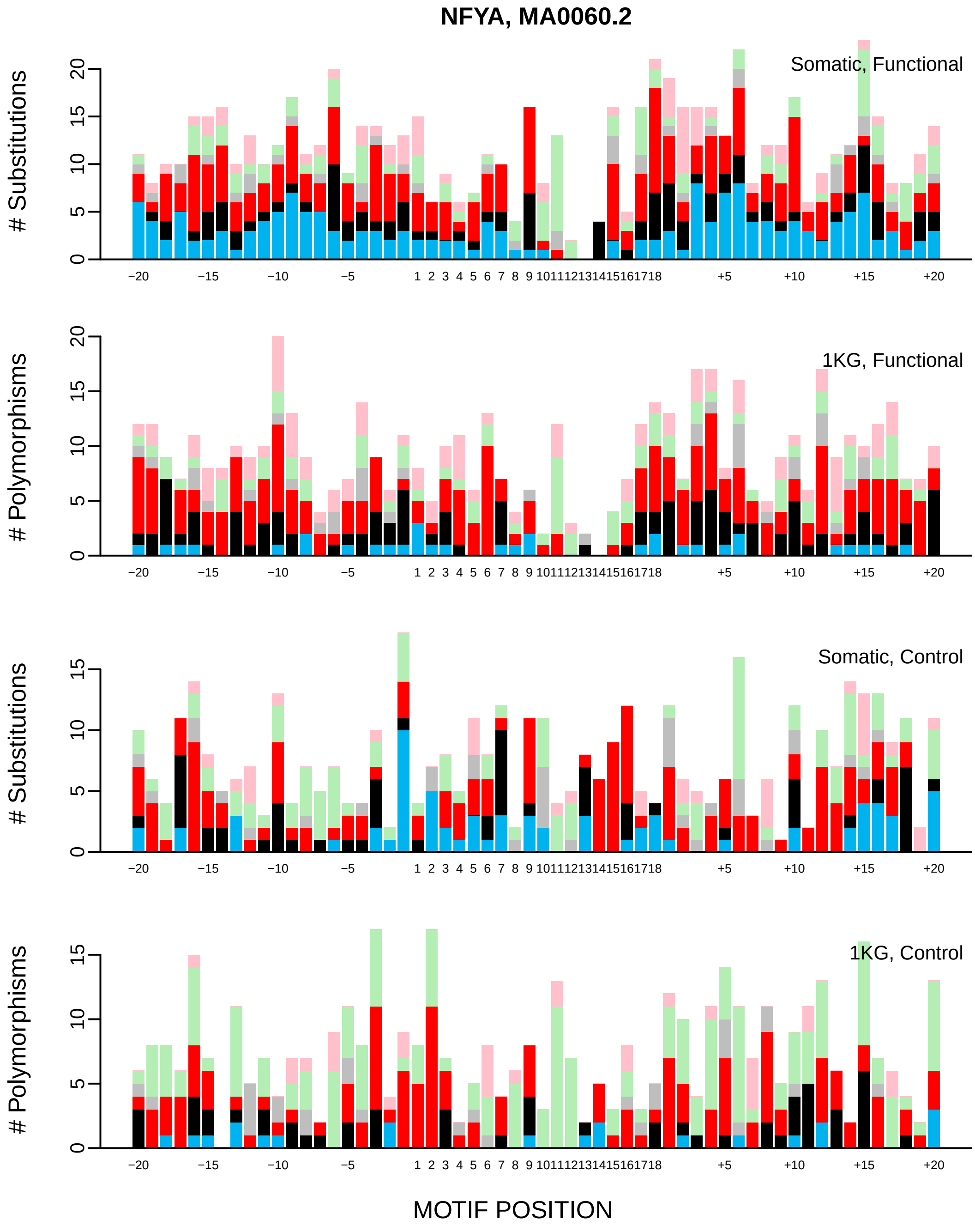

NFYA, MA0060.2
Somatic, Functional
1KG, Functional
Somatic, Control
1KG, Control
Substitutions
Polymorphisms
−20 −15 −10 −5 1 2 3 4 5 6 7 8 9 10 11 12 13 14 15 16 17 18 +5 +10 +15 +20
MOTIF POSITION

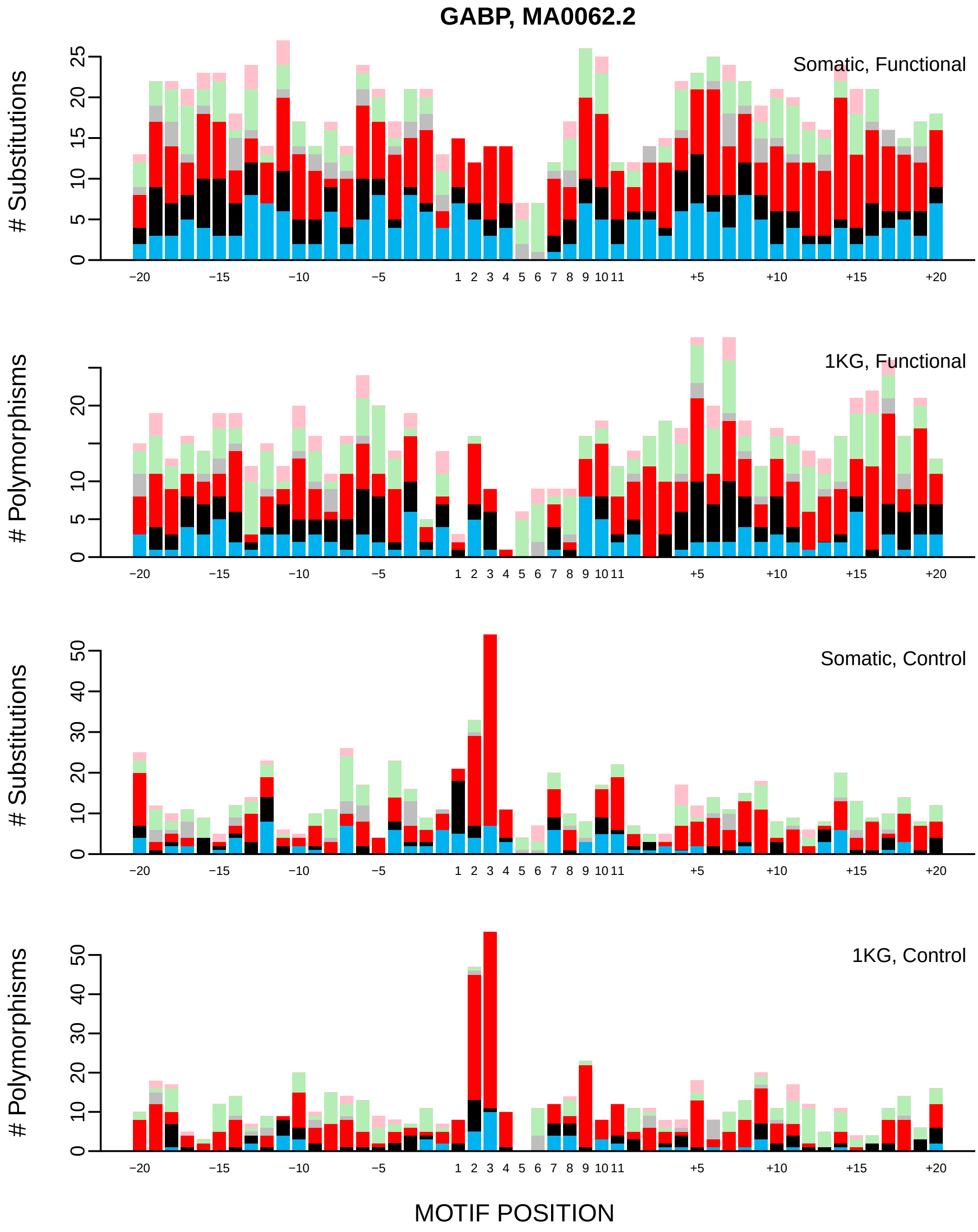
GABP, MA0062.2
Substitutions
Somatic, Functional
Polymorphisms
1KG, Functional
Substitutions
Somatic, Control
Polymorphisms
1KG, Control
−20 −15 −10 −5 1 2 3 4 5 6 7 8 9 10 11 +5 +10 +15 +20
MOTIF POSITION

ELK4, MA0076.2

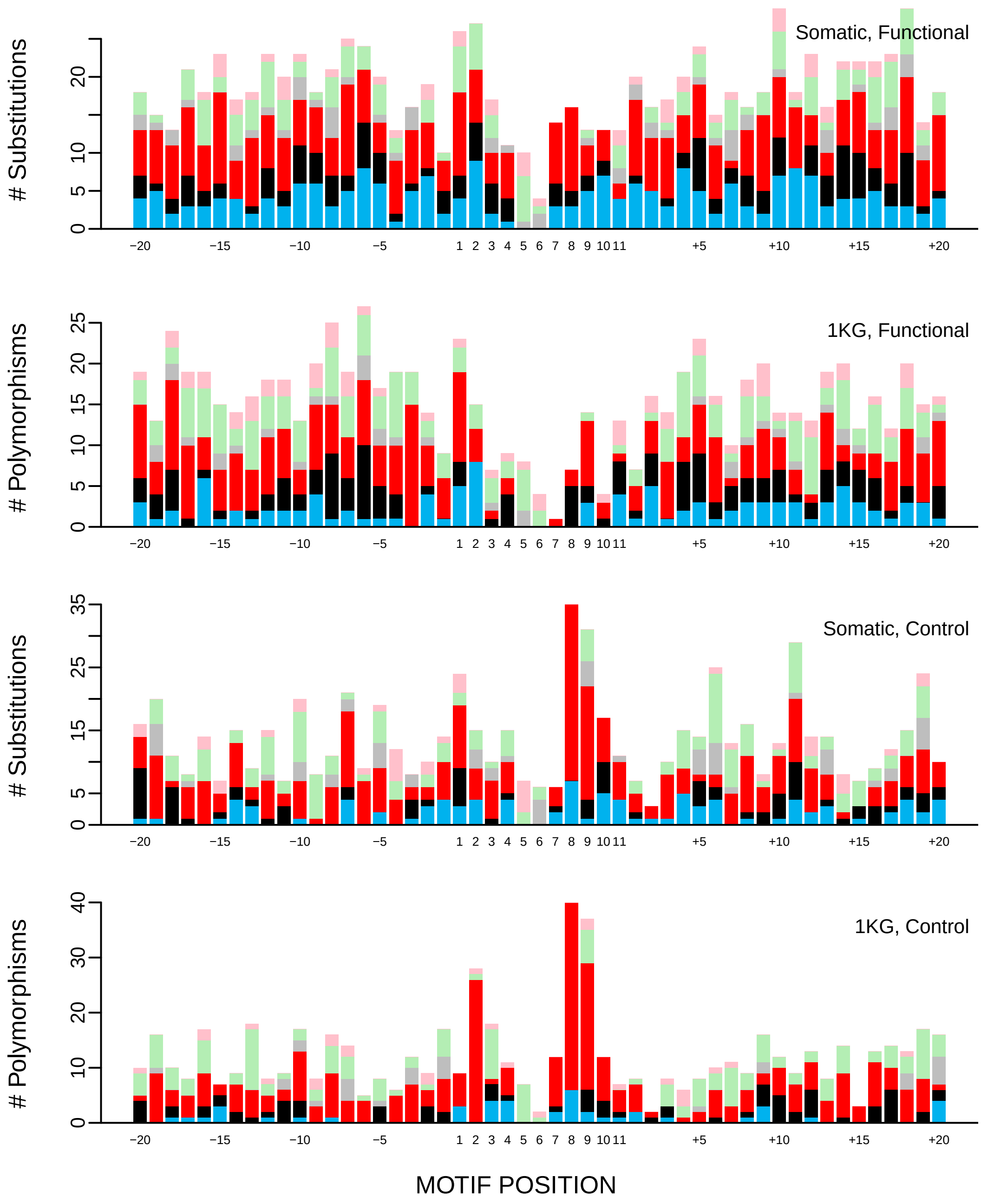

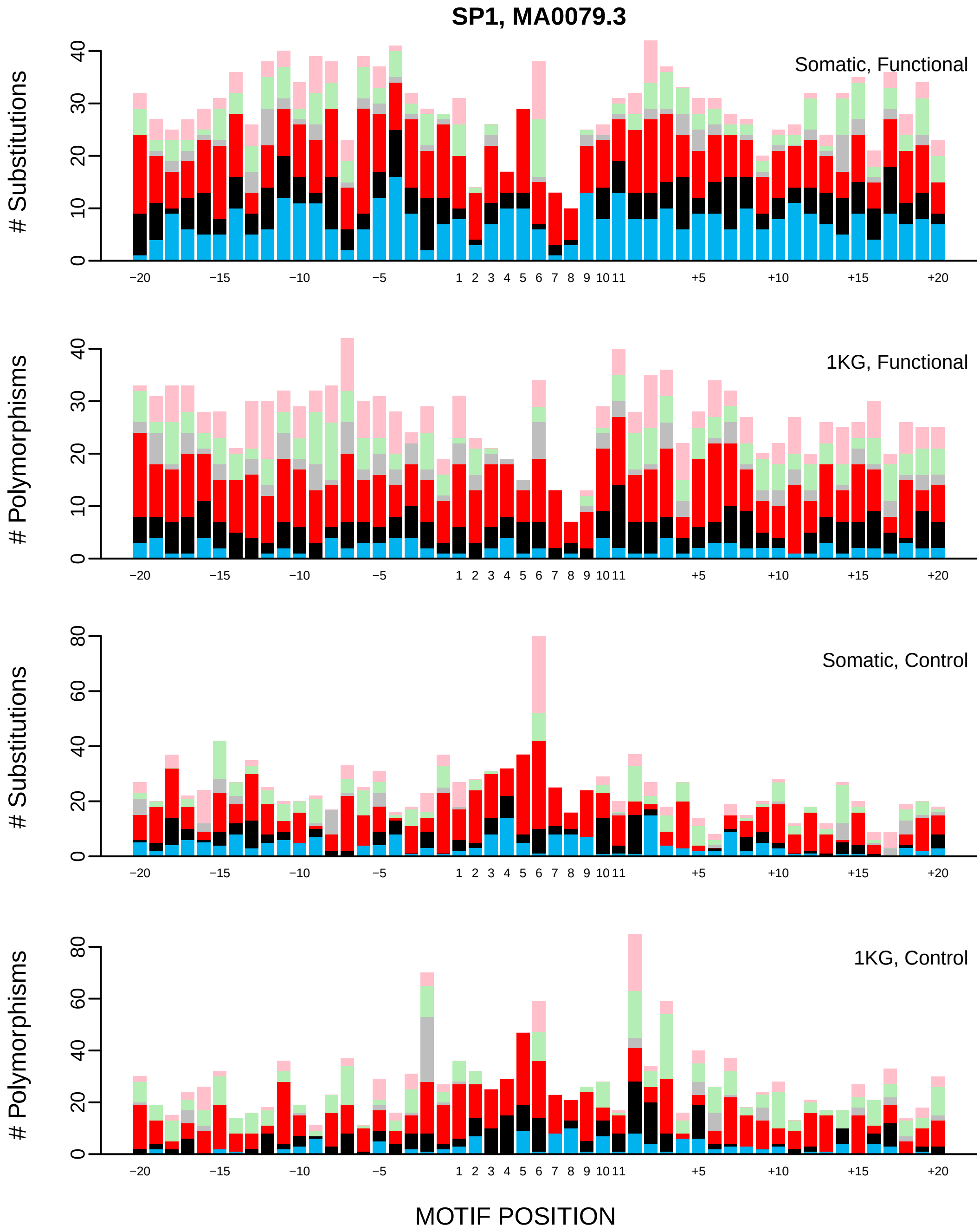
SP1, MA0079.3
Substitutions
Somatic, Functional
Polymorphisms
1KG, Functional
Substitutions
Somatic, Control
Polymorphisms
1KG, Control
−20
−15
−10
−5
1 2 3 4 5 6 7 8 9 10 11
+5
+10
+15
+20
MOTIF POSITION

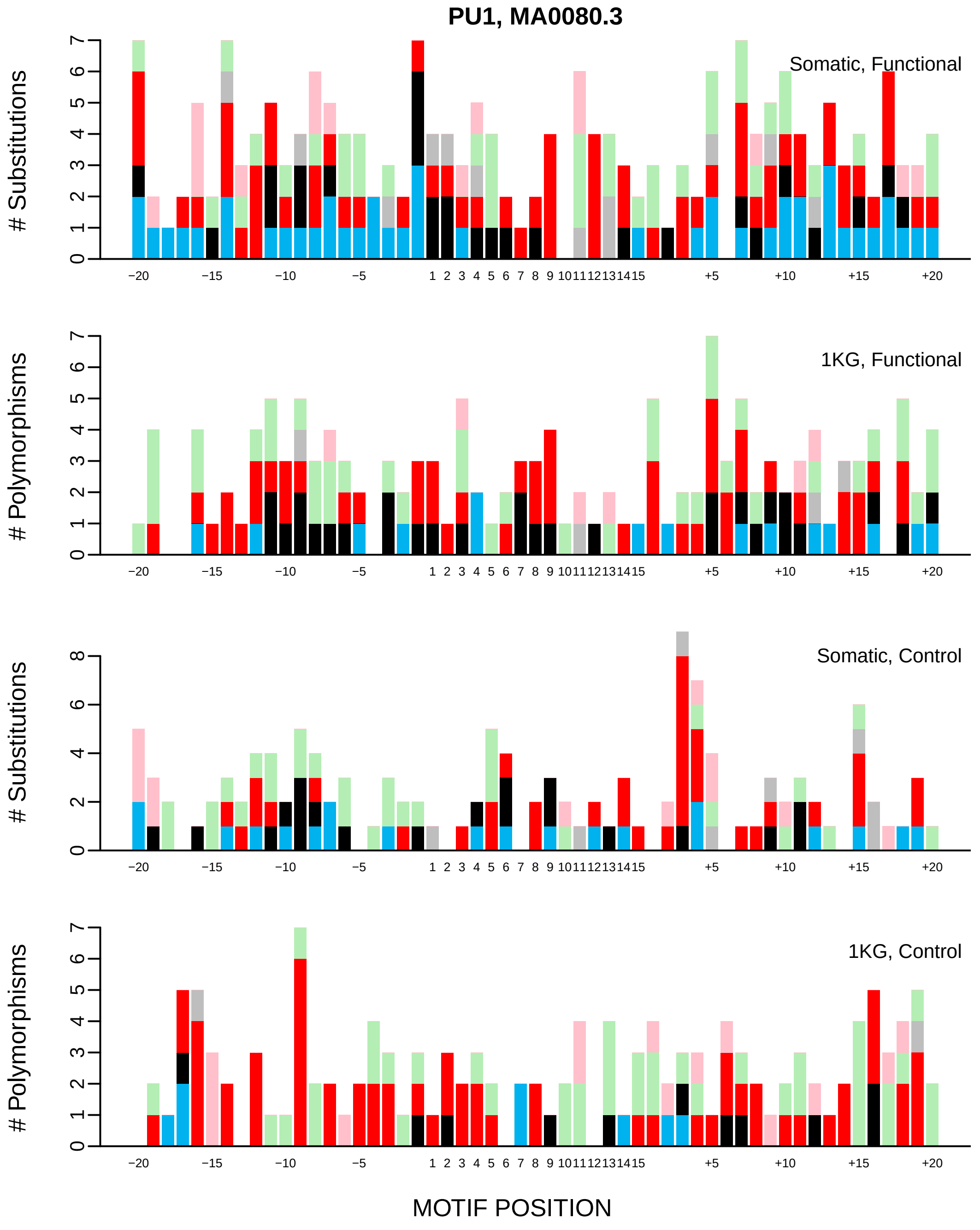
PU1, MA0080.3
Somatic, Functional
Substitutions
1KG, Functional
Polymorphisms
Somatic, Control
Substitutions
1KG, Control
Polymorphisms
−20
−15
−10
−5
1 2 3 4 5 6 7 8 9 10 11 12 13 14 15
+5
+10
+15
+20
MOTIF POSITION

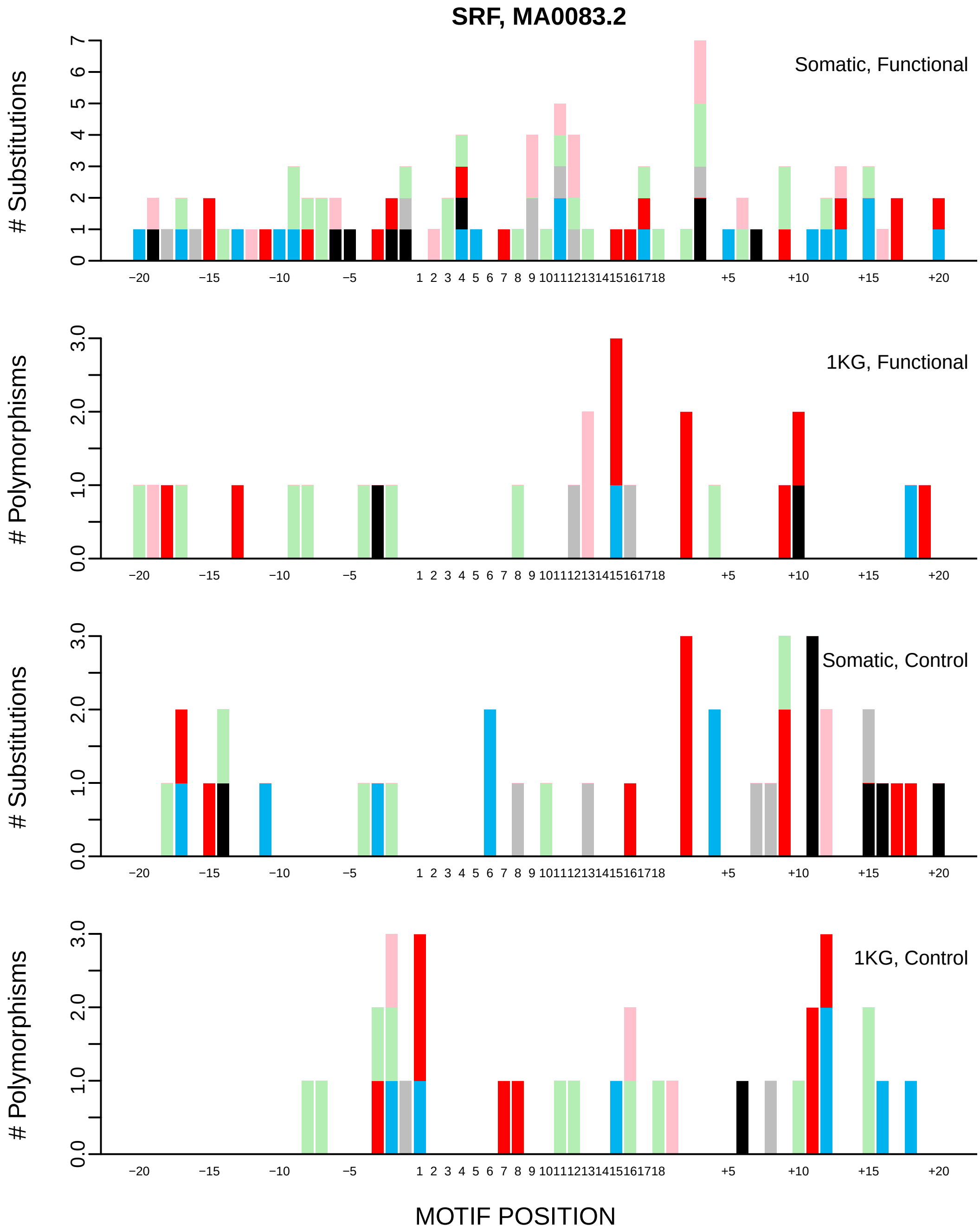
SRF, MA0083.2
Substitutions
Somatic, Functional
Polymorphisms
1KG, Functional
Substitutions
Somatic, Control
Polymorphisms
1KG, Control
−20 −15 −10 −5 1 2 3 4 5 6 7 8 9 10 11 12 13 14 15 16 17 18 +5 +10 +15 +20
MOTIF POSITION

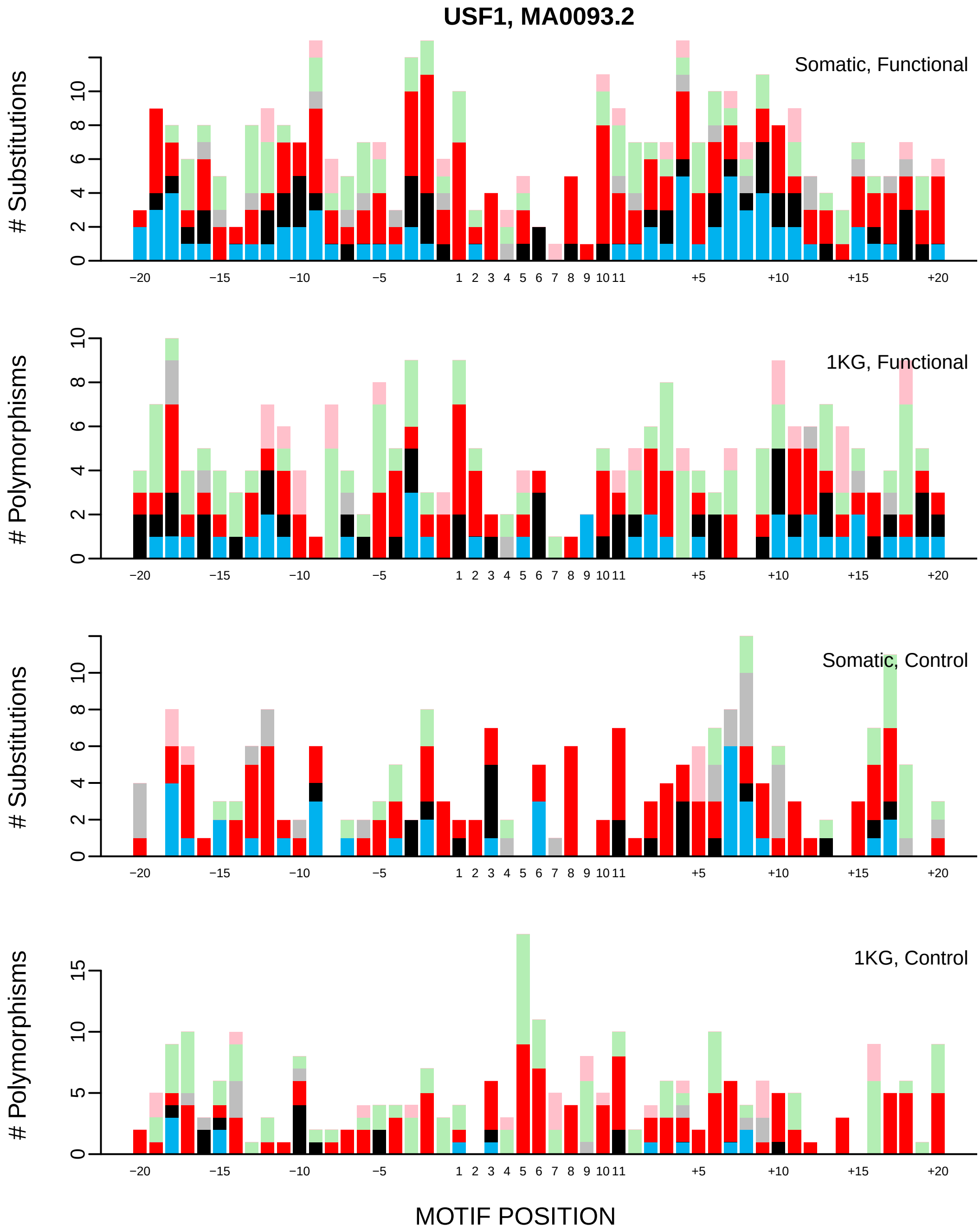
USF1, MA0093.2
Somatic, Functional
1KG, Functional
Somatic, Control
1KG, Control
Substitutions
Polymorphisms
Substitutions
Polymorphisms
−20
−15
−10
−5
1 2 3 4 5 6 7 8 9 10 11
+5
+10
+15
+20
MOTIF POSITION

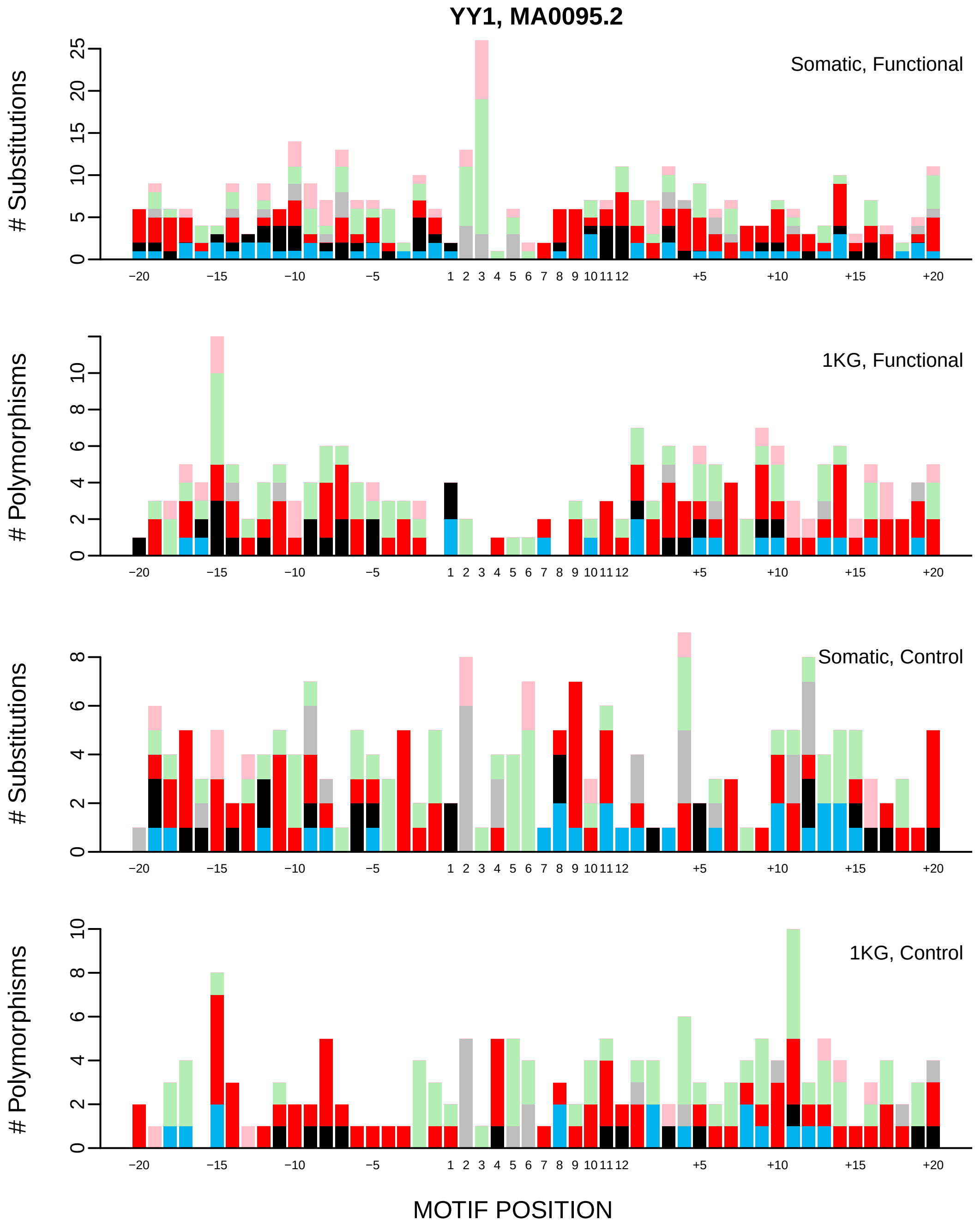
YY1, MA0095.2
Substitutions
Somatic, Functional
Polymorphisms
1KG, Functional
Substitutions
Somatic, Control
Polymorphisms
1KG, Control
MOTIF POSITION

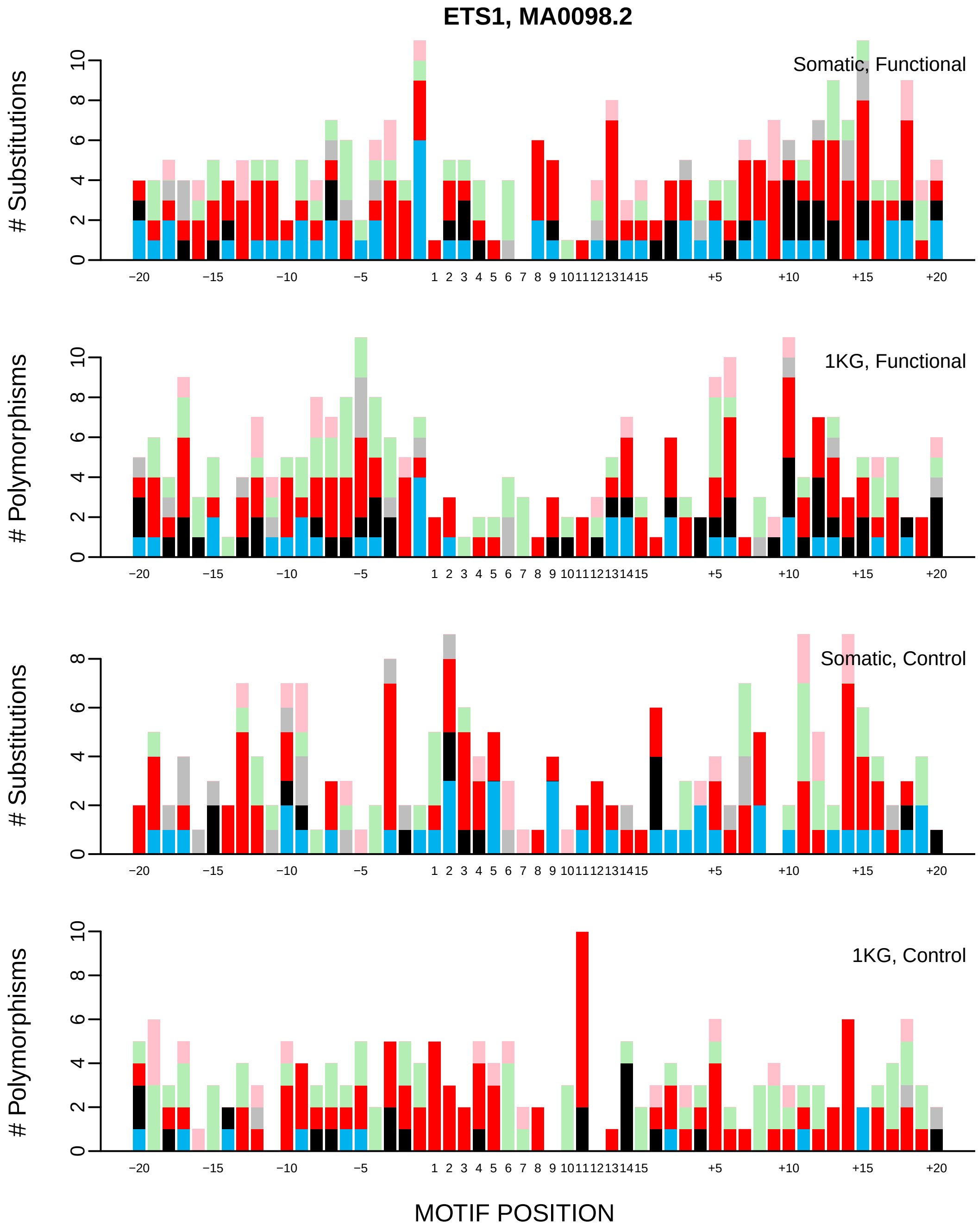

ETS1, MA0098.2
Somatic, Functional
1KG, Functional
Somatic, Control
1KG, Control
Substitutions
Polymorphisms
0
2
4
6
8
10
−20
−15
−10
−5
1 2 3 4 5 6 7 8 9 10 11 12 13 14 15
+5
+10
+15
+20
MOTIF POSITION

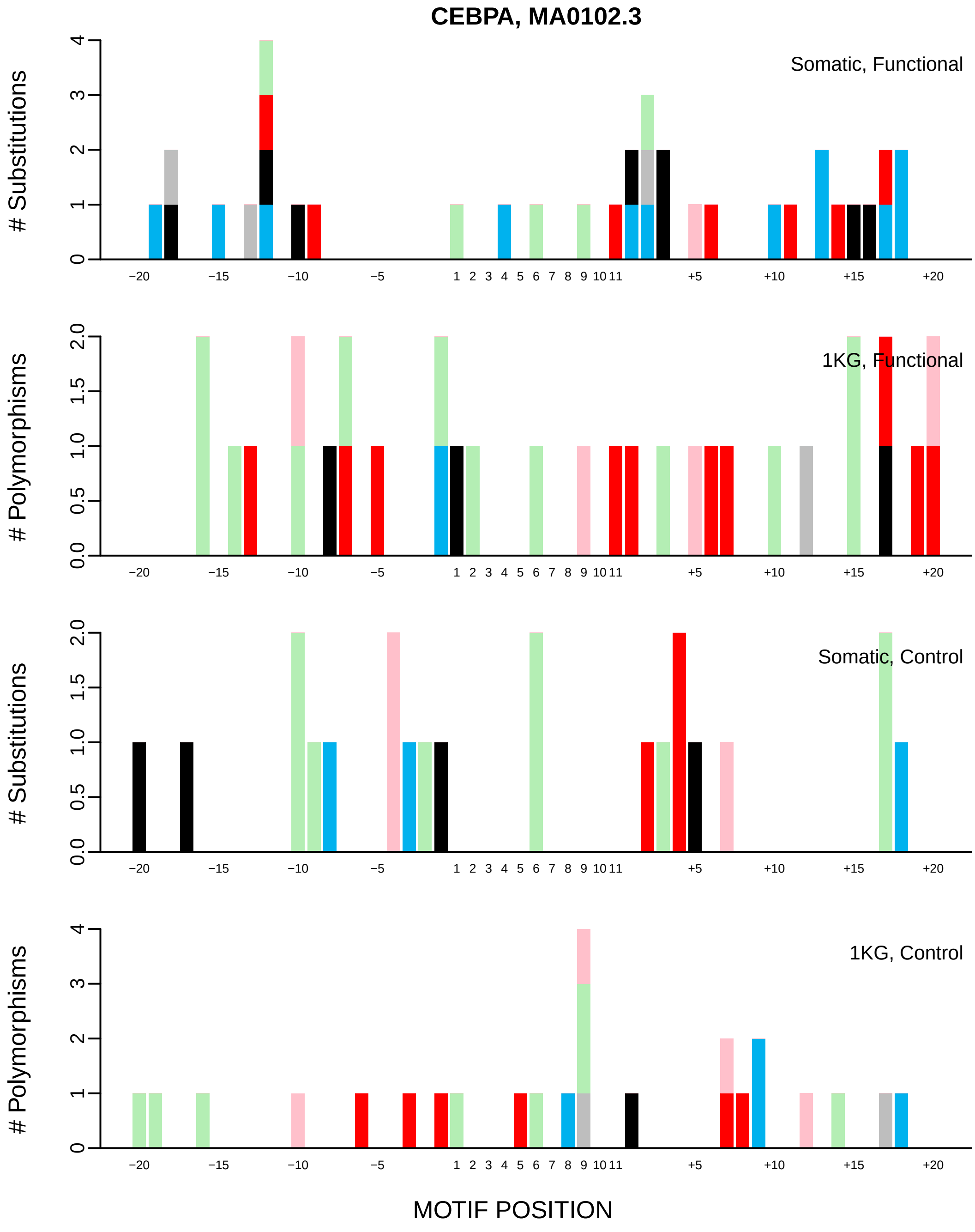
CEBPA, MA0102.3
Substitutions
Somatic, Functional
Polymorphisms
1KG, Functional
Substitutions
Somatic, Control
Polymorphisms
1KG, Control
−20 −15 −10 −5 1 2 3 4 5 6 7 8 9 10 11 +5 +10 +15 +20
MOTIF POSITION

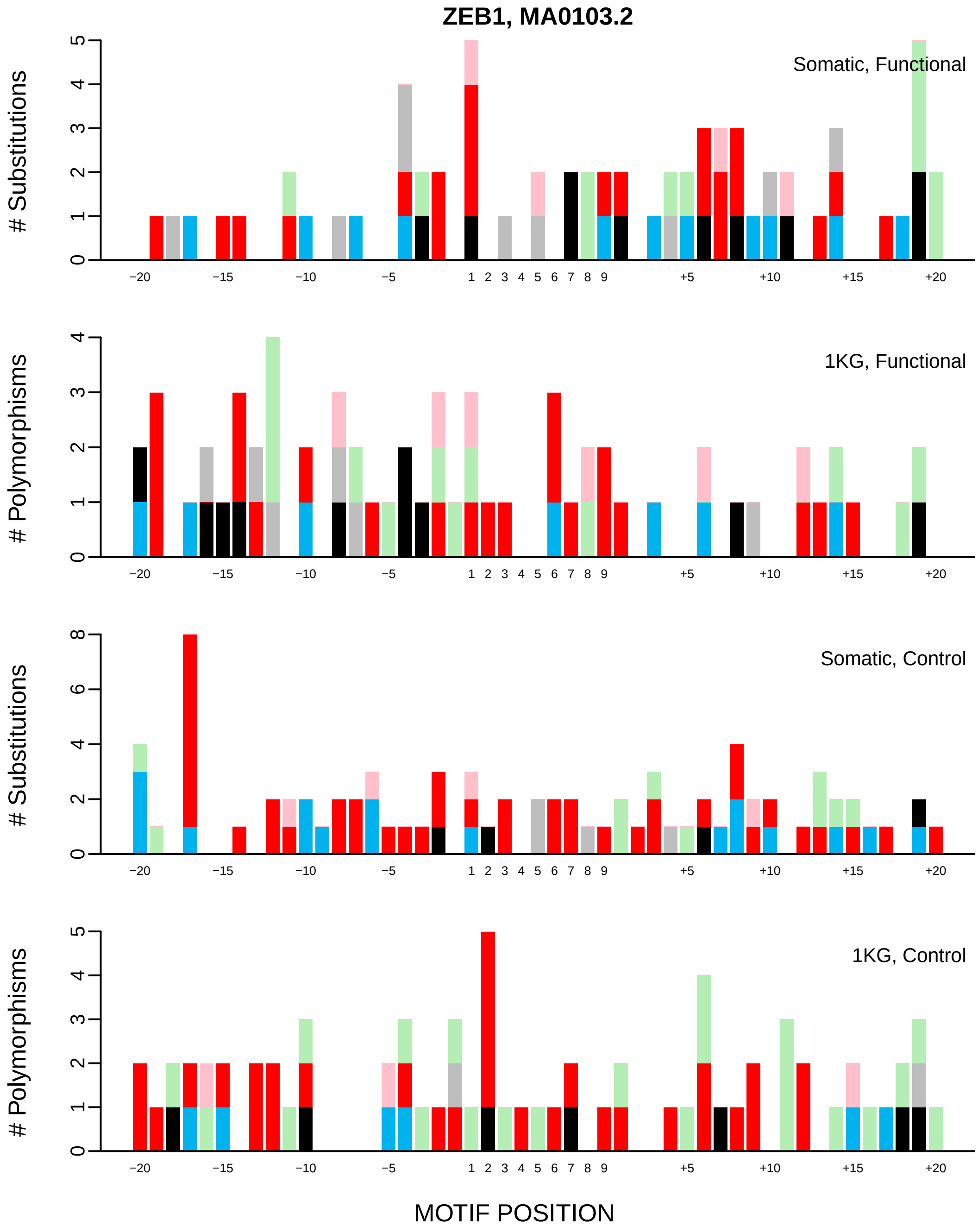
ZEB1, MA0103.2
Substitutions
Somatic, Functional
Polymorphisms
1KG, Functional
Substitutions
Somatic, Control
Polymorphisms
1KG, Control
−20
−15
−10
−5
1 2 3 4 5 6 7 8 9
+5
+10
+15
+20
MOTIF POSITION

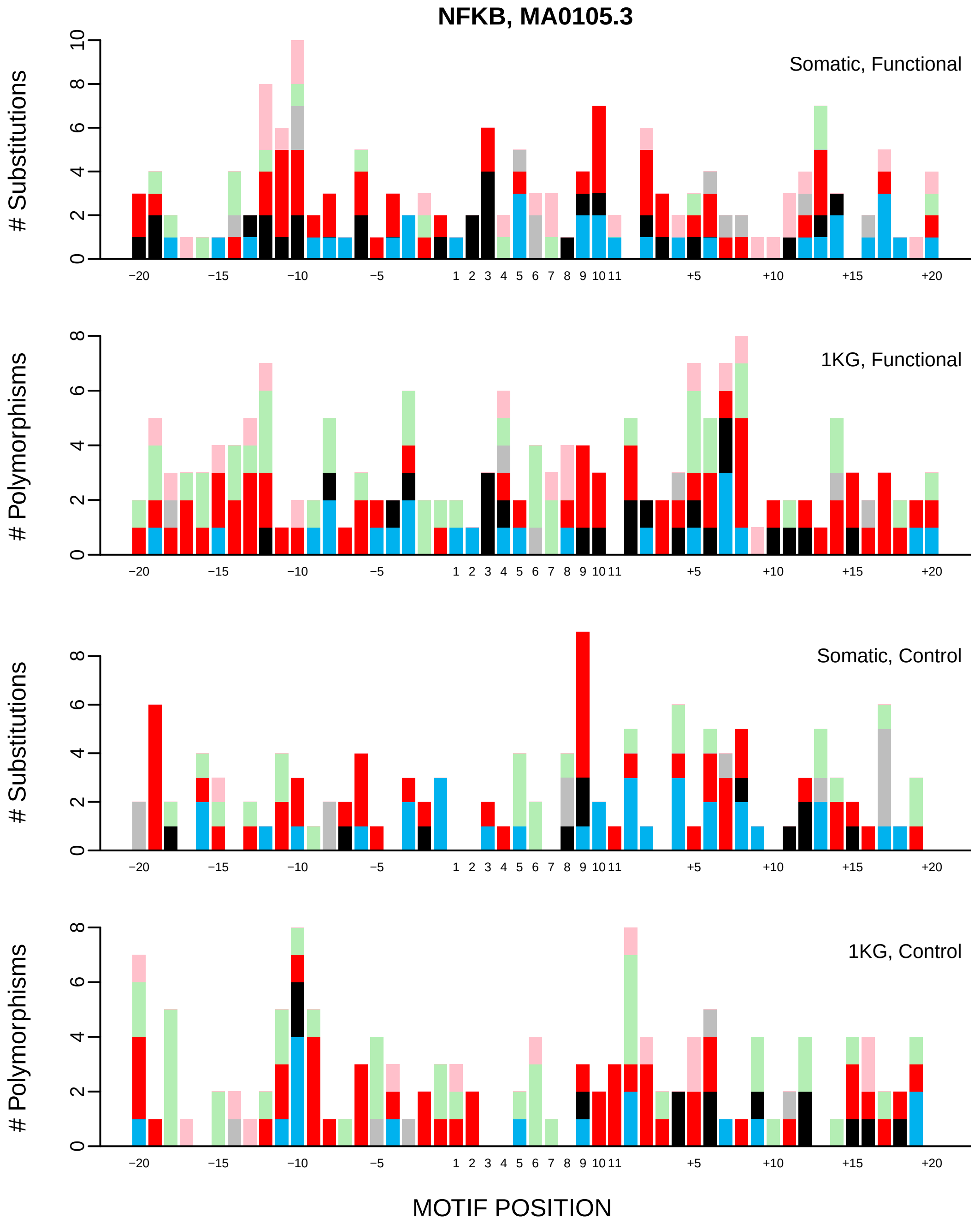
NFKB, MA0105.3
Somatic, Functional
1KG, Functional
Somatic, Control
1KG, Control
Substitutions
Polymorphisms
−20
−15
−10
−5
1 2 3 4 5 6 7 8 9 10 11
+5
+10
+15
+20
MOTIF POSITION

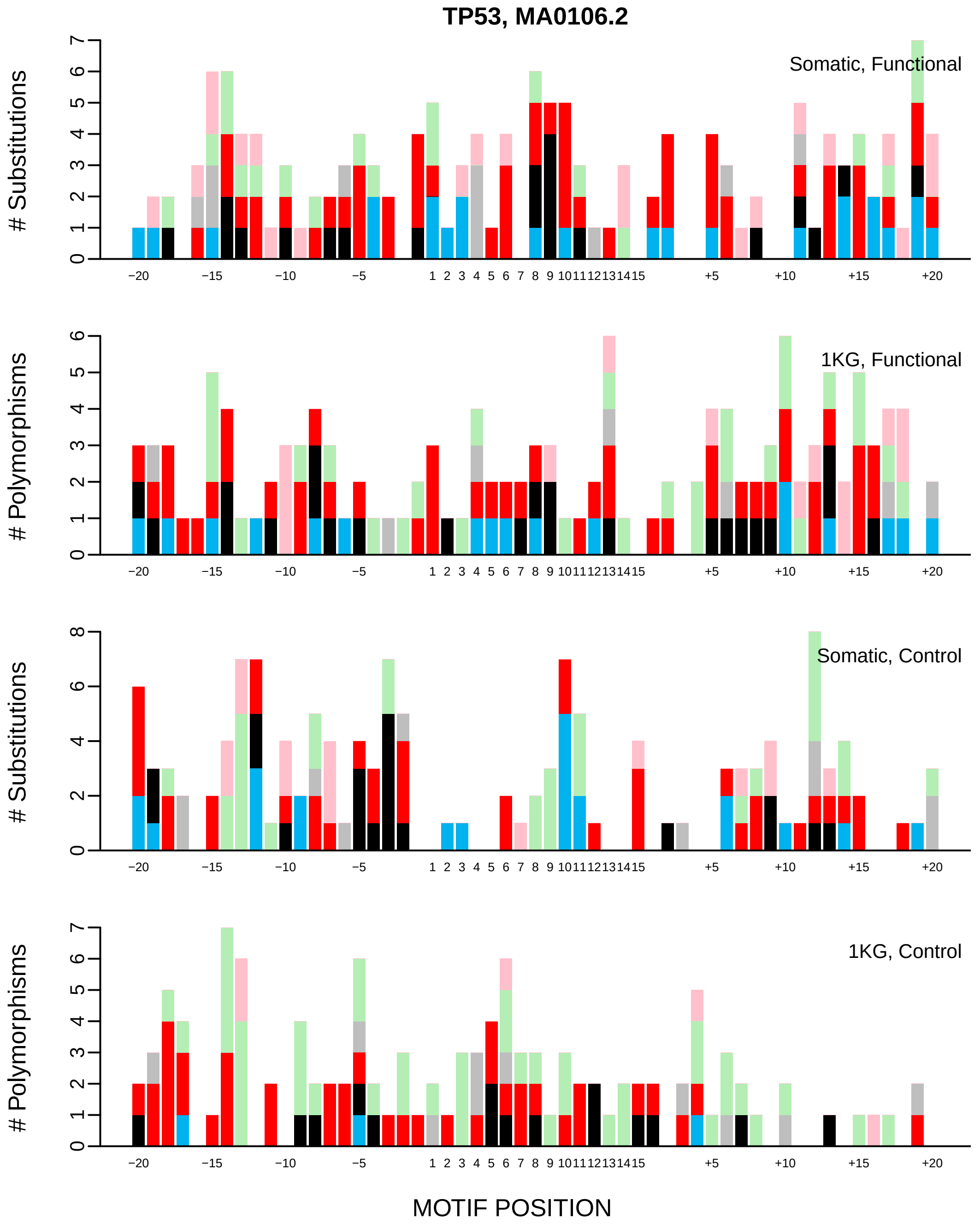
TP53, MA0106.2
Substitutions
Somatic, Functional
Polymorphisms
1KG, Functional
Substitutions
Somatic, Control
Polymorphisms
1KG, Control
−20 −15 −10 −5 1 2 3 4 5 6 7 8 9 10 11 12 13 14 15 +5 +10 +15 +20
MOTIF POSITION

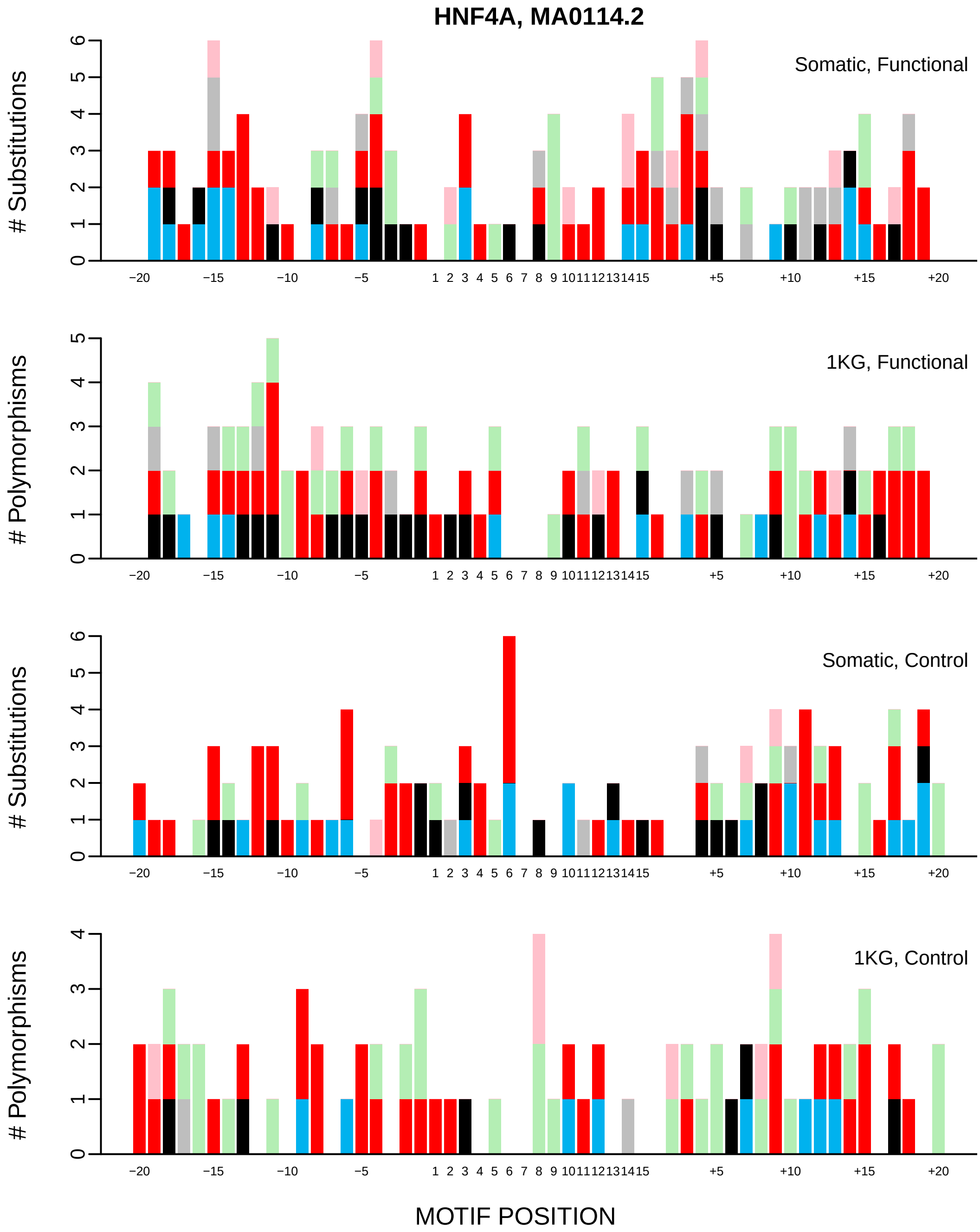

HNF4A, MA0114.2
Substitutions
Somatic, Functional
Polymorphisms
1KG, Functional
Substitutions
Somatic, Control
Polymorphisms
1KG, Control
−20 −15 −10 −5 1 2 3 4 5 6 7 8 9 10 11 12 13 14 15 +5 +10 +15 +20
MOTIF POSITION

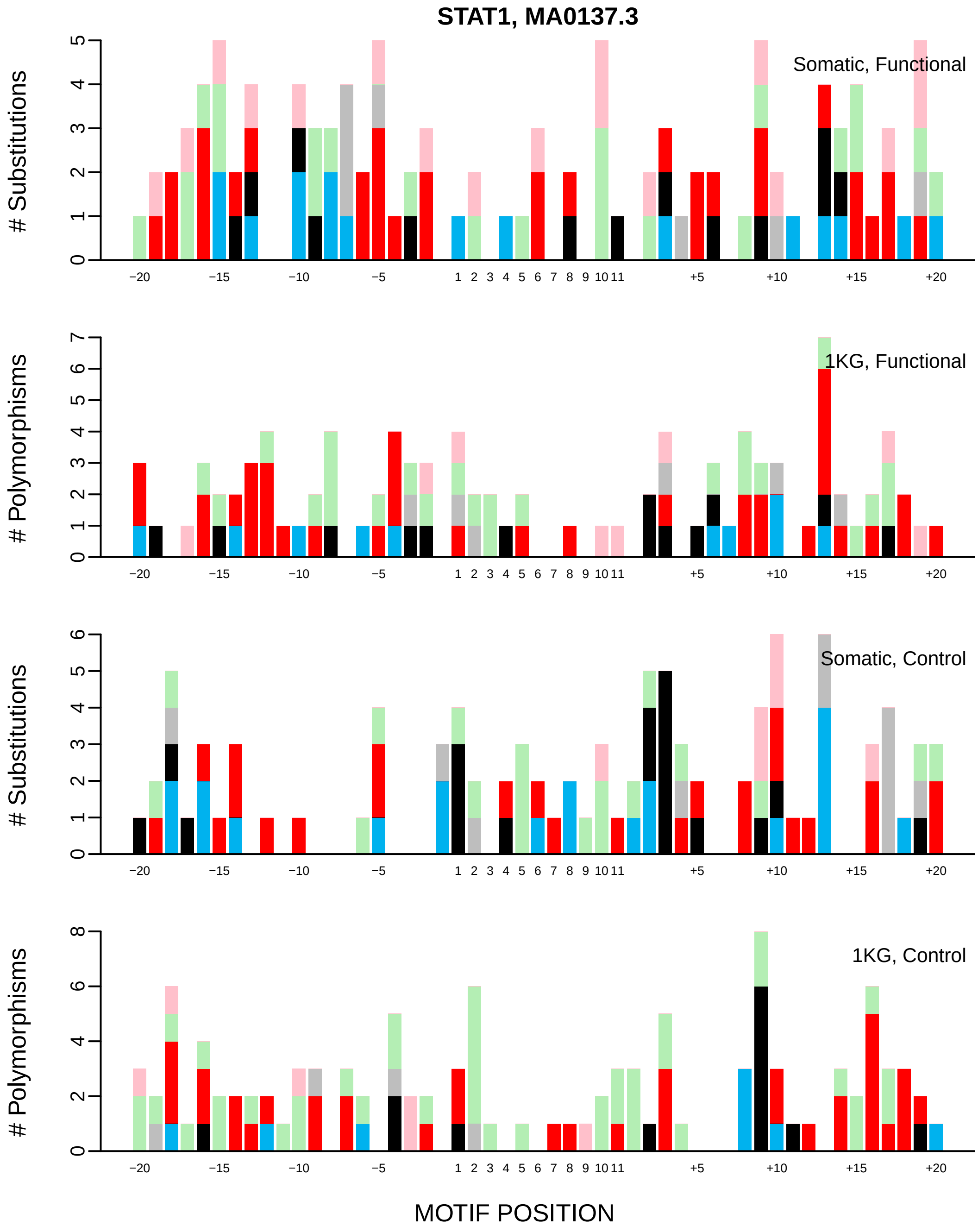
STAT1, MA0137.3
Substitutions
Somatic, Functional
Polymorphisms
1KG, Functional
Substitutions
Somatic, Control
Polymorphisms
1KG, Control
−20 −15 −10 −5 1 2 3 4 5 6 7 8 9 10 11 +5 +10 +15 +20
MOTIF POSITION

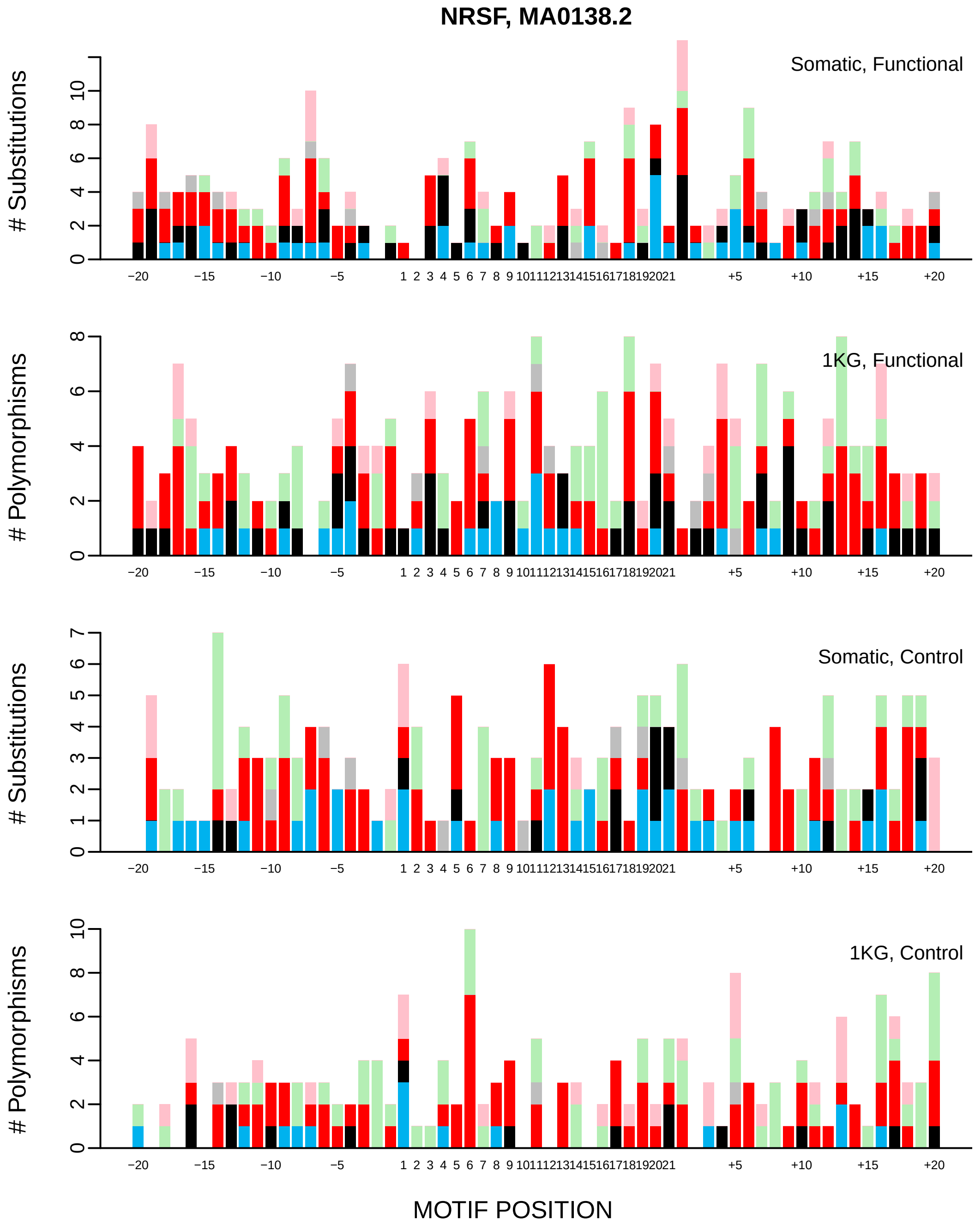
NRSF, MA0138.2
Somatic, Functional
Substitutions
1KG, Functional
Polymorphisms
Somatic, Control
Substitutions
1KG, Control
Polymorphisms
−20 −15 −10 −5 1 2 3 4 5 6 7 8 9 10 11 12 13 14 15 16 17 18 19 20 21 +5 +10 +15 +20
MOTIF POSITION

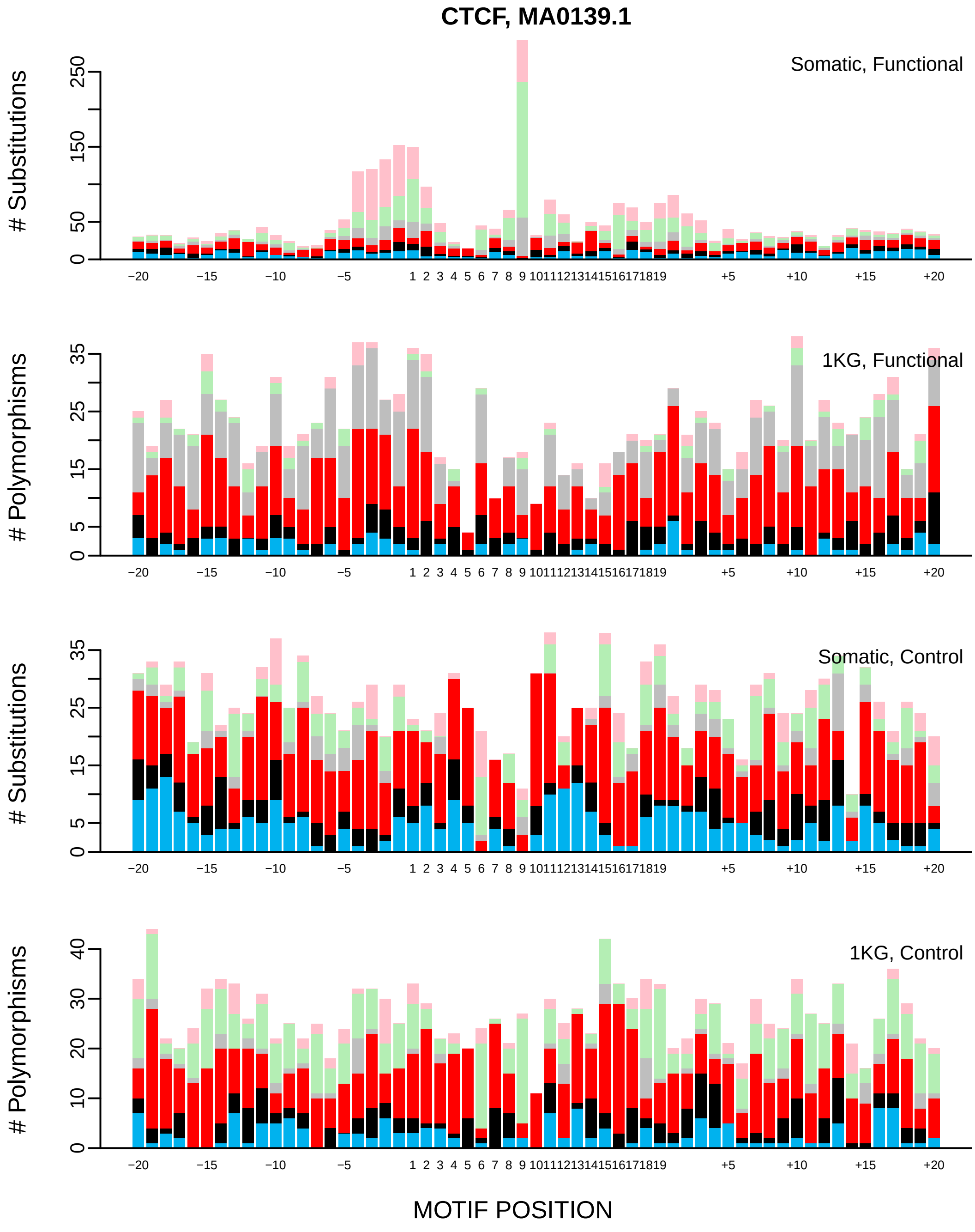
CTCF, MA0139.1
Somatic, Functional
1KG, Functional
Somatic, Control
1KG, Control
Substitutions
Polymorphisms
0
5
15
25
35
50
150
250
10
20
30
40
−20
−15
−10
−5
1 2 3 4 5 6 7 8 9 10 11 12 13 14 15 16 17 18 19
+5
+10
+15
+20
MOTIF POSITION

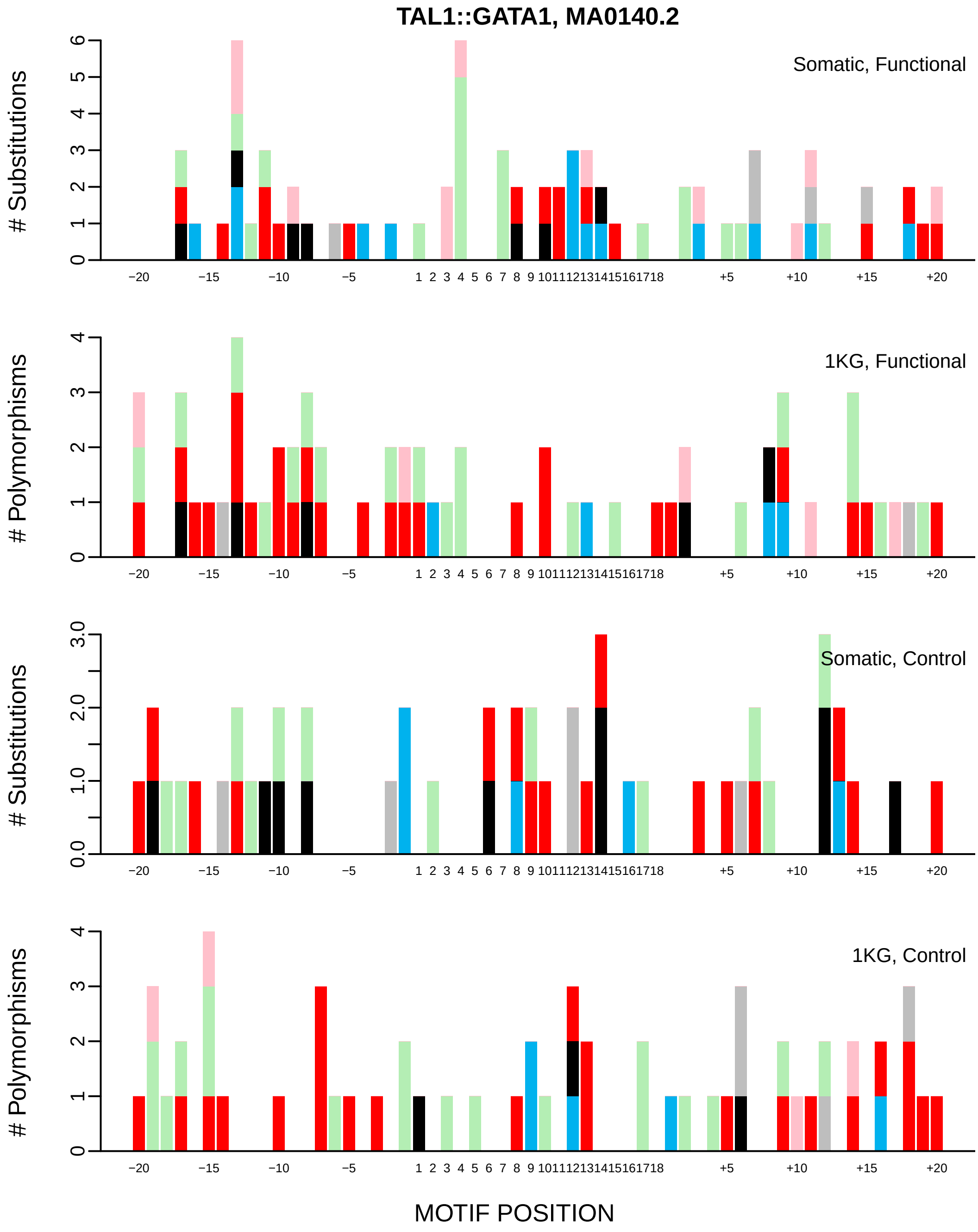

TAL1::GATA1, MA0140.2
Somatic, Functional
1KG, Functional
Somatic, Control
1KG, Control
Substitutions
Polymorphisms
−20 −15 −10 −5 1 2 3 4 5 6 7 8 9 101112131415161718 +5 +10 +15 +20
MOTIF POSITION

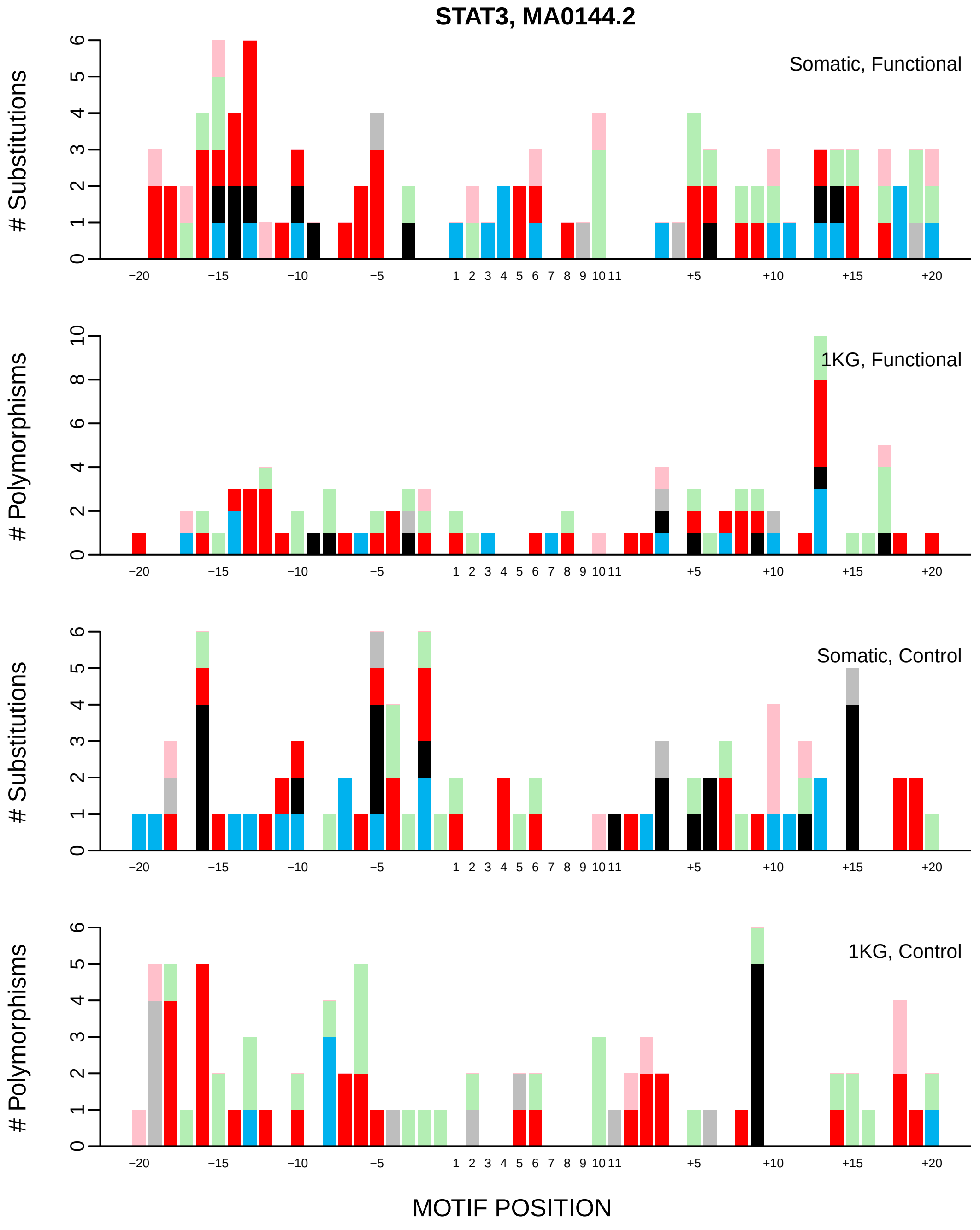
STAT3, MA0144.2
Substitutions
Somatic, Functional
Polymorphisms
1KG, Functional
Substitutions
Somatic, Control
Polymorphisms
1KG, Control
−20 −15 −10 −5 1 2 3 4 5 6 7 8 9 10 11 +5 +10 +15 +20
MOTIF POSITION

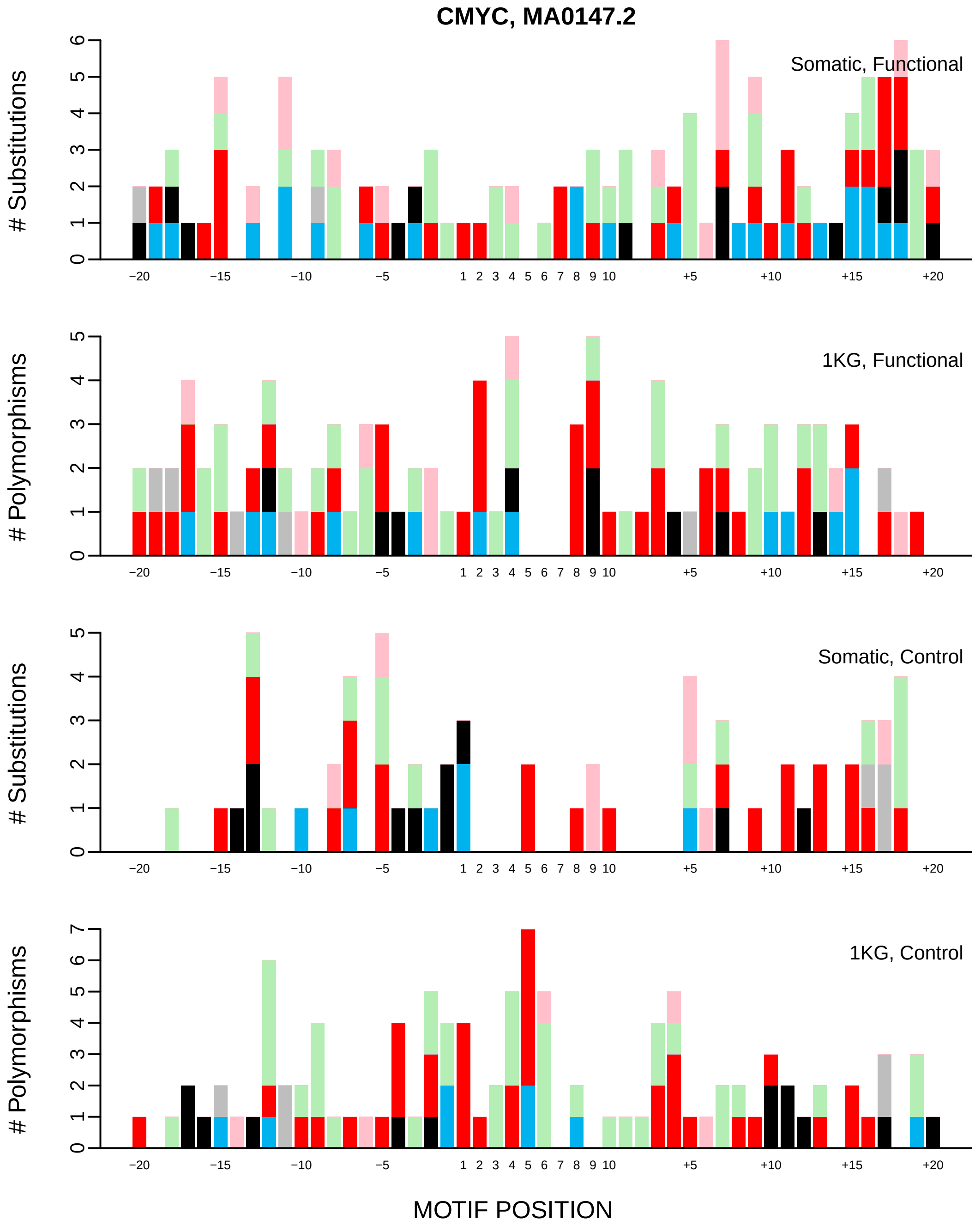
CMYC, MA0147.2
Somatic, Functional
1KG, Functional
Somatic, Control
1KG, Control
Substitutions
Polymorphisms
−20
−15
−10
−5
1 2 3 4 5 6 7 8 9 10
+5
+10
+15
+20
MOTIF POSITION

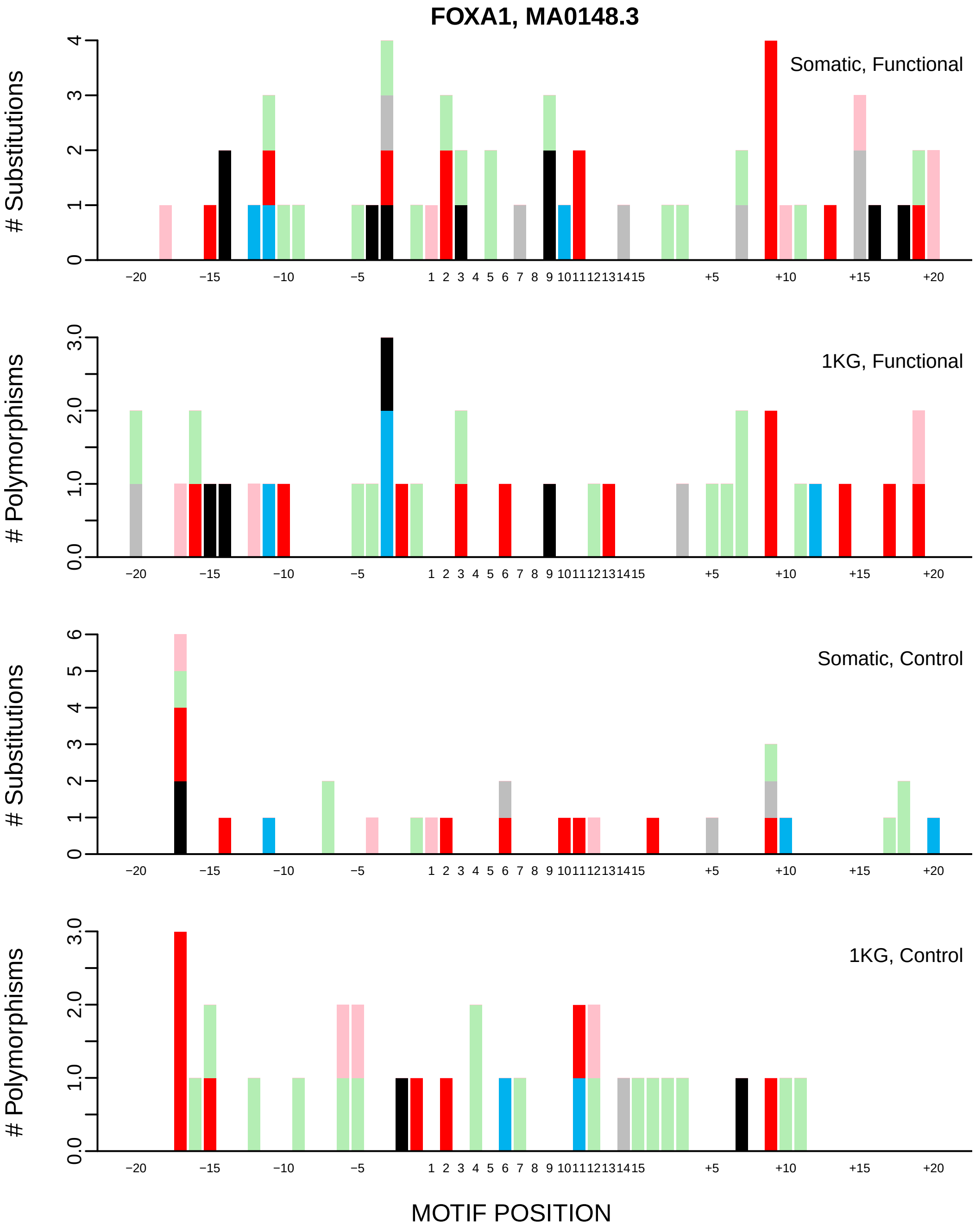
FOXA1, MA0148.3
Somatic, Functional
1KG, Functional
Somatic, Control
1KG, Control
Substitutions
Polymorphisms
−20
−15
−10
−5
1 2 3 4 5 6 7 8 9 10 11 12 13 14 15
+5
+10
+15
+20
MOTIF POSITION

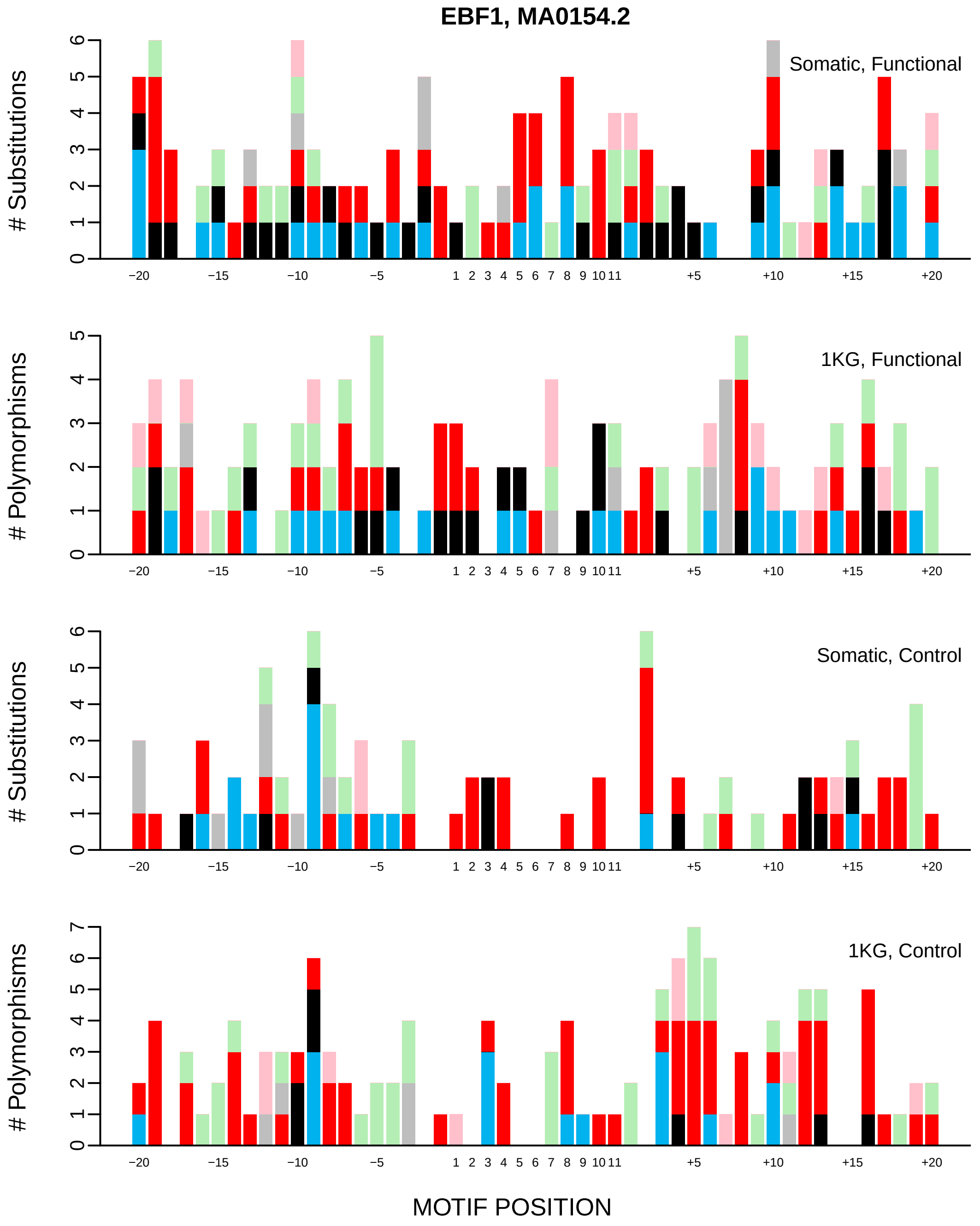
EBF1, MA0154.2
Substitutions
Somatic, Functional
Polymorphisms
1KG, Functional
Substitutions
Somatic, Control
Polymorphisms
1KG, Control
−20
−15
−10
−5
1 2 3 4 5 6 7 8 9 10 11
+5
+10
+15
+20
MOTIF POSITION

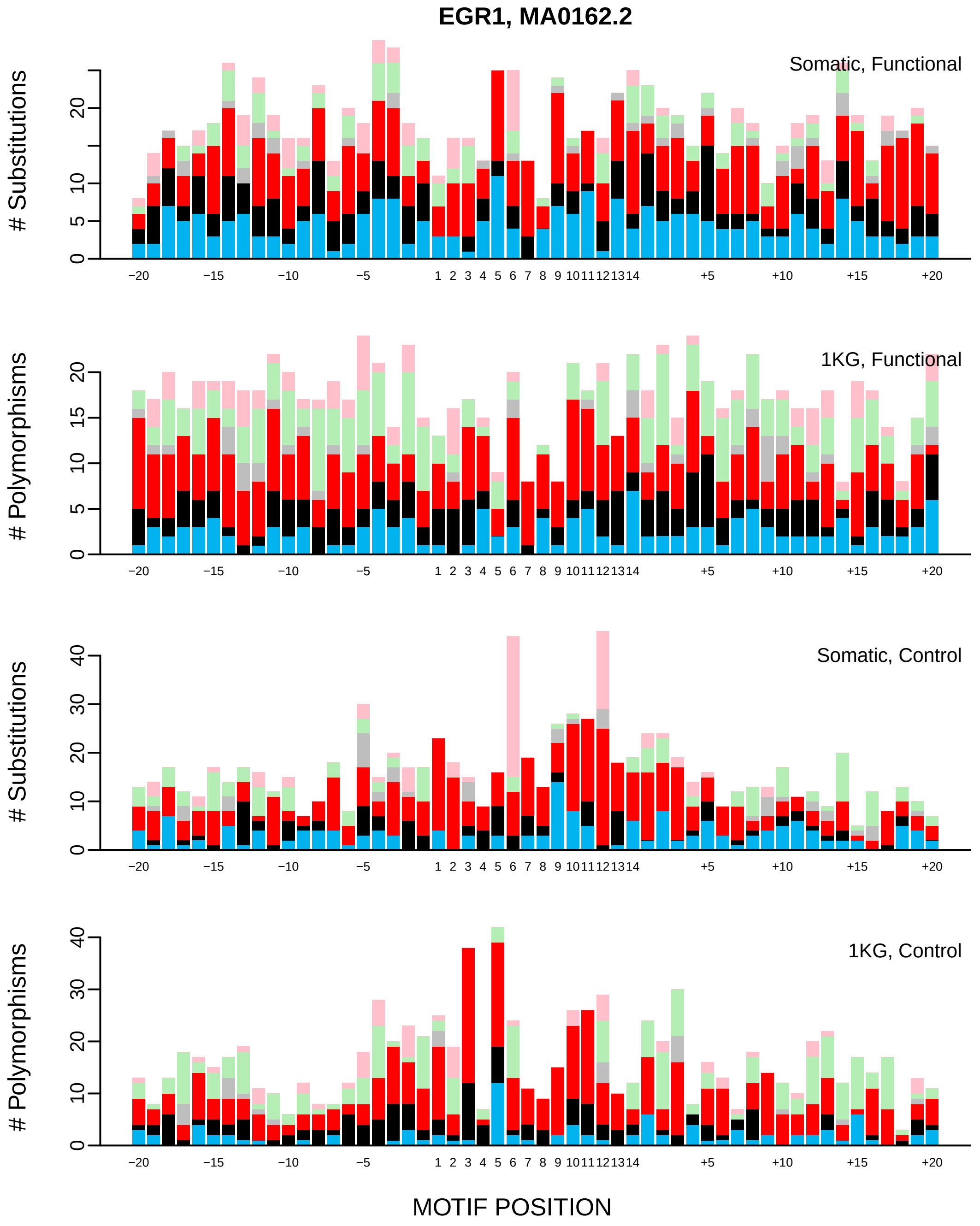
EGR1, MA0162.2
Somatic, Functional
1KG, Functional
Somatic, Control
1KG, Control
Substitutions
Polymorphisms
−20
−15
−10
−5
1 2 3 4 5 6 7 8 9 10 11 12 13 14
+5
+10
+15
+20
MOTIF POSITION

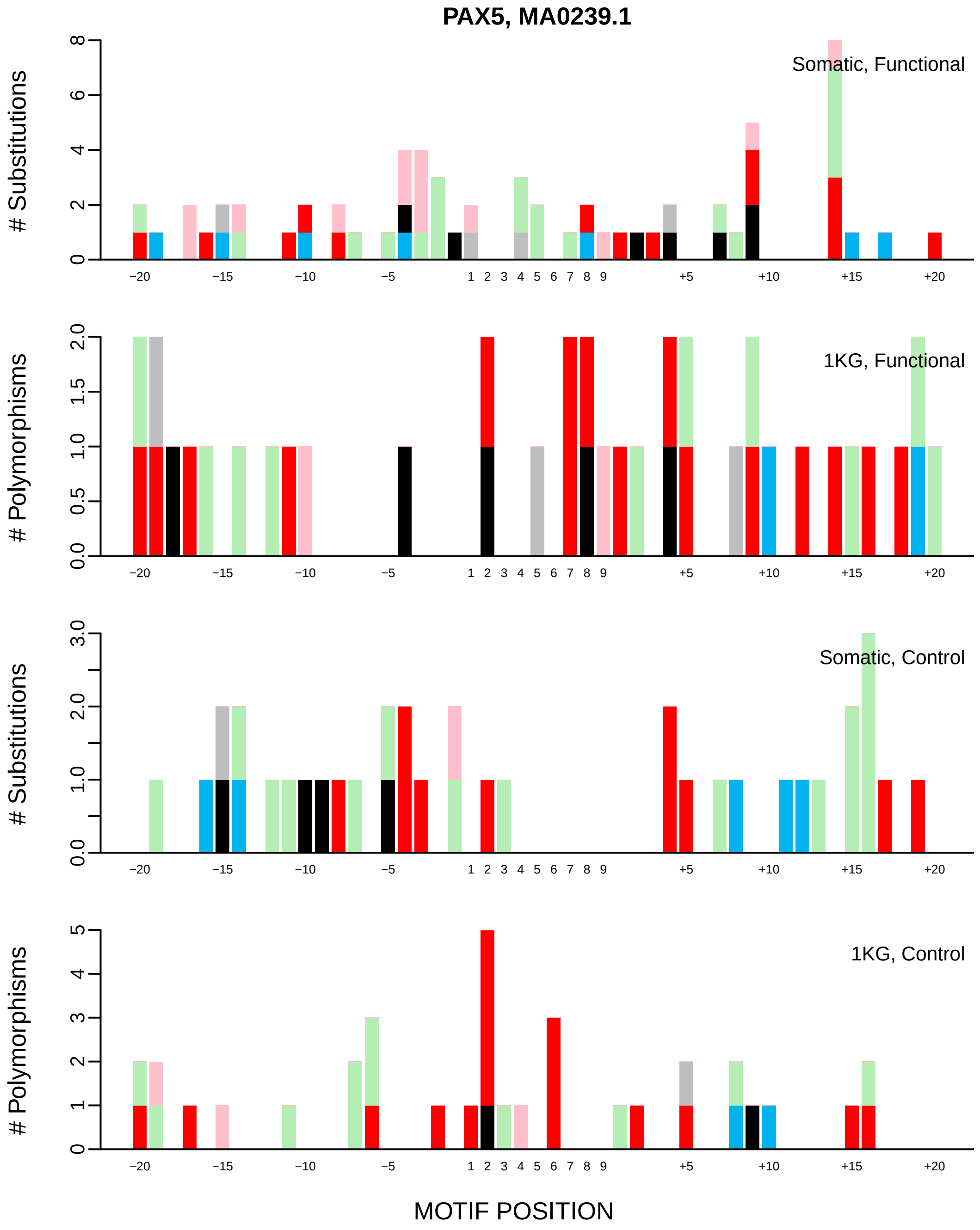
PAX5, MA0239.1
Substitutions
Somatic, Functional
Polymorphisms
1KG, Functional
Substitutions
Somatic, Control
Polymorphisms
1KG, Control
−20 −15 −10 −5 1 2 3 4 5 6 7 8 9 +5 +10 +15 +20
MOTIF POSITION

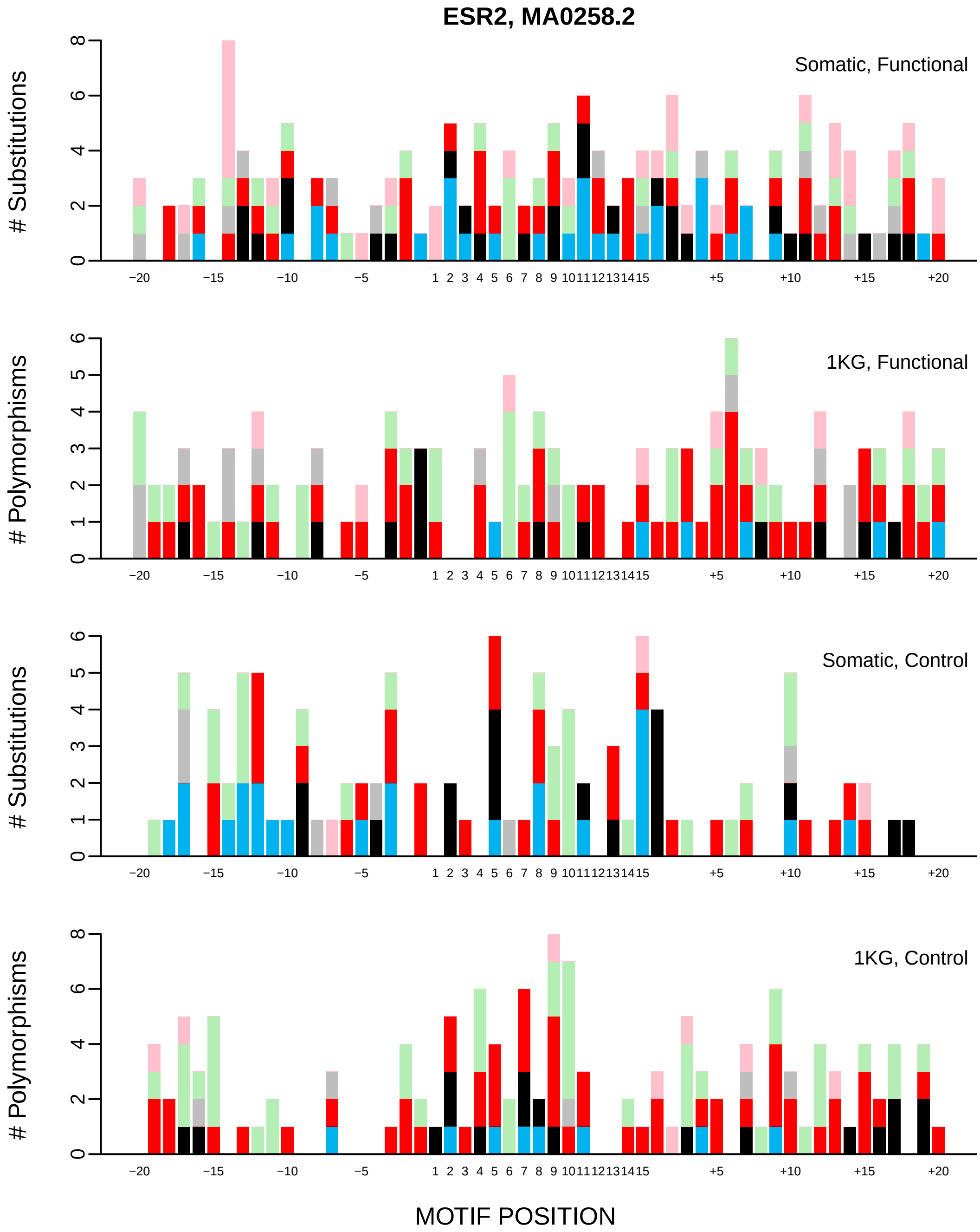

ESR2, MA0258.2
Somatic, Functional
1KG, Functional
Somatic, Control
1KG, Control
Substitutions
Polymorphisms
−20 −15 −10 −5 1 2 3 4 5 6 7 8 9 10 11 12 13 14 15 +5 +10 +15 +20
MOTIF POSITION

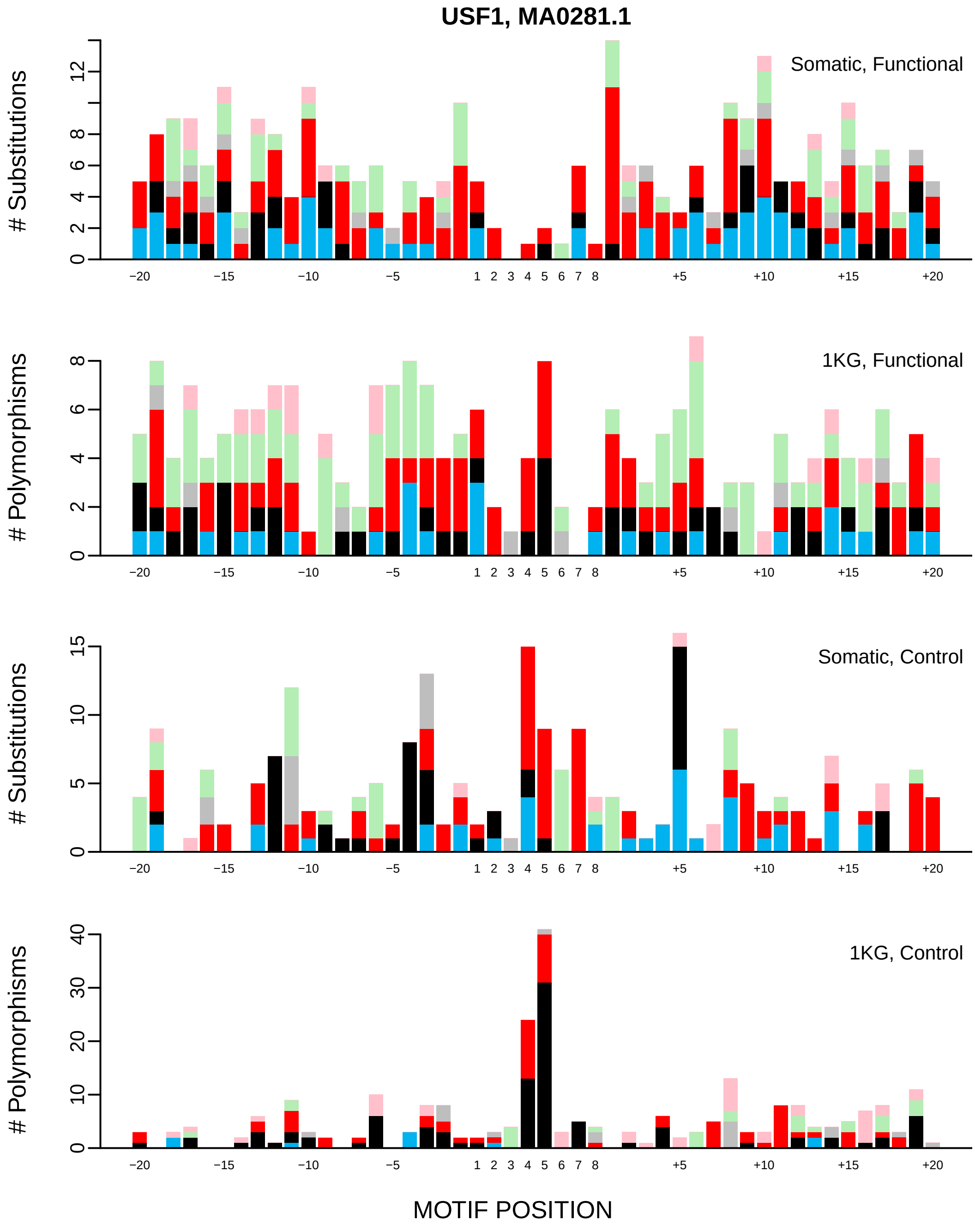
USF1, MA0281.1
Substitutions
Somatic, Functional
Polymorphisms
1KG, Functional
Substitutions
Somatic, Control
Polymorphisms
1KG, Control
−20
−15
−10
−5
1 2 3 4 5 6 7 8
+5
+10
+15
+20
MOTIF POSITION

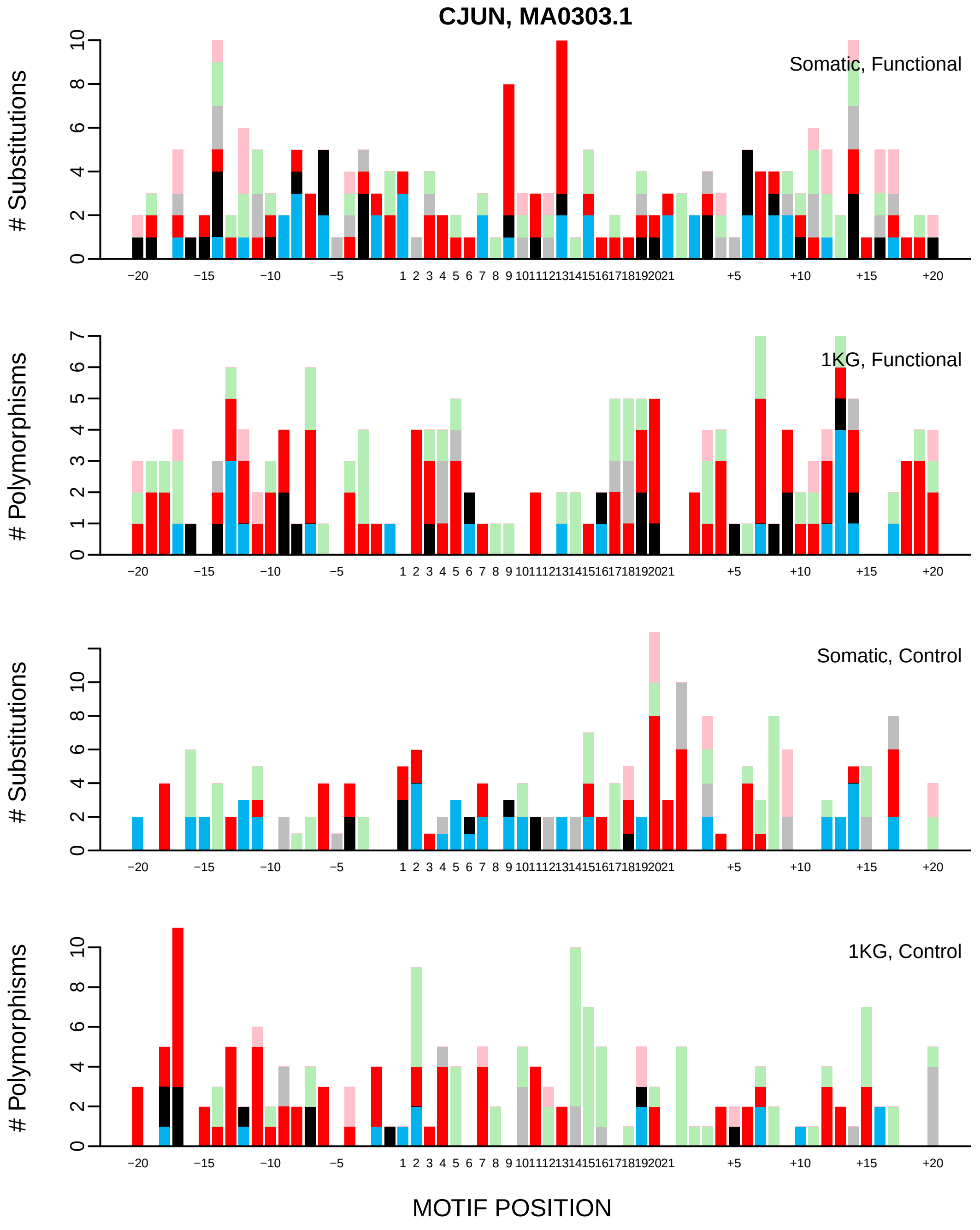

CJUN, MA0303.1
Substitutions
Somatic, Functional
Polymorphisms
1KG, Functional
Substitutions
Somatic, Control
Polymorphisms
1KG, Control
−20 −15 −10 −5 1 2 3 4 5 6 7 8 9 10 11 12 13 14 15 16 17 18 19 20 21 +5 +10 +15 +20
MOTIF POSITION

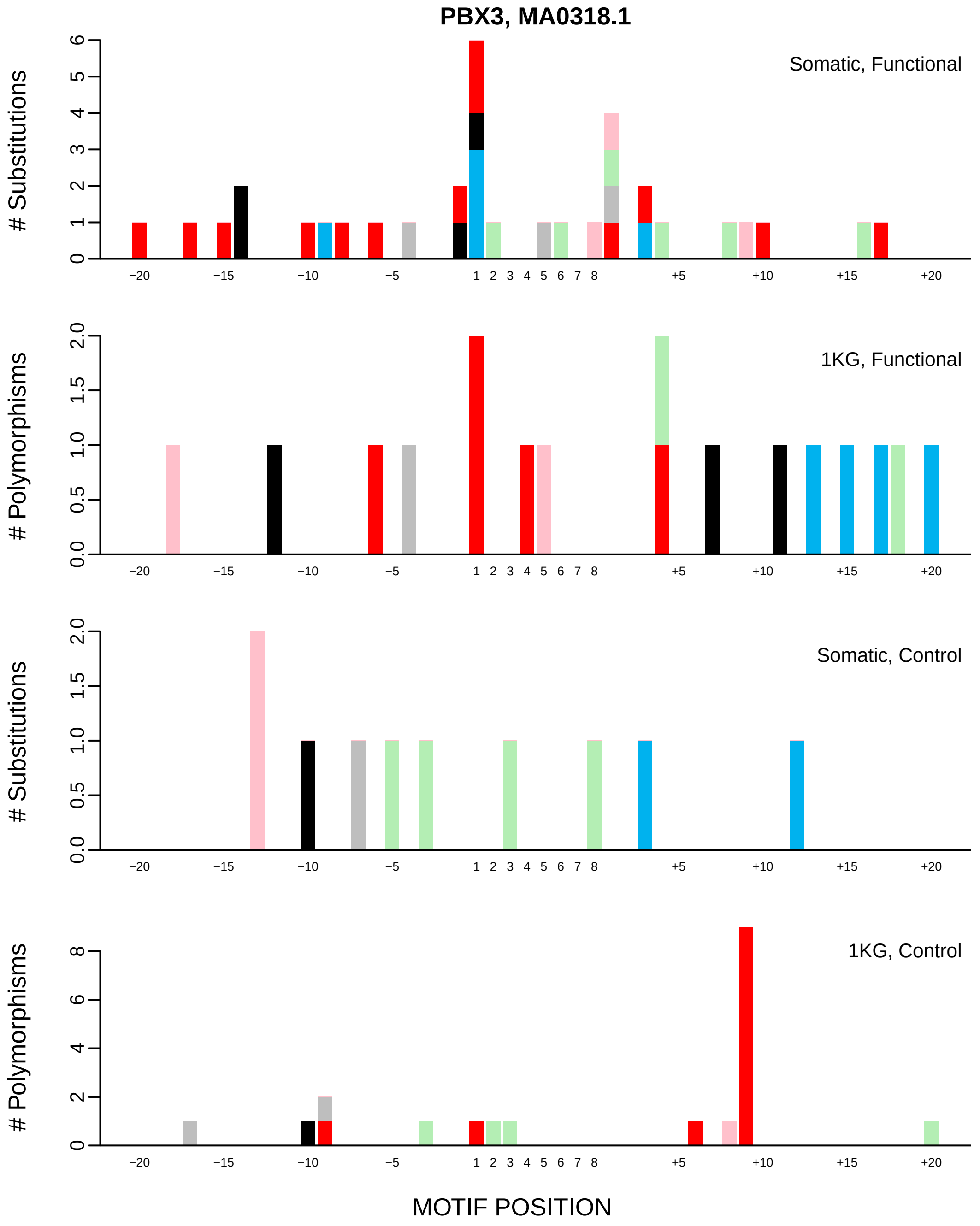

PBX3, MA0318.1
Somatic, Functional
1KG, Functional
Somatic, Control
1KG, Control
Substitutions
Polymorphisms
−20
−15
−10
−5
1 2 3 4 5 6 7 8
+5
+10
+15
+20
MOTIF POSITION

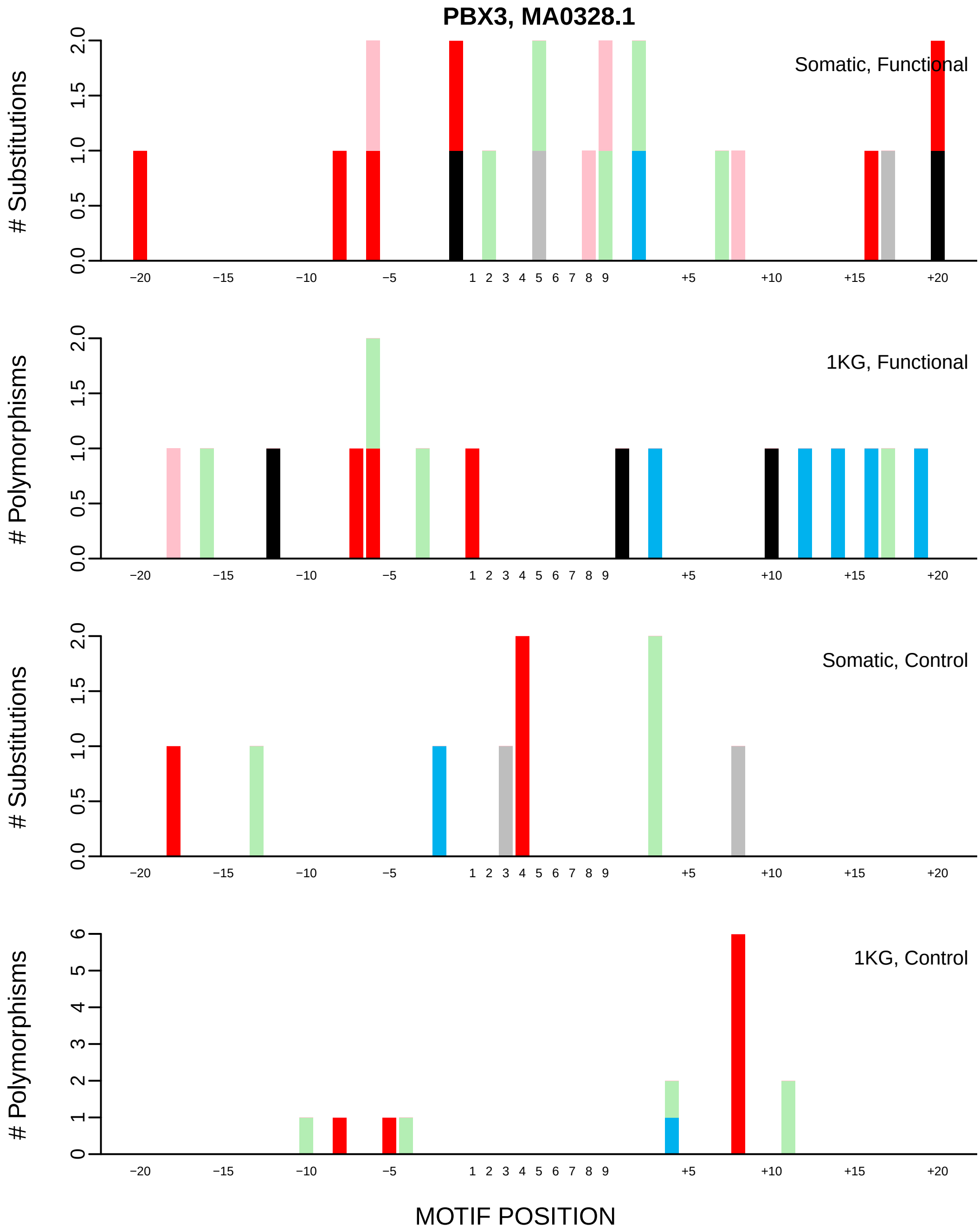
PBX3, MA0328.1
Substitutions
Somatic, Functional
Polymorphisms
1KG, Functional
Substitutions
Somatic, Control
Polymorphisms
1KG, Control
−20
−15
−10
−5
1 2 3 4 5 6 7 8 9
+5
+10
+15
+20
MOTIF POSITION

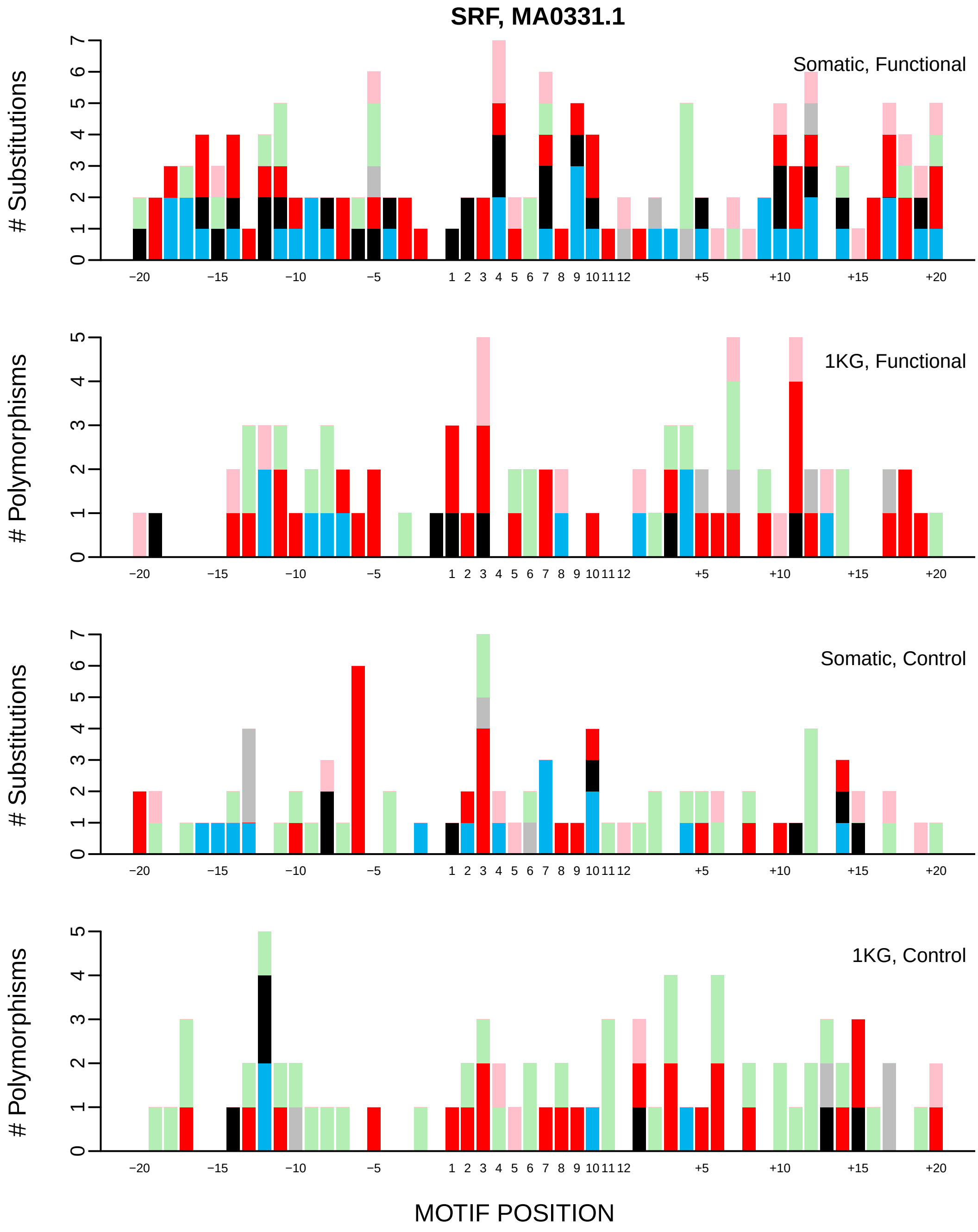
SRF, MA0331.1
Somatic, Functional
1KG, Functional
Somatic, Control
1KG, Control
Substitutions
Polymorphisms
MOTIF POSITION

EGR1, MA0337.1

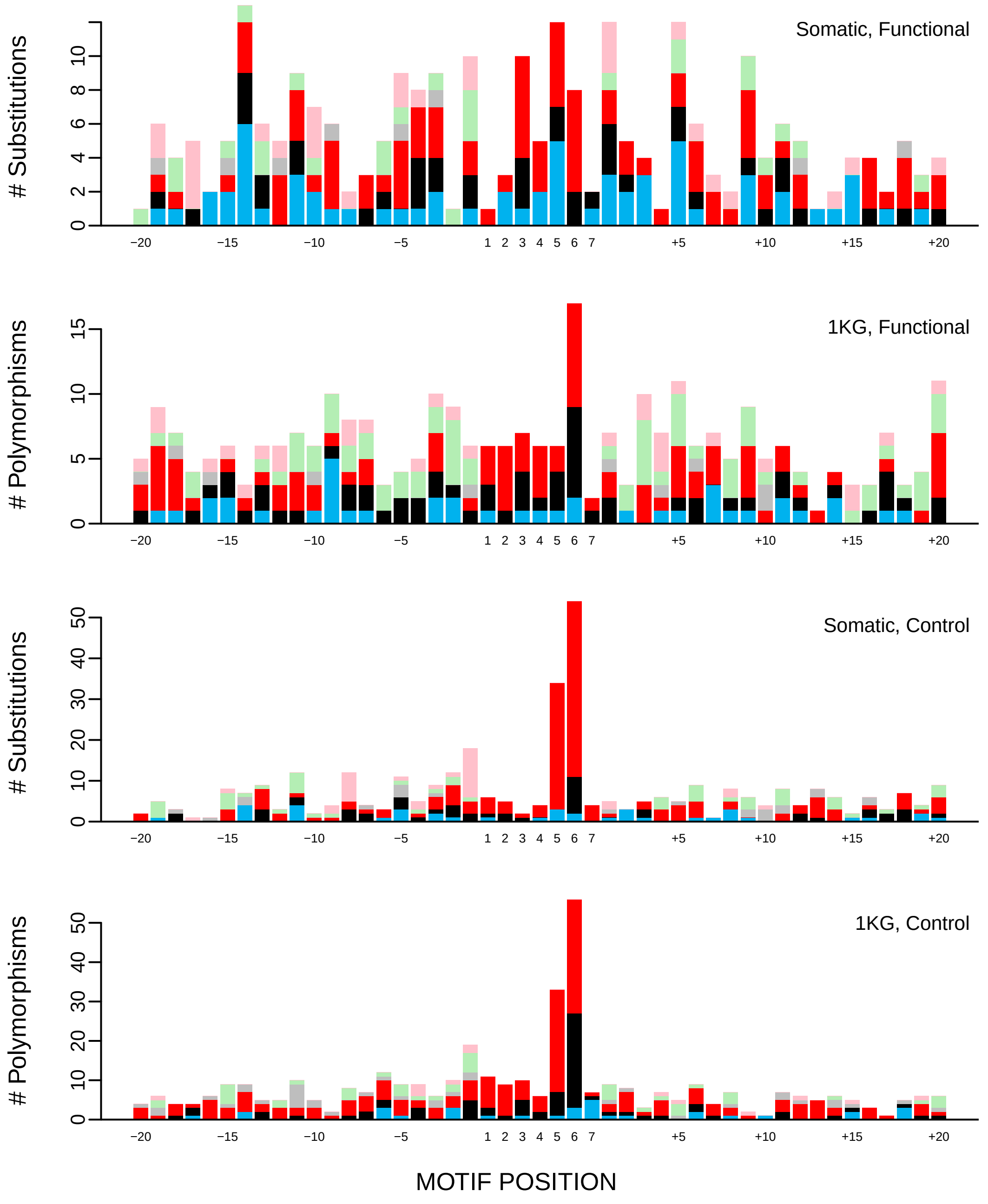

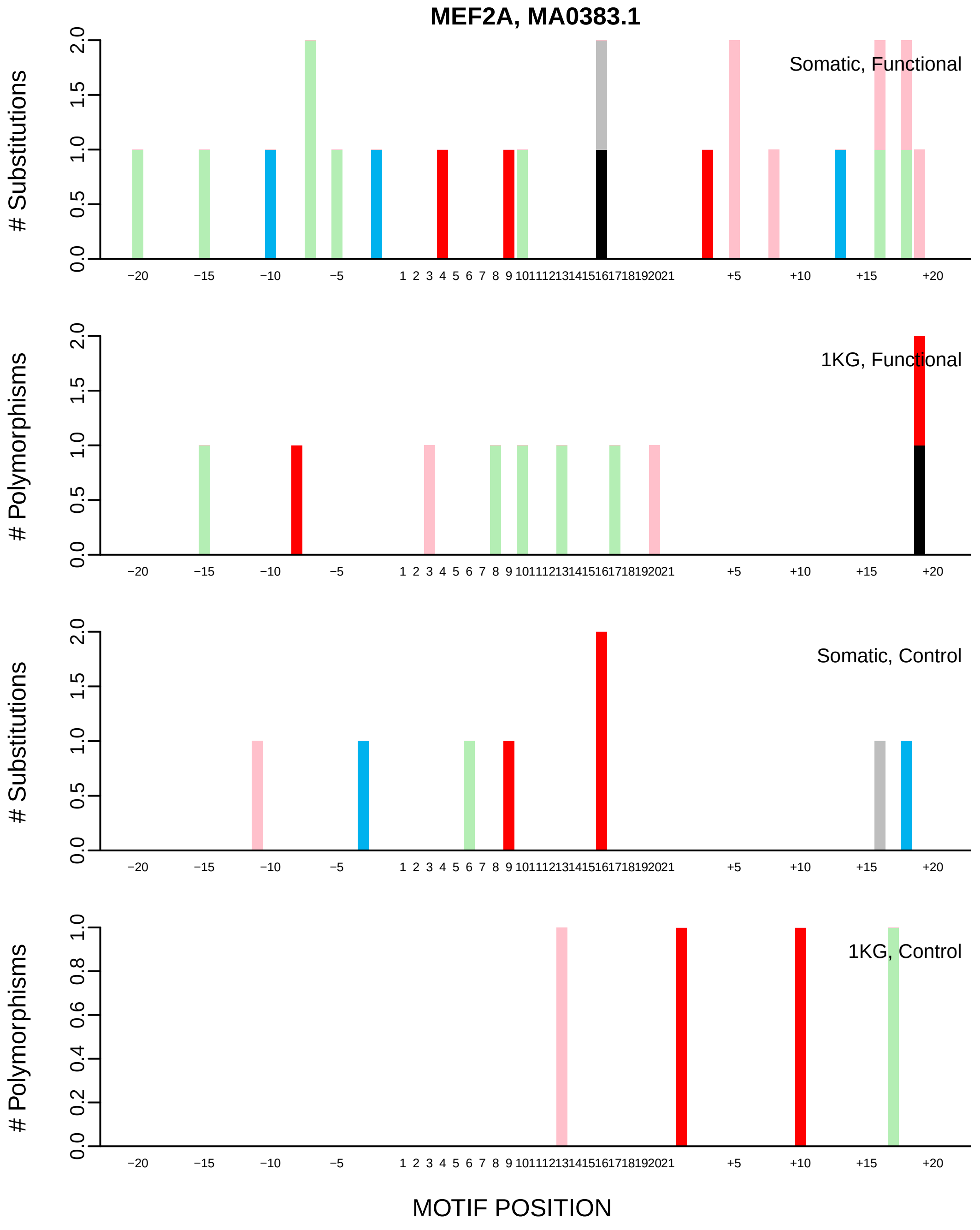

MEF2A, MA0383.1
Substitutions
Somatic, Functional
Polymorphisms
1KG, Functional
Substitutions
Somatic, Control
Polymorphisms
1KG, Control
−20 −15 −10 −5 1 2 3 4 5 6 7 8 9 10 11 12 13 14 15 16 17 18 19 20 21 +5 +10 +15 +20
MOTIF POSITION

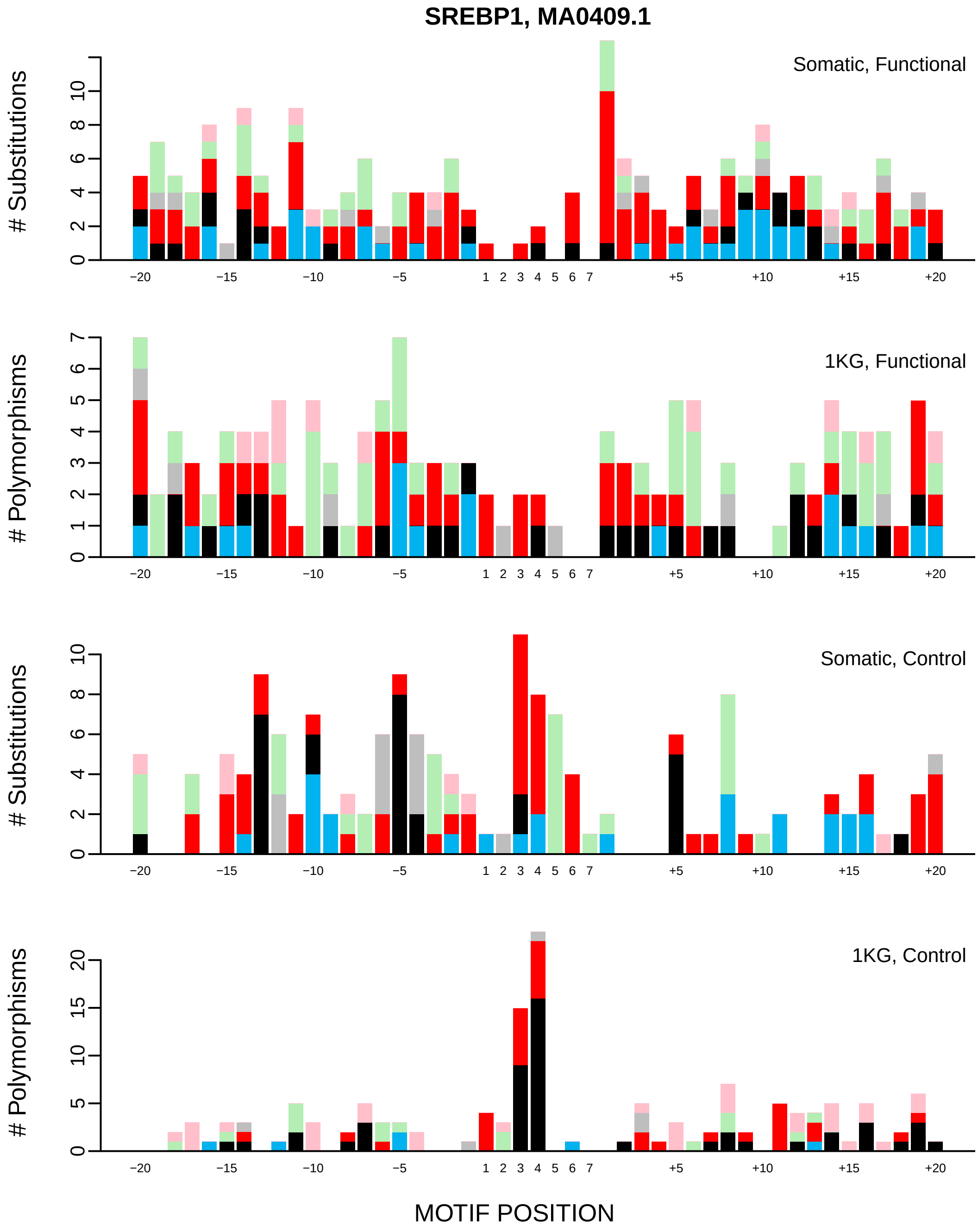

SREBP1, MA0409.1
Substitutions
Somatic, Functional
Polymorphisms
1KG, Functional
Substitutions
Somatic, Control
Polymorphisms
1KG, Control
−20
−15
−10
−5
1 2 3 4 5 6 7
+5
+10
+15
+20
MOTIF POSITION

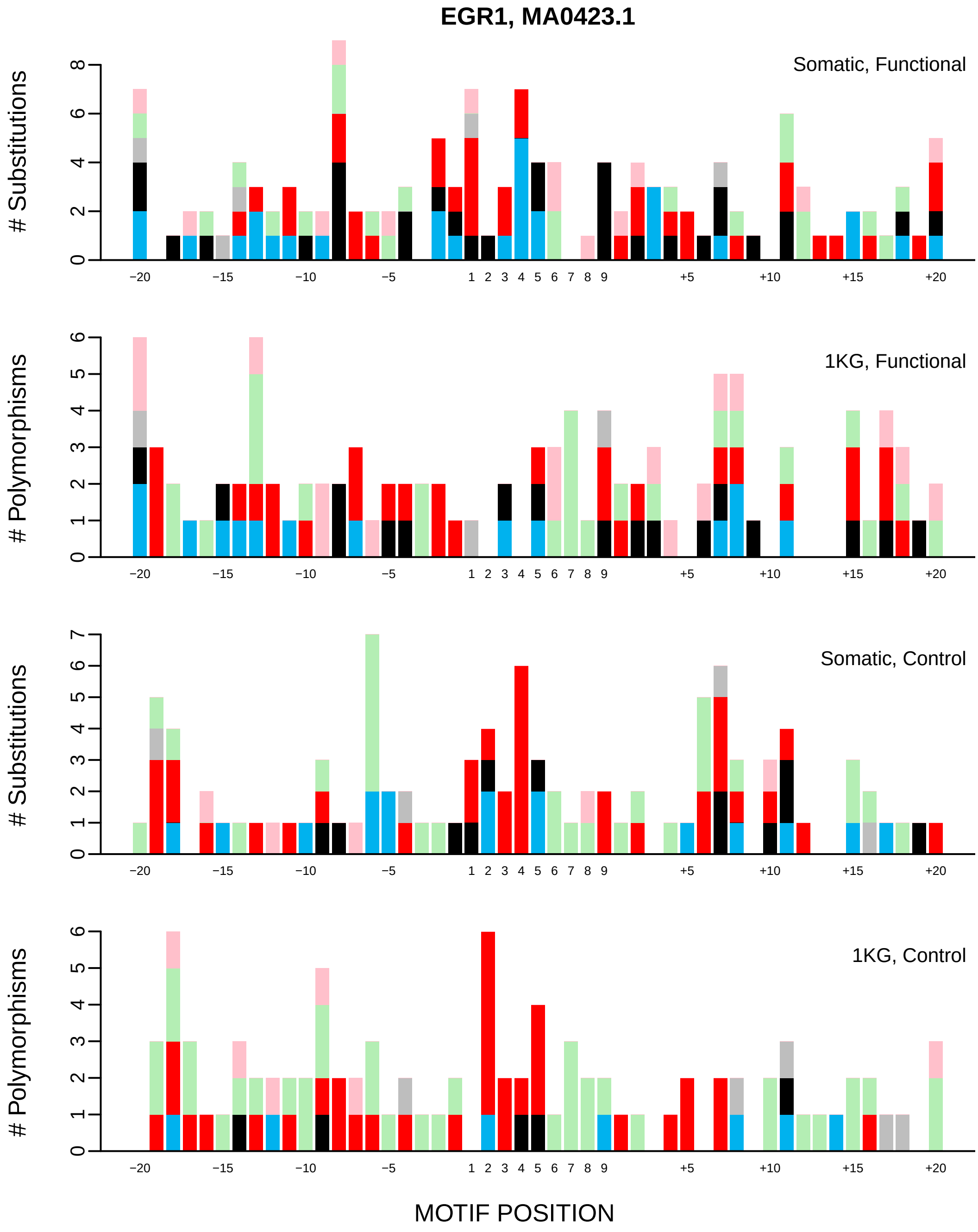

EGR1, MA0423.1
Substitutions
Somatic, Functional
Polymorphisms
1KG, Functional
Substitutions
Somatic, Control
Polymorphisms
1KG, Control
−20
−15
−10
−5
1 2 3 4 5 6 7 8 9
+5
+10
+15
+20
MOTIF POSITION

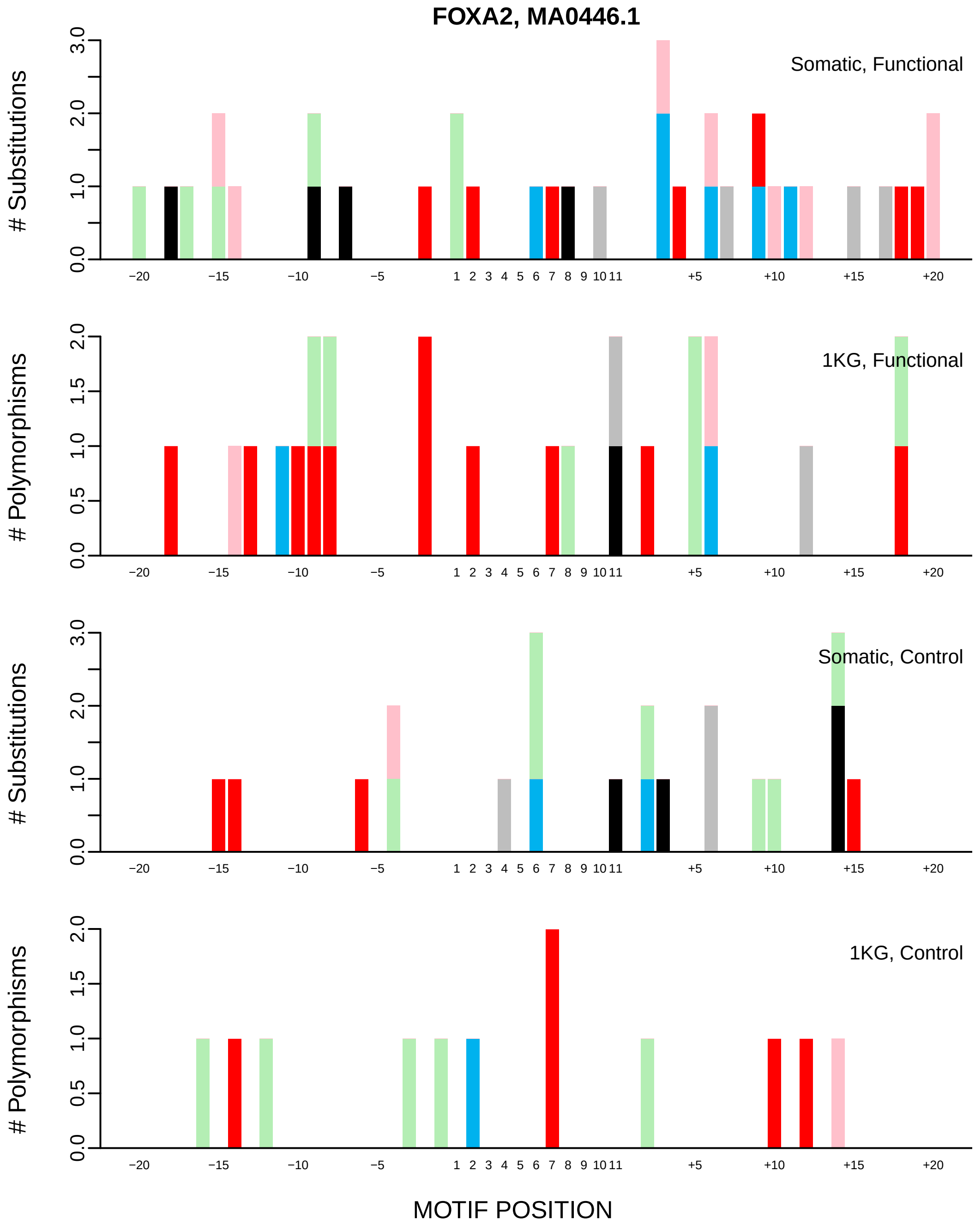

FOXA2, MA0446.1
Substitutions
Somatic, Functional
Polymorphisms
1KG, Functional
Substitutions
Somatic, Control
Polymorphisms
1KG, Control
−20 −15 −10 −5 1 2 3 4 5 6 7 8 9 10 11 +5 +10 +15 +20
MOTIF POSITION

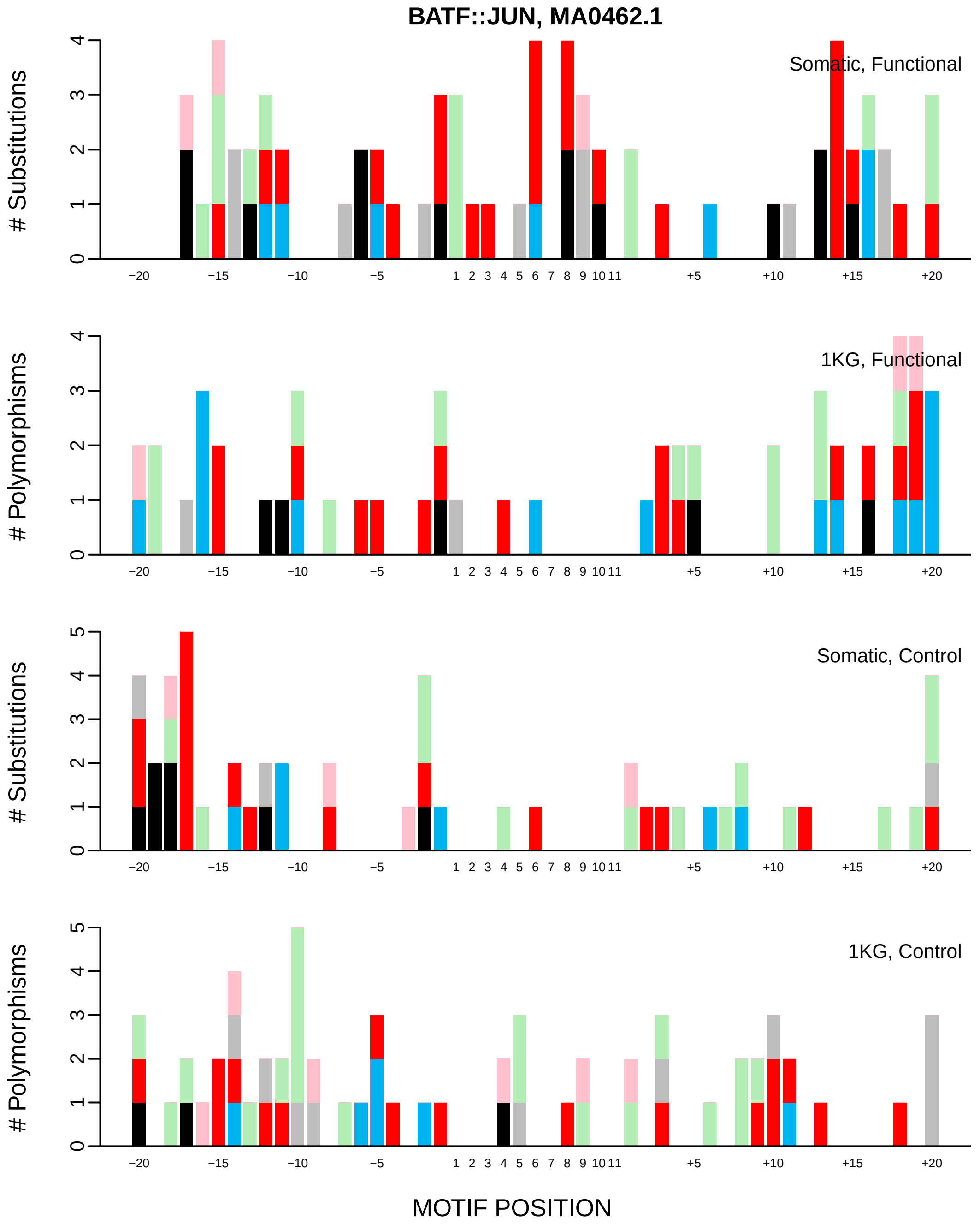
BATF::JUN, MA0462.1
Somatic, Functional
1KG, Functional
Somatic, Control
1KG, Control
Substitutions
Polymorphisms
−20
−15
−10
−5
1 2 3 4 5 6 7 8 9 10 11
+5
+10
+15
+20
MOTIF POSITION

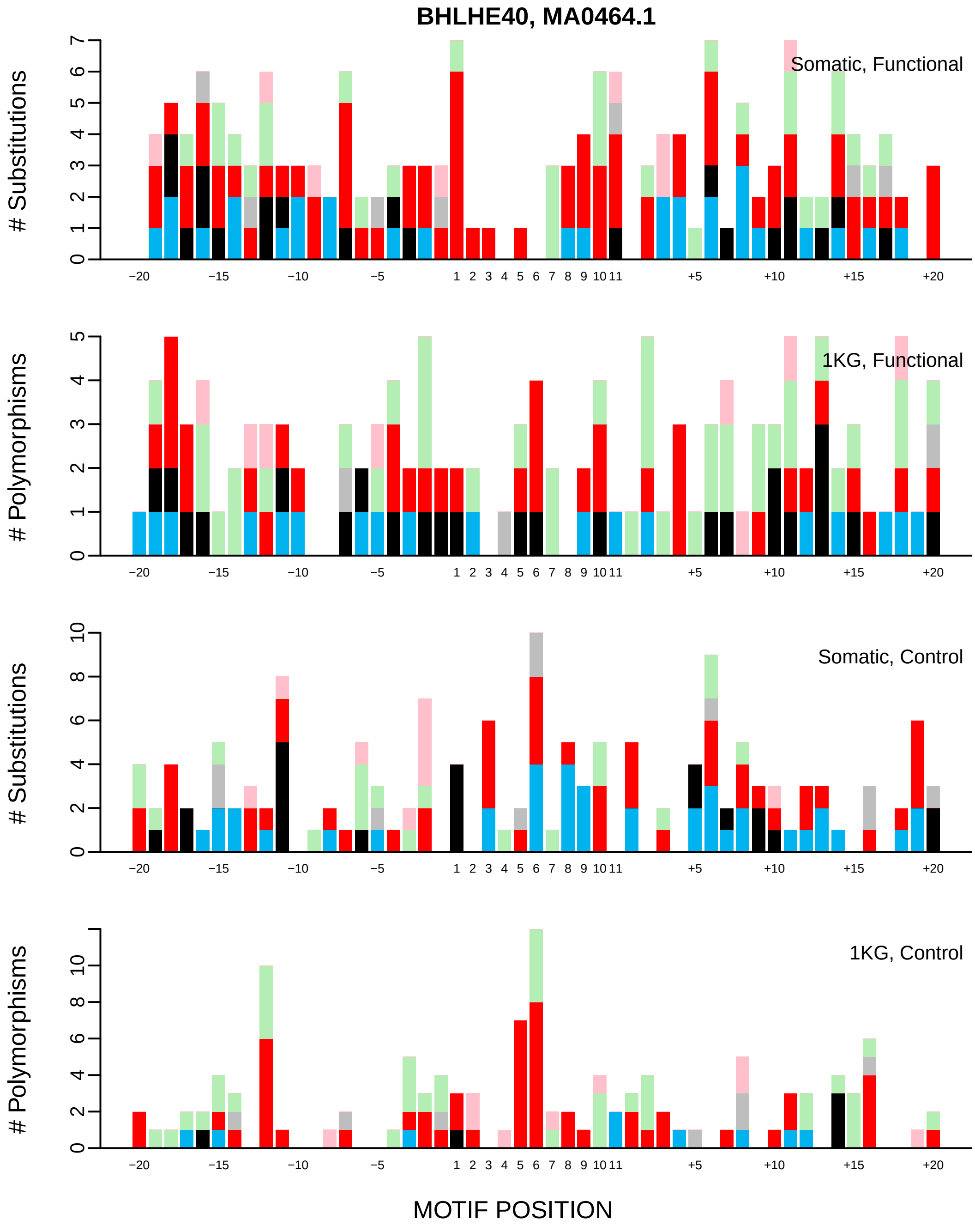
BHLHE40, MA0464.1
Substitutions
Somatic, Functional
Polymorphisms
1KG, Functional
Substitutions
Somatic, Control
Polymorphisms
1KG, Control
−20
−15
−10
−5
1 2 3 4 5 6 7 8 9 10 11
+5
+10
+15
+20
MOTIF POSITION

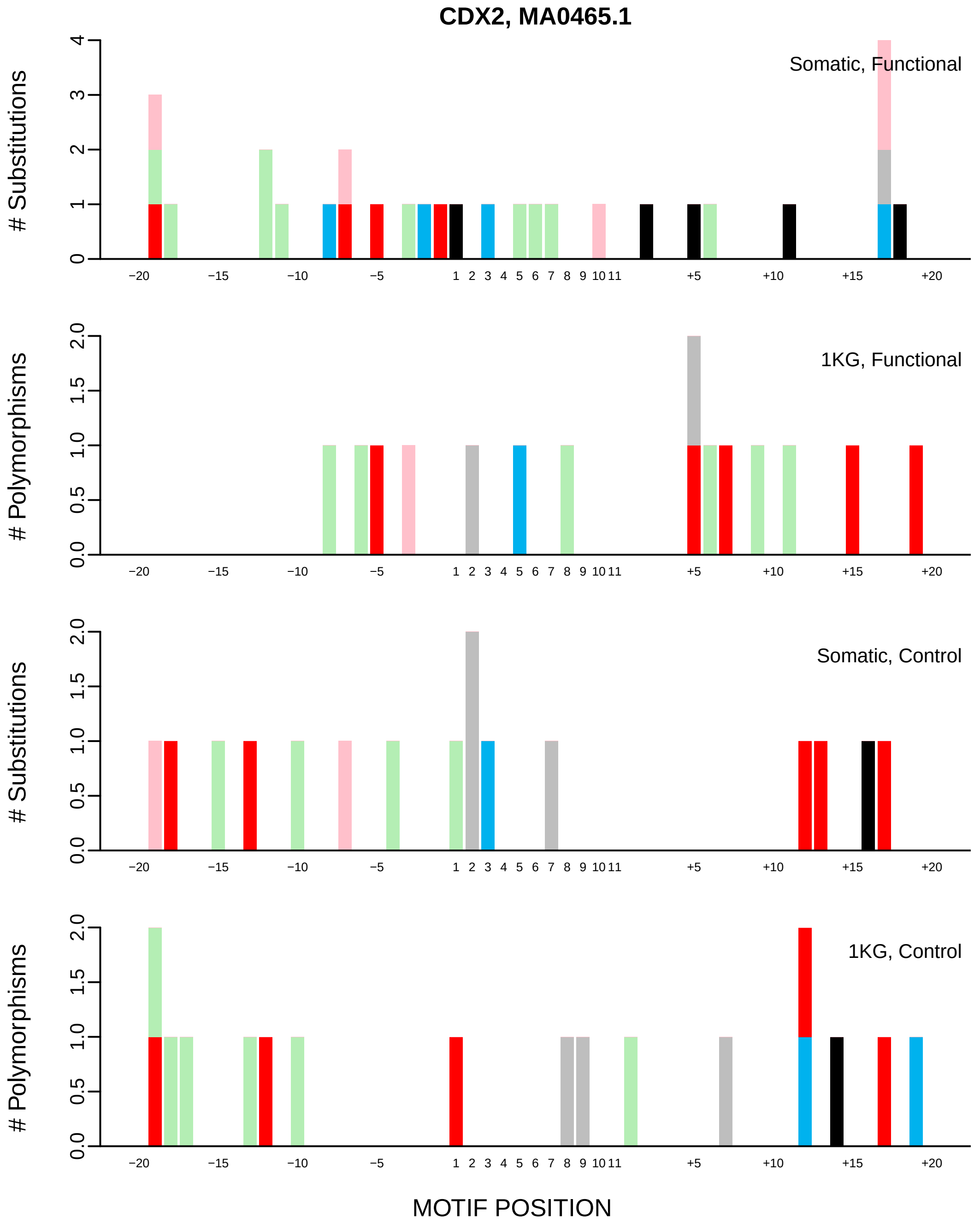
CDX2, MA0465.1
Somatic, Functional
1KG, Functional
Somatic, Control
1KG, Control
Substitutions
Polymorphisms
Substitutions
Polymorphisms
−20 −15 −10 −5 1 2 3 4 5 6 7 8 9 10 11 +5 +10 +15 +20
MOTIF POSITION

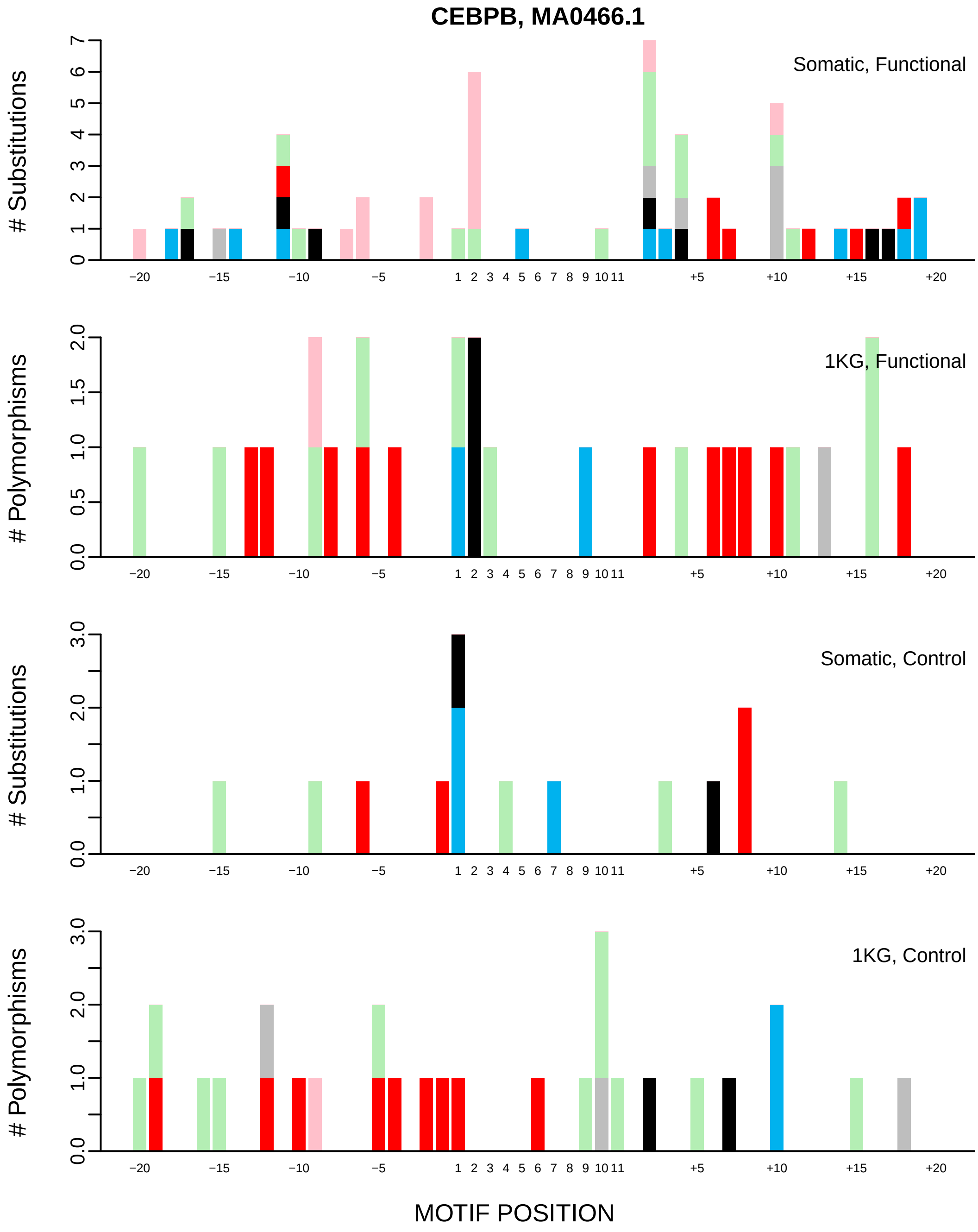
CEBPB, MA0466.1
Substitutions
Somatic, Functional
Polymorphisms
1KG, Functional
Substitutions
Somatic, Control
Polymorphisms
1KG, Control
−20
−15
−10
−5
1 2 3 4 5 6 7 8 9 10 11
+5
+10
+15
+20
MOTIF POSITION

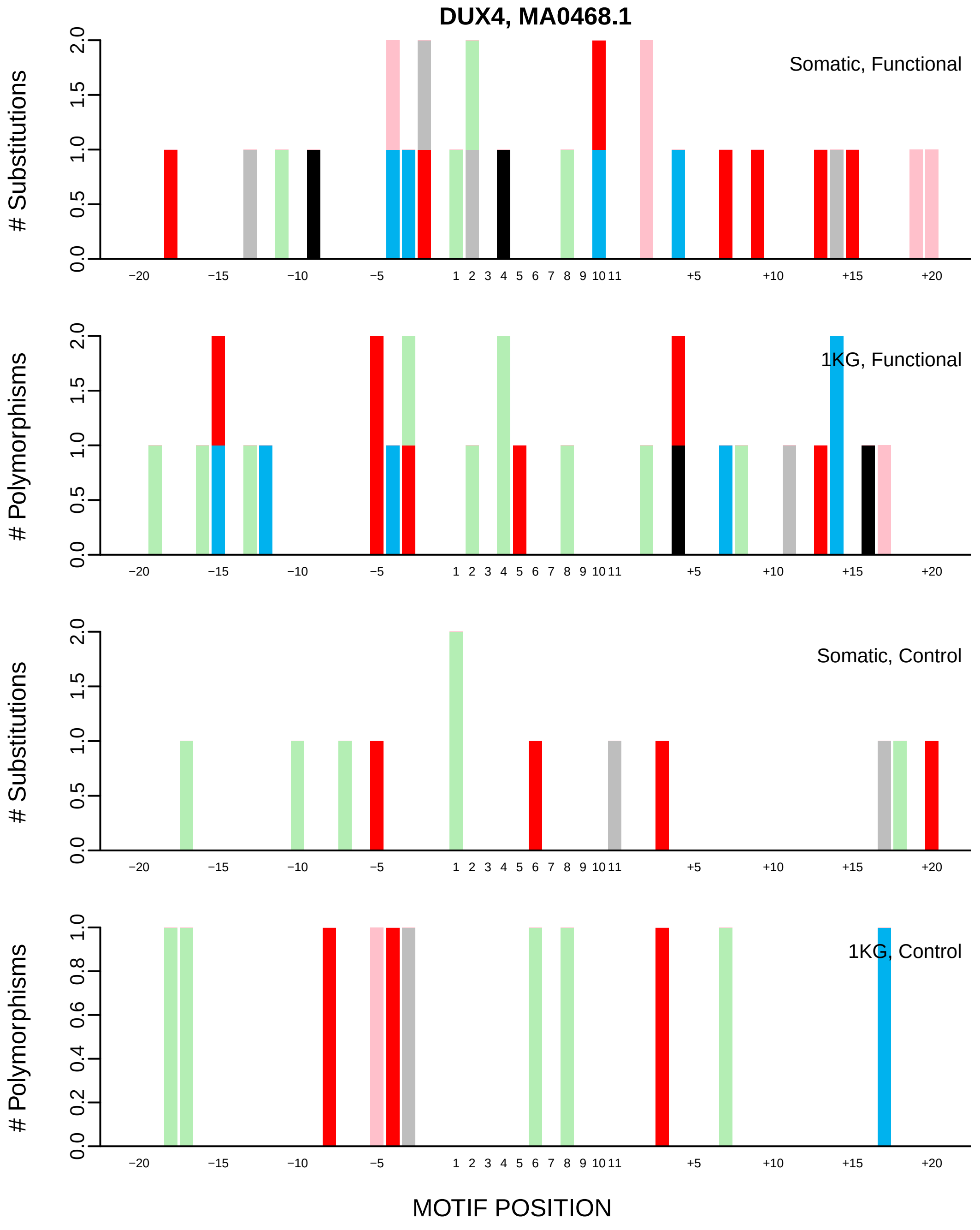
DUX4, MA0468.1
Substitutions
Somatic, Functional
Polymorphisms
1KG, Functional
Substitutions
Somatic, Control
Polymorphisms
1KG, Control
−20 −15 −10 −5 1 2 3 4 5 6 7 8 9 10 11 +5 +10 +15 +20
MOTIF POSITION

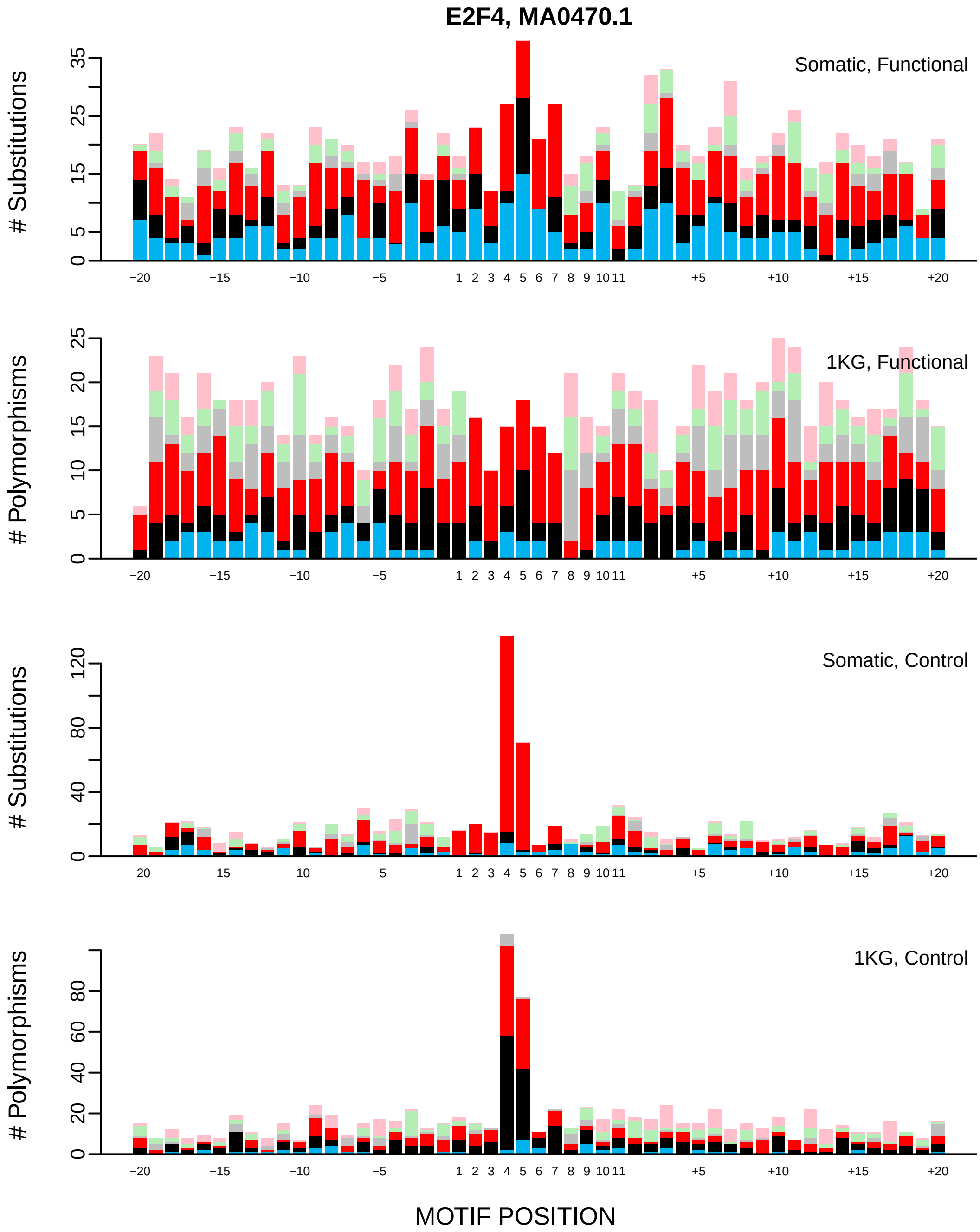
E2F4, MA0470.1
Substitutions
Somatic, Functional
Polymorphisms
1KG, Functional
Substitutions
Somatic, Control
Polymorphisms
1KG, Control
MOTIF POSITION

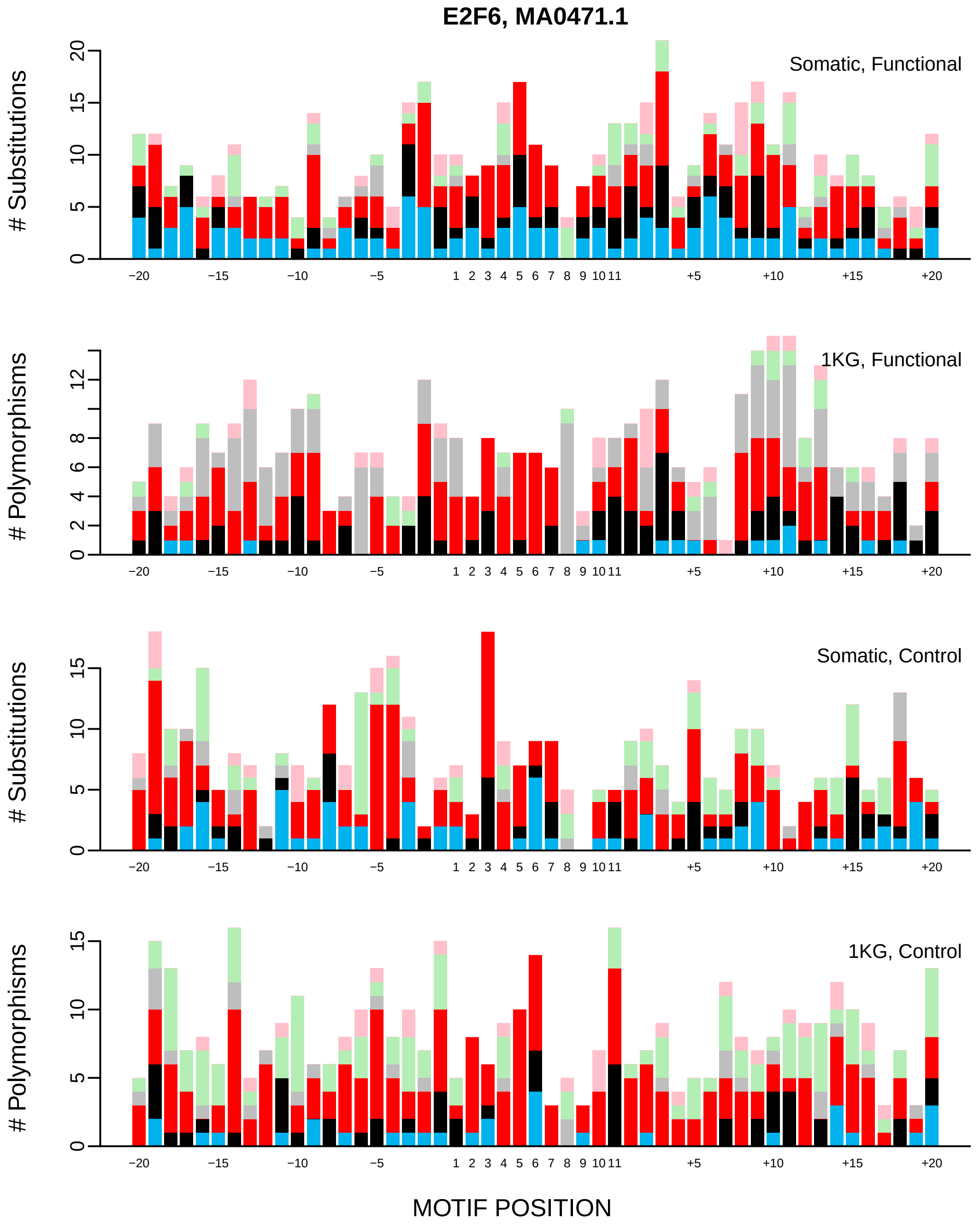

E2F6, MA0471.1
Substitutions
Somatic, Functional
Polymorphisms
1KG, Functional
Substitutions
Somatic, Control
Polymorphisms
1KG, Control
−20
−15
−10
−5
1 2 3 4 5 6 7 8 9 10 11
+5
+10
+15
+20
MOTIF POSITION

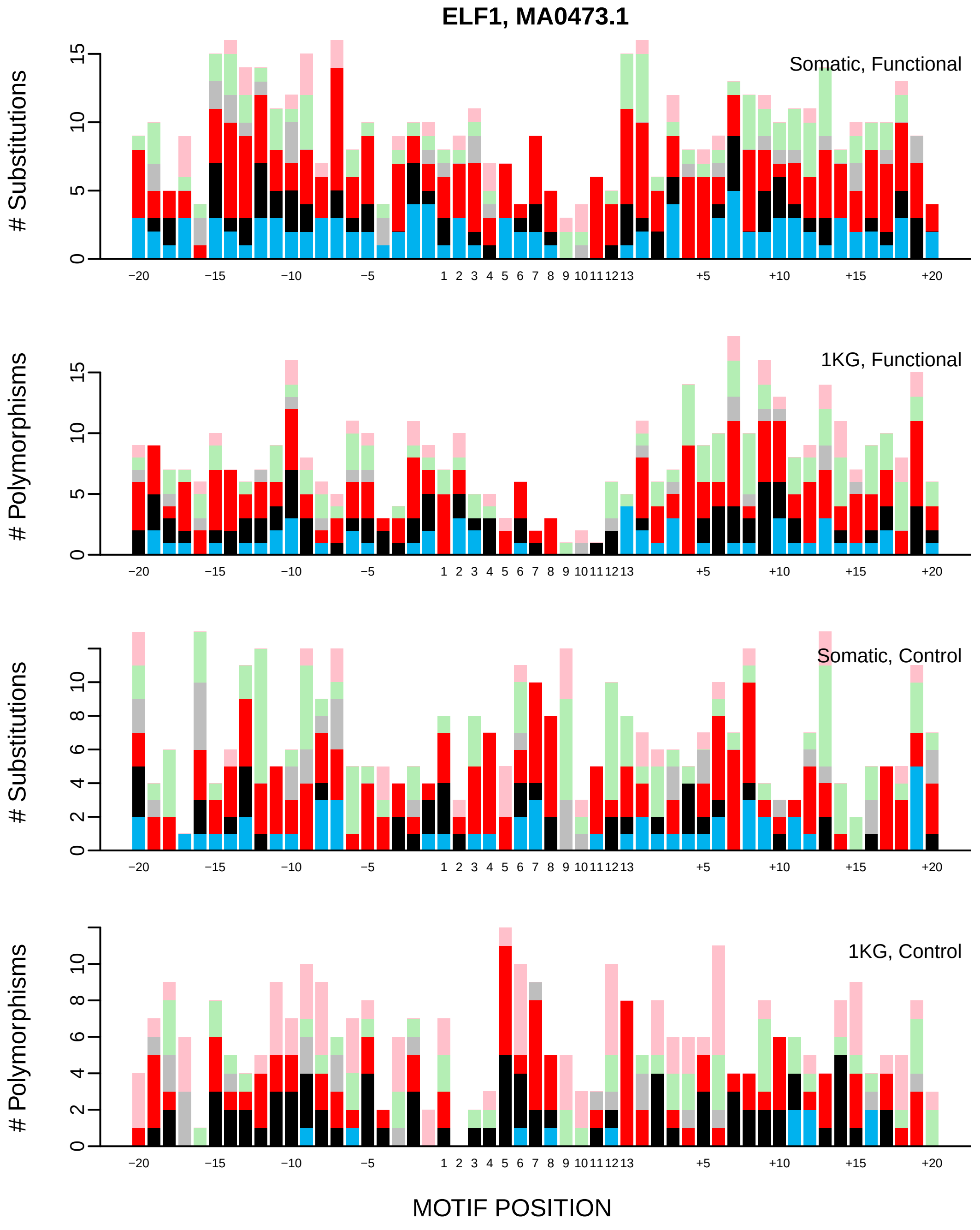

ELF1, MA0473.1
Substitutions
Somatic, Functional
Polymorphisms
1KG, Functional
Substitutions
Somatic, Control
Polymorphisms
1KG, Control
−20 −15 −10 −5 1 2 3 4 5 6 7 8 9 10 11 12 13 +5 +10 +15 +20
MOTIF POSITION

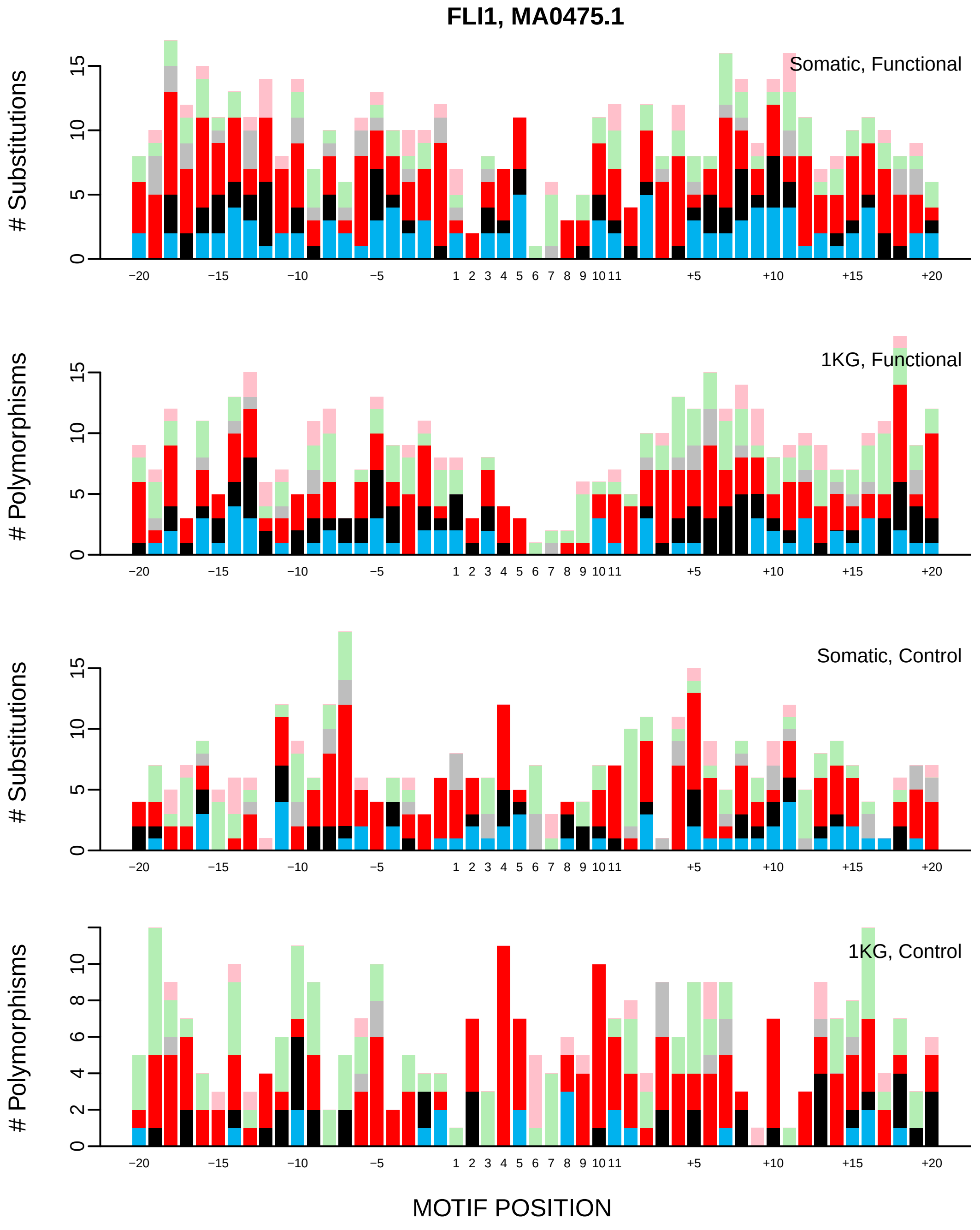
FLI1, MA0475.1
Substitutions
Somatic, Functional
Polymorphisms
1KG, Functional
Substitutions
Somatic, Control
Polymorphisms
1KG, Control
−20
−15
−10
−5
1
2
3
4
5
6
7
8
9
10
11
+5
+10
+15
+20
MOTIF POSITION

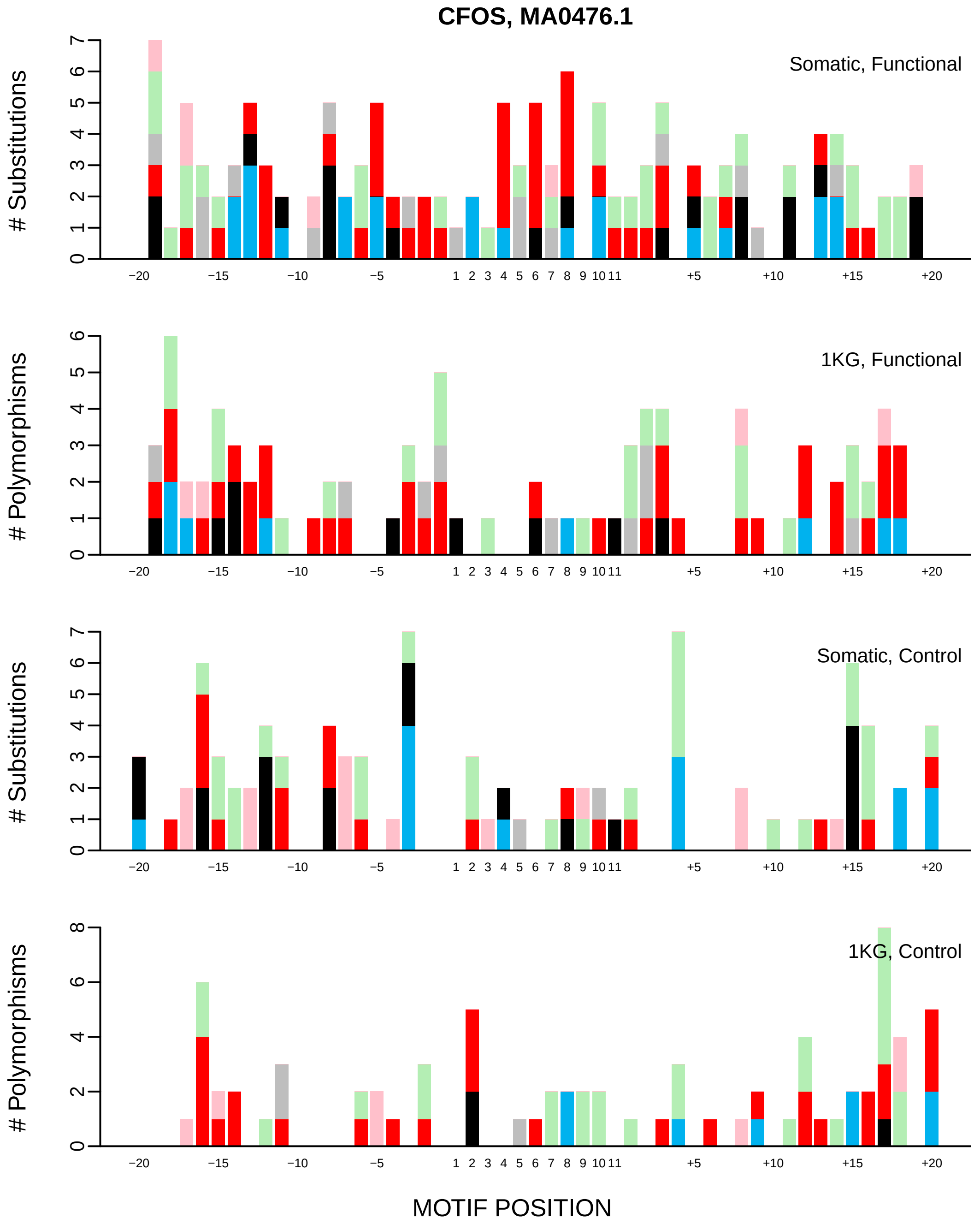
CFOS, MA0476.1
Substitutions
Somatic, Functional
Polymorphisms
1KG, Functional
Substitutions
Somatic, Control
Polymorphisms
1KG, Control
−20 −15 −10 −5 1 2 3 4 5 6 7 8 9 10 11 +5 +10 +15 +20
MOTIF POSITION

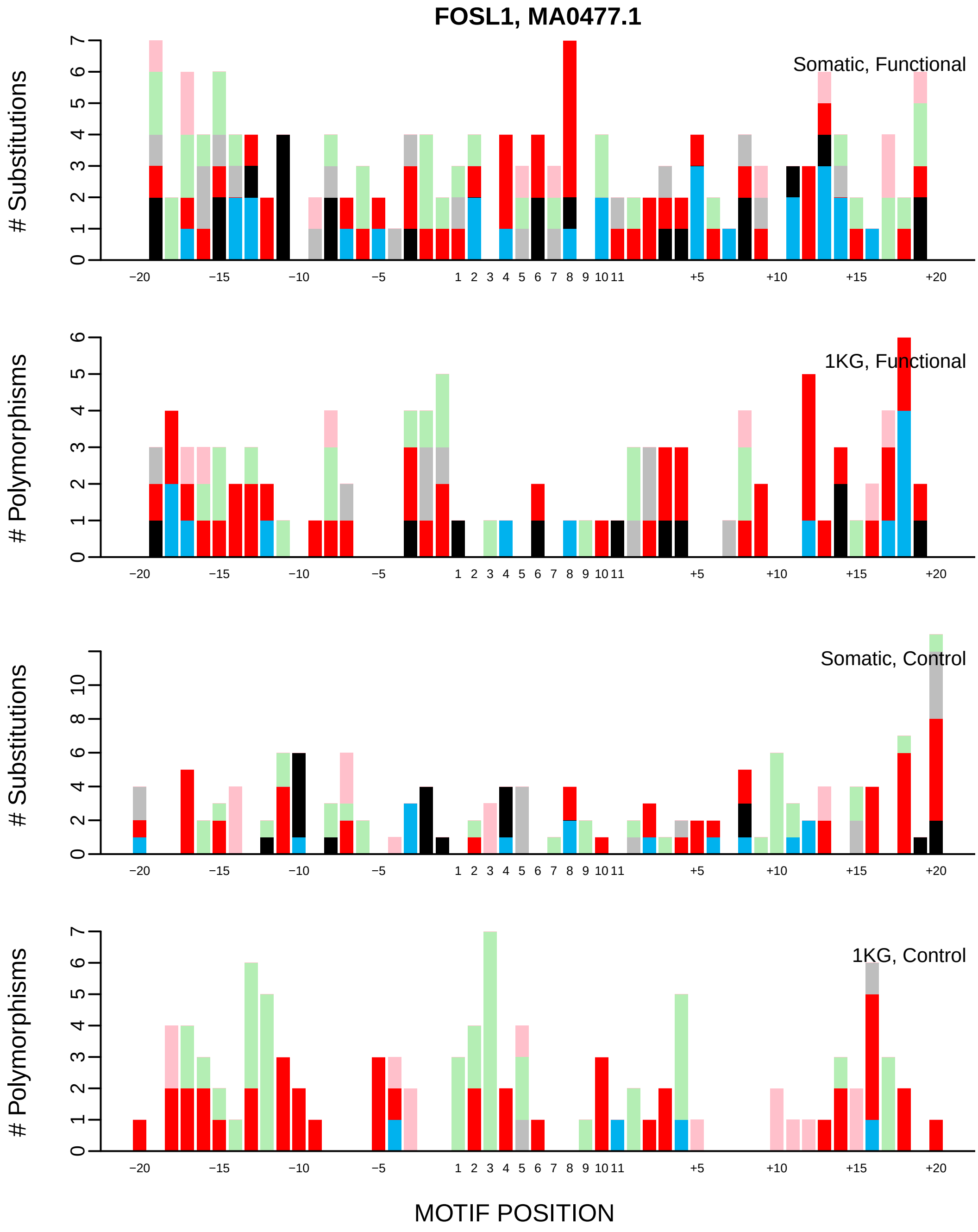
FOSL1, MA0477.1
Somatic, Functional
1KG, Functional
Somatic, Control
1KG, Control
Substitutions
Polymorphisms
−20
−15
−10
−5
1 2 3 4 5 6 7 8 9 10 11
+5
+10
+15
+20
MOTIF POSITION

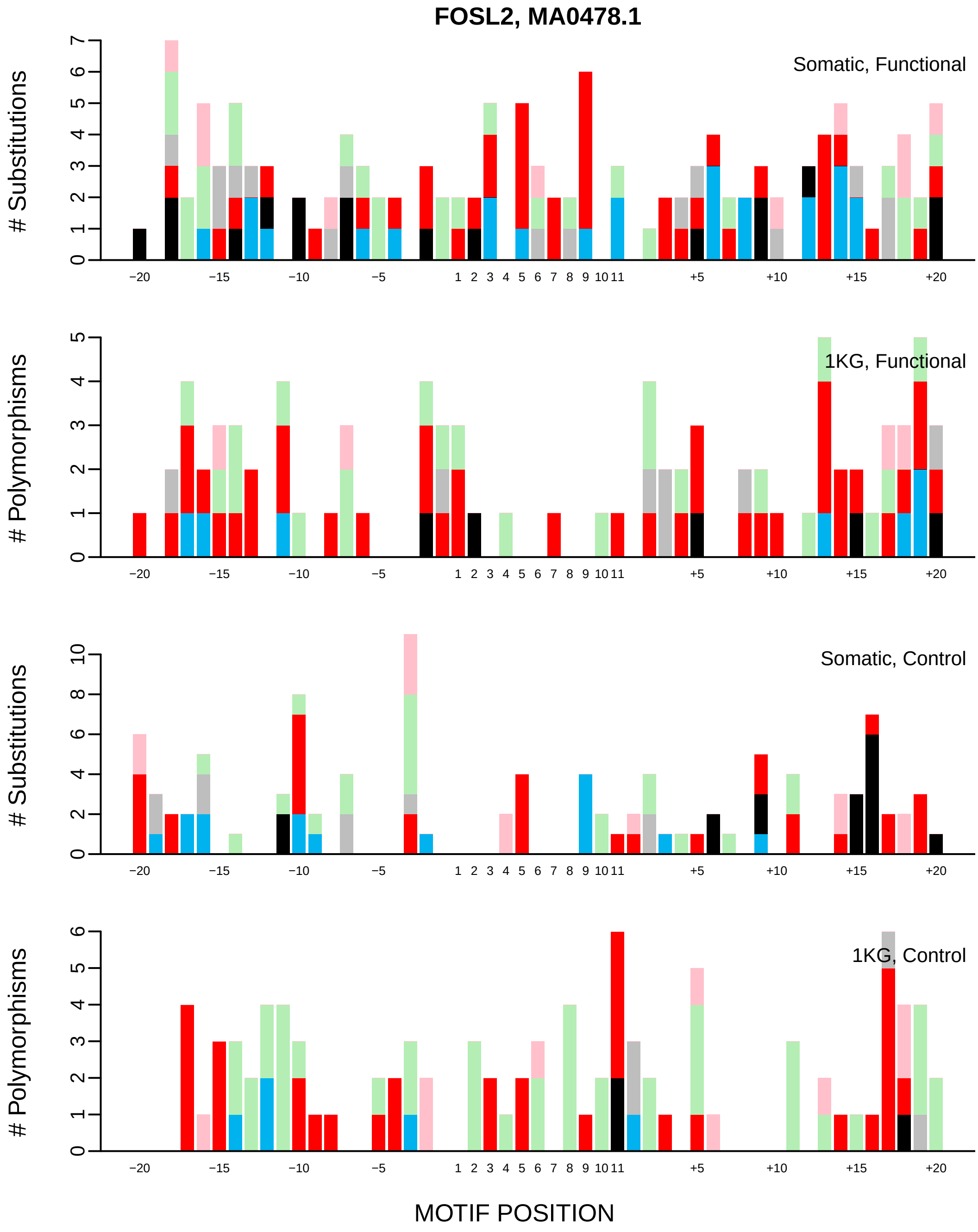

FOSL2, MA0478.1
Substitutions
Somatic, Functional
Polymorphisms
1KG, Functional
Substitutions
Somatic, Control
Polymorphisms
1KG, Control
−20 −15 −10 −5 1 2 3 4 5 6 7 8 9 10 11 +5 +10 +15 +20
MOTIF POSITION

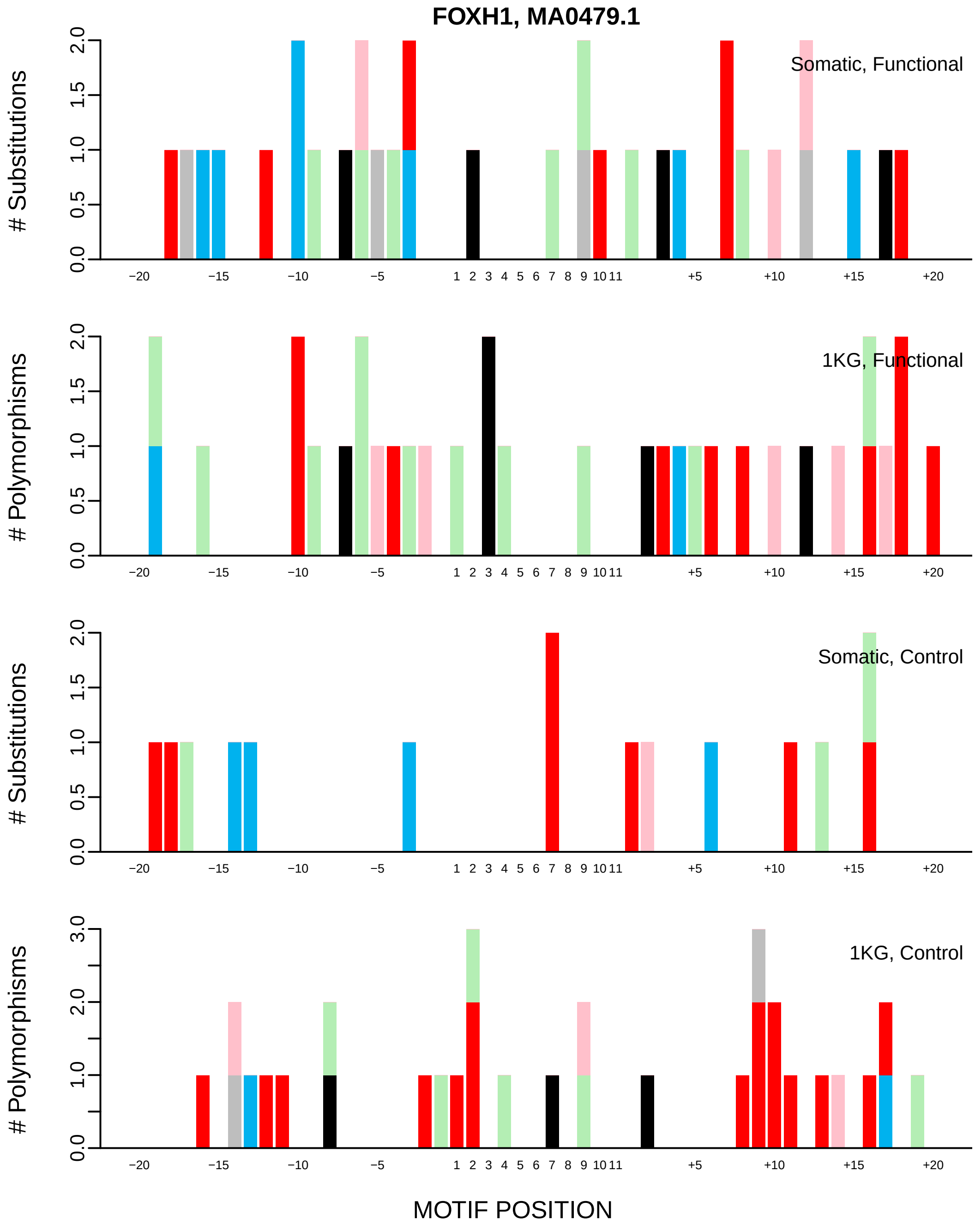

FOXH1, MA0479.1
Substitutions
Somatic, Functional
Polymorphisms
1KG, Functional
Substitutions
Somatic, Control
Polymorphisms
1KG, Control
0.0
0.5
1.0
1.5
2.0
3.0
−20
−15
−10
−5
1 2 3 4 5 6 7 8 9 10 11
+5
+10
+15
+20
MOTIF POSITION

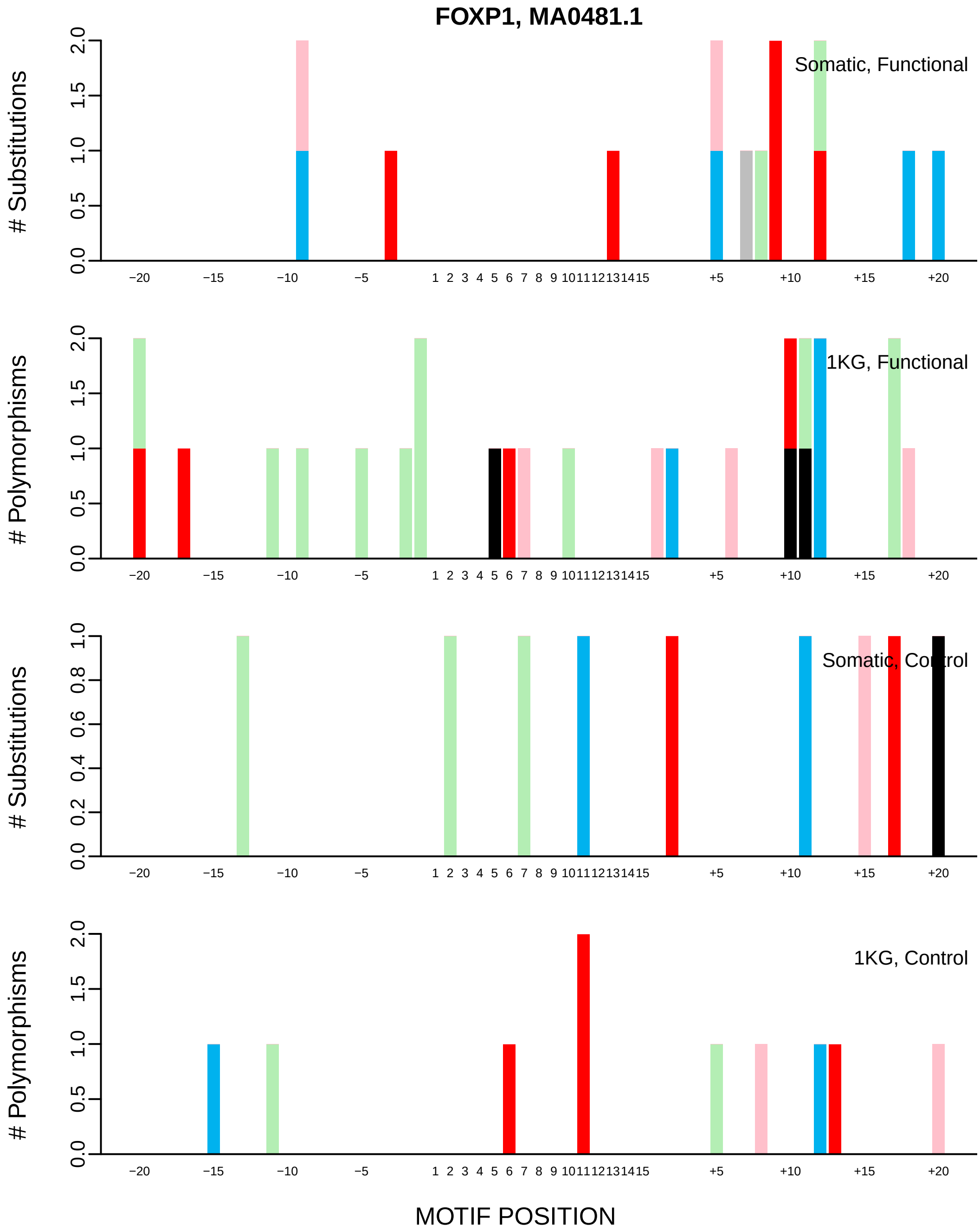
FOXP1, MA0481.1
Substitutions
Somatic, Functional
Polymorphisms
1KG, Functional
Substitutions
Somatic, Control
Polymorphisms
1KG, Control
−20 −15 −10 −5 1 2 3 4 5 6 7 8 9 10 11 12 13 14 15 +5 +10 +15 +20
MOTIF POSITION

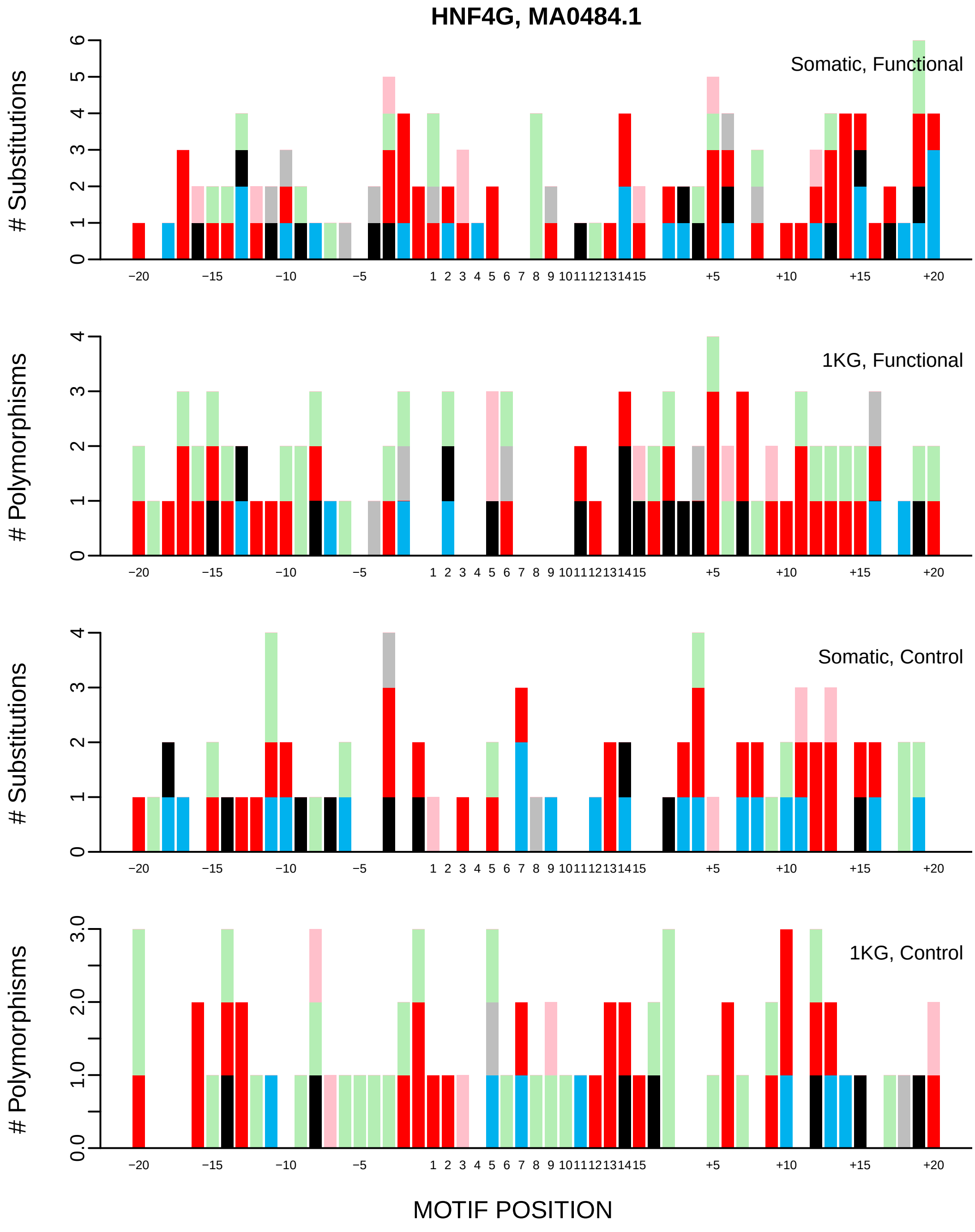

HNF4G, MA0484.1
Substitutions
Somatic, Functional
Polymorphisms
1KG, Functional
Substitutions
Somatic, Control
Polymorphisms
1KG, Control
−20 −15 −10 −5 1 2 3 4 5 6 7 8 9 10 11 12 13 14 15 +5 +10 +15 +20
MOTIF POSITION

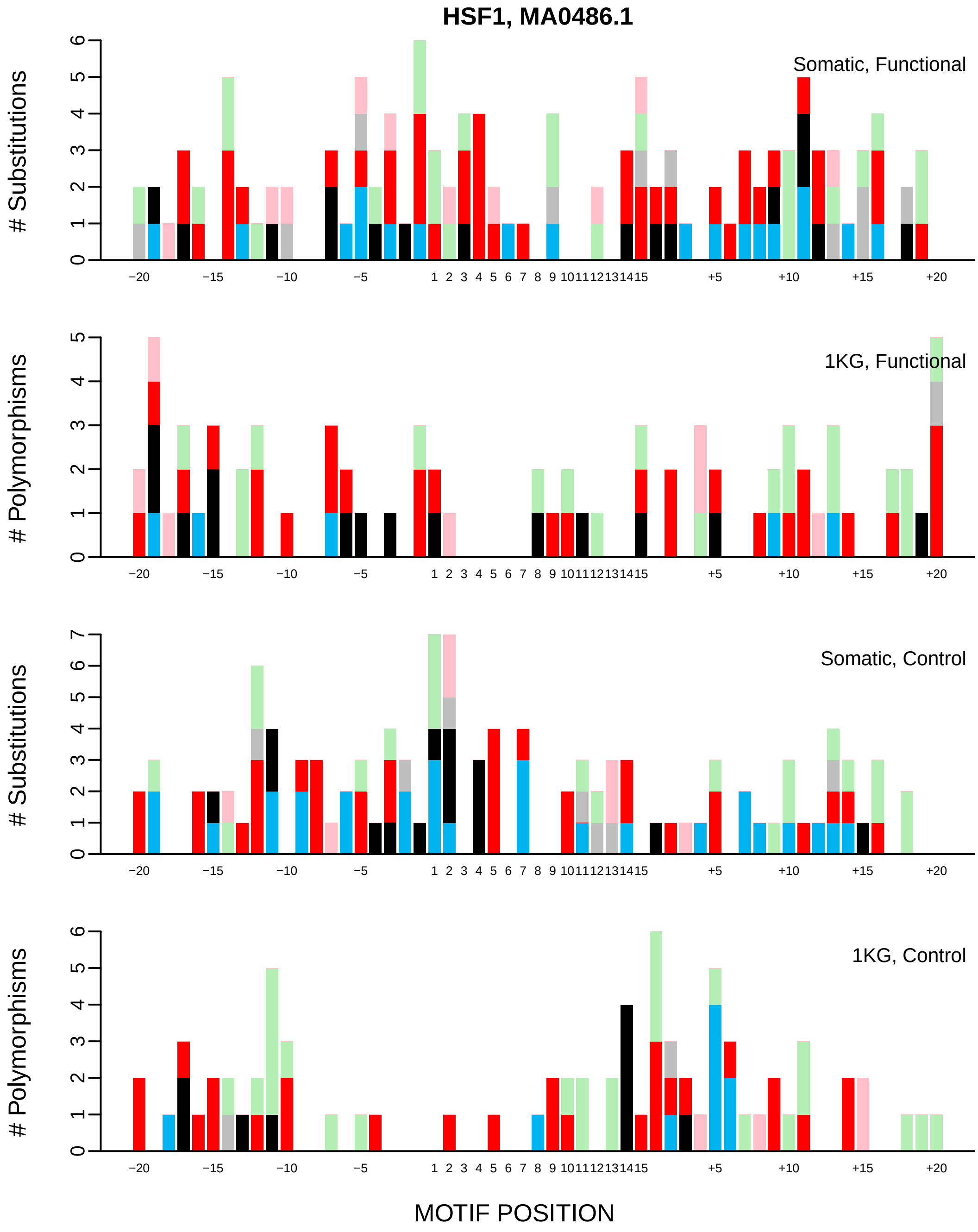
HSF1, MA0486.1
Substitutions
Somatic, Functional
Polymorphisms
1KG, Functional
Substitutions
Somatic, Control
Polymorphisms
1KG, Control
−20 −15 −10 −5 1 2 3 4 5 6 7 8 9 10 11 12 13 14 15 +5 +10 +15 +20
MOTIF POSITION

CJUN, MA0488.1

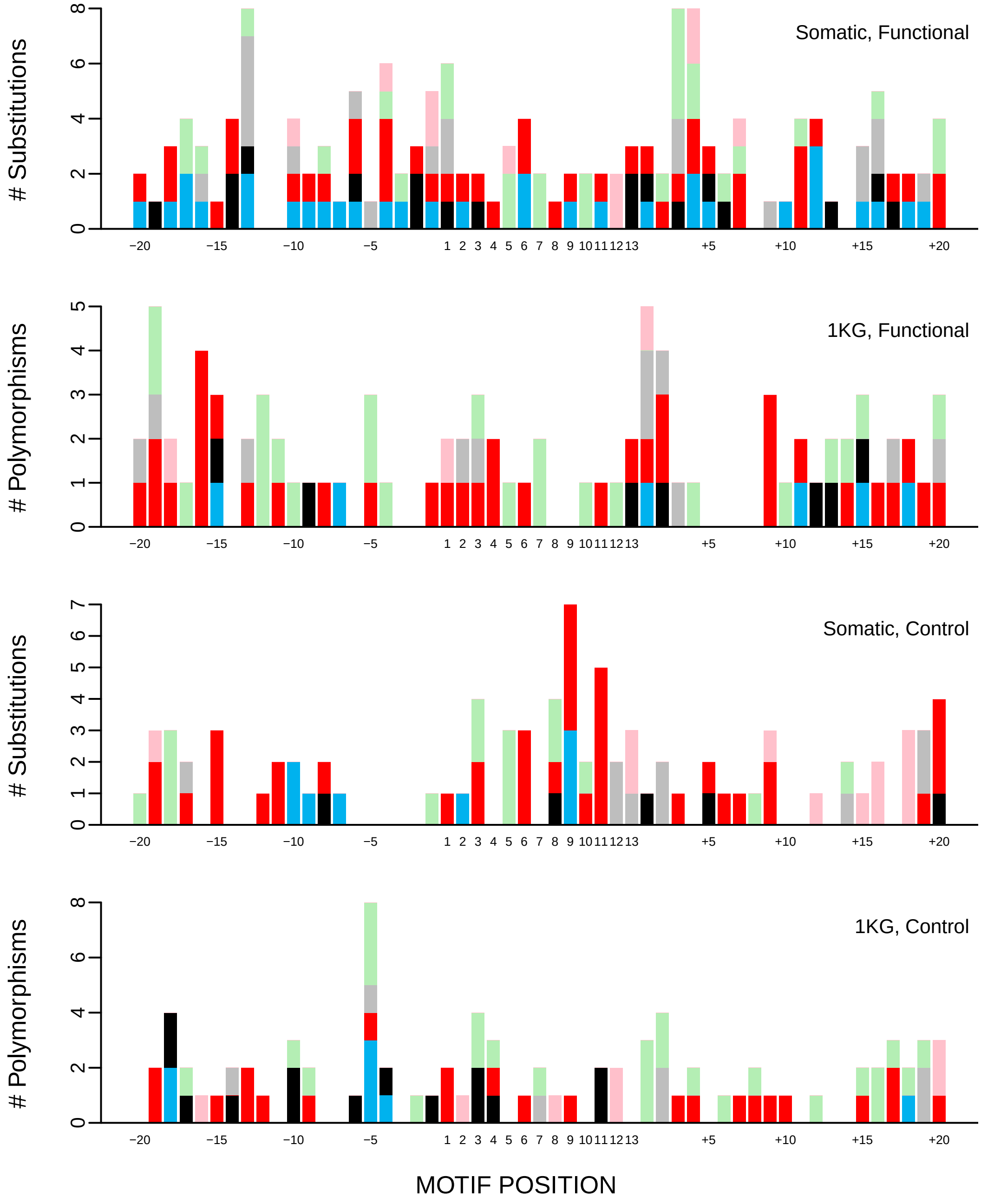

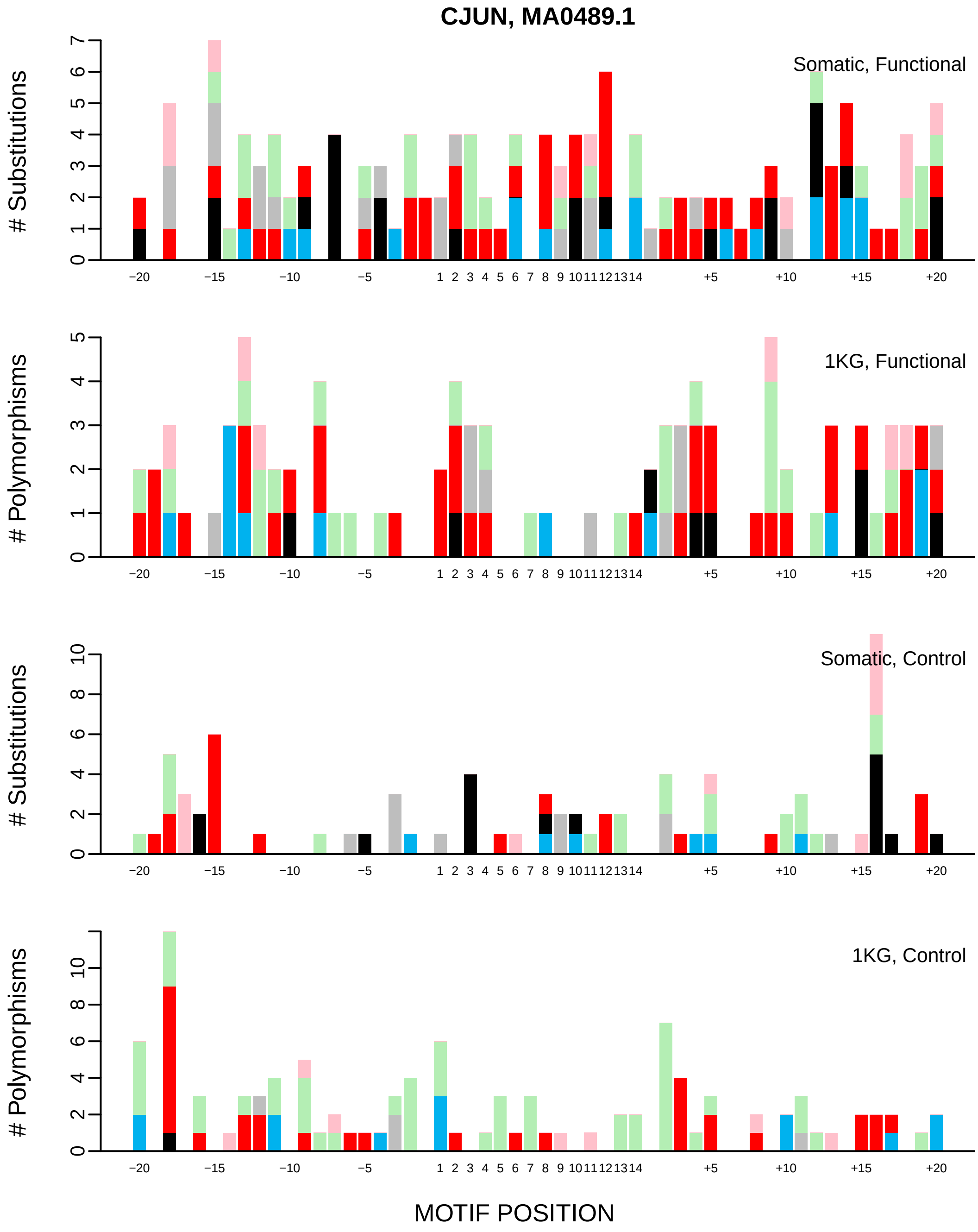

CJUN, MA0489.1
Substitutions
Somatic, Functional
Polymorphisms
1KG, Functional
Substitutions
Somatic, Control
Polymorphisms
1KG, Control
−20
−15
−10
−5
1 2 3 4 5 6 7 8 9 10 11 12 13 14
+5
+10
+15
+20
MOTIF POSITION

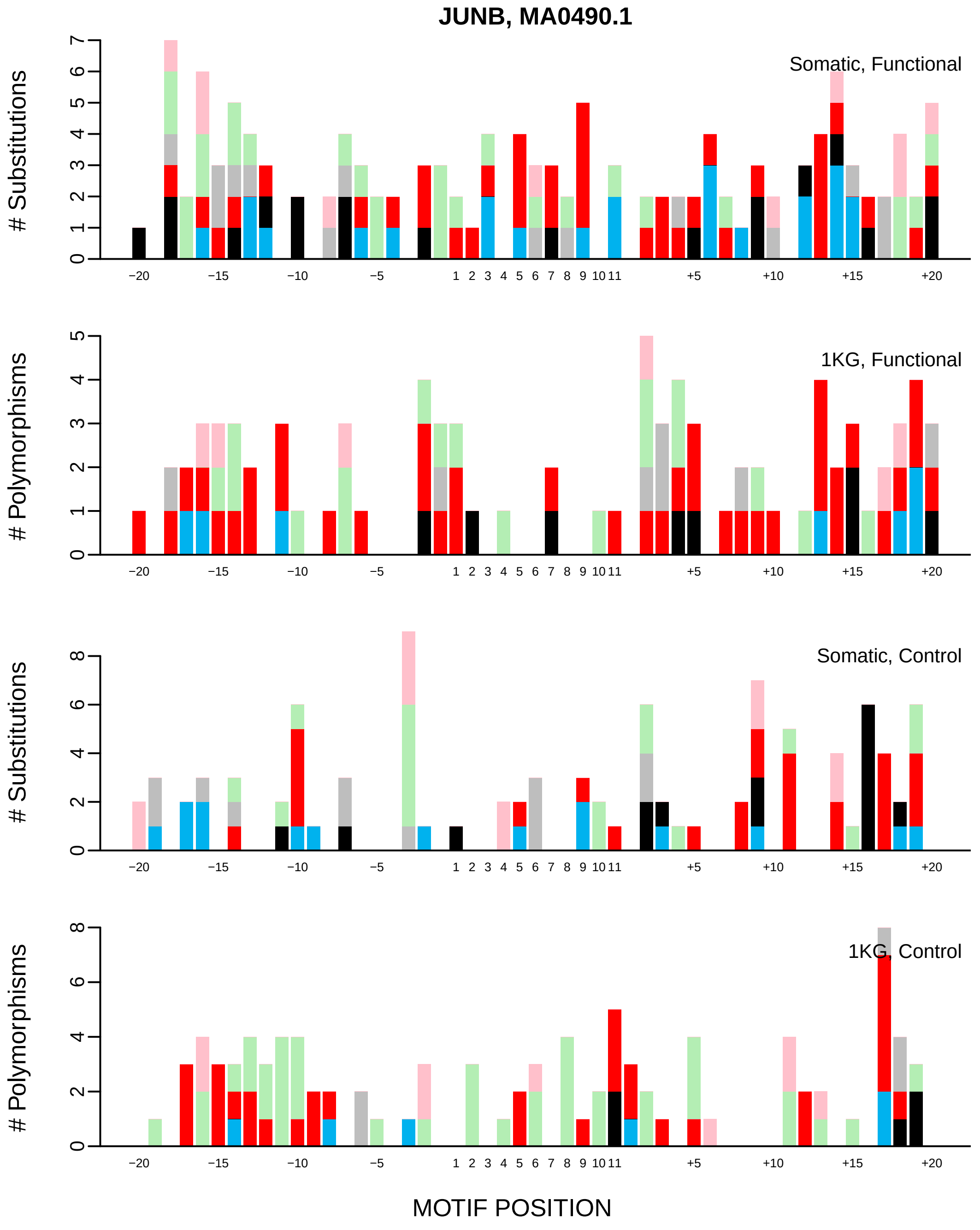

JUNB, MA0490.1
Substitutions
Somatic, Functional
Polymorphisms
1KG, Functional
Substitutions
Somatic, Control
Polymorphisms
1KG, Control
−20
−15
−10
−5
1 2 3 4 5 6 7 8 9 10 11
+5
+10
+15
+20
MOTIF POSITION

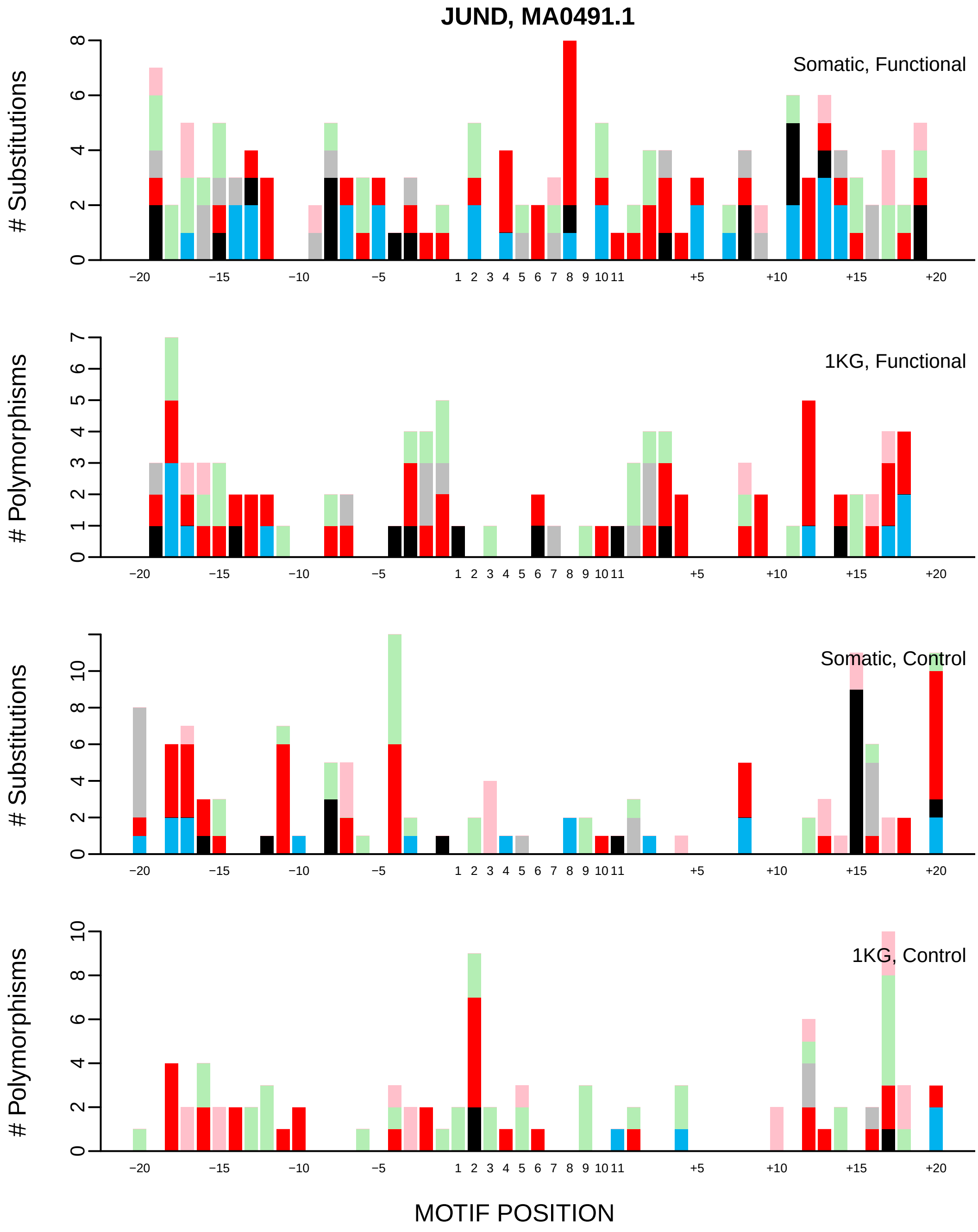
JUND, MA0491.1
Substitutions
Somatic, Functional
Polymorphisms
1KG, Functional
Substitutions
Somatic, Control
Polymorphisms
1KG, Control
−20
−15
−10
−5
1 2 3 4 5 6 7 8 9 10 11
+5
+10
+15
+20
MOTIF POSITION

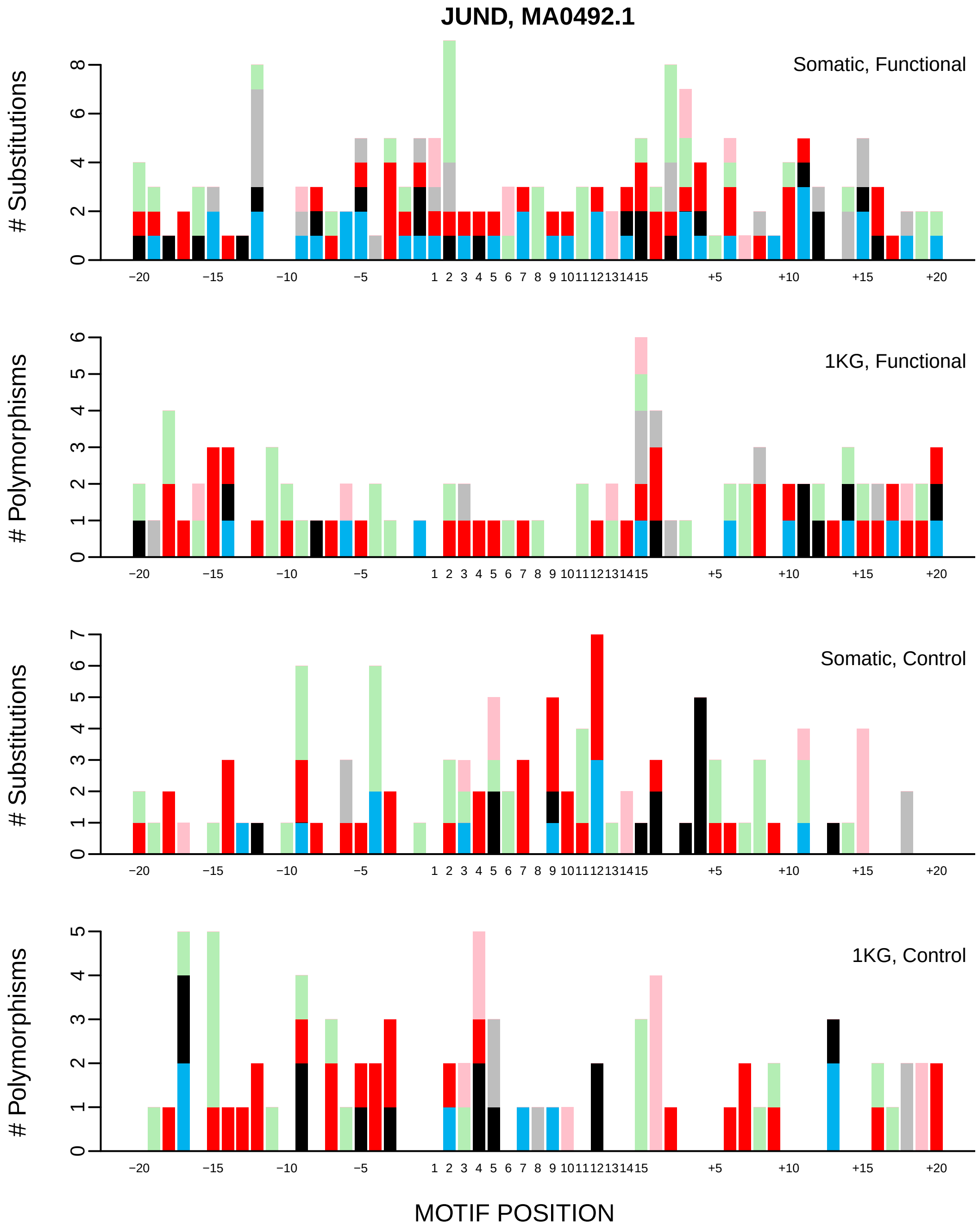
JUND, MA0492.1
Somatic, Functional
1KG, Functional
Somatic, Control
1KG, Control
Substitutions
Polymorphisms
−20
−15
−10
−5
1 2 3 4 5 6 7 8 9 10 11 12 13 14 15
+5
+10
+15
+20
MOTIF POSITION

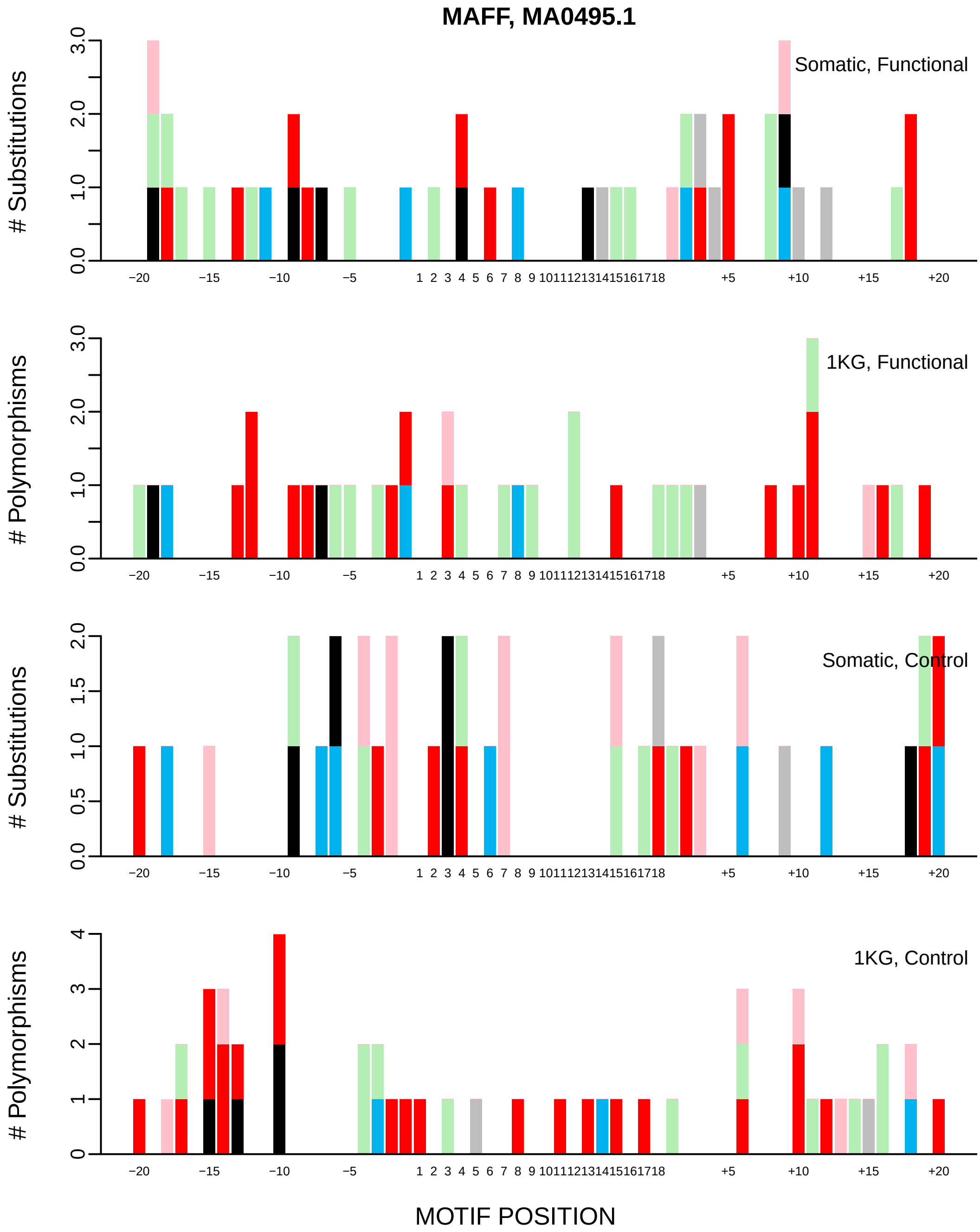
MAFF, MA0495.1
Substitutions
Somatic, Functional
Polymorphisms
1KG, Functional
Substitutions
Somatic, Control
Polymorphisms
1KG, Control
MOTIF POSITION

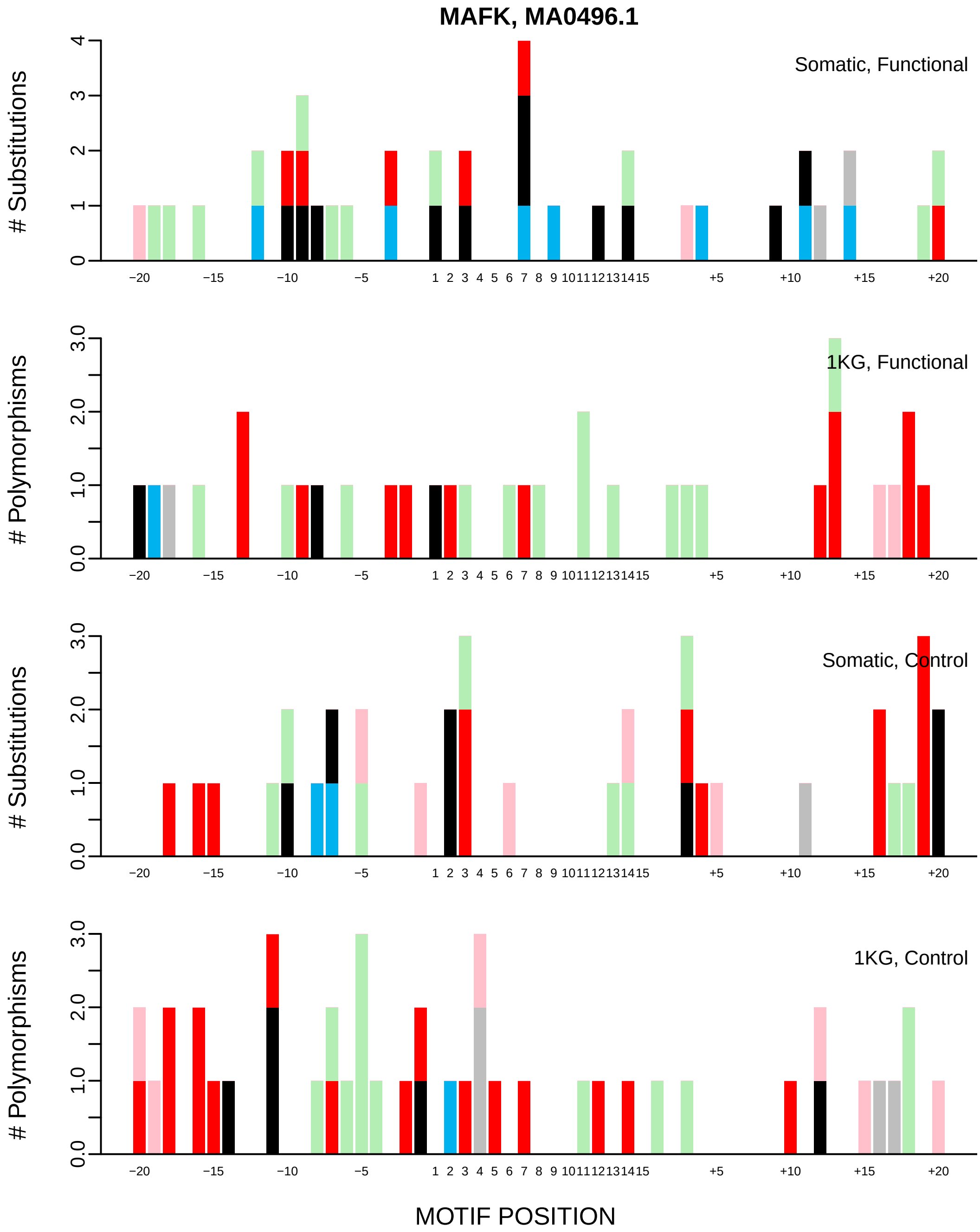
MAFK, MA0496.1
Somatic, Functional
1KG, Functional
Somatic, Control
1KG, Control
Substitutions
Polymorphisms
−20
−15
−10
−5
1 2 3 4 5 6 7 8 9 10 11 12 13 14 15
+5
+10
+15
+20
MOTIF POSITION

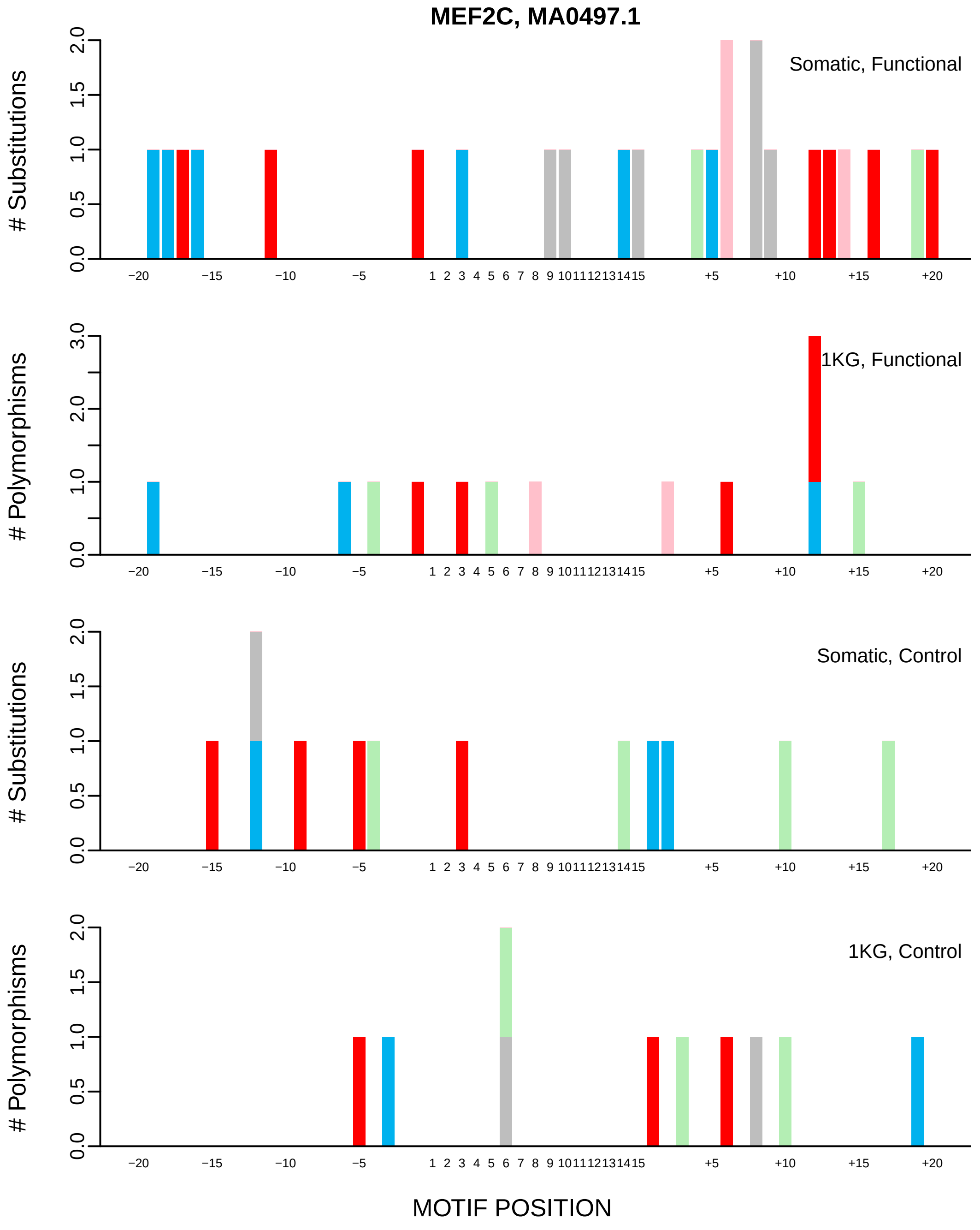
MEF2C, MA0497.1
Substitutions
Somatic, Functional
Polymorphisms
1KG, Functional
Substitutions
Somatic, Control
Polymorphisms
1KG, Control
−20 −15 −10 −5 1 2 3 4 5 6 7 8 9 10 11 12 13 14 15 +5 +10 +15 +20
MOTIF POSITION

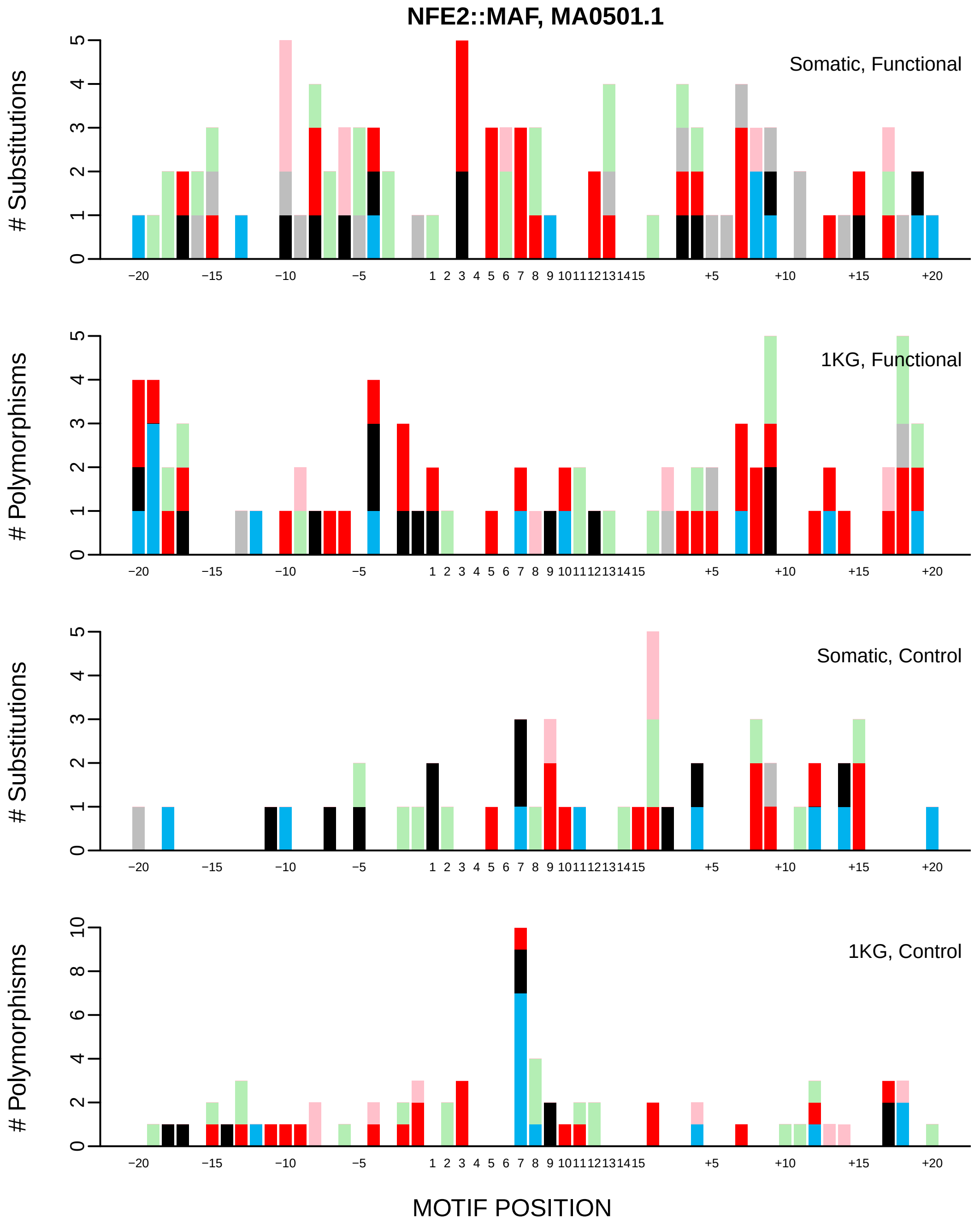
NFE2::MAF, MA0501.1
Substitutions
Somatic, Functional
Polymorphisms
1KG, Functional
Substitutions
Somatic, Control
Polymorphisms
1KG, Control
−20
−15
−10
−5
1 2 3 4 5 6 7 8 9 10 11 12 13 14 15
+5
+10
+15
+20
MOTIF POSITION

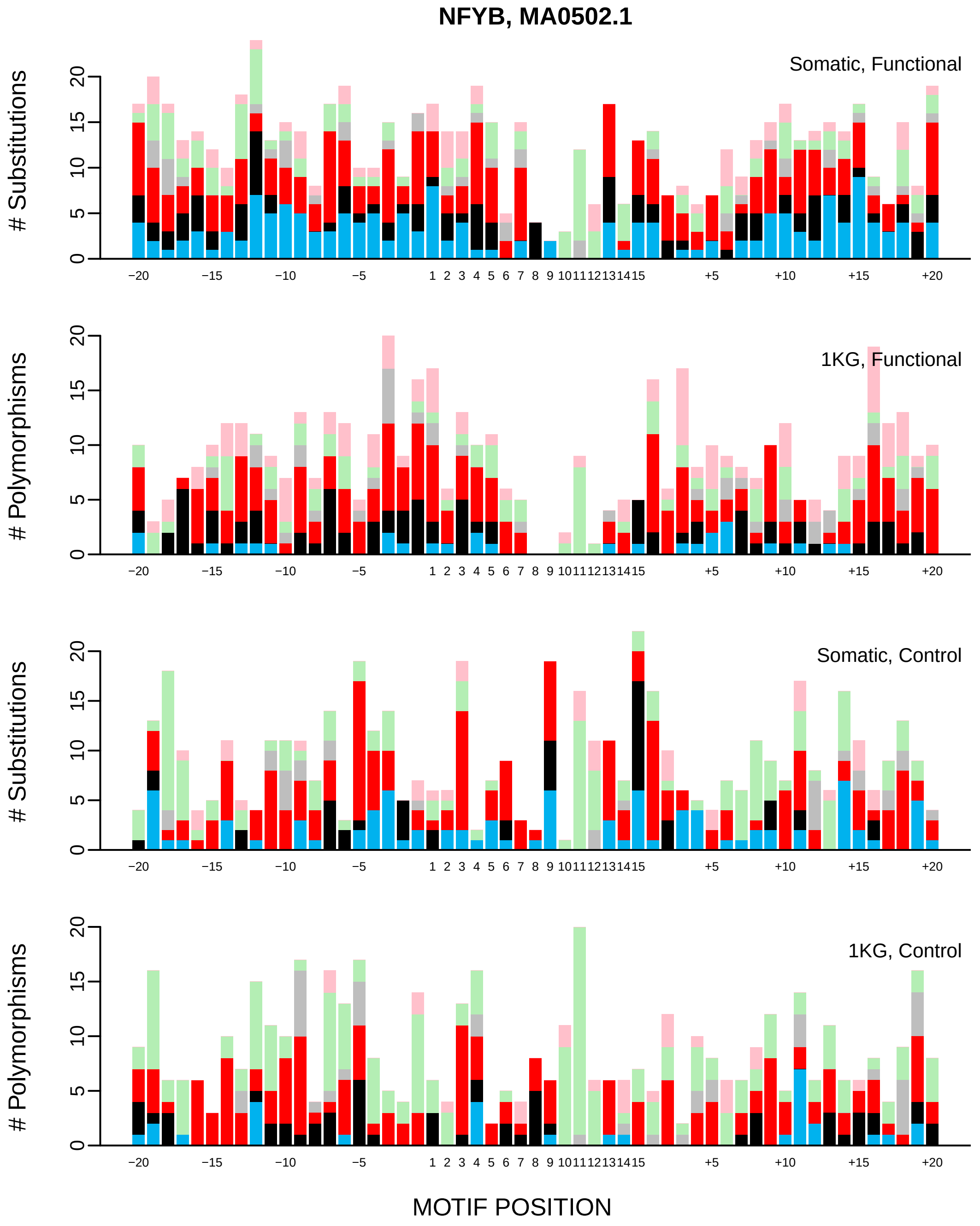

NFYB, MA0502.1
Somatic, Functional
1KG, Functional
Somatic, Control
1KG, Control
Substitutions
Polymorphisms
0
5
10
15
20
−20
−15
−10
−5
1 2 3 4 5 6 7 8 9 10 11 12 13 14 15
+5
+10
+15
+20
MOTIF POSITION

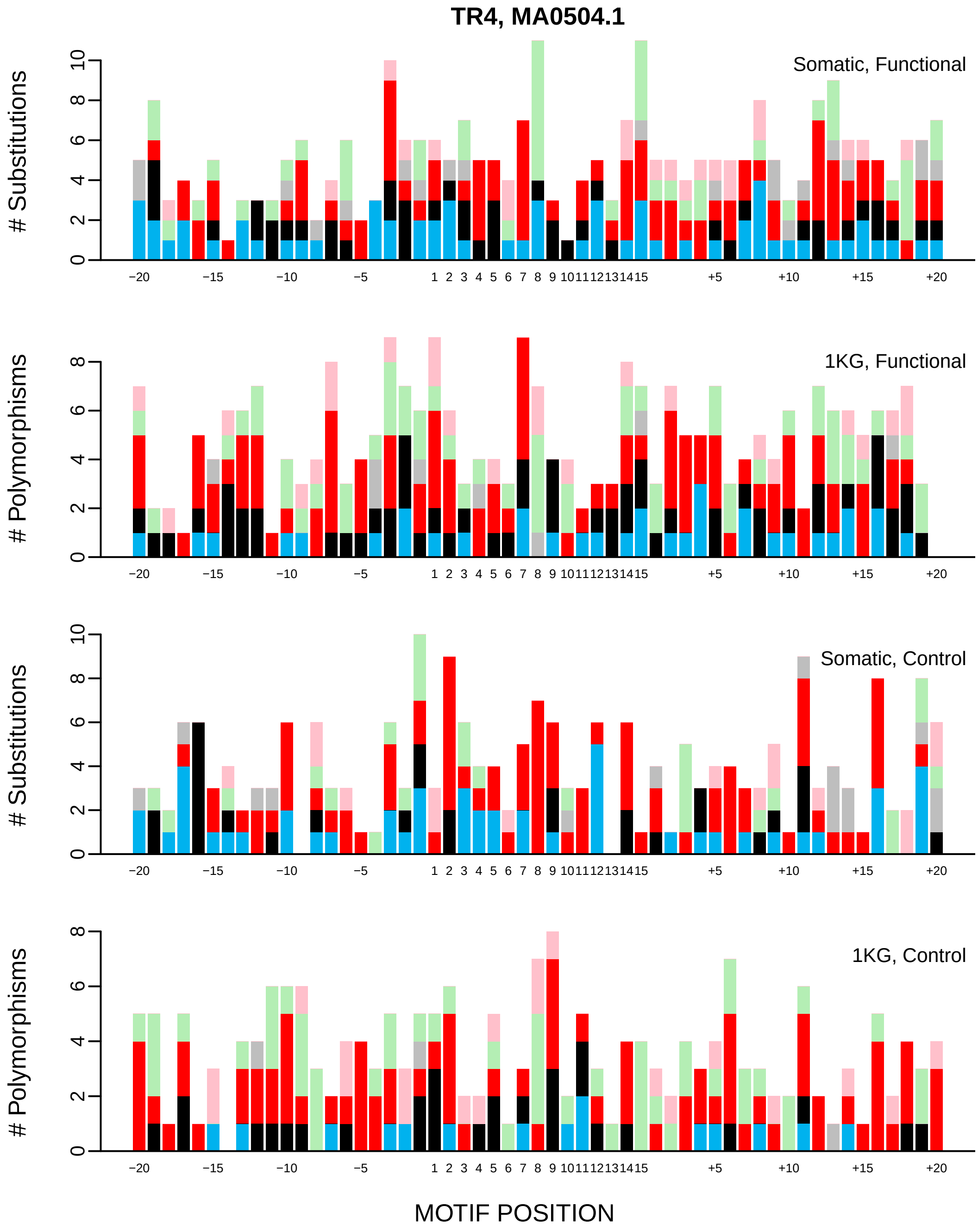

TR4, MA0504.1
Substitutions
Somatic, Functional
Polymorphisms
1KG, Functional
Substitutions
Somatic, Control
Polymorphisms
1KG, Control
−20 −15 −10 −5 1 2 3 4 5 6 7 8 9 10 11 12 13 14 15 +5 +10 +15 +20
MOTIF POSITION

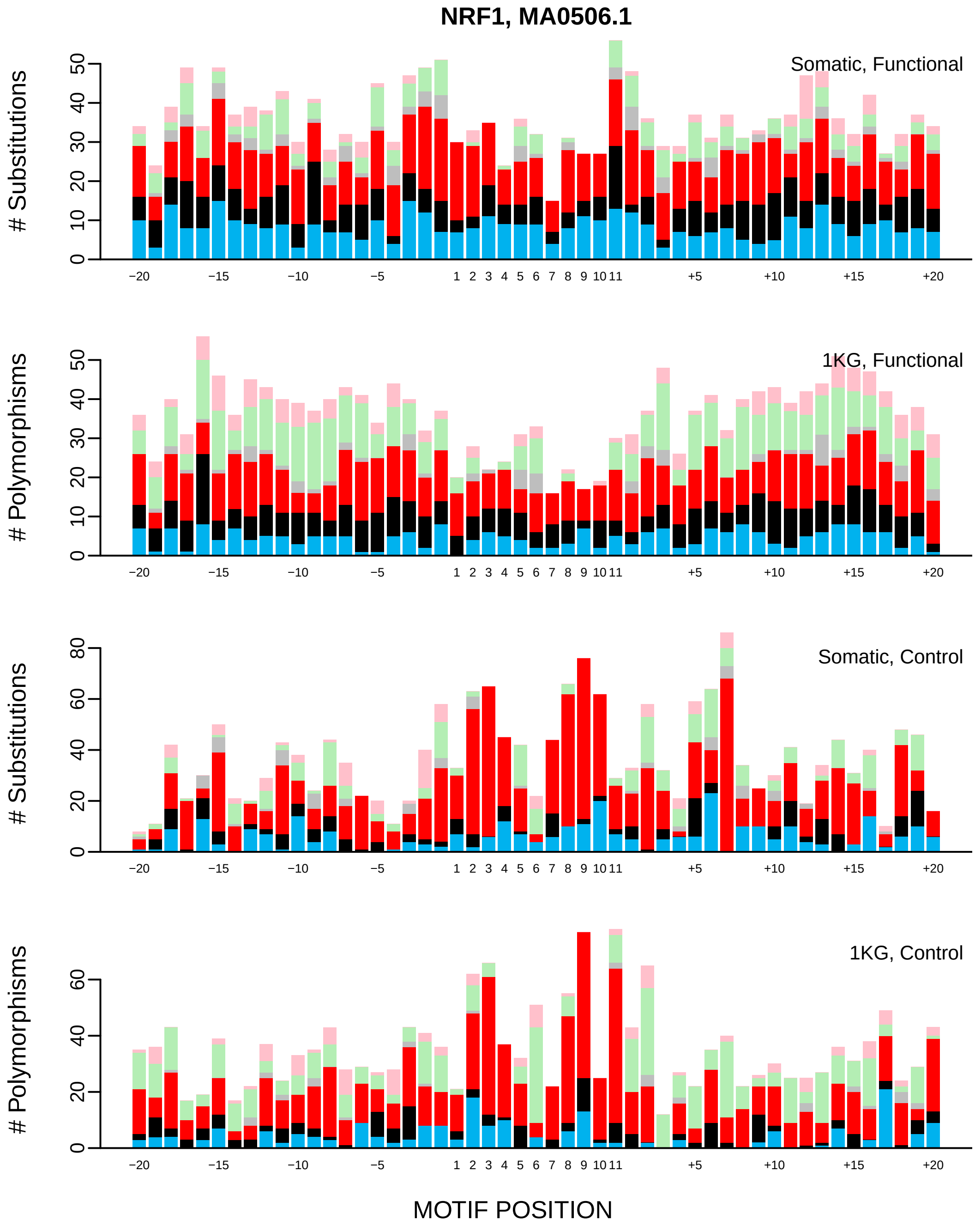
NRF1, MA0506.1
Somatic, Functional
Substitutions
1KG, Functional
Polymorphisms
Somatic, Control
Substitutions
1KG, Control
Polymorphisms
−20
−15
−10
−5
1 2 3 4 5 6 7 8 9 10 11
+5
+10
+15
+20
MOTIF POSITION

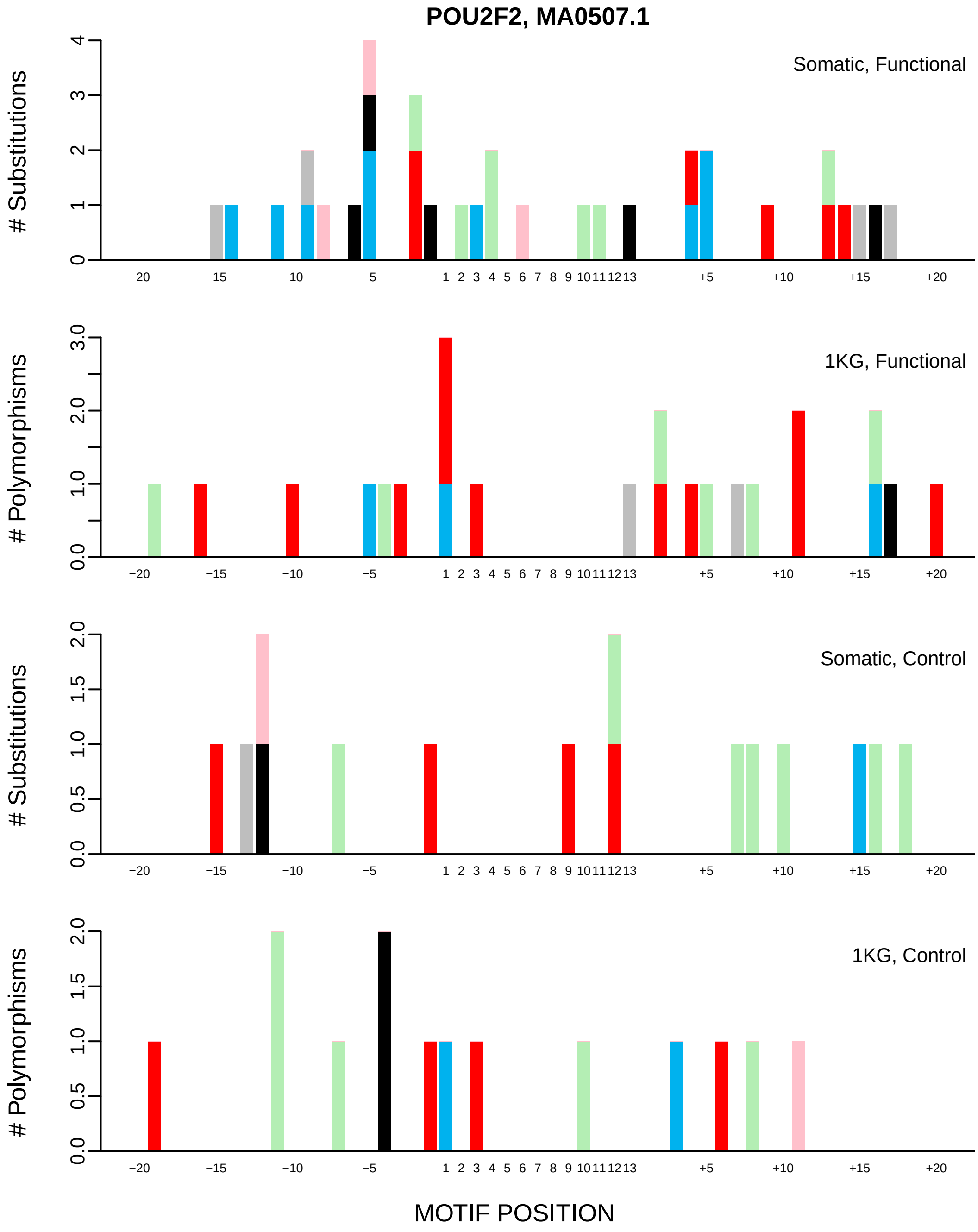

POU2F2, MA0507.1
Somatic, Functional
1KG, Functional
Somatic, Control
1KG, Control
Substitutions
Polymorphisms
MOTIF POSITION

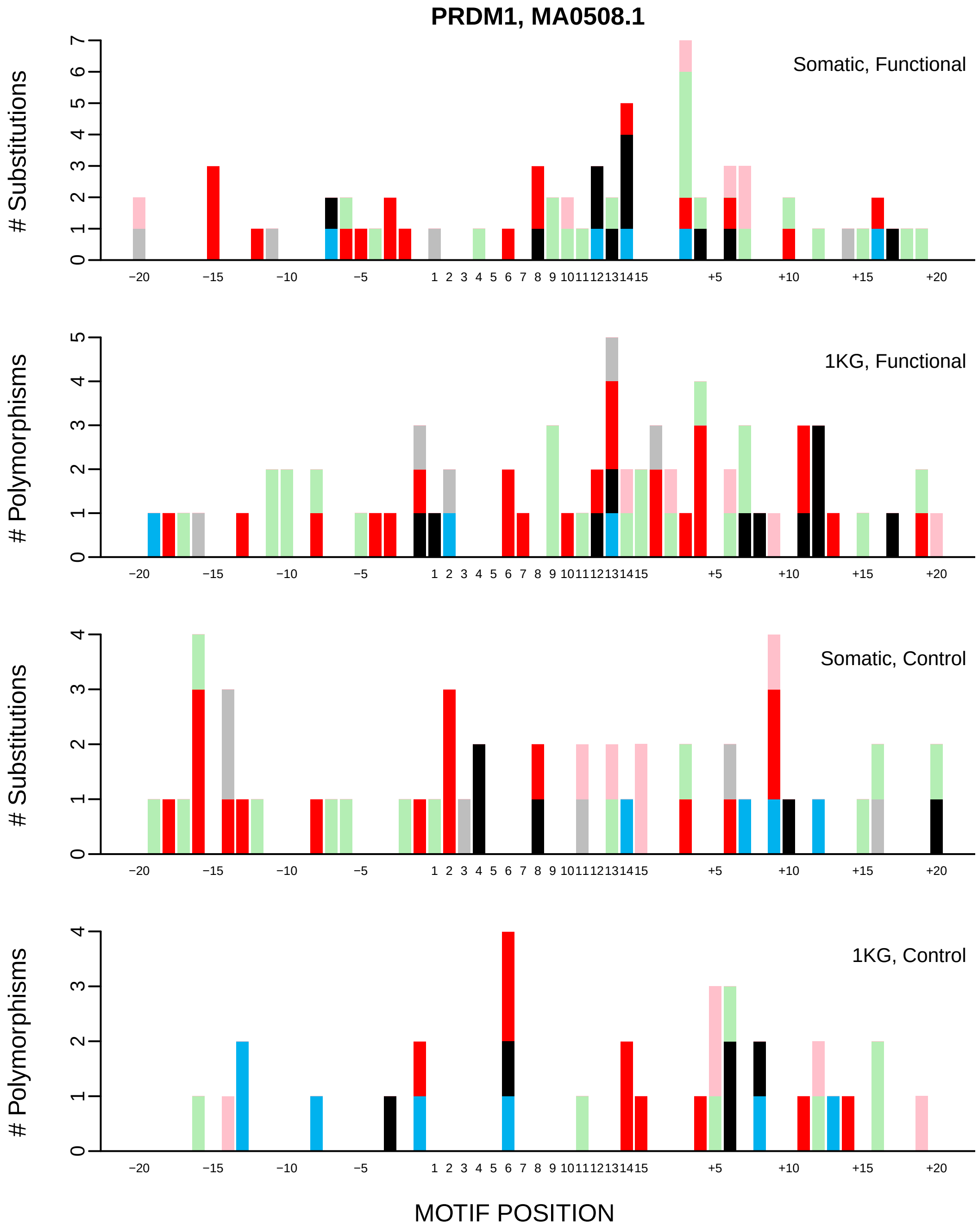
PRDM1, MA0508.1
Substitutions
Somatic, Functional
Polymorphisms
1KG, Functional
Substitutions
Somatic, Control
Polymorphisms
1KG, Control
−20 −15 −10 −5 1 2 3 4 5 6 7 8 9 10 11 12 13 14 15 +5 +10 +15 +20
MOTIF POSITION

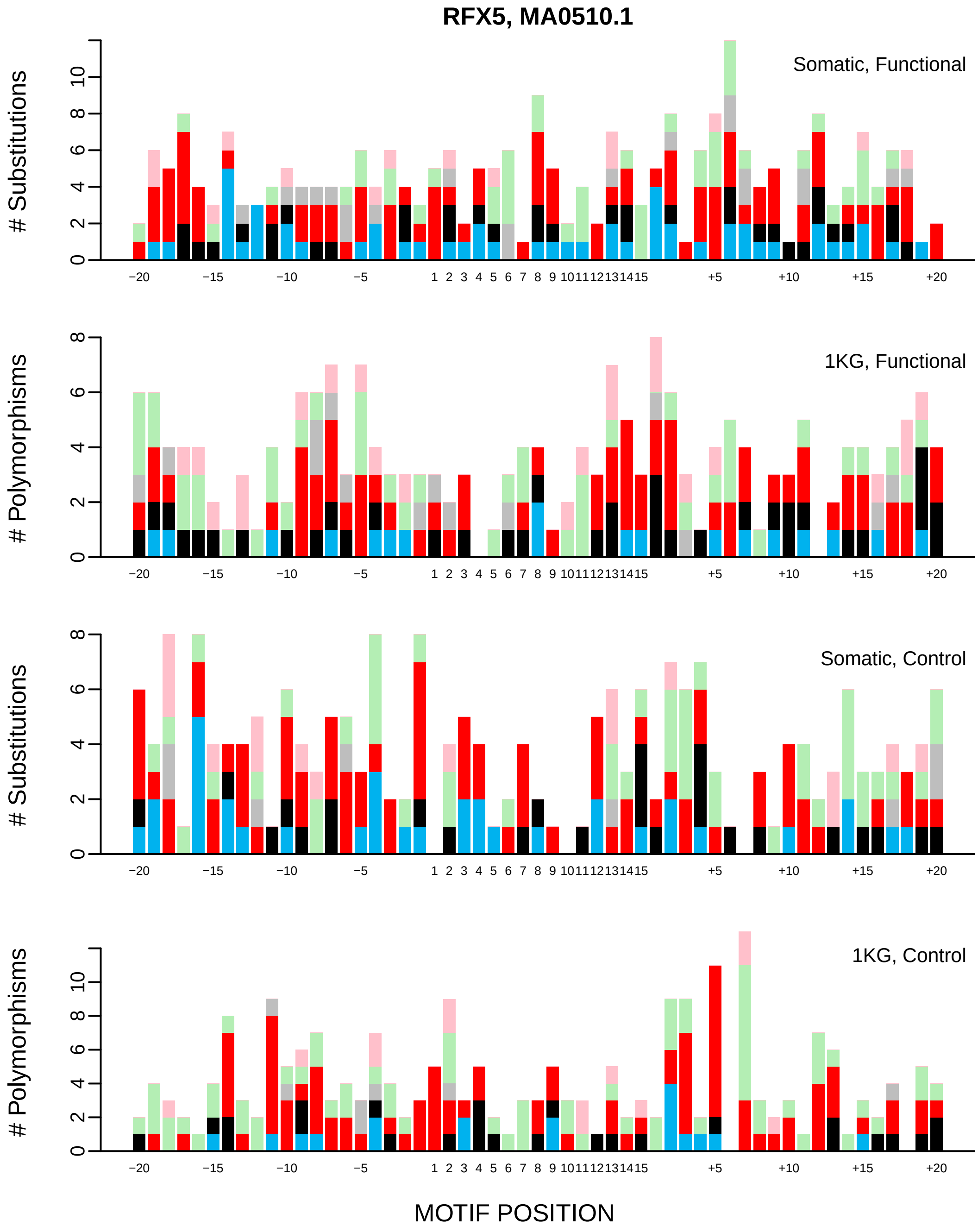
RFX5, MA0510.1
Substitutions
Somatic, Functional
Polymorphisms
1KG, Functional
Substitutions
Somatic, Control
Polymorphisms
1KG, Control
−20 −15 −10 −5 1 2 3 4 5 6 7 8 9 10 11 12 13 14 15 +5 +10 +15 +20
MOTIF POSITION

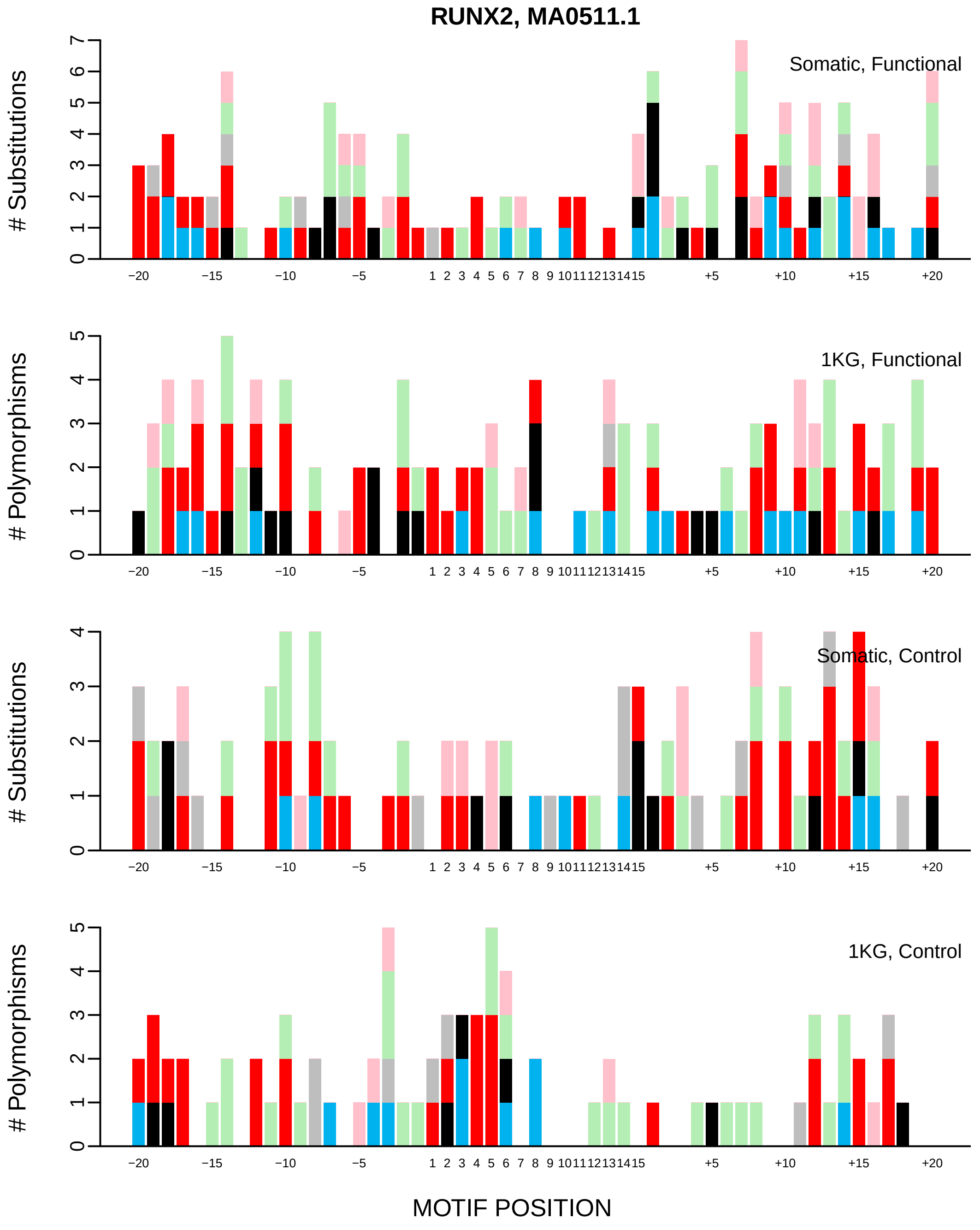
RUNX2, MA0511.1
Substitutions
Somatic, Functional
Polymorphisms
1KG, Functional
Substitutions
Somatic, Control
Polymorphisms
1KG, Control
−20 −15 −10 −5 1 2 3 4 5 6 7 8 9 10 11 12 13 14 15 +5 +10 +15 +20
MOTIF POSITION

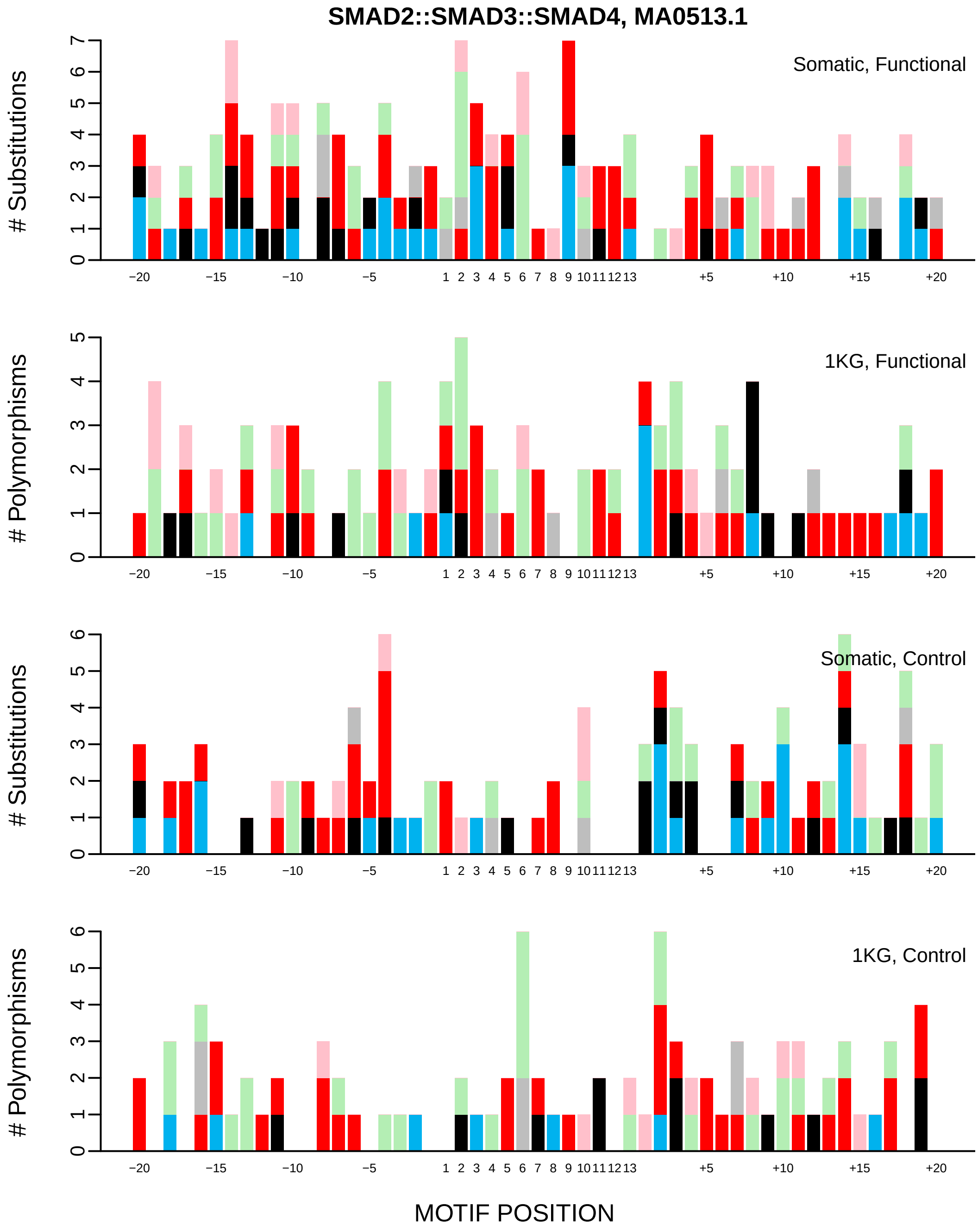
SMAD2::SMAD3::SMAD4, MA0513.1
Somatic, Functional
1KG, Functional
Somatic, Control
1KG, Control
Substitutions
Polymorphisms
−20
−15
−10
−5
1 2 3 4 5 6 7 8 9 10 11 12 13
+5
+10
+15
+20
MOTIF POSITION

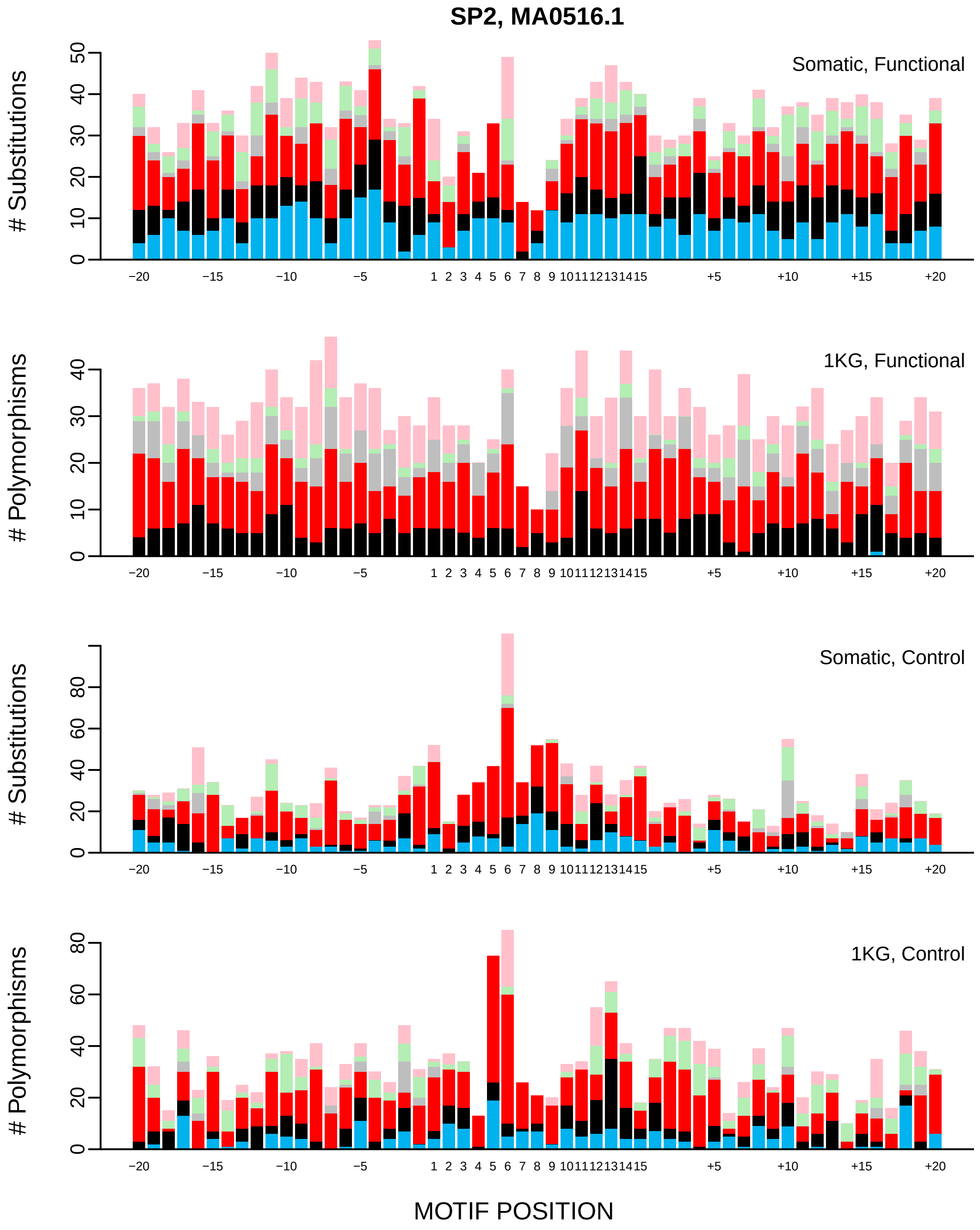

SP2, MA0516.1
Substitutions
Somatic, Functional
Polymorphisms
1KG, Functional
Substitutions
Somatic, Control
Polymorphisms
1KG, Control
−20 −15 −10 −5 1 2 3 4 5 6 7 8 9 10 11 12 13 14 15 +5 +10 +15 +20
MOTIF POSITION

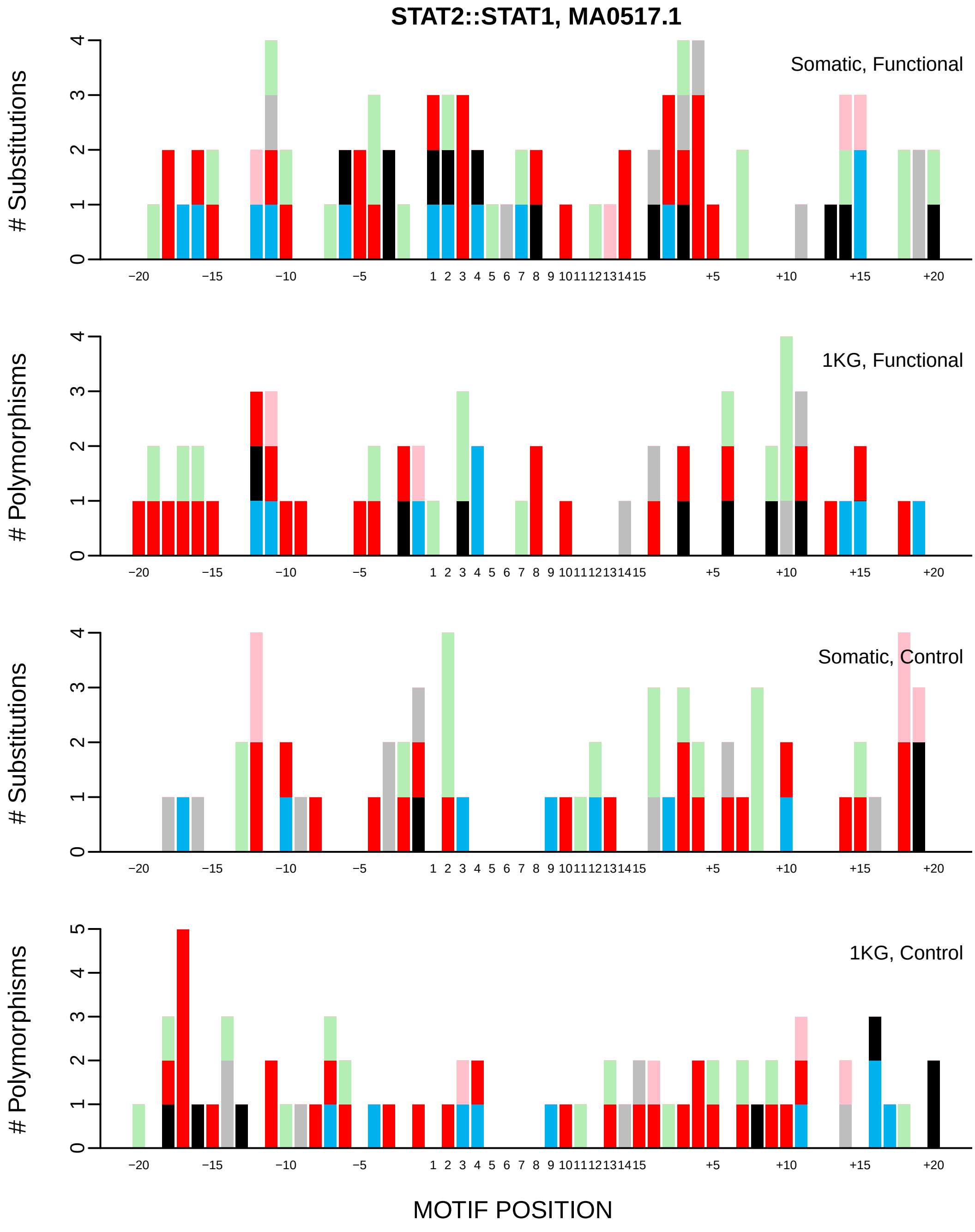
STAT2::STAT1, MA0517.1
Somatic, Functional
1KG, Functional
Somatic, Control
1KG, Control
Substitutions
Polymorphisms
Substitutions
Polymorphisms
−20
−15
−10
−5
1 2 3 4 5 6 7 8 9 10 11 12 13 14 15
+5
+10
+15
+20
MOTIF POSITION

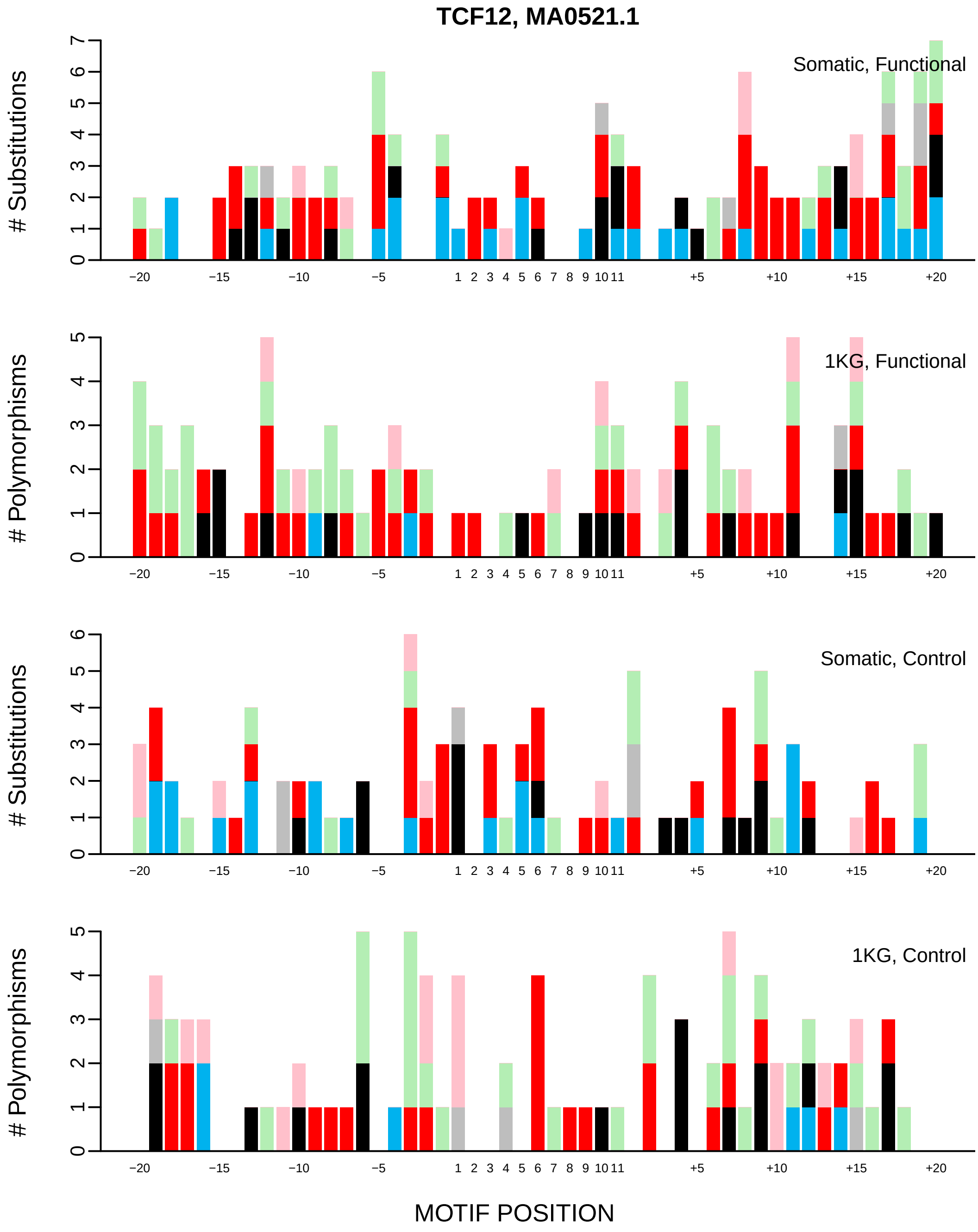
TCF12, MA0521.1
Substitutions
Somatic, Functional
Polymorphisms
1KG, Functional
Substitutions
Somatic, Control
Polymorphisms
1KG, Control
−20
−15
−10
−5
1 2 3 4 5 6 7 8 9 10 11
+5
+10
+15
+20
MOTIF POSITION

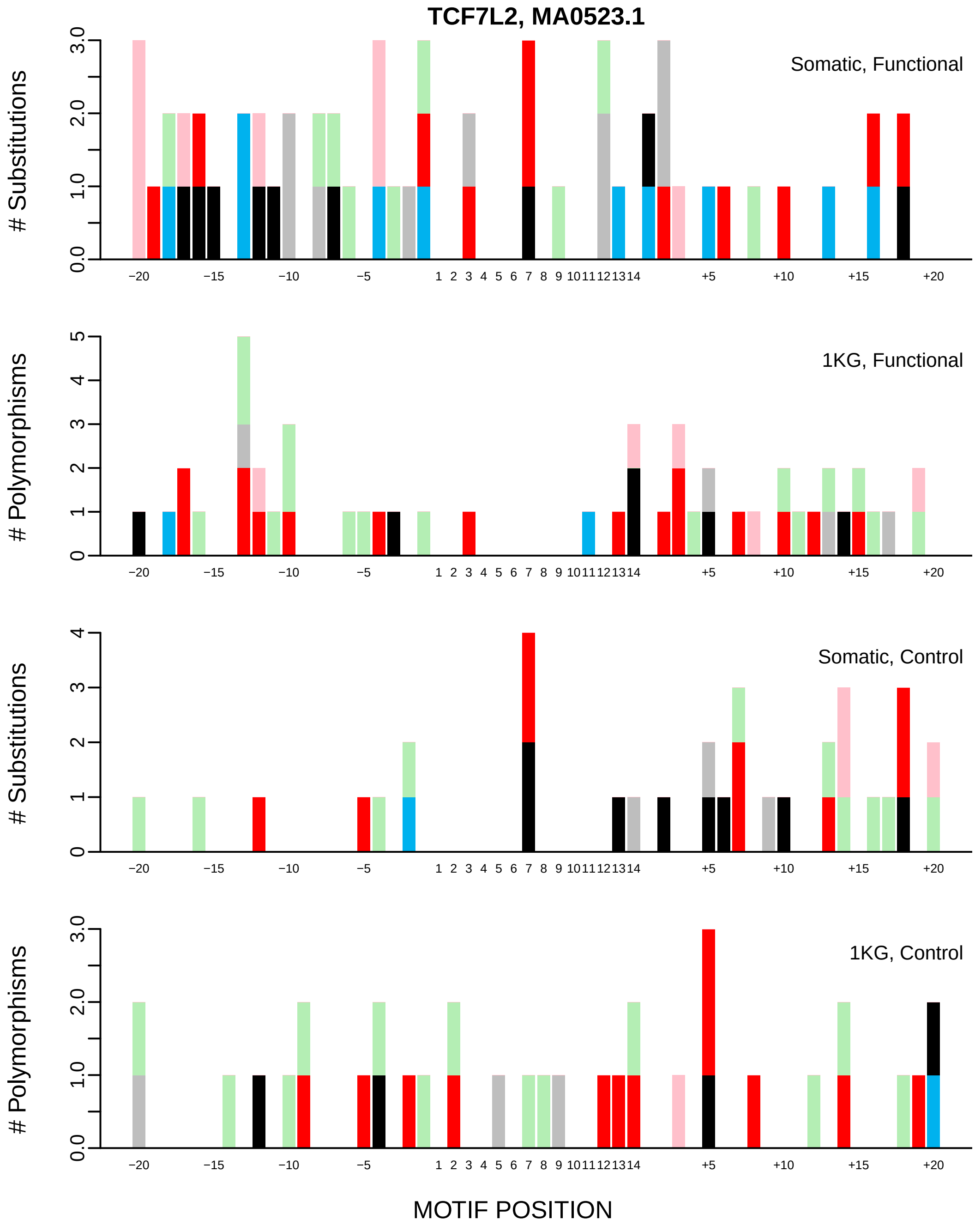
TCF7L2, MA0523.1
Substitutions
Somatic, Functional
Polymorphisms
1KG, Functional
Substitutions
Somatic, Control
Polymorphisms
1KG, Control
−20 −15 −10 −5 1 2 3 4 5 6 7 8 9 10 11 12 13 14 +5 +10 +15 +20
MOTIF POSITION

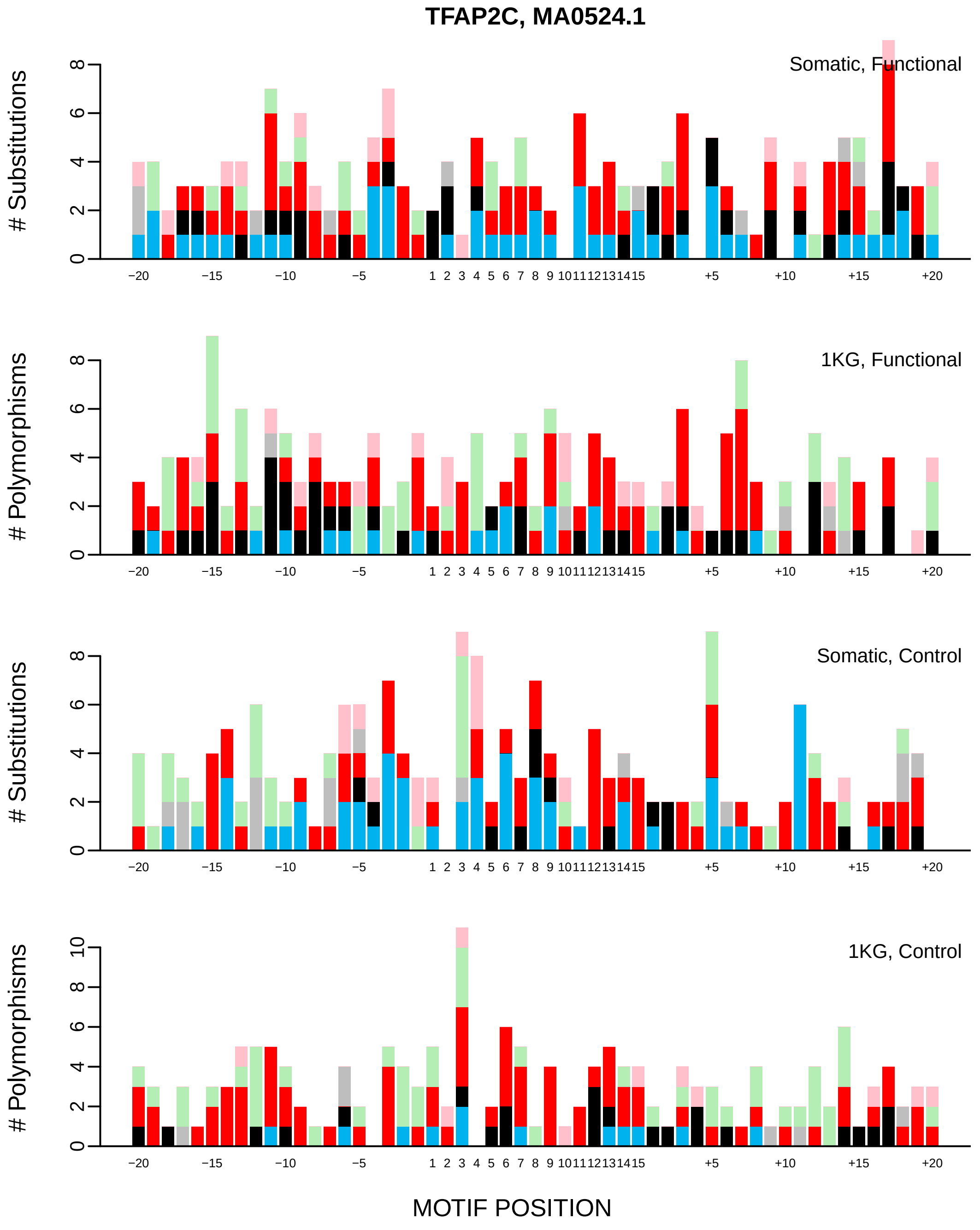
TFAP2C, MA0524.1
Substitutions
Somatic, Functional
Polymorphisms
1KG, Functional
Substitutions
Somatic, Control
Polymorphisms
1KG, Control
−20
−15
−10
−5
1 2 3 4 5 6 7 8 9 10 11 12 13 14 15
+5
+10
+15
+20
MOTIF POSITION

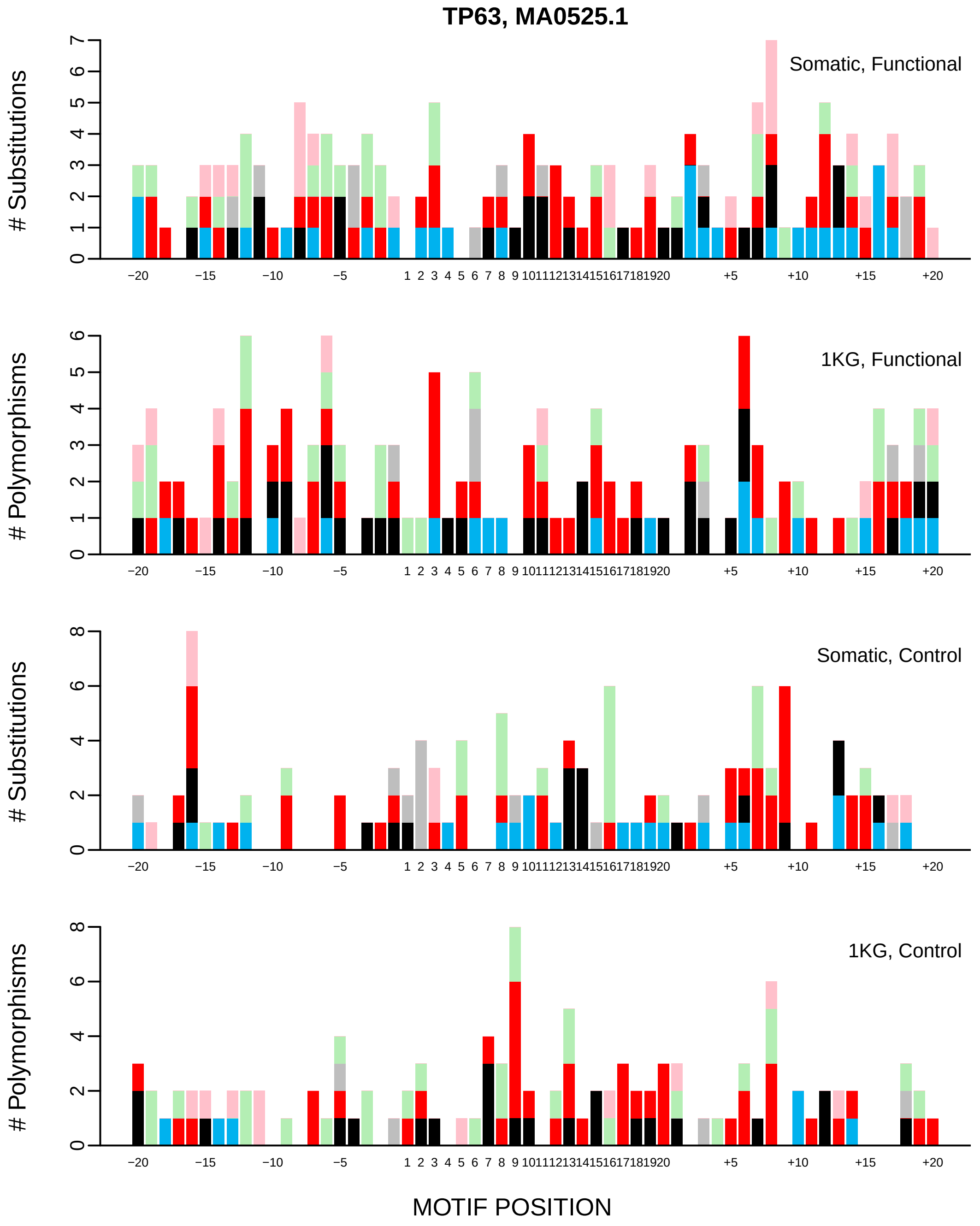
TP63, MA0525.1
Substitutions
Somatic, Functional
Polymorphisms
1KG, Functional
Substitutions
Somatic, Control
Polymorphisms
1KG, Control
−20
−15
−10
−5
1 2 3 4 5 6 7 8 9 10 11 12 13 14 15 16 17 18 19 20
+5
+10
+15
+20
MOTIF POSITION

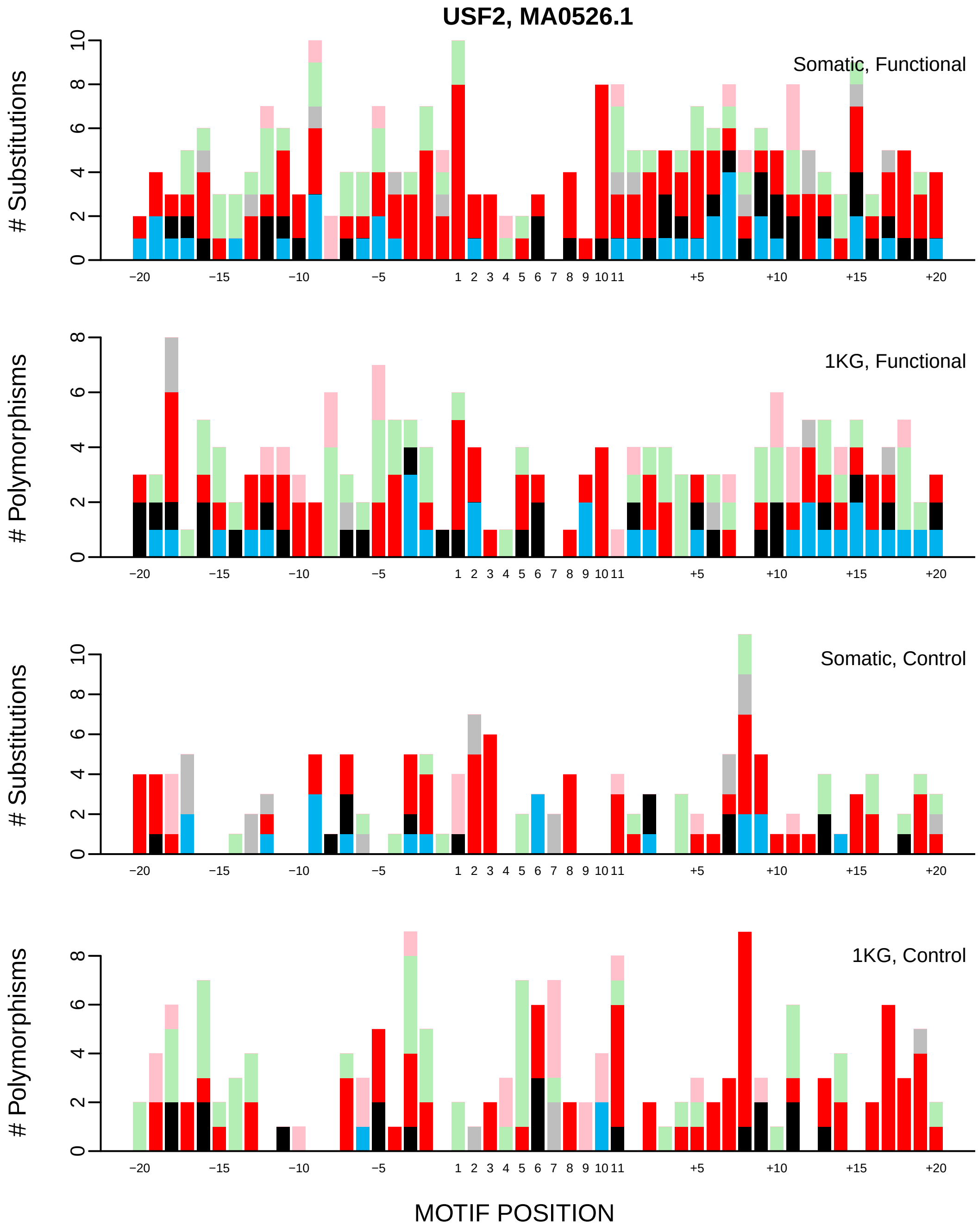

USF2, MA0526.1
Substitutions
Somatic, Functional
Polymorphisms
1KG, Functional
Substitutions
Somatic, Control
Polymorphisms
1KG, Control
MOTIF POSITION

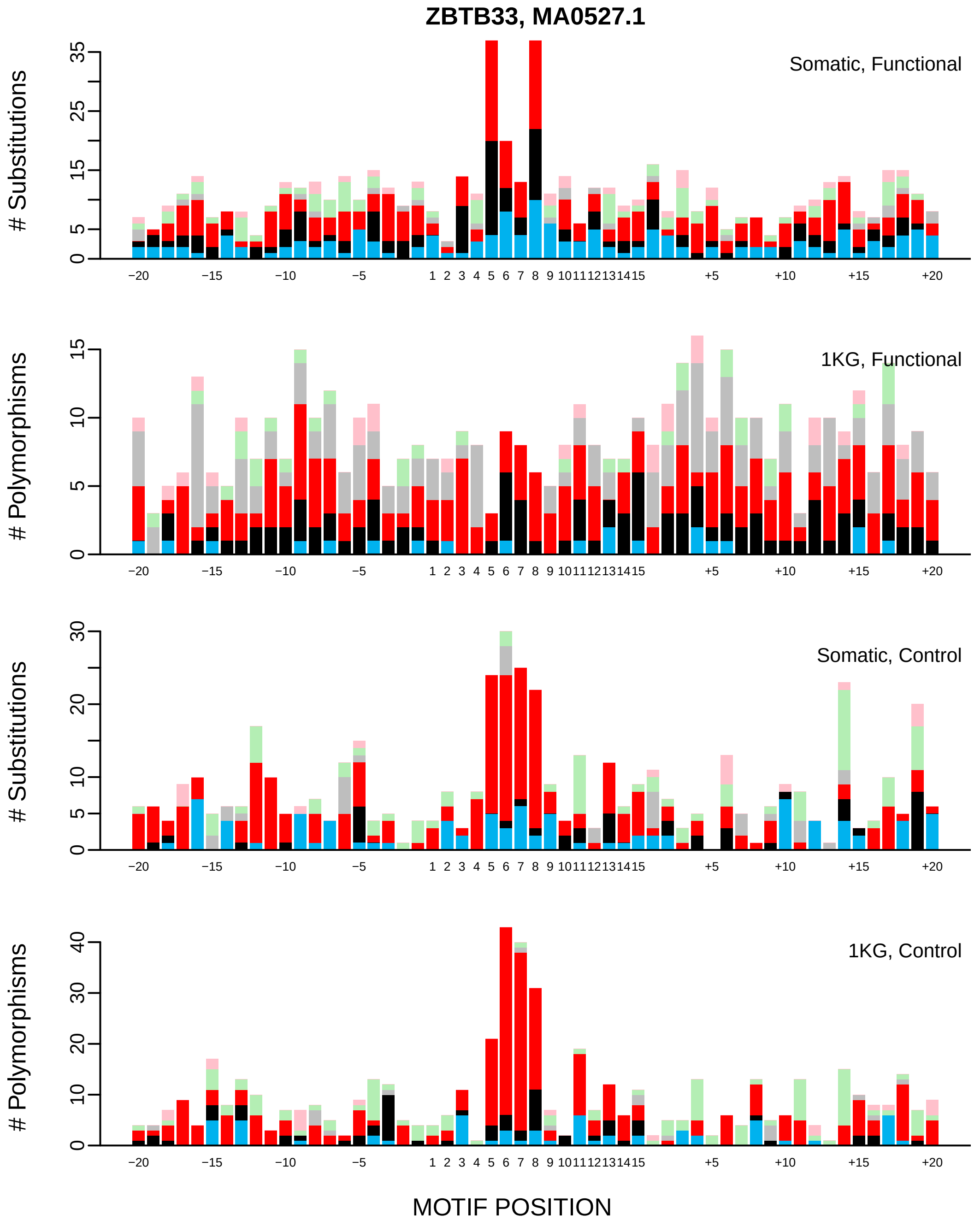
ZBTB33, MA0527.1
Somatic, Functional
1KG, Functional
Somatic, Control
1KG, Control
Substitutions
Polymorphisms
−20 −15 −10 −5 1 2 3 4 5 6 7 8 9 10 11 12 13 14 15 +5 +10 +15 +20
MOTIF POSITION

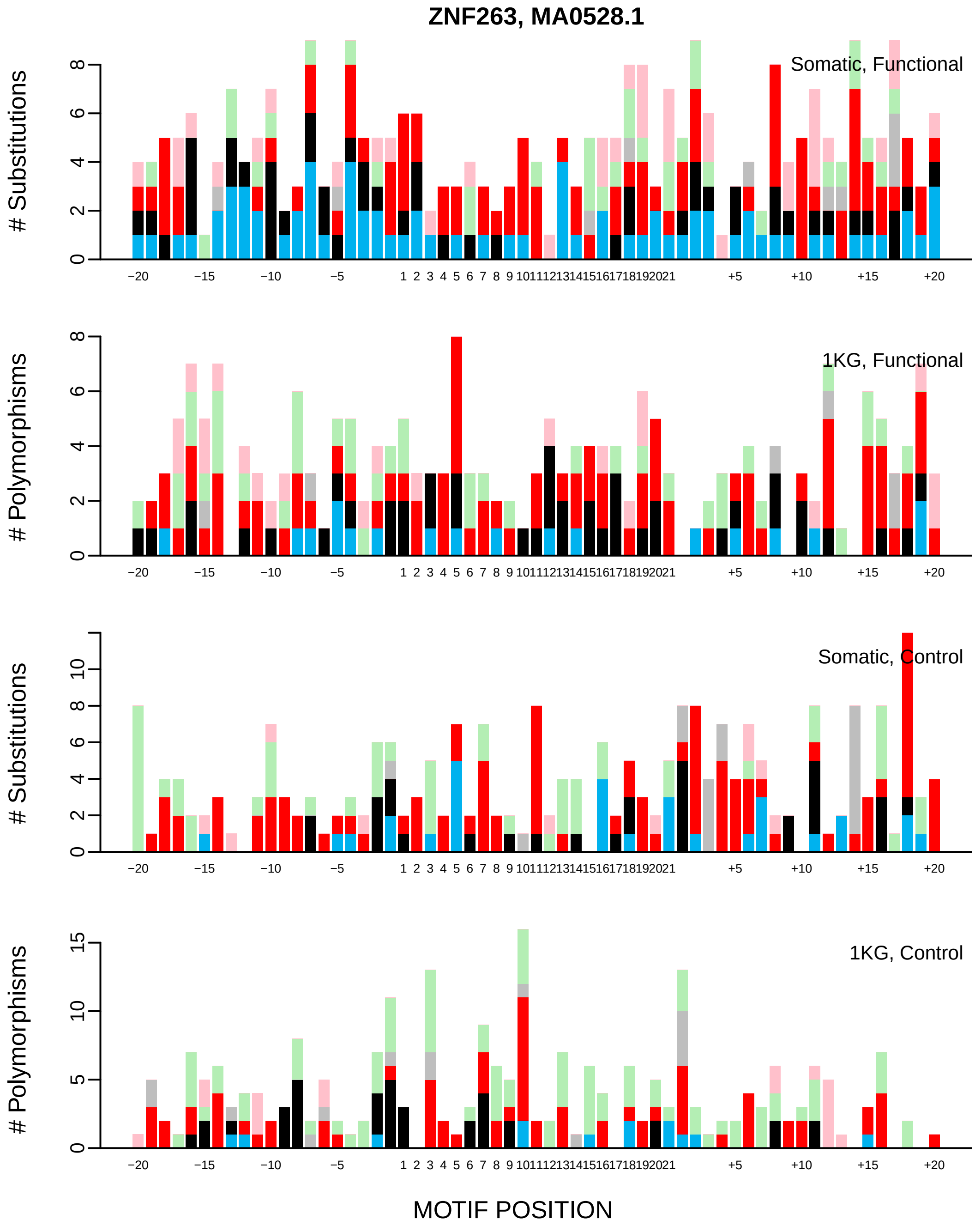
ZNF263, MA0528.1
Somatic, Functional
1KG, Functional
Somatic, Control
1KG, Control
Substitutions
Polymorphisms
−20
−15
−10
−5
1 2 3 4 5 6 7 8 9 10 11 12 13 14 15 16 17 18 19 20 21
+5
+10
+15
+20
MOTIF POSITION

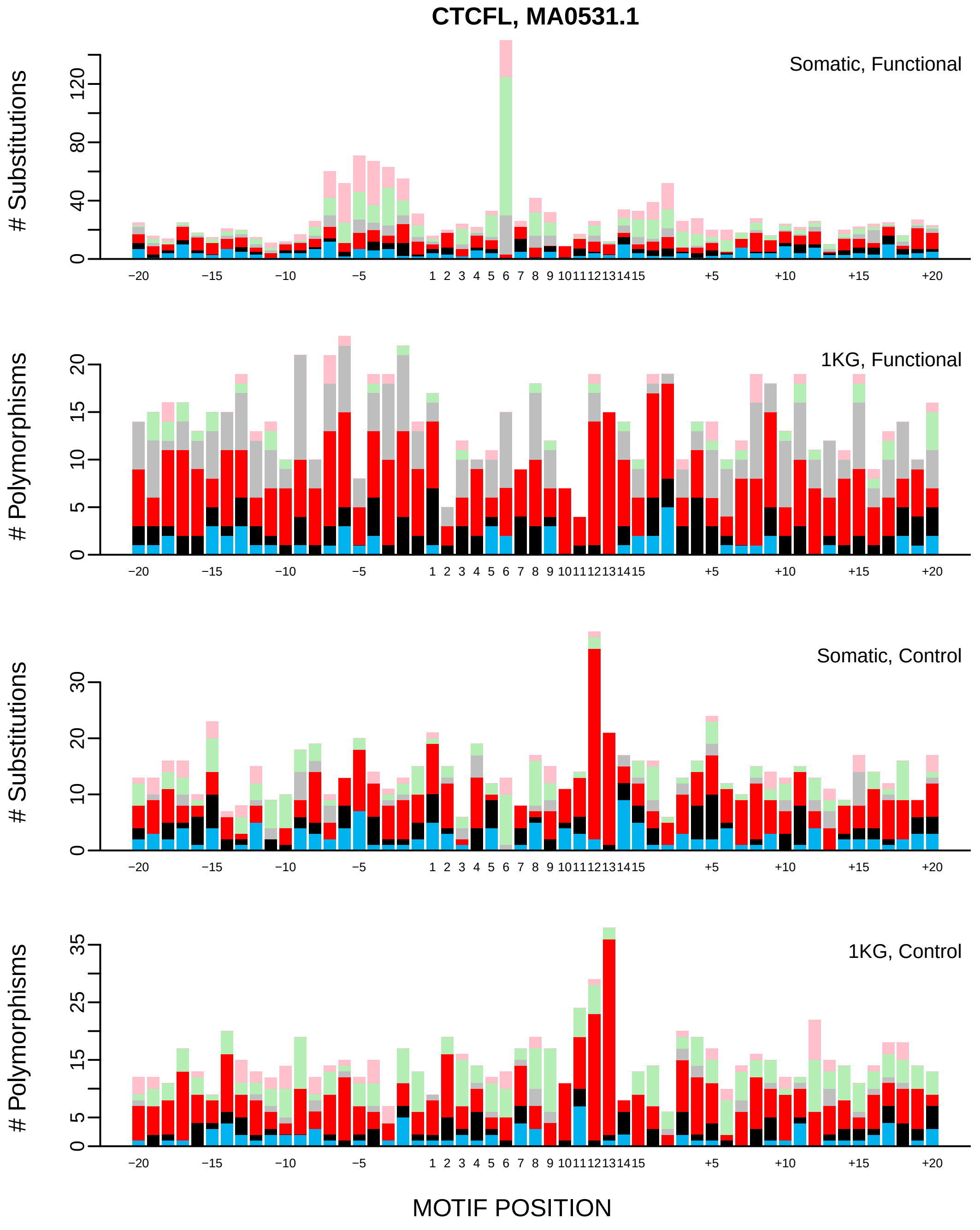
CTCFL, MA0531.1
Somatic, Functional
1KG, Functional
Somatic, Control
1KG, Control
Substitutions
Polymorphisms
−20
−15
−10
−5
1 2 3 4 5 6 7 8 9 10 11 12 13 14 15
+5
+10
+15
+20
MOTIF POSITION

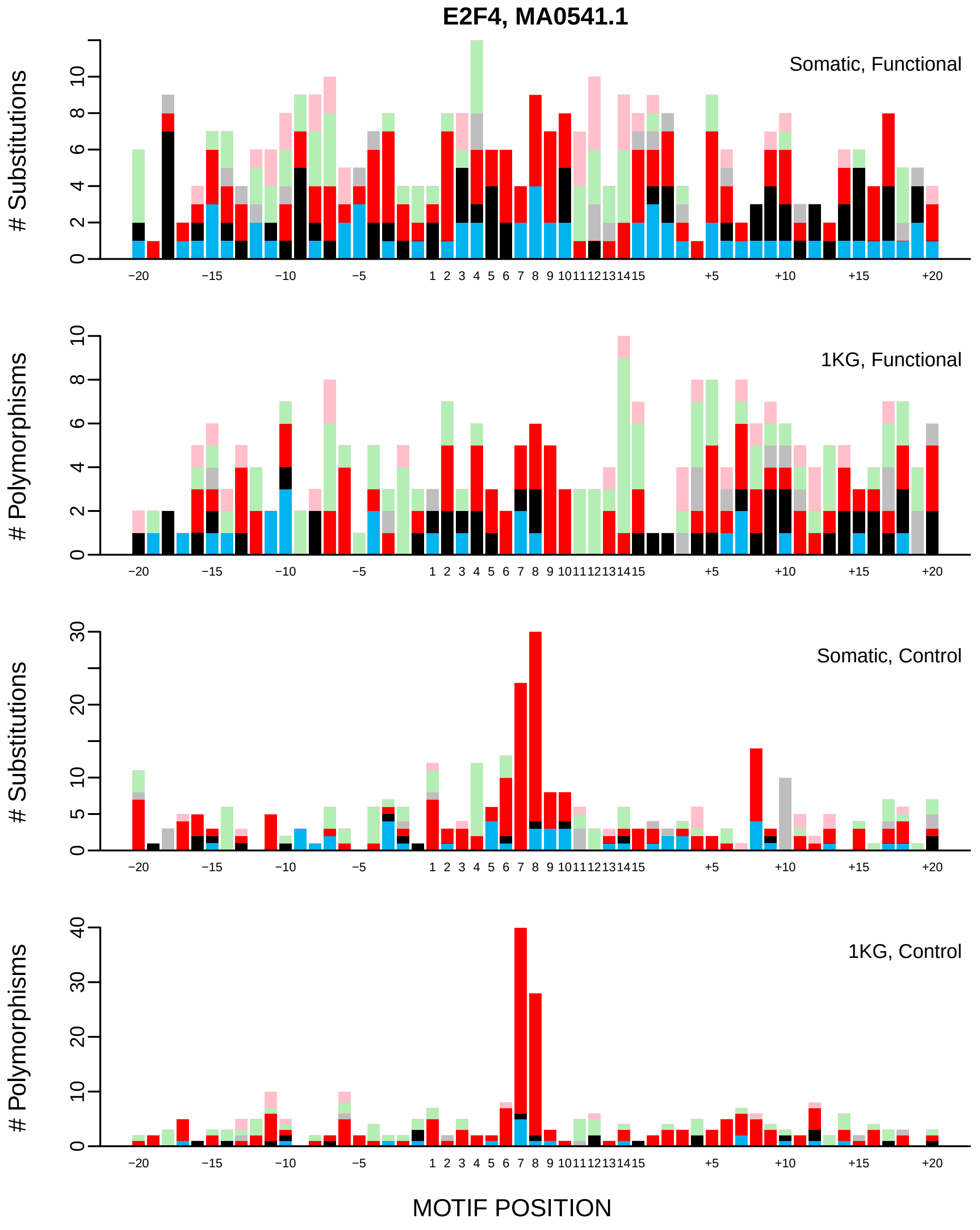
E2F4, MA0541.1
Somatic, Functional
1KG, Functional
Somatic, Control
1KG, Control
Substitutions
Polymorphisms
−20
−15
−10
−5
1 2 3 4 5 6 7 8 9 10 11 12 13 14 15
+5
+10
+15
+20
MOTIF POSITION

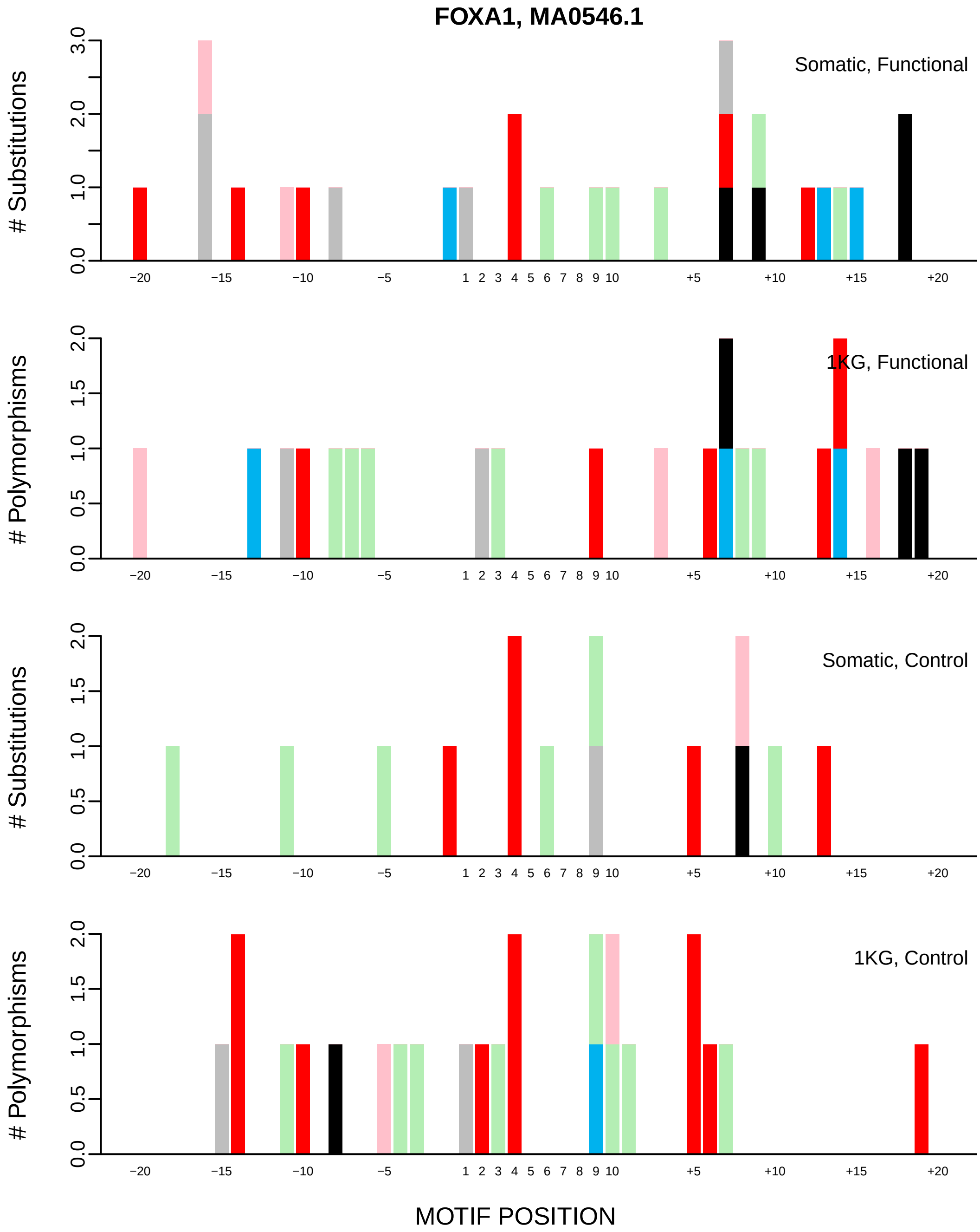
FOXA1, MA0546.1
Substitutions
Somatic, Functional
Polymorphisms
1KG, Functional
Substitutions
Somatic, Control
Polymorphisms
1KG, Control
MOTIF POSITION

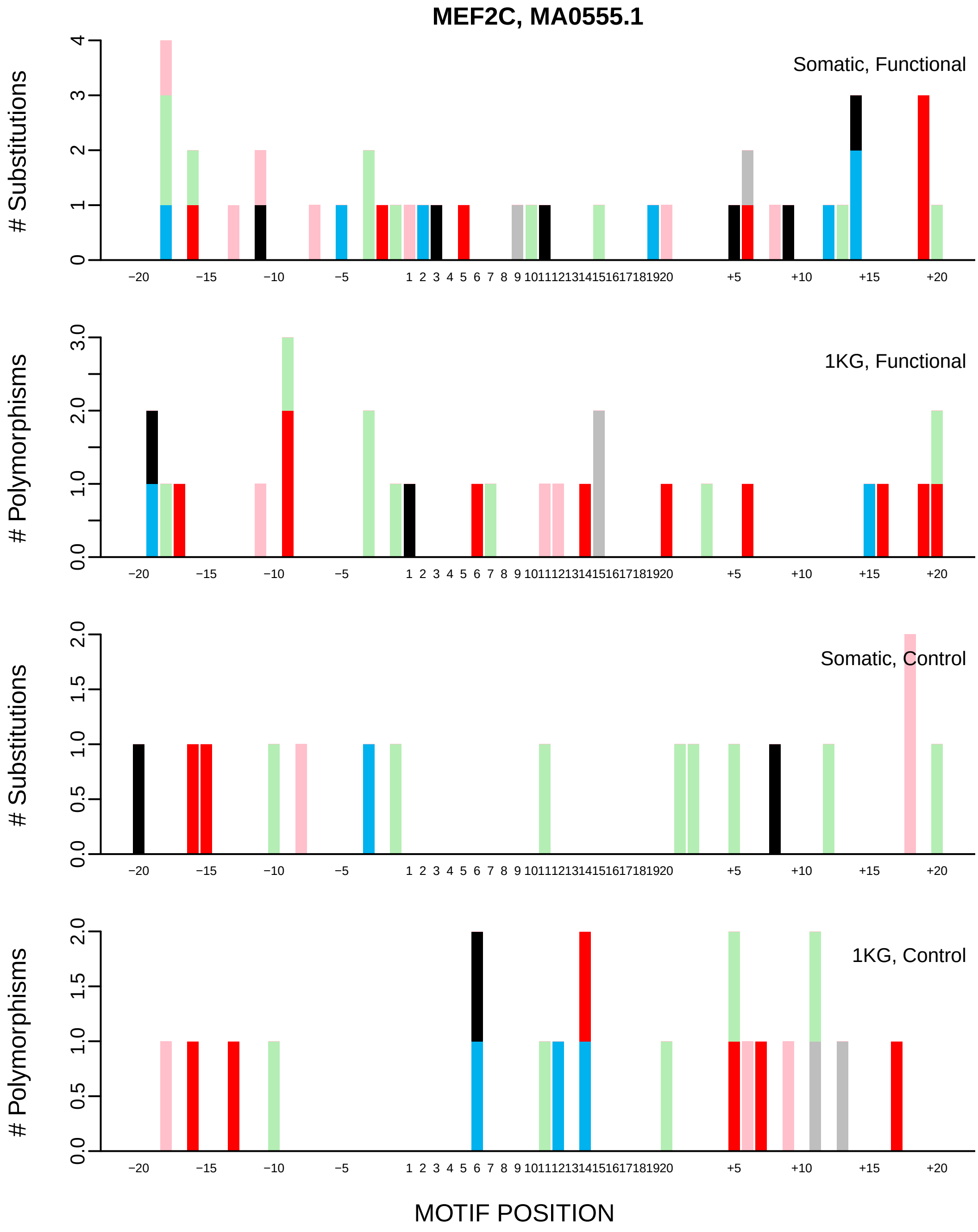

MEF2C, MA0555.1
Substitutions
Somatic, Functional
Polymorphisms
1KG, Functional
Substitutions
Somatic, Control
Polymorphisms
1KG, Control
−20 −15 −10 −5 1 2 3 4 5 6 7 8 9 10 11 12 13 14 15 16 17 18 19 20 +5 +10 +15 +20
MOTIF POSITION

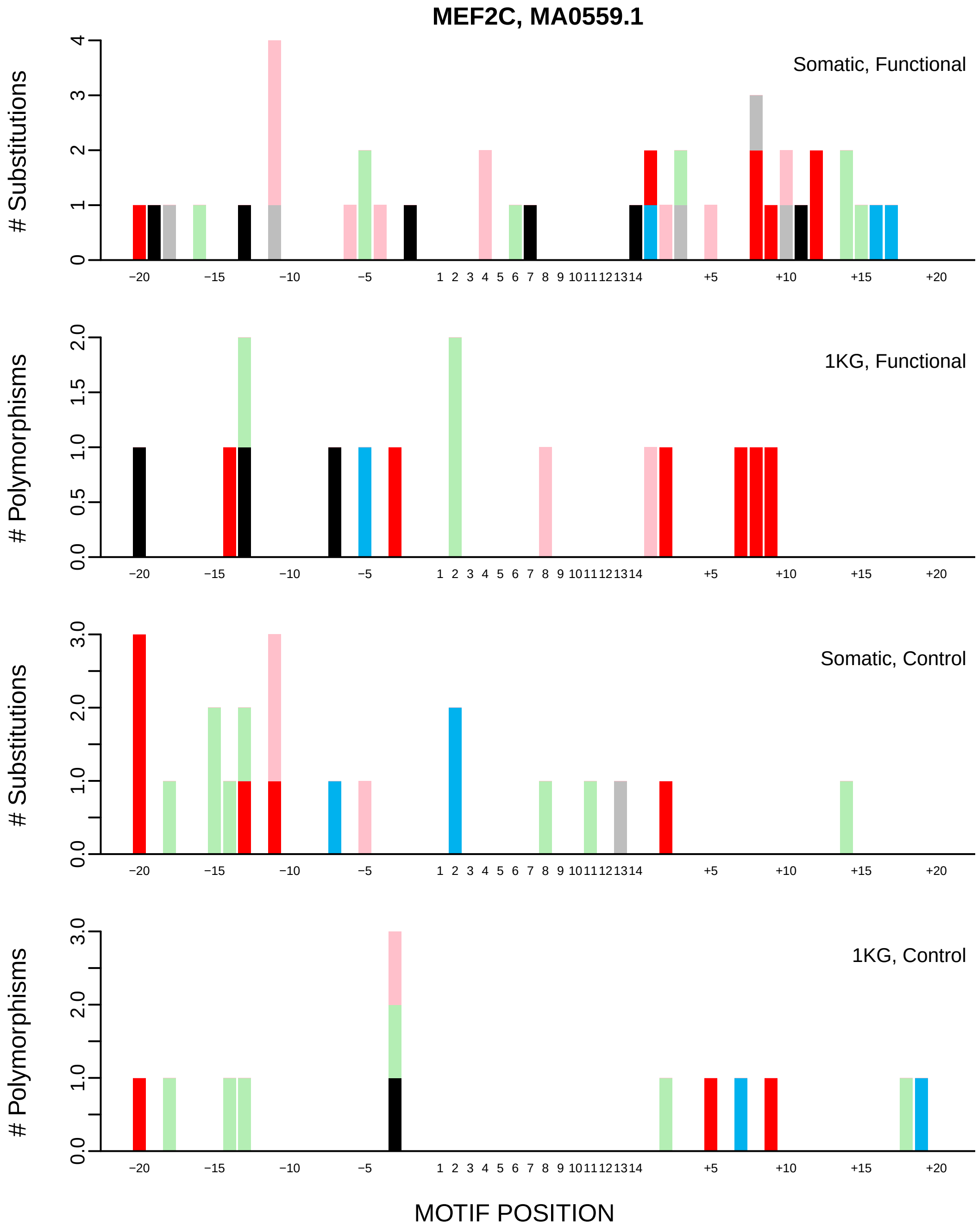

MEF2C, MA0559.1
Substitutions
Somatic, Functional
Polymorphisms
1KG, Functional
Substitutions
Somatic, Control
Polymorphisms
1KG, Control
−20
−15
−10
−5
1 2 3 4 5 6 7 8 9 10 11 12 13 14
+5
+10
+15
+20
MOTIF POSITION

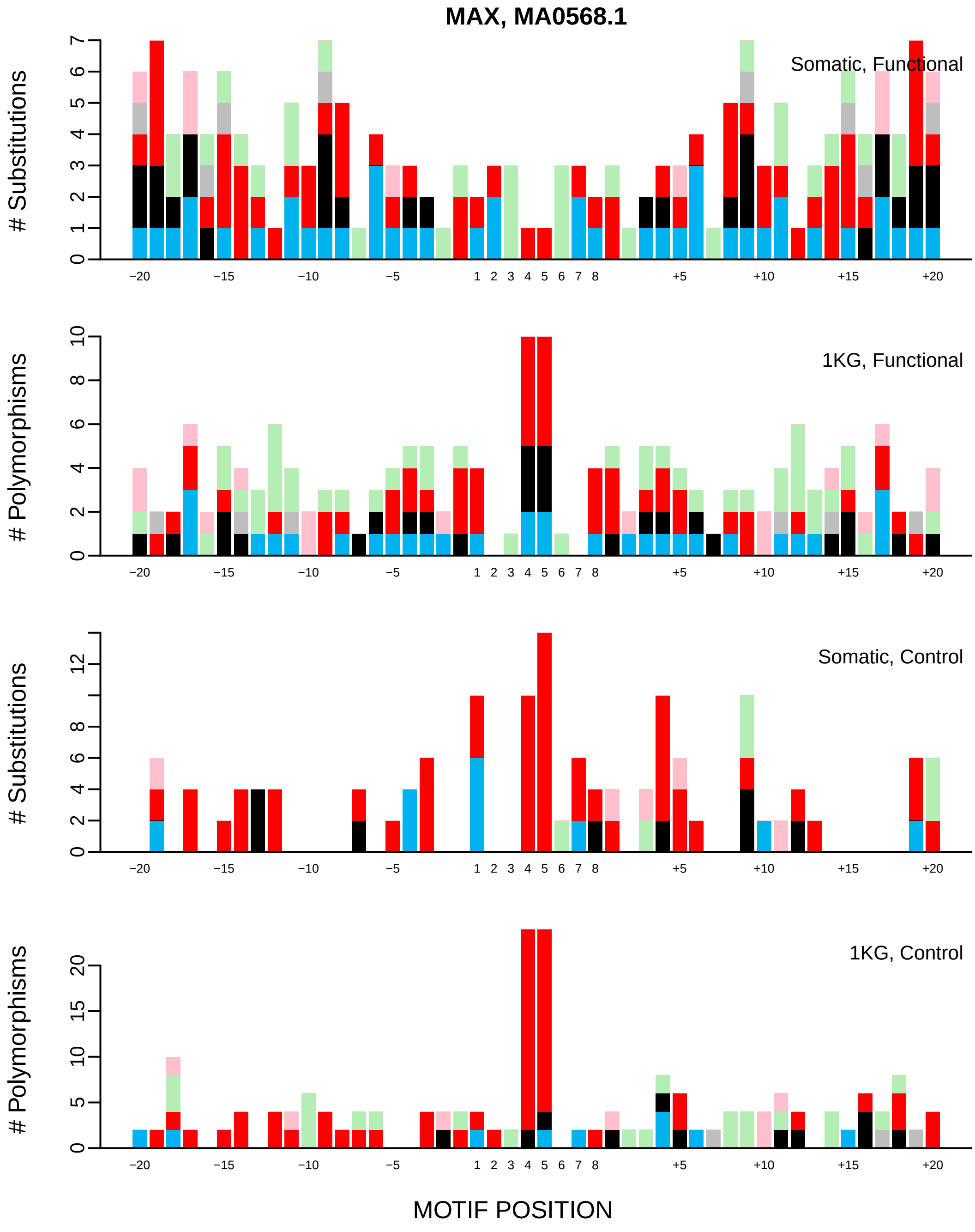

MAX, MA0568.1
Substitutions
Somatic, Functional
Polymorphisms
1KG, Functional
Substitutions
Somatic, Control
Polymorphisms
1KG, Control
−20
−15
−10
−5
1 2 3 4 5 6 7 8
+5
+10
+15
+20
MOTIF POSITION

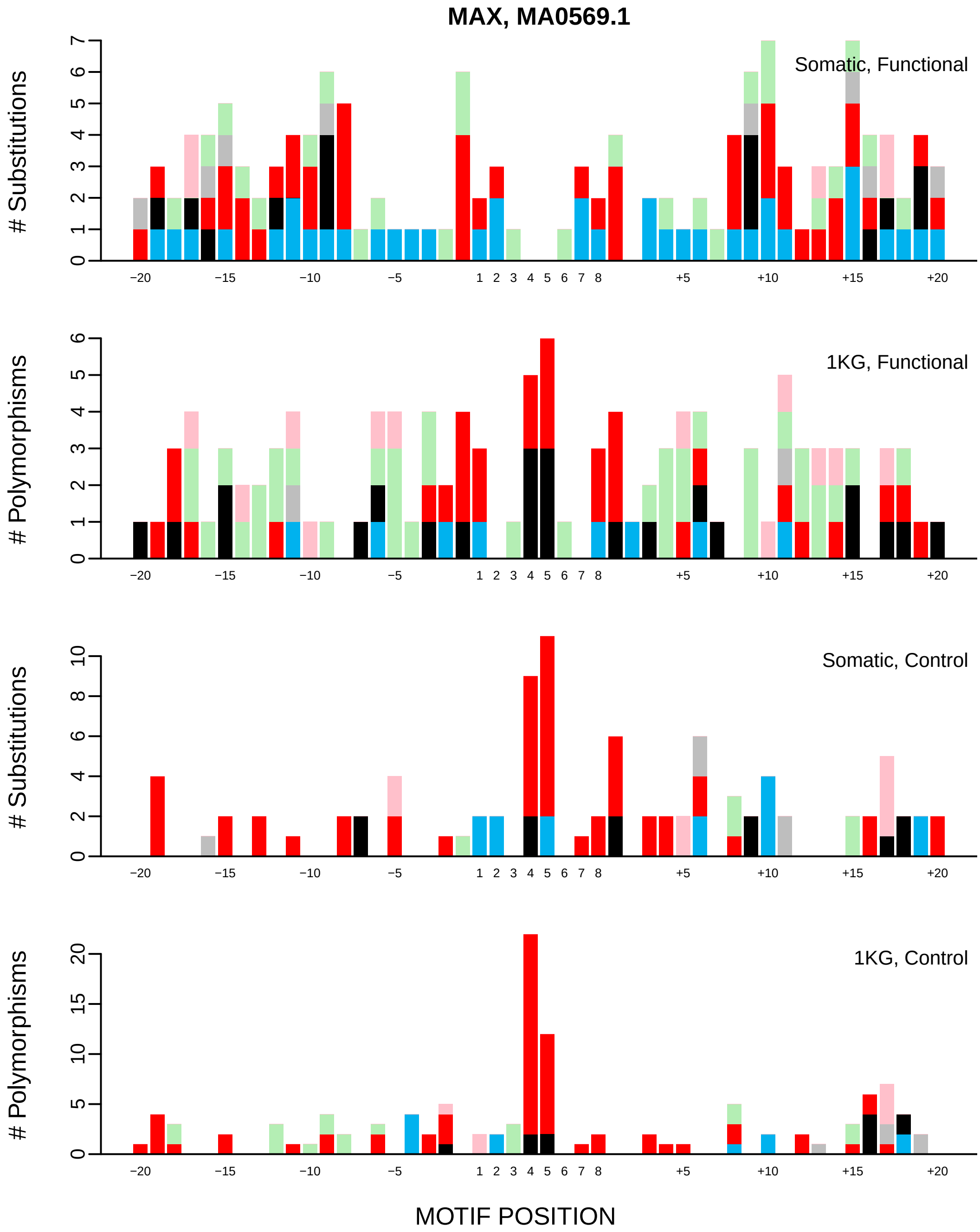
MAX, MA0569.1
Substitutions
Somatic, Functional
Polymorphisms
1KG, Functional
Substitutions
Somatic, Control
Polymorphisms
1KG, Control
−20
−15
−10
−5
1 2 3 4 5 6 7 8
+5
+10
+15
+20
MOTIF POSITION

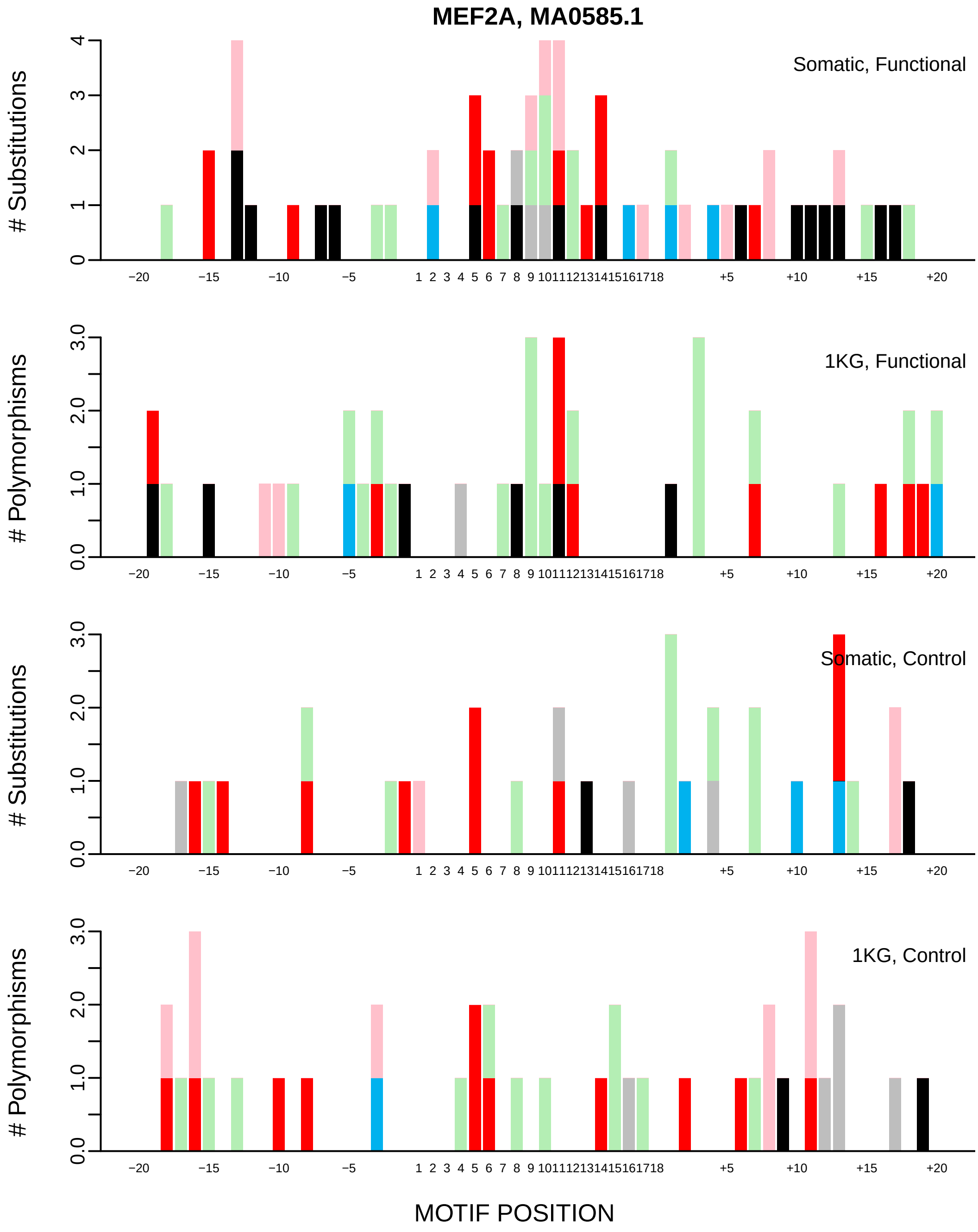
MEF2A, MA0585.1
Substitutions
Somatic, Functional
Polymorphisms
1KG, Functional
Substitutions
Somatic, Control
Polymorphisms
1KG, Control
MOTIF POSITION

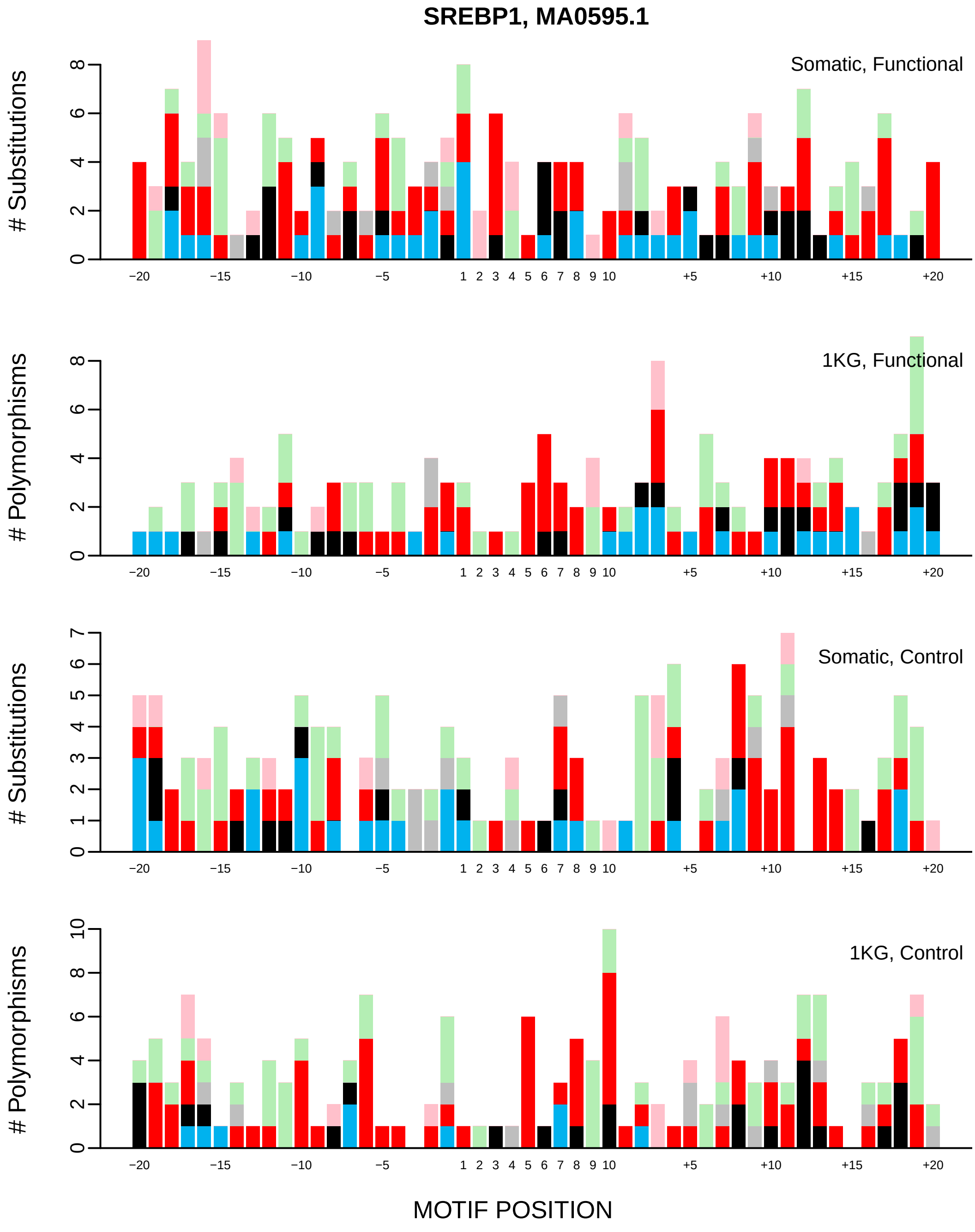
SREBP1, MA0595.1
Substitutions
Somatic, Functional
Polymorphisms
1KG, Functional
Substitutions
Somatic, Control
Polymorphisms
1KG, Control
−20
−15
−10
−5
1 2 3 4 5 6 7 8 9 10
+5
+10
+15
+20
MOTIF POSITION

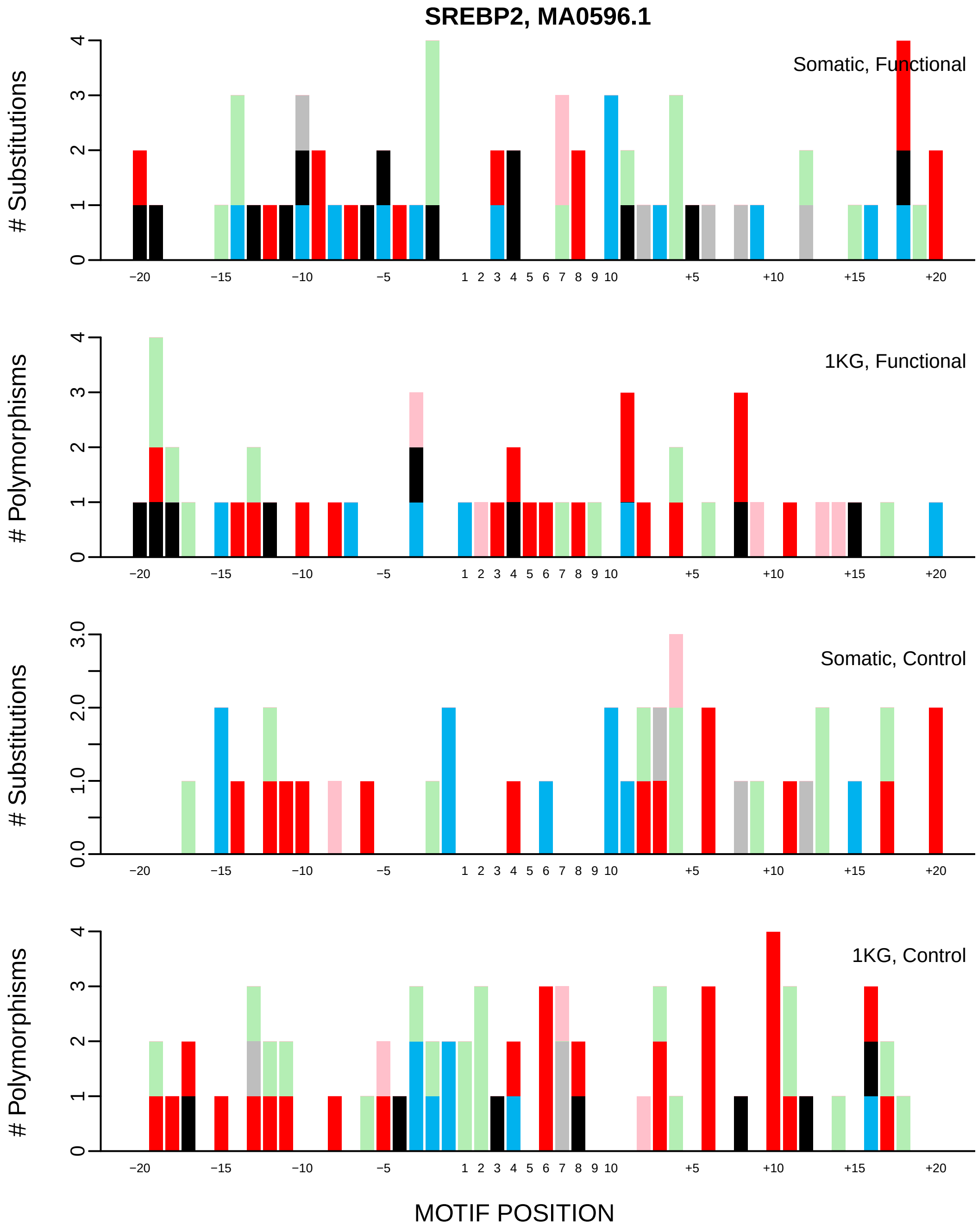
SREBP2, MA0596.1
Substitutions
Somatic, Functional
Polymorphisms
1KG, Functional
Substitutions
Somatic, Control
Polymorphisms
1KG, Control
−20
−15
−10
−5
1 2 3 4 5 6 7 8 9 10
+5
+10
+15
+20
MOTIF POSITION

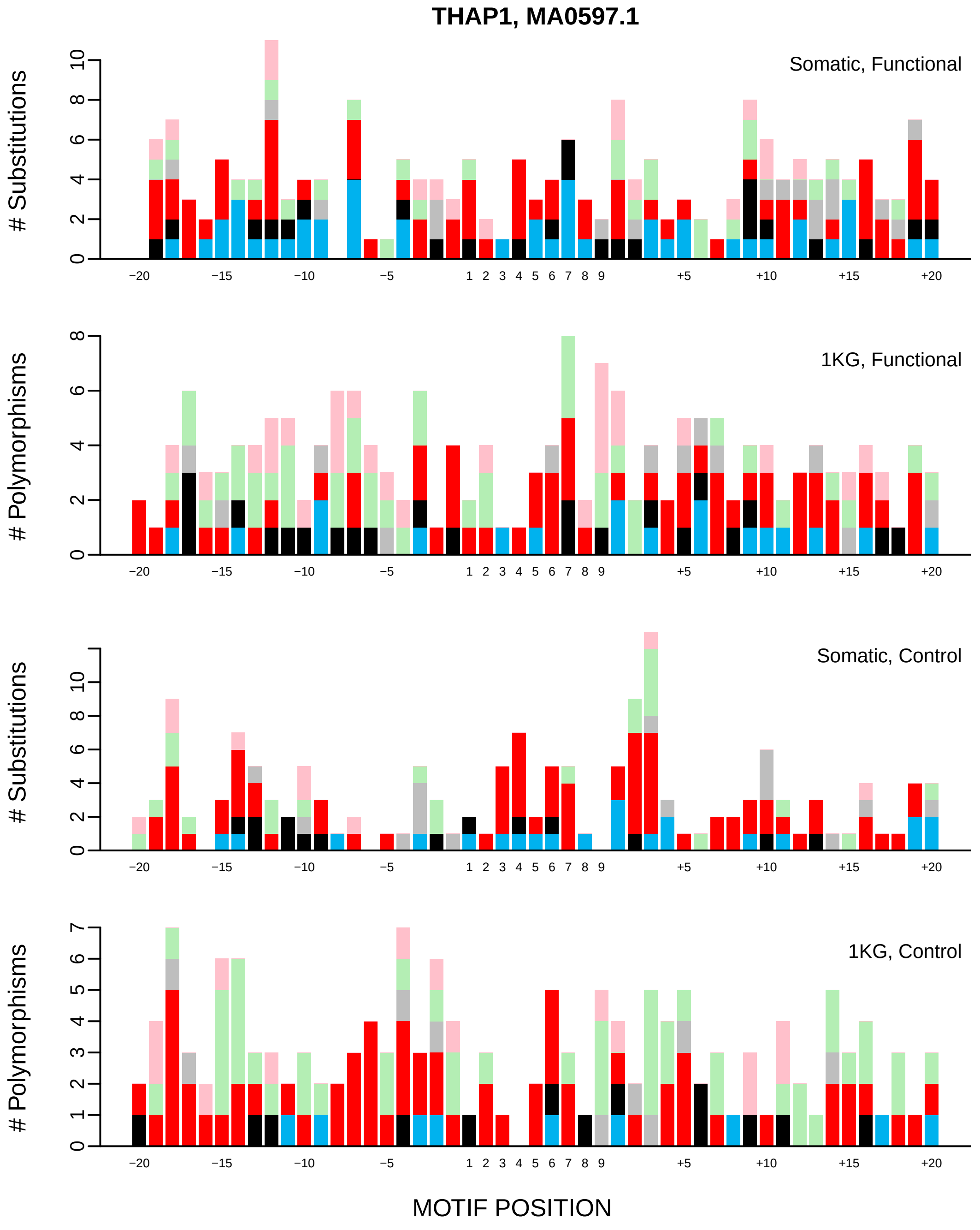
THAP1, MA0597.1
Somatic, Functional
1KG, Functional
Somatic, Control
1KG, Control
Substitutions
Polymorphisms
−20
−15
−10
−5
1 2 3 4 5 6 7 8 9
+5
+10
+15
+20
MOTIF POSITION

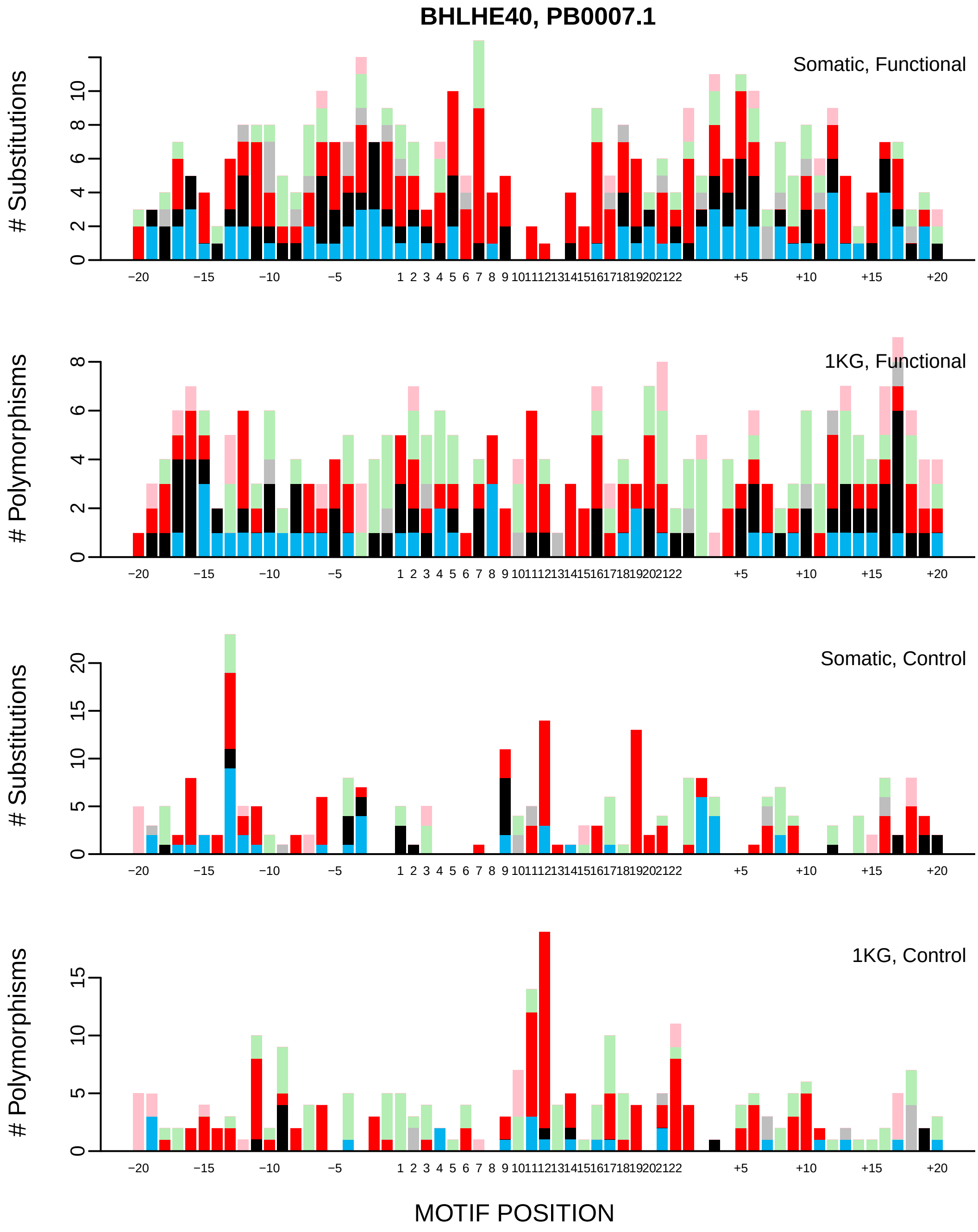
BHLHE40, PB0007.1
Somatic, Functional
1KG, Functional
Somatic, Control
1KG, Control
Substitutions
Polymorphisms
MOTIF POSITION

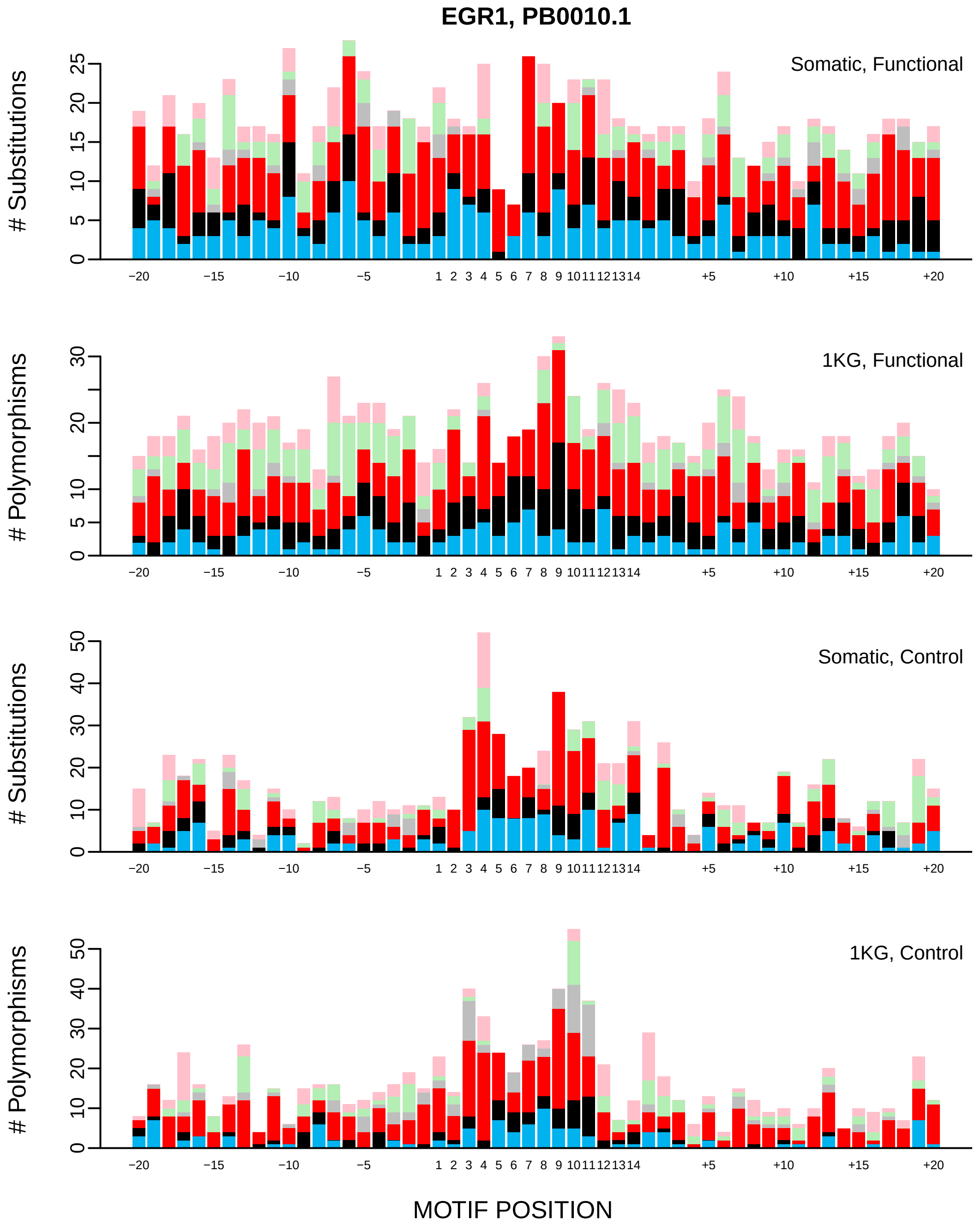

EGR1, PB0010.1
Substitutions
Somatic, Functional
Polymorphisms
1KG, Functional
Substitutions
Somatic, Control
Polymorphisms
1KG, Control
−20
−15
−10
−5
1 2 3 4 5 6 7 8 9 10 11 12 13 14
+5
+10
+15
+20
MOTIF POSITION

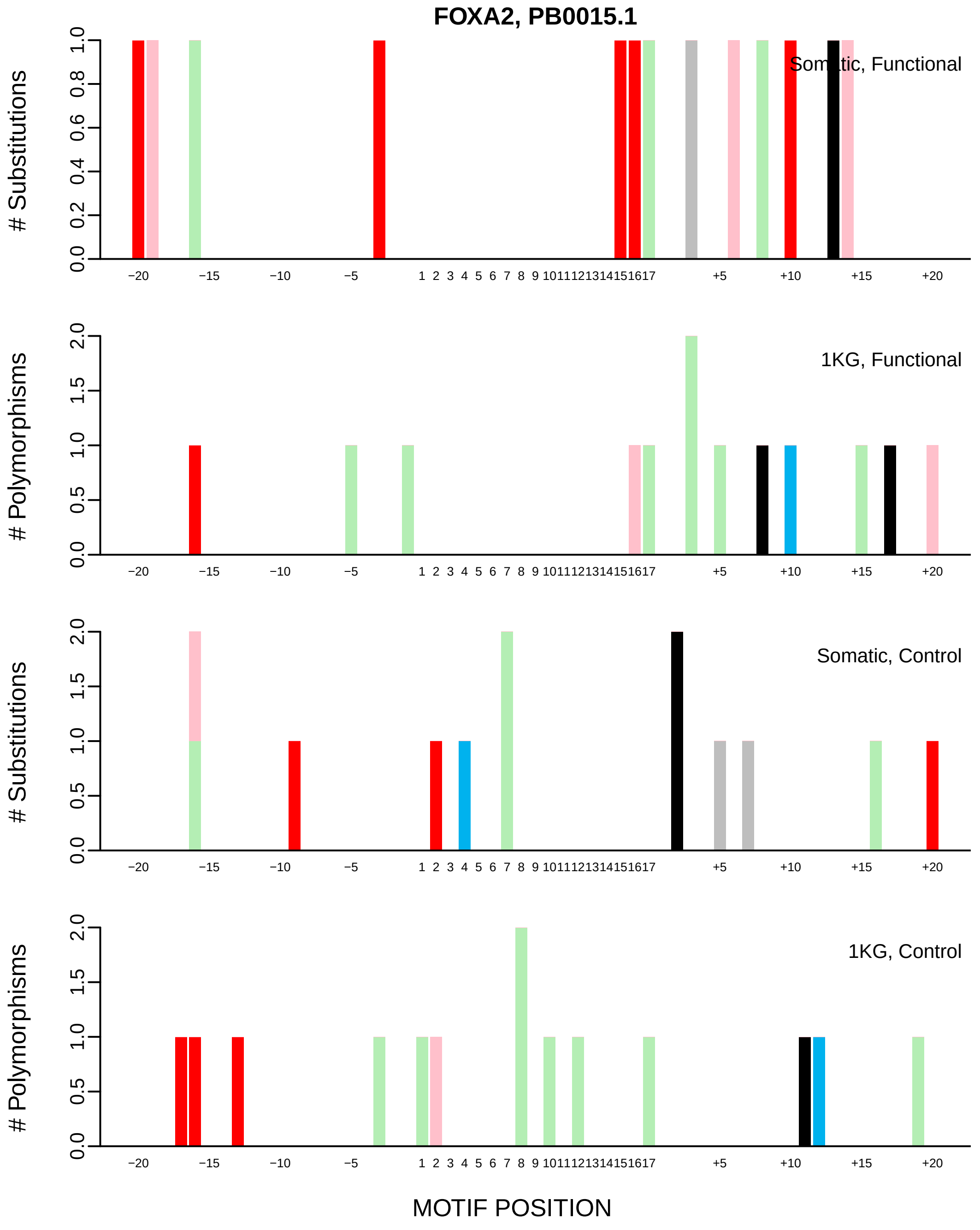
FOXA2, PB0015.1
Substitutions
Somatic, Functional
Polymorphisms
1KG, Functional
Substitutions
Somatic, Control
Polymorphisms
1KG, Control
−20 −15 −10 −5 1 2 3 4 5 6 7 8 9 10 11 12 13 14 15 16 17 +5 +10 +15 +20
MOTIF POSITION

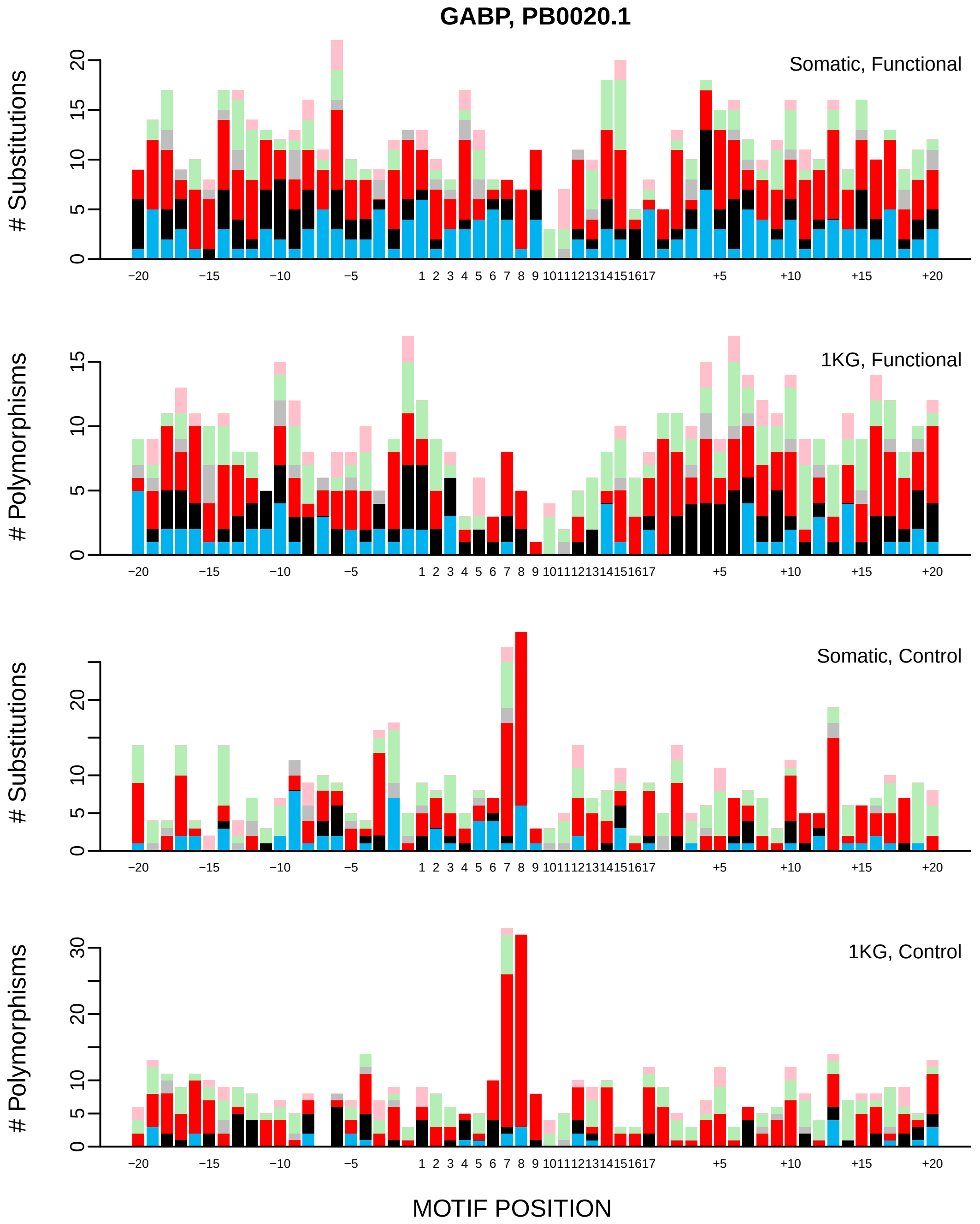

GABP, PB0020.1
Somatic, Functional
Substitutions
1KG, Functional
Polymorphisms
Somatic, Control
Substitutions
1KG, Control
Polymorphisms
MOTIF POSITION

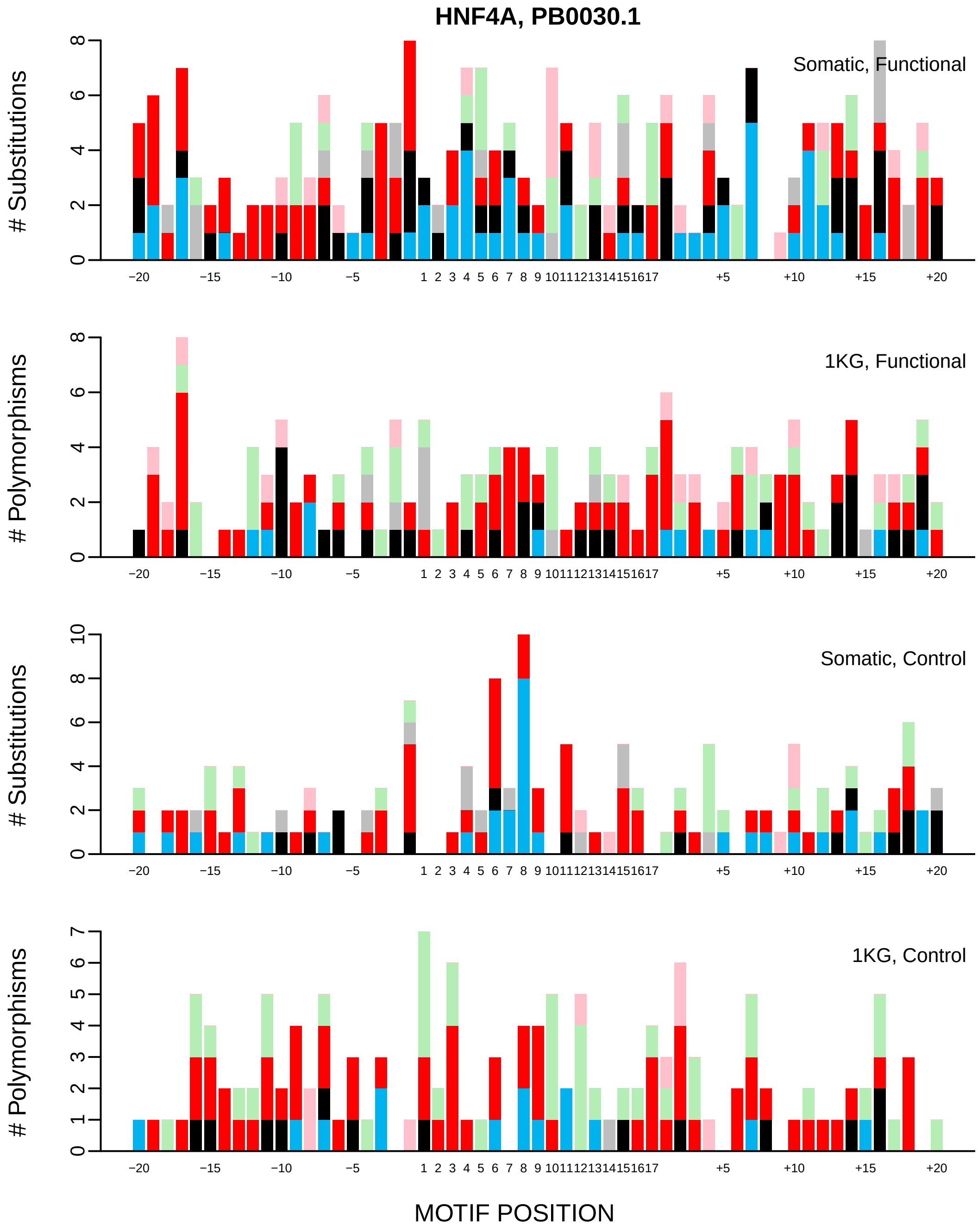
HNF4A, PB0030.1
Substitutions
Somatic, Functional
Polymorphisms
1KG, Functional
Substitutions
Somatic, Control
Polymorphisms
1KG, Control
−20 −15 −10 −5 1 2 3 4 5 6 7 8 9 10 11 12 13 14 15 16 17 +5 +10 +15 +20
MOTIF POSITION

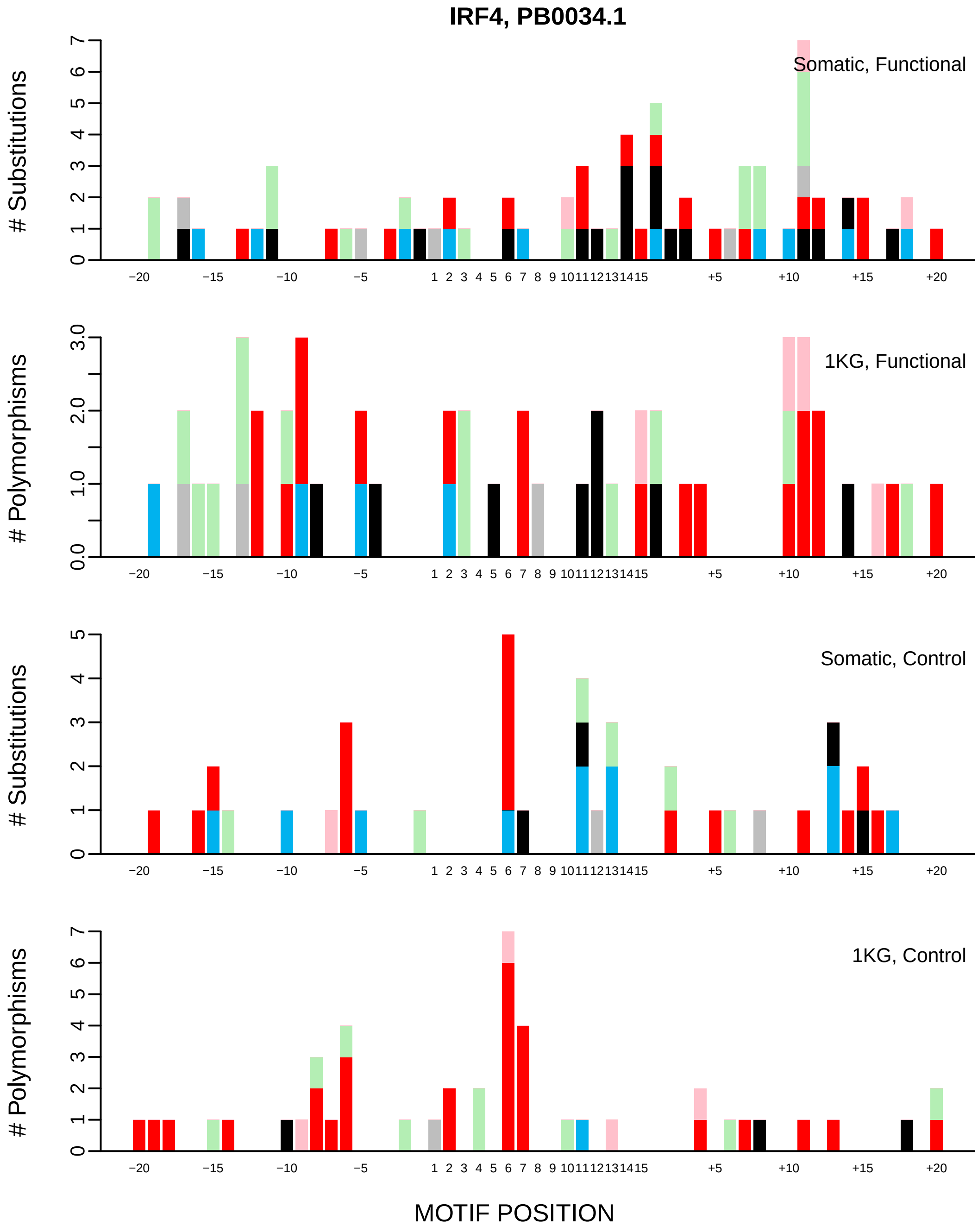
IRF4, PB0034.1
Substitutions
Somatic, Functional
Polymorphisms
1KG, Functional
Substitutions
Somatic, Control
Polymorphisms
1KG, Control
−20 −15 −10 −5 1 2 3 4 5 6 7 8 9 10 11 12 13 14 15 +5 +10 +15 +20
MOTIF POSITION

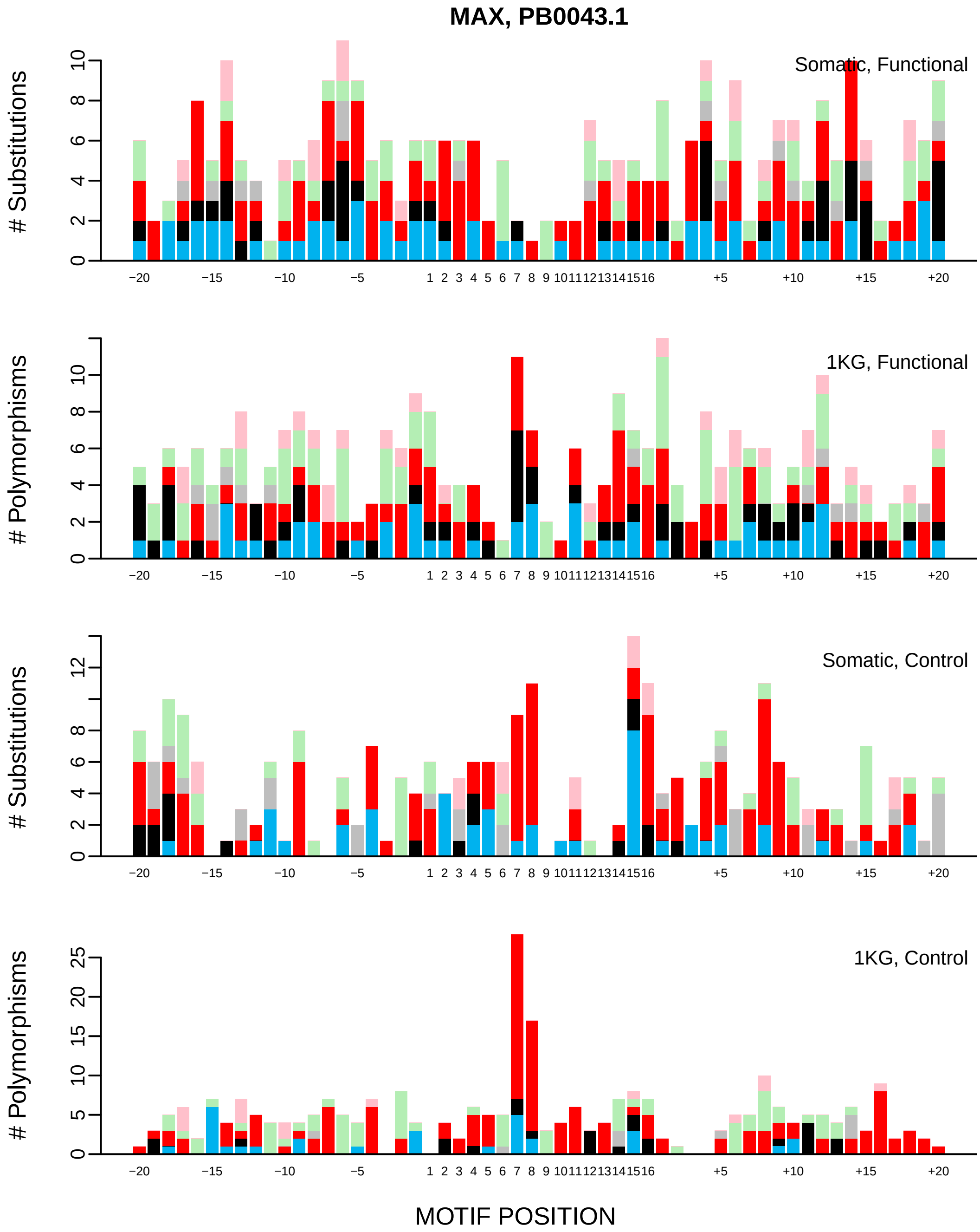
MAX, PB0043.1
Substitutions
Somatic, Functional
Polymorphisms
1KG, Functional
Substitutions
Somatic, Control
Polymorphisms
1KG, Control
−20
−15
−10
−5
1 2 3 4 5 6 7 8 9 10 11 12 13 14 15 16
+5
+10
+15
+20
MOTIF POSITION

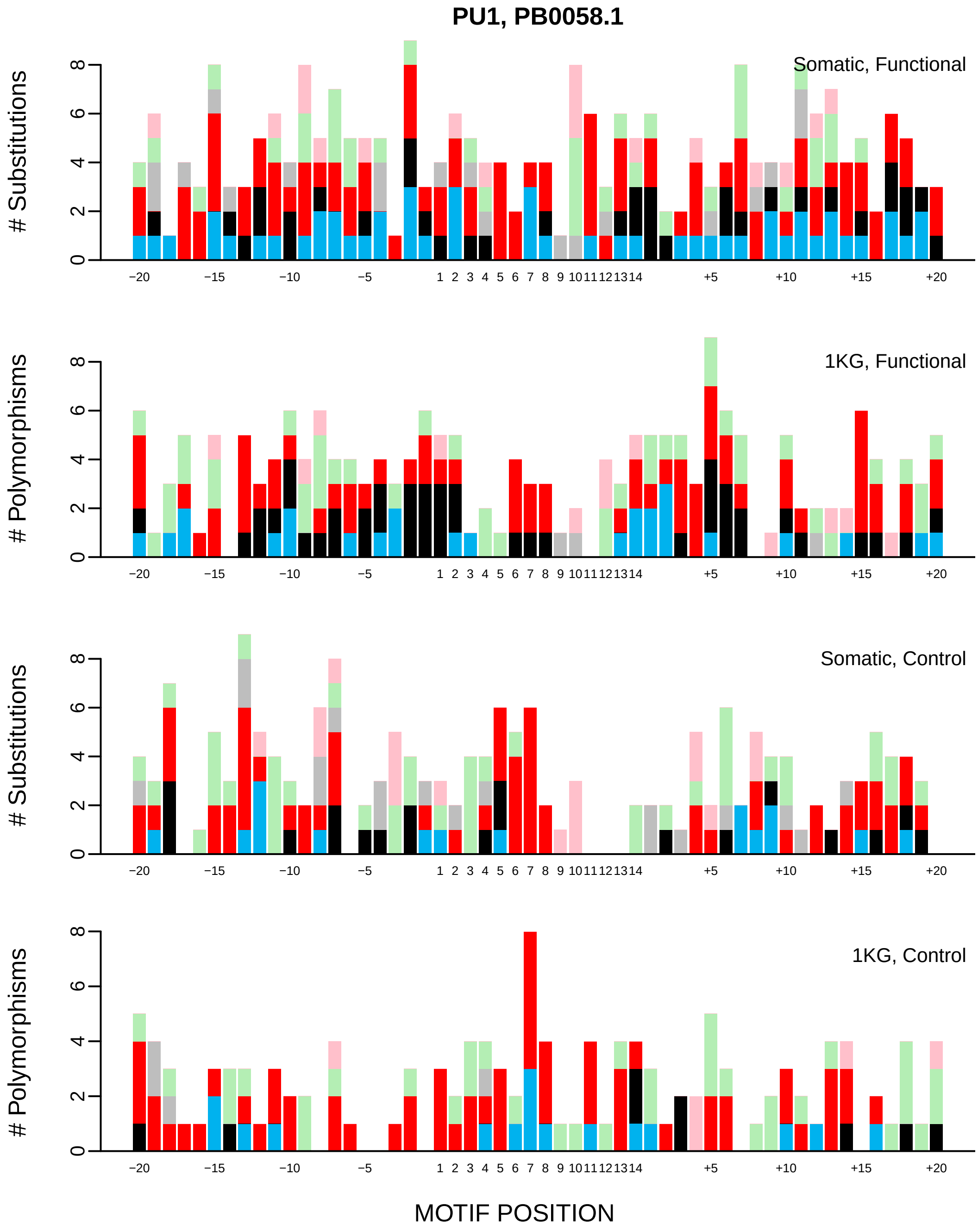
PU1, PB0058.1
Substitutions
Somatic, Functional
Polymorphisms
1KG, Functional
Substitutions
Somatic, Control
Polymorphisms
1KG, Control
MOTIF POSITION

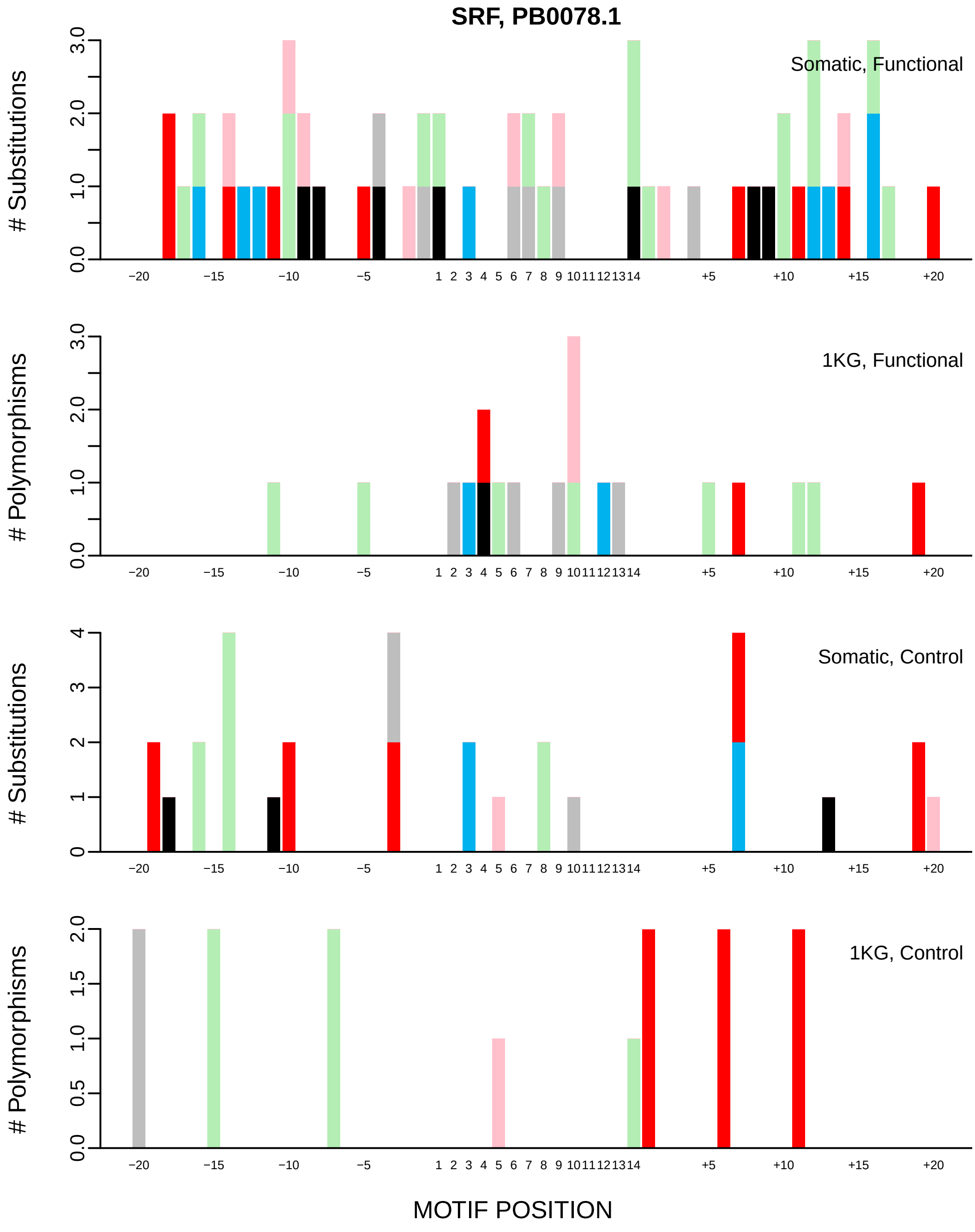
SRF, PB0078.1
Substitutions
Somatic, Functional
Polymorphisms
1KG, Functional
Substitutions
Somatic, Control
Polymorphisms
1KG, Control
−20 −15 −10 −5 1 2 3 4 5 6 7 8 9 10 11 12 13 14 +5 +10 +15 +20
MOTIF POSITION

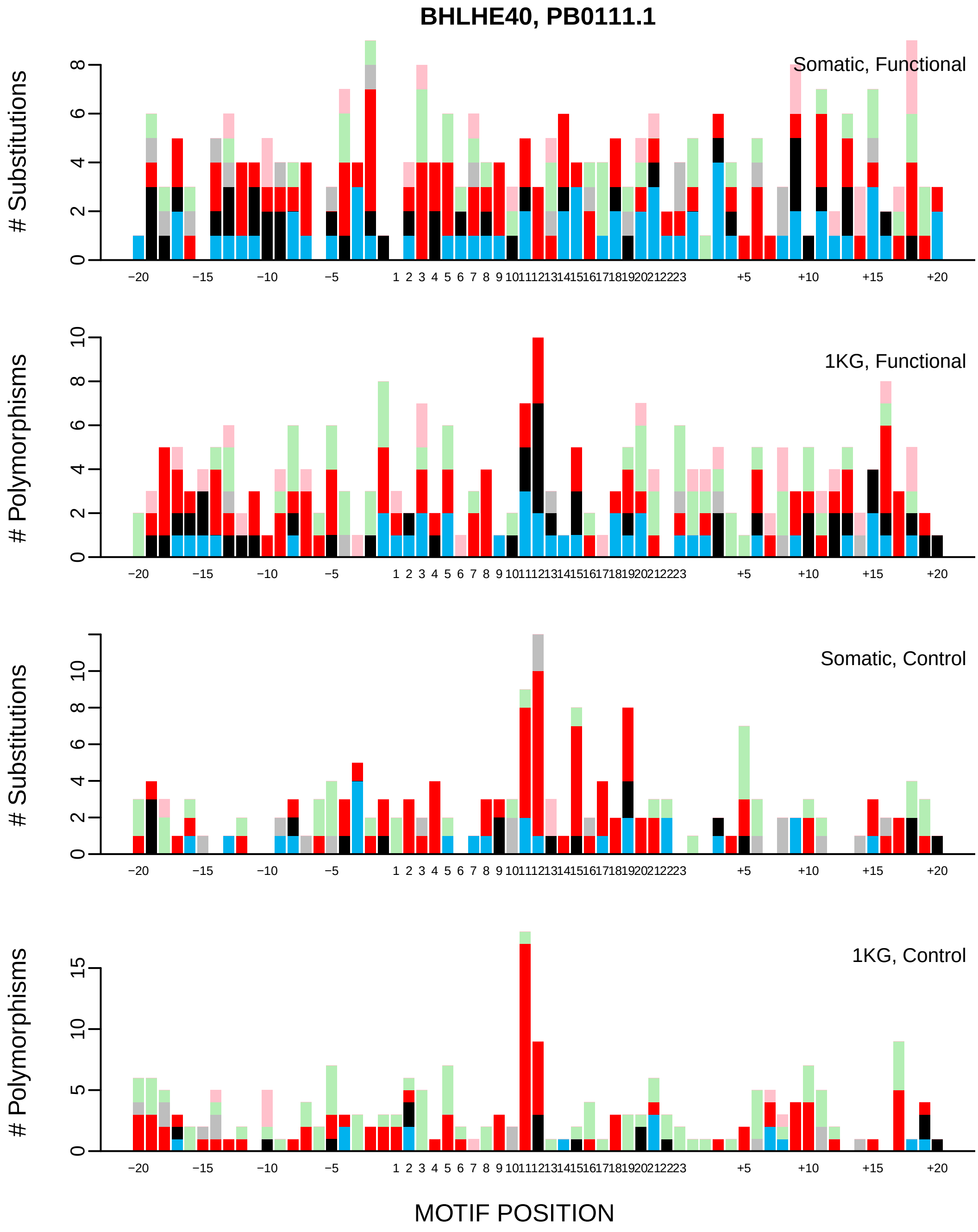
BHLHE40, PB0111.1
Somatic, Functional
1KG, Functional
Somatic, Control
1KG, Control
Substitutions
Polymorphisms
−20
−15
−10
−5
1 2 3 4 5 6 7 8 9 10 11 12 13 14 15 16 17 18 19 20 21 22 23
+5
+10
+15
+20
MOTIF POSITION

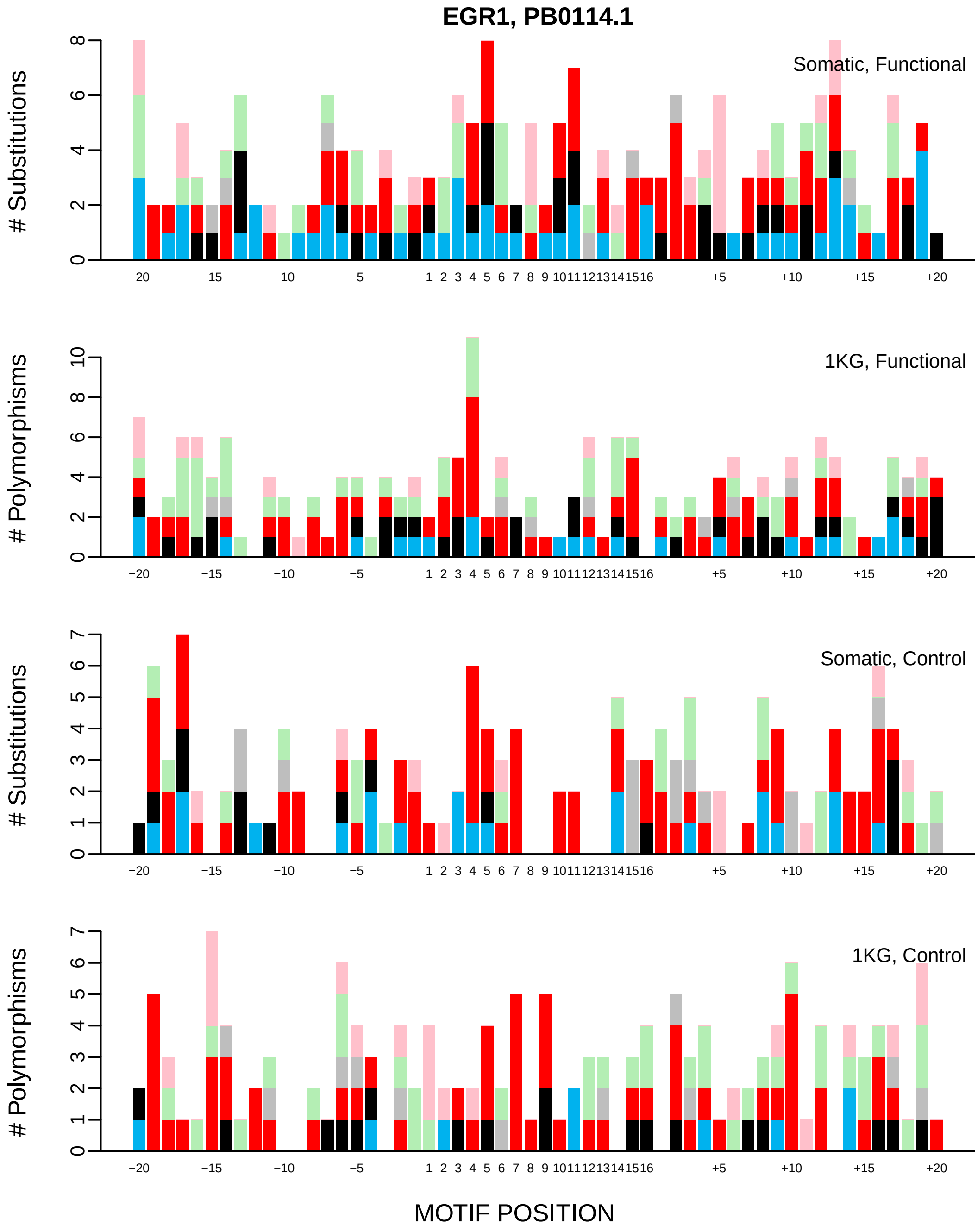

EGR1, PB0114.1
Substitutions
Somatic, Functional
Polymorphisms
1KG, Functional
Substitutions
Somatic, Control
Polymorphisms
1KG, Control
−20 −15 −10 −5 1 2 3 4 5 6 7 8 9 10 11 12 13 14 15 16 +5 +10 +15 +20
MOTIF POSITION

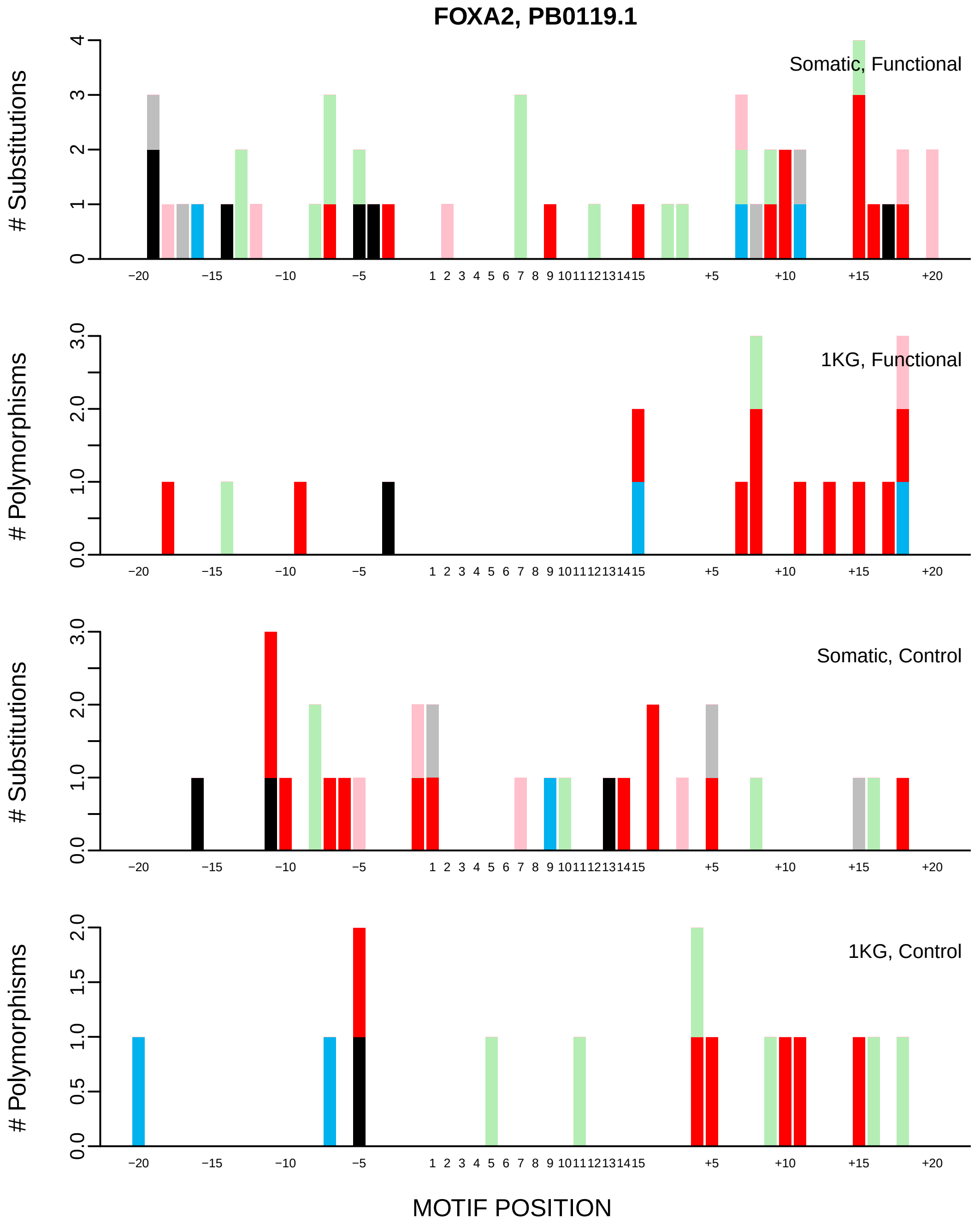
FOXA2, PB0119.1
Substitutions
Somatic, Functional
Polymorphisms
1KG, Functional
Substitutions
Somatic, Control
Polymorphisms
1KG, Control
−20
−15
−10
−5
1 2 3 4 5 6 7 8 9 10 11 12 13 14 15
+5
+10
+15
+20
MOTIF POSITION

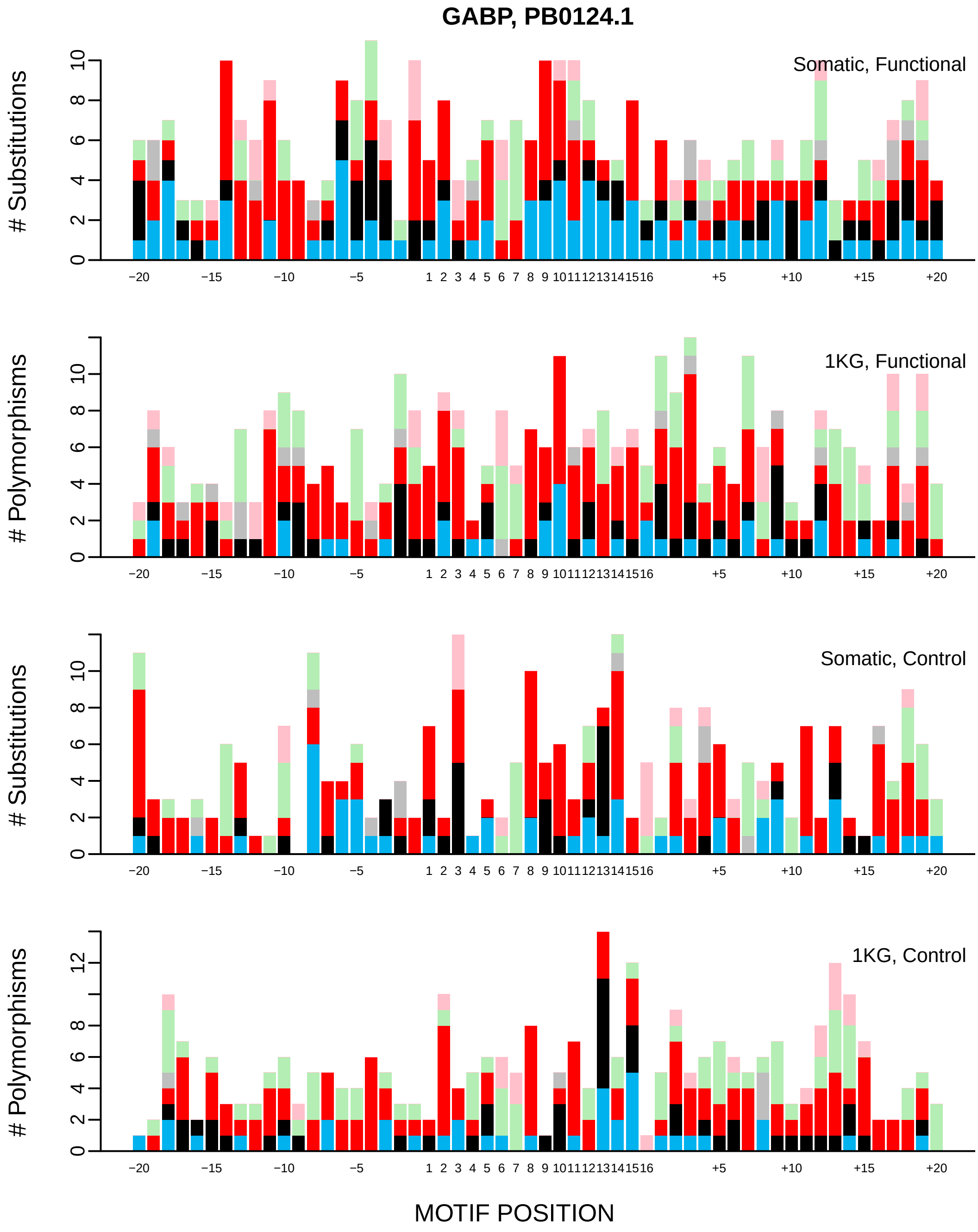
GABP, PB0124.1
Substitutions
Somatic, Functional
Polymorphisms
1KG, Functional
Substitutions
Somatic, Control
Polymorphisms
1KG, Control
MOTIF POSITION

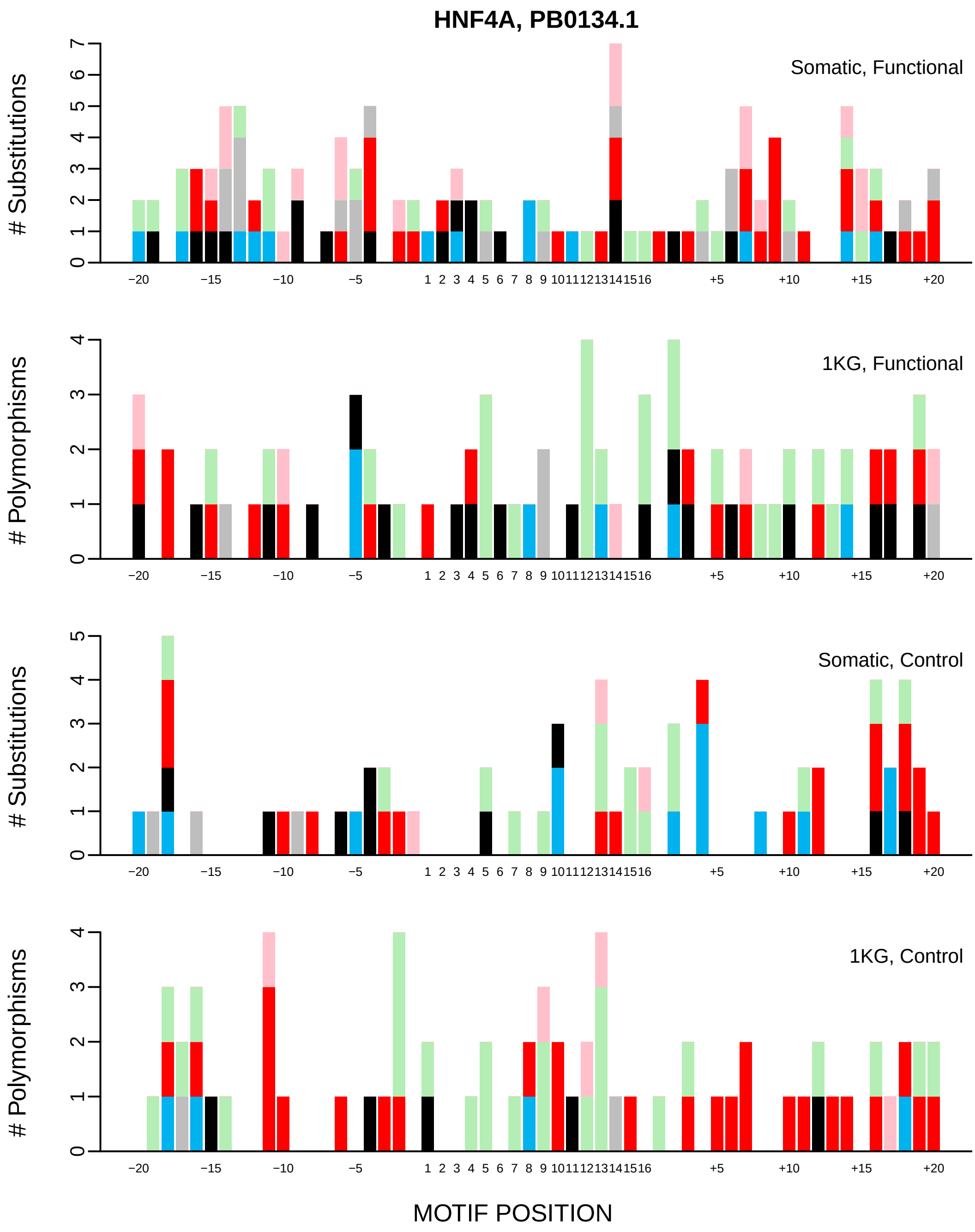
HNF4A, PB0134.1
Substitutions
Somatic, Functional
Polymorphisms
1KG, Functional
Substitutions
Somatic, Control
Polymorphisms
1KG, Control
−20
−15
−10
−5
1 2 3 4 5 6 7 8 9 10 11 12 13 14 15 16
+5
+10
+15
+20
MOTIF POSITION

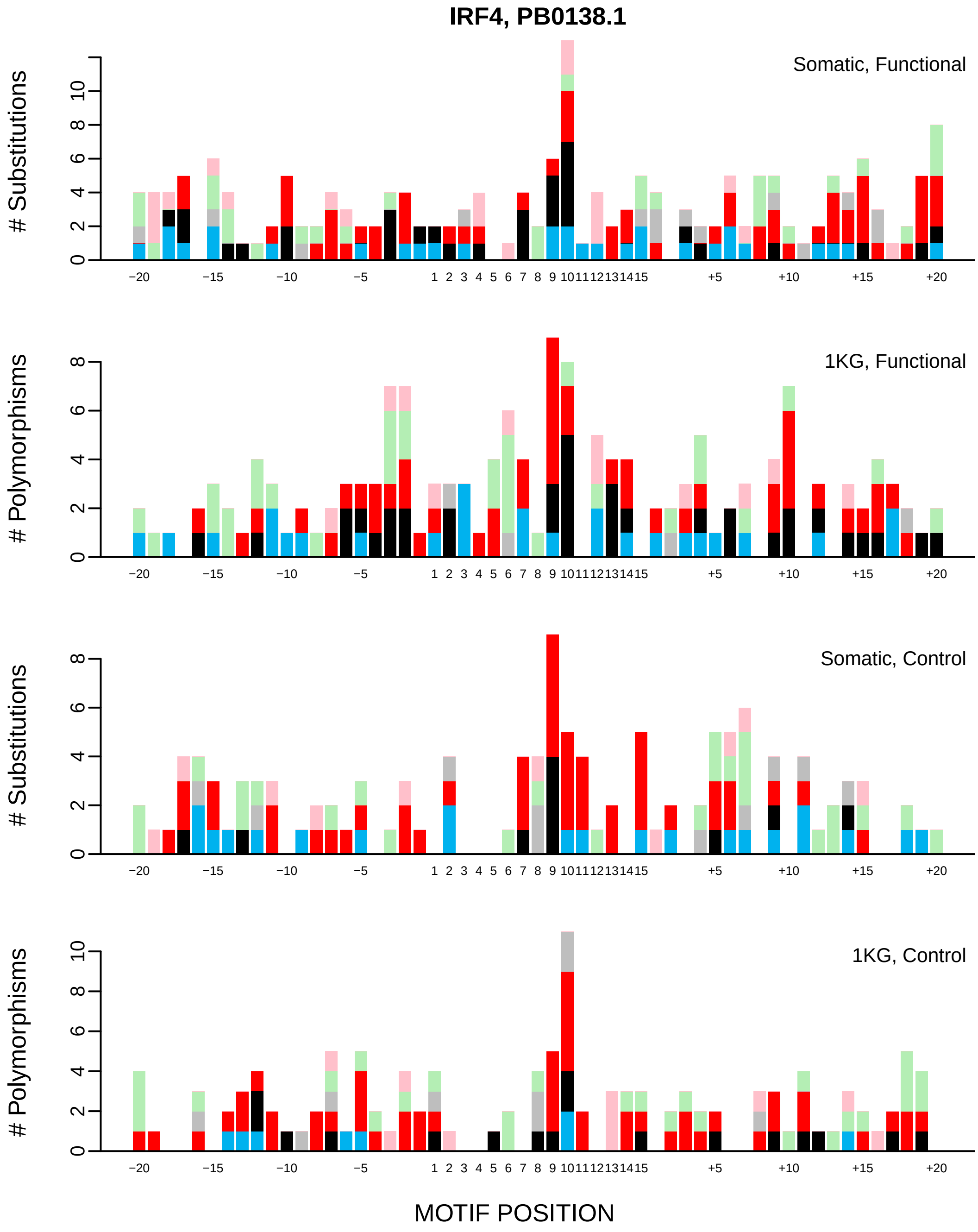
IRF4, PB0138.1
Somatic, Functional
1KG, Functional
Somatic, Control
1KG, Control
Substitutions
Polymorphisms
−20 −15 −10 −5 1 2 3 4 5 6 7 8 9 10 11 12 13 14 15 +5 +10 +15 +20
MOTIF POSITION

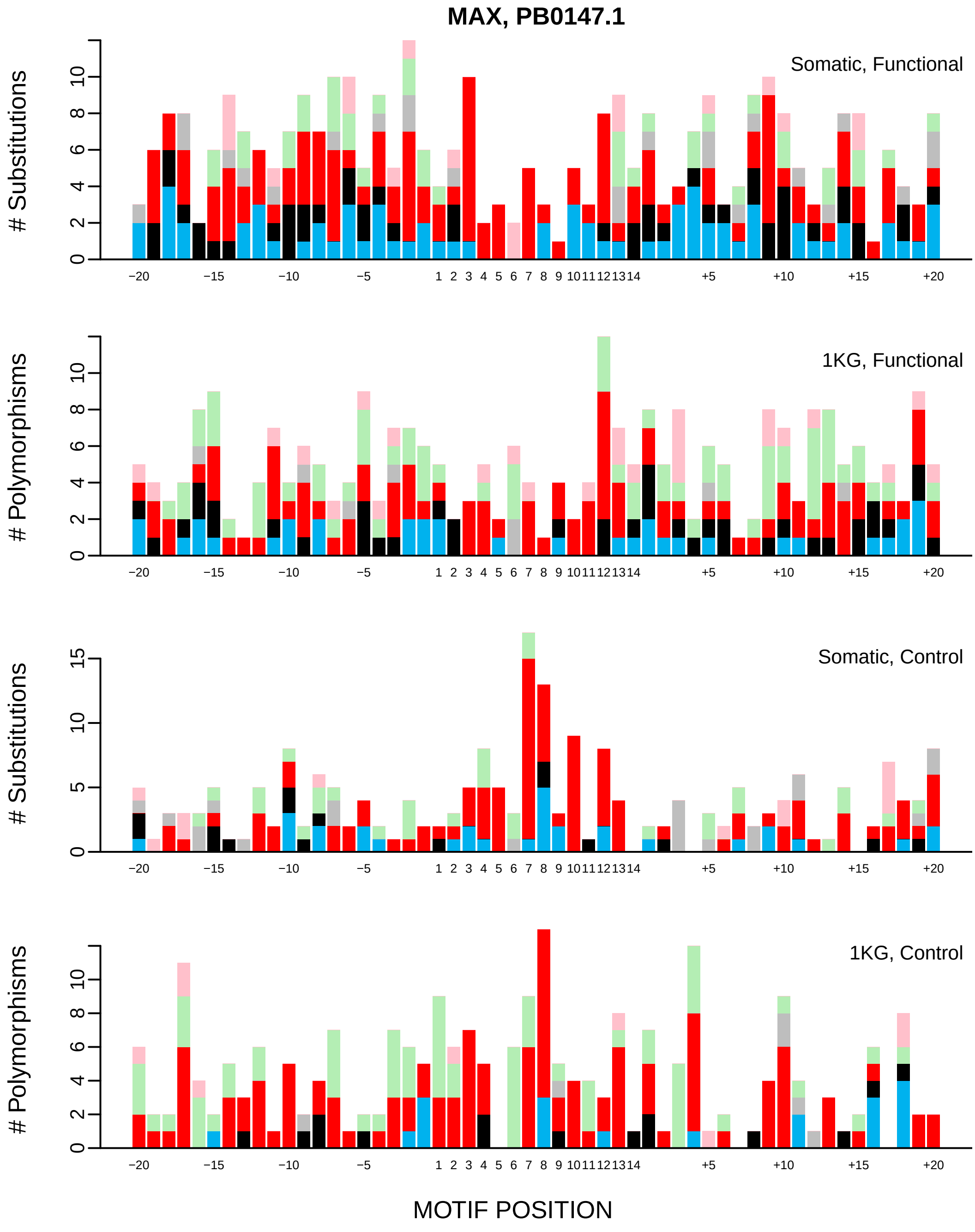
MAX, PB0147.1
Substitutions
Somatic, Functional
Polymorphisms
1KG, Functional
Substitutions
Somatic, Control
Polymorphisms
1KG, Control
−20 −15 −10 −5 1 2 3 4 5 6 7 8 9 10 11 12 13 14 +5 +10 +15 +20
MOTIF POSITION

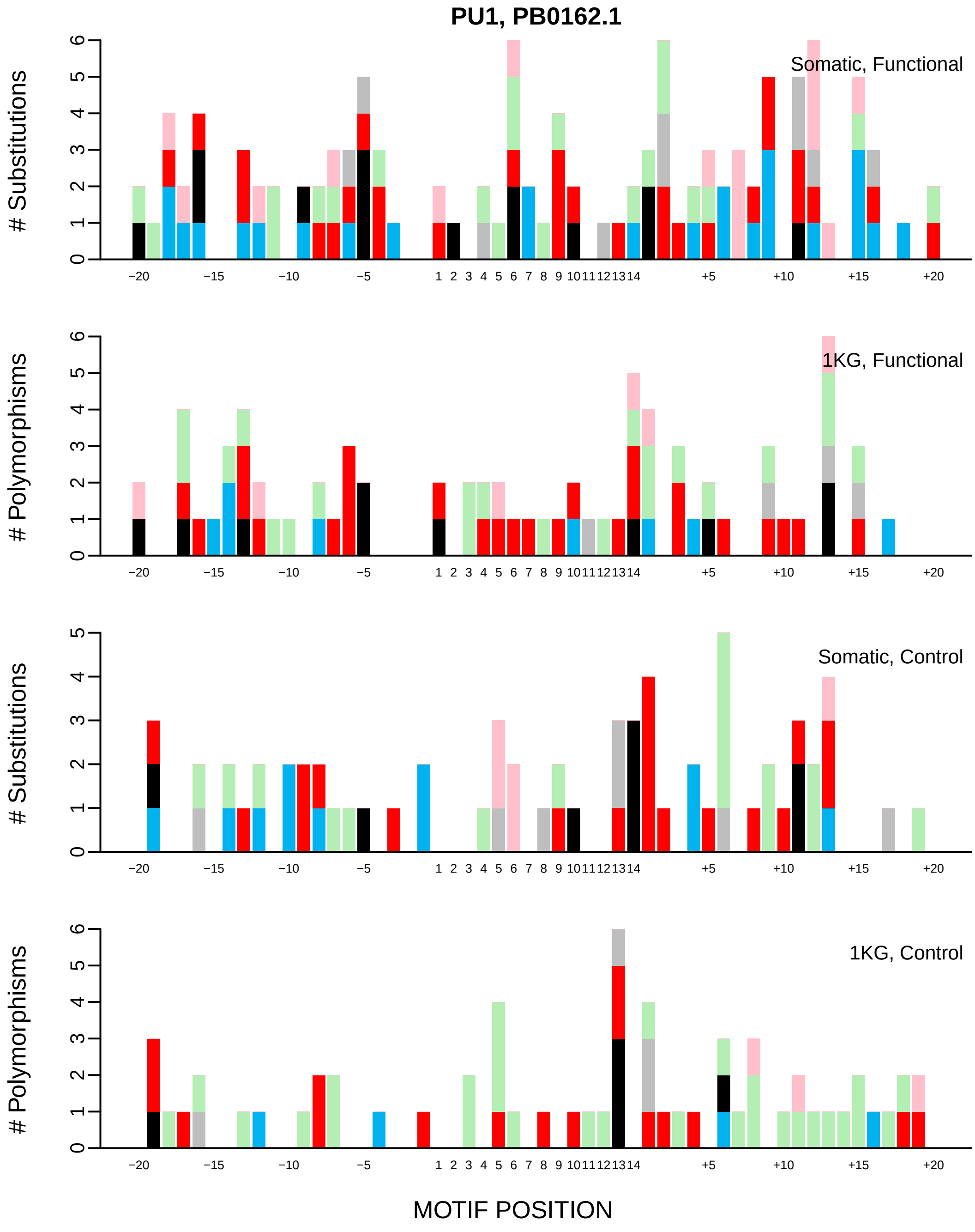

PU1, PB0162.1
Substitutions
Somatic, Functional
Polymorphisms
1KG, Functional
Substitutions
Somatic, Control
Polymorphisms
1KG, Control
−20 −15 −10 −5 1 2 3 4 5 6 7 8 9 10 11 12 13 14 +5 +10 +15 +20
MOTIF POSITION

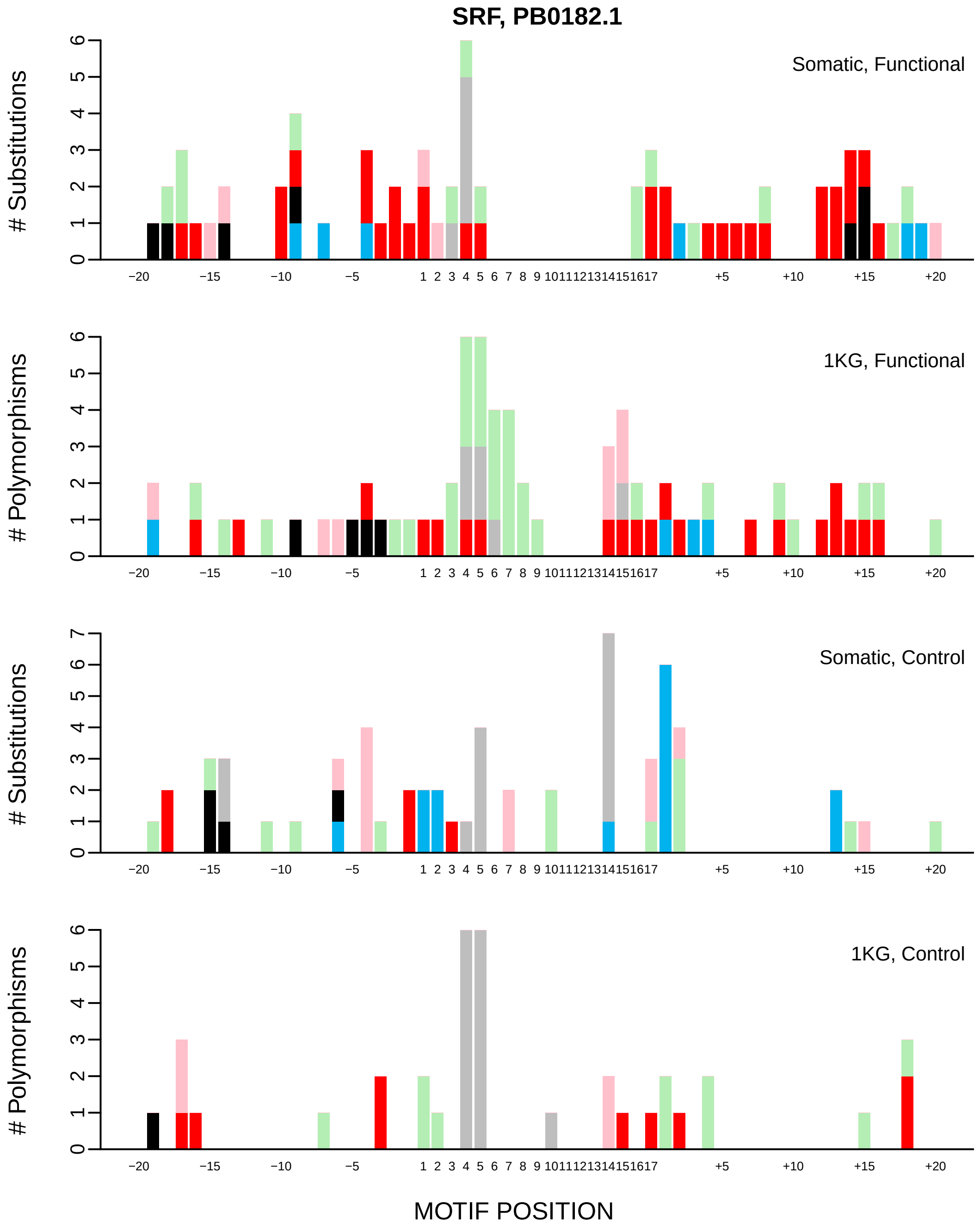
SRF, PB0182.1
Substitutions
Somatic, Functional
Polymorphisms
1KG, Functional
Substitutions
Somatic, Control
Polymorphisms
1KG, Control
−20
−15
−10
−5
1 2 3 4 5 6 7 8 9 1011121314151617
+5
+10
+15
+20
MOTIF POSITION
