# Supplementary material for: Mutational Biases Drive Elevated Rates of Substitution at Regulatory Sites across Cancer Types
Source: PLoS Genet. 2016 Aug 4;12(8):e1006207. doi: 10.1371/journal.pgen.1006207 (PMC4973979; doi:10.1371/journal.pgen.1006207)
Supplement: S1 Table — (DOCX) [file pgen.1006207.s010.docx]

**­­**

| Mutation Count | Somatic | 1KG |
| --- | --- | --- |
|  |  |  |
| Functional | 8,544 | 5,597 |
|  |  |  |
| Control | 7,613 | 7,502 |
|  |  |  |
