# Supplementary material for: Mutational Biases Drive Elevated Rates of Substitution at Regulatory Sites across Cancer Types
Source: PLoS Genet. 2016 Aug 4;12(8):e1006207. doi: 10.1371/journal.pgen.1006207 (PMC4973979; doi:10.1371/journal.pgen.1006207)
Supplement: S2 Table — (DOCX) [file pgen.1006207.s011.docx]

| **Spectrum** | **# Samples** | **Tissue**  **(# Samples)** | **# Mutations in functional TFBSs** |
| --- | --- | --- | --- |
| 1 | 19 | Lung (18)  Pancreas (1) | 1,257 |
| 2 | 10 | Lymphoma B-cell (8) Pancreas (2) | 144 |
| 3 | 227 | Liver (188)  Breast (20)  Pancreas (13) LungAdeno (2) LymphomaB-Cell (2)  Prostate (2) | 2,492 |
| 4 | 9 | Breast (7)  ALL (1)  LungAdeno (1) | 44 |
| 5 | 109 | Pancreas (84)  Liver (8)  LymphomaB-Cell (6) Prostate (6) LungAdeno (3)  Breast (1) Medulloblastoma (1) | 2,392 |
