# Supplementary material for: Mutational Biases Drive Elevated Rates of Substitution at Regulatory Sites across Cancer Types
Source: PLoS Genet. 2016 Aug 4;12(8):e1006207. doi: 10.1371/journal.pgen.1006207 (PMC4973979; doi:10.1371/journal.pgen.1006207)
Supplement: S3 Table — (DOCX) [file pgen.1006207.s012.docx]

| **Group** |  |  | **Functional Sites** | **Control**  **Sites** | **Ratio** | **p-value** |
| --- | --- | --- | --- | --- | --- | --- |
| 1 |  |  | 63 | 75 | 0.84 | N.S. |
| 2 |  |  | 33 | 5 | 6.6 | < 10-4 |
| 3 |  |  | 702 | 120 | 5.85 | < 10-4 |
| 4 |  |  | 18 | 18 | 1.00 | N.S. |
| 5 |  |  | 238 | 142 | 1.67 | < 10-4 |
