# Supplementary material for: Mutational Biases Drive Elevated Rates of Substitution at Regulatory Sites across Cancer Types
Source: PLoS Genet. 2016 Aug 4;12(8):e1006207. doi: 10.1371/journal.pgen.1006207 (PMC4973979; doi:10.1371/journal.pgen.1006207)
Supplement: S4 Table — ntx = non-transcribed strand; tx = transcribed strand. Column 2 shows the counts of the number of A:T basepairs subject to mutation at CTCF-sites (top) and genome-wide (bottom) within unidirectionally transcribed regions; column 3 lists the number of observed A:T>G:C mutations. Strand asymmetry, calculated as in Haradhvala et al. [23], is shown in the last column. (DOCX) [file pgen.1006207.s013.docx]

|  |  | **# bp (A:T) x # Samples** | **#Mutations** | **#Mutations per MB** | **Asymmetry = log2(r1/r2)** |
| --- | --- | --- | --- | --- | --- |
| **CTCF TFBSs** |  |  |  |  |  |
| “A” on ntx, “T” on tx |  | 4,152,960 | 54 | 13.00 (=r1) | 0.44 |
| “A” on tx, “T” on ntx |  | 4,586,715 | 44 | 9.59 (=r2) |  |
|  |  |  |  |  |  |
| **Genome-wide** |  |  |  |  |  |
| “A” on ntx, “T” on tx |  | 1.04*10^11^ | 263,063 | 2.53 (=r1) | 0.83 |
| “A” on tx, “T” on ntx |  | 1.14*10^11^ | 161,250 | 1.42 (=r2) |  |
|  |  |  |  |  |  |
