# Supplementary material for: Mutational Biases Drive Elevated Rates of Substitution at Regulatory Sites across Cancer Types
Source: PLoS Genet. 2016 Aug 4;12(8):e1006207. doi: 10.1371/journal.pgen.1006207 (PMC4973979; doi:10.1371/journal.pgen.1006207)
Supplement: S5 Table — (DOCX) [file pgen.1006207.s014.docx]

**a) Functional Motifs**

| **Cell Line:** | **GM12878** | **HeLa** | **HMEC** | **HUVEC** | **IMR90** | **K562** | **KBM7** | **NHEK** |
| --- | --- | --- | --- | --- | --- | --- | --- | --- |
|  |  |  |  |  |  |  |  |  |
| # mutated, inside Anchor | 549 | 339 | 421 | 407 | 508 | 518 | 304 | 428 |
| # mutated, outside Anchor | 443 | 644 | 562 | 576 | 475 | 465 | 679 | 555 |
| # not mutated, inside Anchor | 4081 | 2485 | 3071 | 2971 | 3975 | 3714 | 2120 | 2877 |
| # not mutated, outside Anchor | 5690 | 7295 | 6709 | 6809 | 5805 | 6066 | 7660 | 6903 |
|  |  |  |  |  |  |  |  |  |
| fraction mutated, inside Anchor | 0.12 | 0.12 | 0.12 | 0.12 | 0.11 | 0.12 | 0.13 | 0.13 |
| fraction mutated, outside Anchor | 0.07 | 0.08 | 0.08 | 0.08 | 0.08 | 0.07 | 0.08 | 0.07 |
|  |  |  |  |  |  |  |  |  |
| χ^2^ *p*-value | < 2.2E-16 | 8.86E-10 | 3.89E-13 | 1.61E-12 | 2.82E-11 | 2.20E-16 | 4.79E-11 | < 2.2E-16 |
| **b) Control Motifs** |  |  |  |  |  |  |  |  |
| **Cell Line:** | **GM12878** | **HeLa** | **HMEC** | **HUVEC** | **IMR90** | **K562** | **KBM7** | **NHEK** |
|  |  |  |  |  |  |  |  |  |
| # mutated, inside Anchor | 69 | 67 | 54 | 105 | 91 | 88 | 70 | 94 |
| # mutated, outside Anchor | 387 | 389 | 402 | 351 | 365 | 368 | 386 | 362 |
| # not mutated, inside Anchor | 1801 | 1777 | 1467 | 2161 | 1921 | 2382 | 1665 | 2209 |
| # not mutated, outside Anchor | 8506 | 8530 | 8840 | 8146 | 8386 | 7925 | 8642 | 8098 |
|  |  |  |  |  |  |  |  |  |
| fraction mutated, inside Anchor | 0.04 | 0.04 | 0.04 | 0.05 | 0.05 | 0.04 | 0.04 | 0.04 |
| fraction mutated, outside Anchor | 0.04 | 0.04 | 0.04 | 0.04 | 0.04 | 0.04 | 0.04 | 0.04 |
|  |  |  |  |  |  |  |  |  |
| χ^2^ *p*-value | N.S. | N.S. | N.S. | N.S. | N.S. | N.S. | N.S. | N.S. |
