# Supplementary material for: Mutational Biases Drive Elevated Rates of Substitution at Regulatory Sites across Cancer Types
Source: PLoS Genet. 2016 Aug 4;12(8):e1006207. doi: 10.1371/journal.pgen.1006207 (PMC4973979; doi:10.1371/journal.pgen.1006207)
Supplement: S6 Table — (DOCX) [file pgen.1006207.s015.docx]

| **Tissue** | **# CTCF-Mutations** | **# CTCF-Mutations/**  **# Individuals** |
| --- | --- | --- |
| ALL | 1 | 1.00 |
| AML | 0 | 0.00 |
| Breast | 87 | 0.73 |
| CLL | 0 | 0.00 |
| Liver | 796 | 2.53 |
| LungAdeno | 63 | 2.63 |
| LymphomaB-cell | 45 | 0.66 |
| Medulloblastoma | 10 | 0.10 |
| Pancreas | 282 | 0.66 |
| PilocyticAstrocytoma | 10 | 0.03 |
| Prostate | 37 | 0.26 |
