# Supplementary material for: Mutational Biases Drive Elevated Rates of Substitution at Regulatory Sites across Cancer Types
Source: PLoS Genet. 2016 Aug 4;12(8):e1006207. doi: 10.1371/journal.pgen.1006207 (PMC4973979; doi:10.1371/journal.pgen.1006207)
Supplement: S7 Table — CTCF-motifs, which were found to be mutated in our dataset, were compared to a background set of all functional CTCF-motifs. (DOCX) [file pgen.1006207.s016.docx]

| **Category** | [**Name**](http://bejerano.stanford.edu/great/public/cgi-bin/yui-dt1-href-Desc) | **Raw P-Value** | **FDR Q-Val** | **Fold Enrichment** | **Foreground Region Hits** | **Total Regions** | **Region Set Coverage** | **Foreground Gene Hits** | **Genes Annotated** |
| --- | --- | --- | --- | --- | --- | --- | --- | --- | --- |
|  |  |  |  |  |  |  |  |  |  |
| **GO Biological Process** | regulation of peptide hormone secretion | 5.64E-06 | 2.94E-02 | 2.1035 | 39 | 203 | 3.97% | 33 | 164 |
|  | regulation of insulin secretion | 9.52E-06 | 1.99E-02 | 2.1306 | 36 | 185 | 3.66% | 30 | 151 |
|  | regulation of peptide secretion | 1.16E-05 | 2.01E-02 | 2.0431 | 39 | 209 | 3.97% | 33 | 168 |
|  | regulation of peptide transport | 1.63E-05 | 2.43E-02 | 2.0142 | 39 | 212 | 3.97% | 33 | 170 |
|  |  |  |  |  |  |  |  |  |  |
| **MSigDB Perturbation** | Down-regulated genes predicting poor survival of patients with thyroid carcinoma.* | 3.11E-05 | 1.49E-02 | 5.4746 | 8 | 16 | 0.81% | 5 | 10 |
|  | Genes up-regulated in kidney biopsies from patients with acute transplant rejection compared to the biopsies from patients with well functioning kidneys more than 1-year post transplant. | 4.77E-05 | 2.00E-02 | 2.4833 | 22 | 97 | 2.24% | 15 | 86 |
|  | Genes up-regulated in SaOS-2 cells (osteosarcoma) upon expression of PAX3-FOXO1 [GeneID=5077;2308] fusion protein off an adenoviral vector. | 1.60E-04 | 4.50E-02 | 2.8317 | 15 | 58 | 1.53% | 11 | 60 |
|  | Top 200 genes with high histone H3 trimethylation mark at K27 (H3K27me3) in PC3 cells (prostate cancer), by ChIP-chip assay on an 88K microarray (all promoters). | 1.80E-04 | 4.34E-02 | 2.0819 | 27 | 142 | 2.75% | 24 | 188 |
|  |  |  |  |  |  |  |  |  |  |
| **MSigDB Oncogenic Signatures** | Genes up-regulated in epithelial kidney cancer cell lines over-expressing an oncogenic form of KRAS [Gene ID=3845] gene. | 2.84E-05 | 5.30E-03 | 2.0797 | 34 | 179 | 3.46% | 28 | 136 |

* Includes APLP2, LMBRD1, SDK2, SH3BGRL2, TMEM243
