# Supplementary material for: Mutational Biases Drive Elevated Rates of Substitution at Regulatory Sites across Cancer Types
Source: PLoS Genet. 2016 Aug 4;12(8):e1006207. doi: 10.1371/journal.pgen.1006207 (PMC4973979; doi:10.1371/journal.pgen.1006207)
Supplement: S8 Table — Shown are the number of genes/their motifs that are in common between each category. Binding motif of hybrid factors, such as TAL1::GATA1 were excluded from this analysis. (DOCX) [file pgen.1006207.s017.docx]

|  | Binding site mutational excess | No binding site mutational excess |
| --- | --- | --- |
|  |  |  |
| In cancer5000 | 1 | 10 |
| Not in cancer5000 | 12 | 88 |
