# Supplementary material for: Mutational Biases Drive Elevated Rates of Substitution at Regulatory Sites across Cancer Types
Source: PLoS Genet. 2016 Aug 4;12(8):e1006207. doi: 10.1371/journal.pgen.1006207 (PMC4973979; doi:10.1371/journal.pgen.1006207)
Supplement: S9 Table — (DOCX) [file pgen.1006207.s018.docx]

| Functional TFBSs | CrgMapabilityAlign100mer | CrgMapabilityAlign36mer | DukeMapabilityUniqueness35bp |
| --- | --- | --- | --- |
| Median | 1.00 | 1.00 | 1.00 |
| Mean | 1.00 | 0.99 | 1.00 |
| 5% percentile | 1.00 | 1.00 | 1.00 |
| 1% percentile | 1.00 | 0.50 | 1.00 |
|  |  |  |  |
|  |  |  |  |
| Control TFBSs | CrgMapabilityAlign100mer | CrgMapabilityAlign36mer | DukeMapabilityUniqueness35bp |
| Median | 1.00 | 1.00 | 1.00 |
| Mean | 0.99 | 0.96 | 0.98 |
| 5% percentile | 1.00 | 0.50 | 1.00 |
| 1% percentile | 0.50 | 0.15 | 0.41 |
